# Distinct Cerebrospinal Fluid Proteomes Differentiate Post-Treatment Lyme Disease from Chronic Fatigue Syndrome

**DOI:** 10.1371/journal.pone.0017287

**Published:** 2011-02-23

**Authors:** Steven E. Schutzer, Thomas E. Angel, Tao Liu, Athena A. Schepmoes, Therese R. Clauss, Joshua N. Adkins, David G. Camp, Bart K. Holland, Jonas Bergquist, Patricia K. Coyle, Richard D. Smith, Brian A. Fallon, Benjamin H. Natelson

**Affiliations:** 1 Department of Medicine, University of Medicine and Dentistry of New Jersey-New Jersey Medical School, Newark, New Jersey, United States of America; 2 Department of Neurology, University of Medicine and Dentistry of New Jersey-New Jersey Medical School, Newark, New Jersey, United States of America; 3 Division of Biostatistics and Epidemiology, University of Medicine and Dentistry of New Jersey-New Jersey Medical School, Newark, New Jersey, United States of America; 4 Biological Sciences Division, Pacific Northwest National Laboratory, Richland, Washington, United States of America; 5 Department of Physical and Analytical Chemistry, Uppsala University, Uppsala, Sweden; 6 Department of Neurology, State University of New York-Stony Brook, Stony Brook, New York, United States of America; 7 Department of Psychiatry, Columbia University Medical Center, New York, New York, United States of America; 8 Department of Pain Medicine and Palliative Care and Beth Israel Medical Center, Albert Einstein School of Medicine, Bronx, New York, United States of America; University of Nebraska, United States of America

## Abstract

**Background:**

Neurologic Post Treatment Lyme disease (*nPTLS*) and Chronic Fatigue (*CFS*) are syndromes of unknown etiology. They share features of fatigue and cognitive dysfunction, making it difficult to differentiate them. Unresolved is whether *nPTLS* is a subset of *CFS*.

**Methods and Principal Findings:**

Pooled cerebrospinal fluid (CSF) samples from *nPTLS patients*, *CFS* patients, and healthy volunteers were comprehensively analyzed using high-resolution mass spectrometry (MS), coupled with immunoaffinity depletion methods to reduce protein-masking by abundant proteins. Individual patient and healthy control CSF samples were analyzed directly employing a MS-based label-free quantitative proteomics approach. We found that both groups, and individuals within the groups, could be distinguished from each other and normals based on their specific CSF proteins (p<0.01). *CFS* (n = 43) had 2,783 non-redundant proteins, *nPTLS* (n = 25) contained 2,768 proteins, and healthy normals had 2,630 proteins. Preliminary pathway analysis demonstrated that the data could be useful for hypothesis generation on the pathogenetic mechanisms underlying these two related syndromes.

**Conclusions:**

*nPTLS* and *CFS* have distinguishing CSF protein complements. Each condition has a number of CSF proteins that can be useful in providing candidates for future validation studies and insights on the respective mechanisms of pathogenesis. Distinguishing *nPTLS* and *CFS* permits more focused study of each condition, and can lead to novel diagnostics and therapeutic interventions.

## Introduction

Prime objectives in studying neurologic and psychiatric disorders are to develop discriminating markers and generate data that can provide insight into disease pathogenesis. This can lead to novel treatment strategies. Chronic Fatigue Syndrome (*CFS*) and Lyme disease, particularly Neurologic Post Treatment Lyme disease syndrome (*nPTLS*), represent two conditions that share common symptoms of fatigue and cognitive dysfunction [1]–[7]. Despite extensive research *CFS* and *nPTLS* remain medically unexplained. There are no biological markers to distinguish these syndromes, creating diagnostic dilemmas and impeding research into understanding each individual syndrome.

Cerebrospinal fluid (CSF) is an ideal body fluid to examine for signature protein profiles informative for diagnosis or etiology of central nervous system (CNS)-related symptoms and dysfunction. Not only is the CSF an accessible liquid extension of the brain, but recent data suggests CSF may provide more relevant data than brain parenchyma itself in certain neurologic diseases [8]. Specific abnormalities found in CSF relating to *CFS* and *nPTLS* would suggest CNS involvement, and could facilitate their mechanistic understanding.

Liquid chromatography coupled to mass spectrometry (LC-MS) is becoming the method of choice for examining complex biological specimens, that contain hundreds to thousands of proteins [9], such as CSF [10]. This is particularly the case in the initial discovery phase. This *discovery* phase may be viewed as casting a wide net to maximize identification of as many proteins as possible in a sample. This initial list of identified proteins has value by itself for qualitative or semi-quantitative comparisons between diseases. Recent studies demonstrated the reliability and reproducibility of LC-MS results, with different mass spectrometers across different laboratories, when performed by experienced individuals [9], [11]. In a discovery phase investigation, the MS technique is unbiased and does not require prior knowledge of what proteins may be in a sample. This is in contrast to subsequent *validation* studies where targeted approaches are used and which do require knowledge of target proteins. In searching for a disease biomarker, the discovery phase should provide a list of proteins and serve as a precursor phase for targeted approaches. These subsequent targeted approaches, whether they use other MS techniques or are immuno-based, are designed to validate the use of the biomarker protein(s) to distinguish one disease from another.

In practice tailored strategies are often needed to achieve a balance between ideal and real world constraints – especially where sample volumes and numbers are limited such as with CSF. In an ideal situation it is desirable to have numerous samples from individuals with a particular disease. It is further desirable to have sufficient total protein content in each sample so that a variety of protein separation and fractionation methods can be used prior to MS analysis. This will minimize abundant proteins from masking the detection of less abundant ones, and will permit full qualitative and quantitative analyses. Limited sample numbers and quantities do not preclude employment of tailored strategies to get meaningful results. It should be remembered that in the example of a biomarker search, the protein(s) will be confirmed or dismissed in future targeted validation studies, but failure to identify them in the broad discovery list would preclude them from examination for validation.

Until recently, technical hurdles impeded the use of CSF to distinguish conditions such as *CFS* and *nPTLS*. Advances in sample preparation, separations and MS platform capabilities enabled us to recently establish a comprehensive reference normal CSF proteome [10]. This provides the basis for comparative proteome analyses with other diseases, which should provide greater insight into their underlying pathogenesis.

To address the possibility that *CFS* and *nPTLS* could be distinguished from one another and healthy subjects, we searched for distinguishing protein marker profiles by applying our advanced proteomics strategy [10] to characterize the CSF proteomes from well described *CFS* and *nPTLS* patients (detailed in Methods). We performed comparative whole CSF proteome analyses between *CFS*, *nPTLS*, and healthy normal controls, and complemented these findings with label-free quantitative analysis of individual subject samples. In addition, we performed a preliminary pathway analysis [12] using these data, to examine the feasibility of this type of tool for future investigations to probe for clues to the pathogenetic mechanisms behind these diseases.

## Materials and Methods

### Ethics Statement

Approval for the conduct of this study was obtained from the Institutional Review Board of New Jersey Medical School and the Institutional Review Board of Pacific Northwest National Laboratory (Exempt status and consent not required, using previously banked de-identified samples in accordance with federal regulations).

### Overview and Rationale

We performed analysis of pooled CSF samples allowing for a broad and deep view as well as qualitative comparison of each disease-related and control CSF proteome. To determine if these two syndromes could be quantitatively differentiated we performed a label-free quantitative analysis of protein abundances for individual subject CSF samples. Pooling samples provided sufficient protein mass for effective downstream proteomics analysis following immunoaffinity depletion of the 14 most abundant proteins present (representing approximately 95% of the total protein mass in CSF), reducing the dynamic range of protein concentrations present in CSF, where proteins with highest concentrations mask proteins at lower concentrations from detection. Coupling immunoaffinity depletion with strong cation exchange (SCX) fractionation further reduces sample complexity, and allowed for the in-depth analysis of the CSF proteomes. These comprehensive CSF proteomics datasets were then used to create an accurate mass and time (AMT) tag database for subsequent label-free quantitative analysis of individual subject CSF samples. Due to the limit in sample volume, the CSF samples used in individual LC-MS analyses were not immunoaffinity depleted and fractionated, and therefore had much lower proteome coverage compared to the pooled samples. Nevertheless, the label-free quantitative analysis of single subject samples provided a means for statistical evaluation of the quantified protein abundances for many subjects suffering from *CFS* and *nPTLS* as well as normal healthy volunteers. Together these analyses represent the discovery phase of our studies on *CFS* and *nPTLS,* generating targets for follow up verification and validation in the later stages of the biomarker discovery workflow [13].

### Cerebrospinal Fluid (CSF) specimens

#### *CFS* Subjects

Both pooled and individuals CSF samples were analyzed. Equal aliquots from individual CSF samples were pooled to provide sufficient volume for extensive fractionation and two-dimensional LC coupled to tandem MS (2D-LC-MS/MS) analysis with immunoaffinity depletion from 30 women and 13 men (n = 43) who fulfilled the 1994 case definition for *CFS* [1]. All subjects were 18–54 years old (median  = 43) and underwent a careful history and physical examination by an expert experienced in evaluating patients with medically unexplained fatigue and pain. Patients had blood tests to rule out common causes of severe fatigue such as anemia, liver disease, hypothyroidism, systemic lupus erythematosus, and Lyme disease [14]. All subjects then underwent a psychiatric diagnostic interview designed to identify major psychiatric diagnoses for exclusion in this study. Eleven of the patients were not taking medicines. Subjects then underwent lumbar puncture. CSF was sent to the laboratory for white blood cell (wbc) count and total protein [10]. A majority of *CFS* patients had normal CSF protein and cell counts (protein less than 45 mg/dl and wbc less than or equal to 5/mm^3^). Ten of the patients had increased protein values ranging from 46–93 mg/dl (with a median of 59 mg/dl) and 3 patients had minimally elevated wbc counts of 6, 7, and 9 respectively. Individual CSF samples from 14 of the 43 *CFS* subjects (aged 33–48 years with a median age of 43 years, 7 female and 7 male) were also used in direct LC-MS analysis (i.e., no MS/MS was performed) without immunoaffinity depletion. Twelve of the 14 patients had normal CSF protein levels and all had normal cell counts. All subjects provided written informed consent approved by the Institutional Review Board.

#### *nPTLS* Subjects

Both pooled and individuals CSF samples were analyzed. Equal aliquots from individual CSF samples were pooled to provide sufficient volume for extensive fractionation and 2D-LC-MS/MS analysis with immunoaffinity depletion from 15 females and 10 males (n = 25) with *nPTLS.* All were documented to have had prior Lyme disease which met CDC surveillance case definition criteria [15], persistent neurologic features, including cognitive impairment and fatigue, despite appropriate antibiotic treatment [16], [17]. Subjects were 17–64 years old (median  = 48). All were seropositive for antibodies to *B. burgdorferi* (the etiologic agent of Lyme disease). Patients, enrolled in an NIH funded study, met the following criteria [17]: (1) current positive IgG Western blot using CDC surveillance criteria assessed using a single reference laboratory (University Hospital of Stony Brook); (2) treatment for Lyme disease with at least 3 weeks of intravenous ceftriaxone or cefotaxime that was completed at least 4 months before study entry; and (3) objective evidence of memory impairment as documented by the Wechsler Memory Scale-III compared to age-, sex-and education-adjusted population norms. *nPTLS* subjects were excluded if history or testing revealed a medical condition that could cause cognitive impairment or confound neuropsychological assessment (e.g., neurological disease, autoimmune disease, unstable thyroid disease, learning disability, substance abuse, B12 deficiency). Patients with cephalosporin allergy or a history of significant psychiatric disorder prior to onset of Lyme disease were also excluded. All patients had a comprehensive battery of neurocognitive testing and a full-physical exam with detailed rheumatologic and neurologic assessments. *nPTLS* patients then had a lumbar puncture and CSF was evaluated for cell count, total protein, glucose, total gammaglobulin, oligoclonal bands and evidence of *B. burgdorferi* (ELISA, Bb DNA by PCR, and culture using BSKII medium). None had evidence of another active tick-borne disease. A majority of *nPTLS* patients included in the pooled sample had normal CSF protein and cell counts (protein less than 45 mg/dl and wbc less than or equal to 5/mm^3^), except for 3 patients who had elevated protein values of 58, 69, and 71 mg/dl respectively and 1 patient with elevated wbc count of 6. Individual CSF samples from a group of 14 of the 25 *nPTLS* subjects (aged 25–58 years with a median age of 48 years, 6 female and 8 male) were also used in direct LC-MS analysis without immunoaffinity depletion. Two of the 14 patients had increased CSF protein levels of 69 and 71 mg/dl and 1 had a slightly elevated wbc of 6. All subjects provided written informed consent approved by the Institutional Review Board.

#### Normal Controls

We used the 2D-LC-MS/MS data obtained previously from pooled CSF of 11 healthy control subjects [10]. Briefly, there were 8 women and 3 men, aged 24–55 years with a median age of 28 years. Individual CSF samples from another set of 10 healthy volunteers, age 37–44 years (median  = 40) and 5 women and 5 men, were analyzed by LC-MS analysis without immunoaffinity depletion.

### Immunoaffinity depletion of 14 high abundance CSF proteins

We had previously shown that this technique could increase our protein identification yield by 70% [10]. Pooled CSF samples from *CFS* or *nPTLS* patients (total volume of 18 mL each), were fractionated using a 12.7×79.0 mm Seppro® IgY14 LC10 affinity LC column (Sigma, St Louis, MO) as previously described [18]. Pooling was done to compensate for lack of sufficient volume (and consequent protein content) available for immunoaffinity depletion of individual patient samples. Both the flow-through (lower abundance proteins) and bound fractions from both pooled CSF samples were collected and processed identically until LC-MS/MS analysis. These analyses resulted in an in-depth characterization of the CSF proteome and the combined results of abundant protein and less abundant protein fractions allowed the creation of an AMT tag database [19] for high-throughput analysis of a larger number of individual subject samples using LC-MS.

### Protein digestion

CSF proteins (from the immunoaffinity depletion processed pooled samples and the individual samples without immunoaffinity depletion processing) were digested with trypsin and cleaned up with SPE C18 columns as previously described [10]. Final peptide concentration was determined by BCA assay (Pierce, Rockford, IL). All tryptic digests were snap frozen in liquid nitrogen and stored at −80°C until further processing and analysis.

### Strong cation exchange (SCX) fractionation

A total of 300 µg of tryptic peptides from both the IgY14 bound and flow-through fractions from the pooled *CFS* and *nPTLS* CSF samples were fractionated by SCX chromatography as described [20]. Thirty SCX fractions were collected for each sample and 20% of each fraction was injected for reversed-phase LC-MS/MS analysis.

### Reversed-phase capillary LC-MS/MS for CSF pooled fraction analysis

SCX fractions of the IgY14 bound fraction samples were analyzed on an LTQ (ThermoFisher, San Jose, CA) linear ion trap, and SCX fractions of the IgY14 flow-through fraction samples were analyzed on an LTQ-Orbitrap Velos (ThermoFisher) instrument, operated in data-dependent mode with the same LC conditions as previously described [10].

### Reversed-phase capillary LC-MS for label-free quantification of unfractionated CSF samples

For label-free quantification analyzing unfractionated CSF samples (individual patient samples with insufficient volume (protein content) for immunoaffinity depletion and SCX fractionation), the LTQ-Orbitrap Velos mass spectrometer was operated in the data-dependent mode with full scan MS spectra (m/z 400–2000) acquired in the LTQ-Orbitrap Velos with resolution of 60,000 at m/z 400 (accumulation target: 1,000,000). MS/MS data acquired here were not used for the quantitative analysis.

### Data analysis

The LTQ raw data from the pooled samples was extracted using Extract_MSn (version 3.0; ThermoFisher) and analyzed with the SEQUEST algorithm (V27 revision 12; ThermoFisher) searching the MS/MS data against the human IPI database (Version 3.40). Mass tolerances of 3 Daltons for precursor ions and 1 Dalton for fragment ions without an enzyme defined, as well as static carboxyamidomethylation of cysteine and dynamic oxidation of methionine were used for the database search. The LTQ-Orbitrap Velos MS/MS data were first processed by in-house software DeconMSn [21] accurately determining the monoisotopic mass and charge state of parent ions, followed by SEQUEST search against the IPI database in the same fashion as described above, with the exception that a 0.1-Dalton mass tolerance for precursor ions and 1-Dalton mass tolerance for fragment ions were used. Data filtering criteria based on the cross correlation score (Xcorr) and delta correlation (ΔCn) values along with tryptic cleavage and charge states were developed using the decoy database approach and applied for filtering the raw data to limit false positive identifications to <1% at the peptide level [22]–[24]. For the LTQ-Orbitrap Velos data, the distribution of mass deviation (from the theoretical masses) was first determined as having a standard deviation (σ) of 2.05 part per million (ppm), and a mass error of smaller than 3σ was used in combination with Xcorr and ΔCn to determine the filtering criteria that resulted in <1% false positive peptide identifications.

The AMT tag strategy [19] was used for label-free quantification of MS features observed in the LTQ-Orbitrap Velos analysis of the individual CSF samples from normal, *CFS* and *nPTLS* conditions. The filtered MS/MS peptide identifications obtained from the 2D-LC-MS/MS analyses of all pooled CSF samples were included in an AMT tag database with their theoretical mass and normalized elution time (NET; from 0 to 1) recorded. LC-MS datasets were then analyzed by in-house software VIPER [25] that detects features in mass–NET space and assigned them to peptides in the AMT tag database [26]. The data was further filtered by requiring that all peptides must be detected in at least 30% of the datasets in each of the three conditions. The false discovery rate of the AMT tag analysis was estimated using an 11-Da shift strategy as previously described [27]. A false positive rate of <4% was estimated for each of the LC-MS data sets. The resulting lists of peptides from 2D-LC-MS/MS or direct LC-MS analysis were further processed by ProteinProphet software [28] to remove redundancy in protein identification.

Data normalization and quantification of the changes in protein abundance between the normal, *CFS* and *nPTLS* CSF samples were performed and visualized using in-house software DAnTE [29]. Briefly, peptide intensities from the LC-MS analyses of the individual samples (volume limited) were log2 transformed and normalized using a mean central tendency procedure. Peptide abundances from the individual samples were then “rolled up” to the protein level employing the R-rollup method (based on trends at peptide level) implemented in DAnTE. ANOVA, principal component analysis (PCA) and clustering analyses were also performed using DAnTE.

Pathway Analysis of the data was performed with Ingenuity Pathways Analysis (Ingenuity Systems, www.ingenuity.com). Canonical pathway analysis identified the pathways from the Ingenuity Pathways Analysis library of canonical pathways that were most significant to the *CFS* and *nPTLS* proteins identified. The significance of the associations were assessed with the Fisher's exact test.

## Results

We first performed pooled sample analysis, then individual sample analysis, and then pathway analysis using the observed proteins. These analyses represent a discovery phase of our studies on *CFS* and *nPTLS*, generating targets which can be followed up in future verification and validation stages studies [13].

### Proteomic analysis of pooled CSF samples

**Figure 1:**
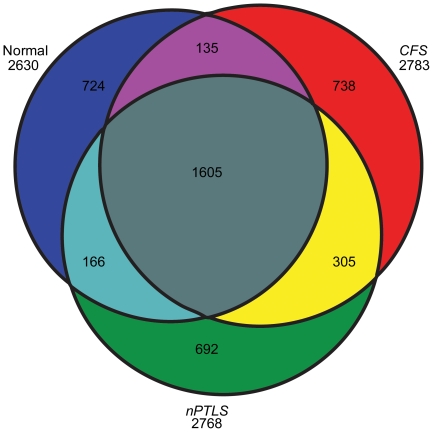
Characterization of the proteome from pooled and individual CSF samples. **A**) Venn diagram of the qualitative distribution of proteins identified in the pooled, immunodepleted, and fractionated cerebrospinal fluid (CSF) from normal healthy control subjects, Chronic Fatigue Syndrome (*CFS*), and Neurologic Post Treatment Lyme Syndrome (*nPTLS*). The numbers of proteins for each of these three categories separately is shown outside the circles below the category (2,630 for true normal controls, 2,783 for *CFS*, and 2,768 for *nPTLS*). The subsets of intersections between these categories are shown within the circles. There were 1) 738 proteins that were identified in *CFS,* but not in either healthy normal controls or *nPTLS*; 2) 1,582 proteins that were not identified in *CFS,* but were in either *nPTLS* disease or healthy normal controls; 3) 692 proteins that were identified in the *nPTLS* patients, but not in healthy normal controls or *CFS*; and 4) 1,597 proteins that were not identified in *nPTLS,* but were identified in either healthy normal controls or *CFS*. This figure also shows that the *nPTLS* and *CFS* groups shared significantly more proteins (n = 305) than each disease group shared with controls (n's = 135 and 166). The specific lists of these subsets are presented in additional Table S1.

In the pooled analysis, we examined individual sets of CSF samples from *CFS* patients (n = 43) and *nPTLS* patients (n = 25), respectively. We used the proteomic strategy described in Methods to assure that the maximum number of proteins would be analyzed and the more abundant proteins did not obscure the less abundant ones having biomarker potential. The bound fraction of abundant proteins from the immunoaffinity depleted flow through fraction was analyzed separately and included in the subsequent analysis. Combining immunoaffinity-based partitioning, SCX fractionation and LC-MS/MS, we identified approximately 30,000 peptides for each pooled sample corresponding to 2,783 nonredundant proteins in *CFS* patient samples and 2,768 proteins in *nPTLS* patient samples, compared to the 2,630 proteins present in the CSF of healthy normal control subjects. These can be graphically seen in Figure 1 which shows the number of proteins identified solely in each group, and shared or not shared between the groups (see Table S1). Figure 1 also shows that the *nPTLS* and *CFS* groups shared significantly more proteins (n = 305) than each disease group shared with healthy controls (n's = 135 and 166, respectively). (Note that, as with any assay, when we indicate that a protein was “not found” or “not identified” that is defined as within the limits of detection).

### Proteomic analysis of individual CSF samples

Quantitative analyses were performed on individual CSF samples from 14 *CFS* patients and 14 *nPTLS* patients. They were compared to 10 normal healthy volunteers (samples chosen at random) to provide insights on the variation among individuals within and between different groups. Limited volumes of the individual samples reduced the sample preparation options (i.e., immunoaffinity depletion and SCX fractionation), and hence resulted in less depth of proteome coverage than possible with the pooled samples, where approximately 20 ml were available for depletion and fractionation. Nevertheless, we identified 4,522 peptides across all individual samples, representative of 474 non-redundant proteins identified and quantified in the individual sample analysis (Table S2).

**Figure 2:**
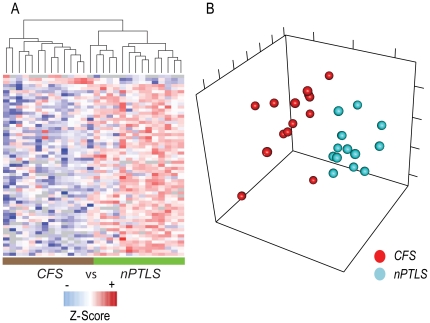
Comparative analysis of individual *CFS* and *nPTLS* CSF proteomes. **A**) Unsupervised hierarchical clustering of 59 proteins (see Table S3) that are differentially abundant as determined by ANOVA (p<0.01) clearly separates these two disease states with the exception of one *nPTLS* sample clustering with *CFS* patient samples. **B**) Principal Component Analysis (PCA) of *CFS* and *nPTLS* samples demonstrates that the CSF proteomes, and by extension of the CNS status, differ between *CFS* and *nPTLS*.

Unsupervised hierarchical clustering and PCA were employed to determine if the observed quantitative differences in protein abundances were sufficient to distinguish these two patient groups (this was *de facto* blinded – as samples were run in a random order and uncoded as to disease group afterwards). The proteins considered in the unsupervised hierarchical clustering analysis were quantified in individual samples and found to be significantly different in abundance by analysis of variance (ANOVA p ≤ 0.01, Table S3); while PCA analysis considered all proteins quantified in each individual sample. The CSF proteome of the two disease states were markedly different from each other (Fig. 2A and B). Individual patients also showed consistent patterns of protein abundances discriminating *CFS* from *nPTLS* (Fig. 2A). These results demonstrated that it is unlikely that any single subject's CSF sample in the pooled analysis contributed disproportionately to the differential proteome distributions observed between the disease groups. Moreover, the individual analyses also highlighted the potential for diagnostic marker confirmation upon extension to larger sample sets in validation studies.

### Illustrative pathway analyses of protein results from CSF samples

We utilized pathway analysis as an exploratory tool to assess the value of our data, beyond distinguishing the two syndromes from each other, to see if the data was amenable to analysis that would help generate hypotheses of pathogenesis. We chose representative pathways to analyze for illustration based in part on their quantitative ranking (Table S4) and in part by the potential relevance of the pathway involved. Even this limited investigation demonstrated that there is a wealth of proteome information that can be leveraged for hypotheses generation.

#### Example of proteins in common and elevated in abundance in the two disease conditions, compared to normal, but at different levels

An illustration, where the same proteins are elevated in abundance in both conditions, but at different magnitudes, is provided by inspection of proteins in the complement system. This is of interest because both syndromes may be triggered by infections (*nPTLS* in all cases by *B. burgdorferi*; many *CFS* cases by one or more microbes yet to be identified). We found that the complement cascade related proteins were identified and significantly enriched in both *CFS* and *nPTLS* pooled CSF proteomes by the Fisher Exact test (p = 0.005) implemented in Ingenuity Pathways Analysis (Figure S1A). In individual patient samples analyzed, we identified and quantified 4 components (C1S, C4B, C1QB, C1QC) which are seen with activation of the complement cascade and which were differentially increased in abundance consistently across the *nPTLS* patients compared to *CFS* (Figure S1B and C). This represents the type of data that can be useful in the formulation of pathogenetic hypotheses because the role of complement in these disorders is under-explored.

#### Example of proteins solely identified in one condition

Analysis of the highly fractionated pooled patient samples led to the identification of proteins solely identified in each of the disease states. To investigate if these disease specific proteins have common annotated functional properties, we performed pathway analysis (Tables S5 and S6). As an example, the CDK5 signaling pathway, was found to be significantly enriched (p = 0.00009) for proteins identified only in the pooled *CFS* proteome. This signaling pathway has been linked to Parkinson's [30] and Alzheimer's diseases [31].

#### Example of proteins in common and decreased in abundance in the two disease conditions, compared to normal, but at different levels

In certain cases, proteins were found to be decreased in both *CFS* and *nPTLS* compared to healthy normal controls. However, quantitative distinguishing differences could still be found between the two conditions. A specific example relates to networks relevant to neurological function such as axonal guidance (Figures S2A and B), where the proteins in *CFS* were further decreased relative to *nPTLS*. These findings highlight quantifiable differences between *CFS* and *nPTLS* that may be found, with respect to certain proteins such as those that are known to effect the dynamic changes in CNS cellular architecture, such as axon, neurite, and dendritic spine growth and organization.

## Discussion

Our results support the concept that *CFS* and *nPTLS* are distinguishable disorders with distinct CSF proteomes, where one can be separated from the other. The results also demonstrate that each condition has a multitude of candidate diagnostic biomarkers for future validation and optimization studies. The discovery of many of the same proteins in each proteome is important because it allows comparative pathway analysis, so that useful hypotheses of pathogenesis can be formulated and tested.

Our results represent the most comprehensive analysis of the whole CSF proteome to date for both *CFS* and *nPTLS*. These two disorders have similar symptoms that have created diagnostic dilemmas. It has been speculated that one (*nPTLS*) is a subset of the other, but our results do not support that notion. Our findings alone do not describe why *CFS* or *nPTLS* occur, but are provided to illustrate that CSF proteome analysis may provide important and meaningful insights into the biological processes modulated as a function of disease and facilitate the identification of protein candidates for further investigation. Analytical strategies need to be developed for application to those proteins and their pathways that may not have been described yet. Nevertheless, *in toto*, these results are encouraging because there is an abundance of data now that can be analyzed with existing tools and future methods to develop hypotheses on pathogenesis [9], [32].

We regard the proteins that were identified only in one group or differentially abundant between groups, as possible or candidate biomarkers that can be subjected to further analysis in validation and verification studies. The clinical significance of the proteins identified in each pooled sample is difficult to determine in the current discovery phase. As with most technologic methods, we expect multiple replicate analyses of the highly fractionated samples would result in a reduction of the number of seemingly unique proteins identified for each disease group [33].

An important strategy that can be used post-discovery towards validation, is the use of targeted approaches that are either MS-based, immuno-based, or a combination of these approaches [12], [34]. One approach, selected reaction monitoring (SRM) MS, allows for much higher sensitivity and specificity, more accurate quantification, and much higher throughput to be achieved for simultaneously measuring many biomarker candidates in large clinical cohorts [35]–[37]. This approach also compensates for any theoretical over-representation of proteins in pooled samples by a single or small number of individuals. This is a strategy that we plan to use not only for these diseases, but in the investigation of other diseases with neuropsychiatric features. SRM-MS analysis will permit us to directly use small-sized samples, such as the individual CSF samples, enable verification of marker candidates that currently do not have available antibodies (hence not amenable to conventional analyses such as ELISA or Western blots), and provide robust statistical analyses on individual candidate markers or combinations of them to determine which would make the best biomarker(s) for a particular disease condition. Immunobased assays such as ELISA or Western blots may also be used for targeted approaches, but will likely have more utility during a clinical validation phase where much larger sample cohorts are used. Some may choose to apply these methods for additional orthogonal confirmation of a result. However, its greater value may lie in its widespread use as a common diagnostic platform. Regardless of the method chosen, identification of diagnostic CSF biomarkers may be the necessary prelude to a search for the same markers in the highly complex blood, because it permits targeted searches for markers that might otherwise be obscured or have uncertain relevance.

With respect to biomarkers, we believe our proteomic strategy [10], that did not require prior knowledge of which proteins might be present in the CSF, will accelerate the transition from a discovery phase of candidate biomarkers, as described in this study, to full validation for clinical application. We and others have cited important elements that should be considered when an assay or biomarker is being developed for preliminary or full validation [38]–[40].

Distinguishing *CFS* and *nPTLS* will have etiologic implications which could lead to novel diagnostics and therapeutic interventions. On a broader level the strategy we employed may prove useful in providing investigative foundations in other poorly understood neurological conditions.

## Supporting Information

Figure S1 **Illustrative example of pathway analysis with respect to complement pathways.** Protein network and pathway analysis was performed employing Ingenuity Pathways Analysis tools (v8.6- www.ingenuity.com). A) Proteins that participate in complement signaling were significantly enriched (p  =  6×10^−20^) in the CSF proteomes for pooled disease-specific samples. A comparison of protein abundance determined by spectral counts reveals difference between disease states and normal healthy control CSF. Proteins with an increased abundance are colored red and those that decrease in abundance relative to normal healthy control are colored green. B) Proteins annotated as participating in complement that were detected in individual patient analysis are shown the heatmap. Protein abundances measured by ion intensity transformed to Z scores clearly show differences between *CFS* and *nPTLS* patients. C) Receiver operator characteristic (ROC) curves demonstrate the discriminating power of the select set of proteins that were detected as having statistical differences by ANOVA (p<0.05) in abundance in the analysis of individual patient samples.
(TIF)

Figure S2 **Illustrative example of pathway analysis with respect to axonal guidance pathways.** Protein network and pathway analysis were performed employing Ingenuity Pathways Analysis tools (v8.6- www.ingenuity.com). A) Proteins that associated with axonal guidance and signaling were significantly enriched (p = 6×10^−20^) in the CSF proteomes for all pooled samples. A comparison of protein abundances determined by spectral counts revealed differences between disease states and normal healthy control CSF. Proteins with an increased abundance are colored red and proteins with decreased abundance relative to normal/controls are colored green. B) Normalized protein abundance clearly differs between *CFS* and *nPTLS* patients. C) Receiver operator characteristic (ROC) curves demonstrate the discriminating power of the select set of proteins that were detected in individual CSF samples as well as in the pooled proteome.
(TIF)

Table S1 Proteins identified in normal, *CFS*, and *nPTLS* pooled samples.
(PDF)

Table S2 Proteins (n = 474) identified in the analysis of non-fractionated and immunodepleted individual patient samples.
(PDF)

Table S3 Proteins (n = 59) that were quantified and determined to be significantly different in abundance by ANOVA (p ≤ 0.01) when comparing *CFS* from *nPTLS* subject samples and allow for separation of these two syndromes when performing unsupervised hierarchical cluster analysis.
(PDF)

Table S4 Pathway enrichment determination using Ingenuity pathways analysis tools for proteins present in *nPTLS* and *CFS* proteomes. Analysis of proteins detected in the highly fractionated, immunodepleted, pooled CSF samples led to the identification of pathways that are significantly enriched (p ≤ 0.05) by the proteins from the CSF proteomes.
(PDF)

Table S5 Pathways significantly enriched by proteins identified only in the pooled sample proteome for *nPTLS* patients.
(PDF)

Table S6 Pathways significantly enriched by proteins identified only in the pooled sample proteome for *CFS* patients.
(PDF)
