# Supplementary figures and images for: Distinct Cerebrospinal Fluid Proteomes Differentiate Post-Treatment Lyme Disease from Chronic Fatigue Syndrome
Source: PLoS One. 2011 Feb 23;6(2):e17287. doi: 10.1371/journal.pone.0017287 (PMC3044169; doi:10.1371/journal.pone.0017287)

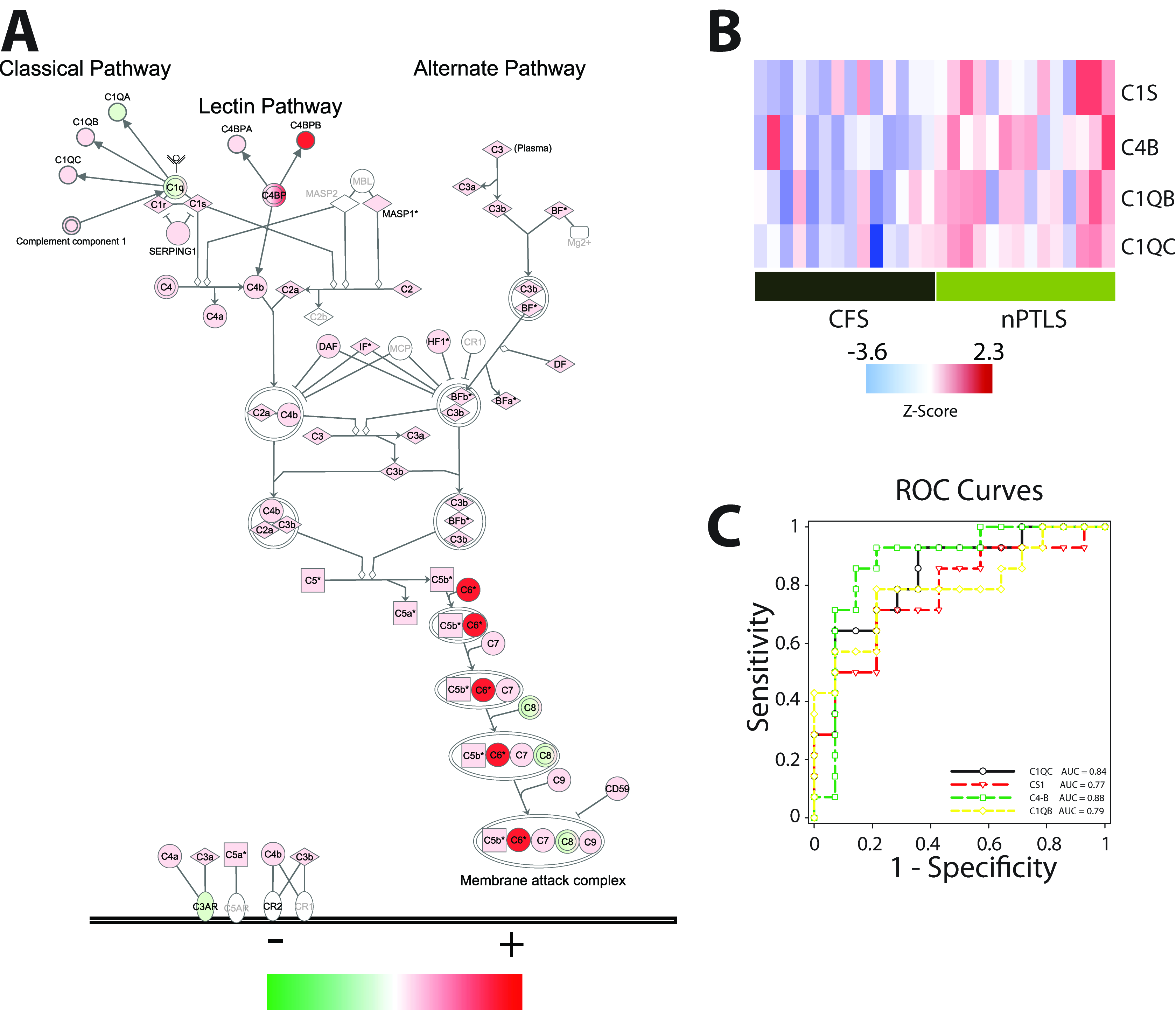

Supplement: Figure S1 — Illustrative example of pathway analysis with respect to complement pathways. Protein network and pathway analysis was performed employing Ingenuity Pathways Analysis tools (v8.6- www.ingenuity.com). A) Proteins that participate in complement signaling were significantly enriched (p = 6×10^−20^) in the CSF proteomes for pooled disease-specific samples. A comparison of protein abundance determined by spectral counts reveals difference between disease states and normal healthy control CSF. Proteins with an increased abundance are colored red and those that decrease in abundance relative to normal healthy control are colored green. B) Proteins annotated as participating in complement that were detected in individual patient analysis are shown the heatmap. Protein abundances measured by ion intensity transformed to Z scores clearly show differences between CFS and nPTLS patients. C) Receiver operator characteristic (ROC) curves demonstrate the discriminating power of the select set of proteins that were detected as having statistical differences by ANOVA (p<0.05) in abundance in the analysis of individual patient samples. (TIF) [file pone.0017287.s001.tif]

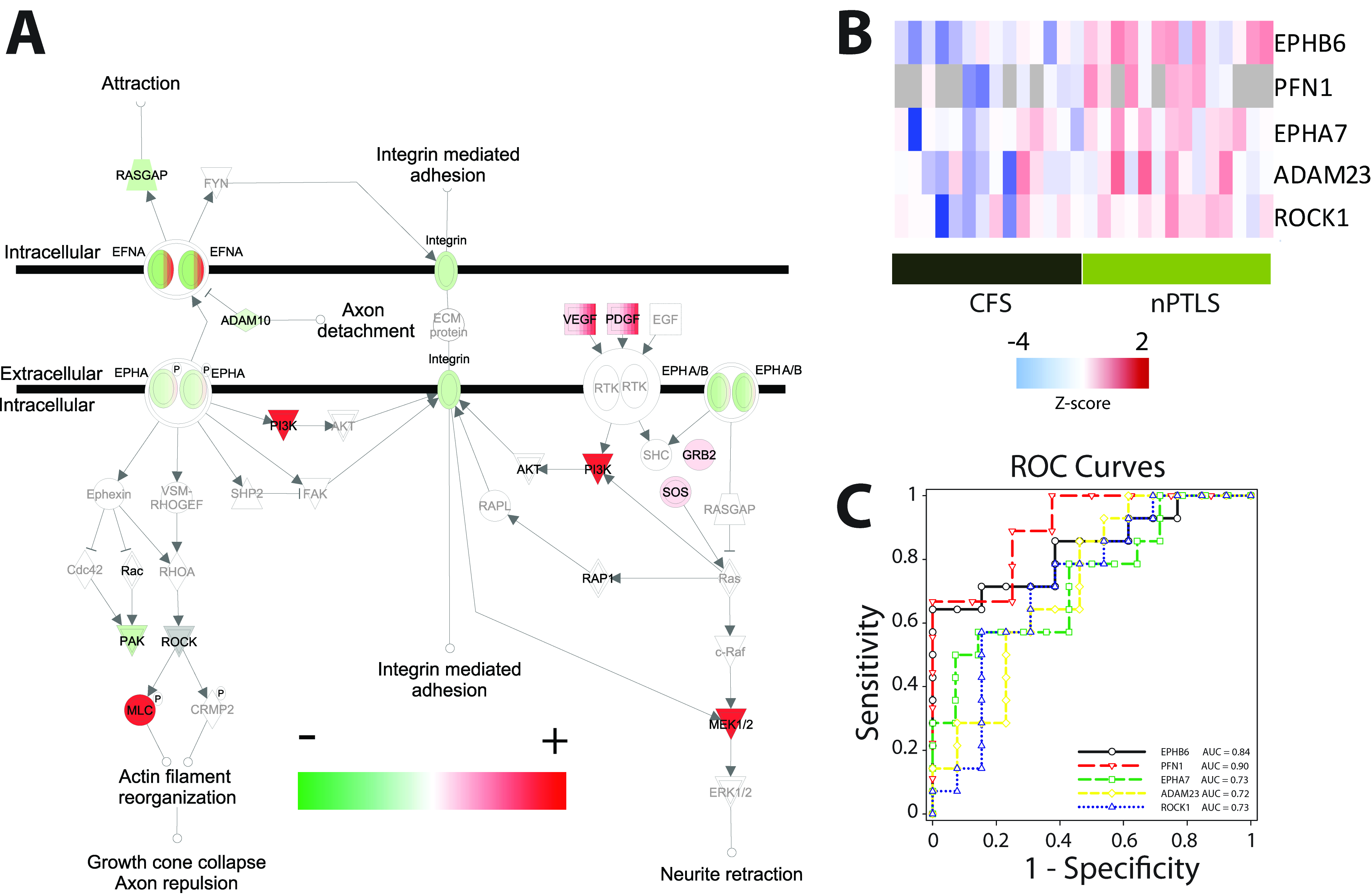

Supplement: Figure S2 — Illustrative example of pathway analysis with respect to axonal guidance pathways. Protein network and pathway analysis were performed employing Ingenuity Pathways Analysis tools (v8.6- www.ingenuity.com). A) Proteins that associated with axonal guidance and signaling were significantly enriched (p = 6×10^−20^) in the CSF proteomes for all pooled samples. A comparison of protein abundances determined by spectral counts revealed differences between disease states and normal healthy control CSF. Proteins with an increased abundance are colored red and proteins with decreased abundance relative to normal/controls are colored green. B) Normalized protein abundance clearly differs between CFS and nPTLS patients. C) Receiver operator characteristic (ROC) curves demonstrate the discriminating power of the select set of proteins that were detected in individual CSF samples as well as in the pooled proteome. (TIF) [file pone.0017287.s002.tif]
