# Supplementary material for: Distinct Cerebrospinal Fluid Proteomes Differentiate Post-Treatment Lyme Disease from Chronic Fatigue Syndrome
Source: PLoS One. 2011 Feb 23;6(2):e17287. doi: 10.1371/journal.pone.0017287 (PMC3044169; doi:10.1371/journal.pone.0017287)
Supplement: Table S1 — Proteins identified in normal, CFS, and nPTLS pooled samples. (PDF) [file pone.0017287.s003.pdf]

Table S1. Proteins identified in normal, CFS, and nPTLS pooled samples

Number of unique peptides identified

IPI	Protein name	Gene symbol	Swiss Prot ID	Normal	CFS	nPTLS
IPI00022434	Uncharacterized protein ALB	ALB		189	247	230
IPI00783987	Complement C3 precursor (Fragment)	C3	P01024	153	180	176
IPI00418163	C4B1	C4B		153	410	426
IPI00032258	Complement C4-A precursor	C4A	P0C0L4	151	394	414
IPI00878517	56 kDa protein	ALB		141	185	165
IPI00006601	Secretogranin-1 precursor	CHGB	P05060	133	249	246
IPI00384697	Isoform 2 of Serum albumin precursor	ALB	P02768	119	163	145
IPI00022463	Serotransferrin precursor	TF	P02787	105	141	105
IPI00069058	VGF nerve growth factor inducible precursor	VGF		105	152	154
IPI00798430	Transferrin variant (Fragment)	TF		104	130	99
IPI00478003	Alpha-2-macroglobulin precursor	A2M	P01023	97	120	110
IPI00017601	Ceruloplasmin precursor	CP	P00450	95	269	243
IPI00292071	Secretogranin-3 precursor	SCG3	Q8WXD2	90	147	133
IPI00026314	Isoform 1 of Gelsolin precursor	GSN	P06396	86	254	261
IPI00289501	Neurosecretory protein VGF precursor	VGF	O15240	83	133	131
IPI00029751	Isoform 1 of Contactin-1 precursor	CNTN1	Q12860	83	177	179
IPI00013179	Prostaglandin-H2 D-isomerase precursor	PTGDS	P41222	81	114	128
IPI00514285	Prostaglandin D2 synthase 21kDa	PTGDS		79	113	124
IPI00296777	SPARC-like protein 1 precursor	SPARCL1	Q14515	79	176	175
IPI00022229	Apolipoprotein B-100 precursor	APOB	P04114	79	55	62
IPI00006114	Pigment epithelium-derived factor precursor	SERPINF1	P36955	76	143	152
IPI00783390	Isoform 1 of Neural cell adhesion molecule L1-like protein precursor	CHL1	O00533	73	165	168
IPI00029739	Isoform 1 of Complement factor H precursor	CFH	P08603	73	110	106
IPI00242956	IgGFc-binding protein precursor	FCGBP	Q9Y6R7	73	153	174
IPI00299059	Isoform 2 of Neural cell adhesion molecule L1-like protein precursor	CHL1	O00533	72	164	167
IPI00291262	Clusterin precursor	CLU	P10909	71	120	118
IPI00794184	97 kDa protein	CP		70	200	179
IPI00788189	similar to Fc fragment of IgG binding protein	FCGBP		68	140	157
IPI00553177	Isoform 1 of Alpha-1-antitrypsin precursor	SERPINA1	P01009	68	61	56
IPI00304273	Apolipoprotein A-IV precursor	APOA4	P06727	65	117	122

Table S1.

IPI	Protein name	Gene symbol	Swiss Prot ID	Number of unique peptides identified<br>Normal	CFS	nPTLS
IPI00883753	NRCAM protein	NRCAM		64	161	158
IPI00847179	apolipoprotein A-IV precursor	APOA4		64	115	124
IPI00024284	Basement membrane-specific heparan sulfate proteoglycan core protein precursor	HSPG2	P98160	64	91	98
IPI00555812	Vitamin D-binding protein precursor	GC	P02774	63	221	183
IPI00333776	Isoform 1 of Neuronal cell adhesion molecule precursor	NRCAM	Q92823	63	165	160
IPI00742696	vitamin D-binding protein precursor	GC		62	220	182
IPI00415032	Isoform 4 of Neuronal cell adhesion molecule precursor	NRCAM	Q92823	62	161	158
IPI00072917	alpha 3 type VI collagen isoform 3 precursor	COL6A3		61	38	46
IPI00022488	Hemopexin precursor	HPX	P02790	61	197	189
IPI00647027	32 kDa protein	CHGB		60	98	91
IPI00607600	amyloid precursor-like protein 1 isoform 1 precursor	APLP1		59	116	122
IPI00020557	Prolow-density lipoprotein receptor-related protein 1 precursor	LRP1	Q07954	59	63	68
IPI00241562	reelin isoform a	RELN	P78509	59	75	74
IPI00020012	Amyloid-like protein 1 precursor	APLP1	P51693	59	119	128
IPI00032220	Angiotensinogen precursor	AGT	P01019	58	159	149
IPI00007257	calsyntenin 1 isoform 2	CLSTN1		57	118	121
IPI00878576	Autotaxin isoform gamma	-		57	186	175
IPI00032291	Complement C5 precursor	C5	P01031	57	98	101
IPI00414249	Isoform 1 of Neurexin-3-alpha precursor	NRXN3	Q9Y4C0	57	89	94
IPI00156171	Isoform 1 of Ectonucleotide pyrophosphatase/phosphodiesterase family member 2 precursor	ENPP2	Q13822	57	189	175
IPI00413959	Calsyntenin-1 precursor	CLSTN1	O94985	56	118	121
IPI00021842	Apolipoprotein E precursor	APOE	P02649	56	105	101
IPI00064667	Beta-Ala-His dipeptidase precursor	CNDP1	Q96KN2	55	110	128
IPI00303210	Isoform 2 of Ectonucleotide pyrophosphatase/phosphodiesterase family member 2 precursor	ENPP2	Q13822	54	182	170
IPI00216728	Neurexin 3-alpha	NRXN3		53	82	90
IPI00006608	Isoform APP770 of Amyloid beta A4 protein precursor (Fragment)	APP	P05067	53	159	139
IPI00007921	Isoform 1 of Neurexin-2-alpha precursor	NRXN2	Q9P2S2	51	63	71
IPI00655702	Isoform 5 of Neurofascin precursor	NFASC	O94856	50	70	77
IPI00550991	Alpha-1-antichymotrypsin precursor	SERPINA3	P01011	50	139	118
IPI00009362	Secretogranin-2 precursor	SCG2	P13521	50	85	93
IPI00470535	Dihydropyridine receptor alpha 2 subunit	CACNA2D1		49	81	82

Table S1.

IPI	Protein name	Gene symbol	Swiss Prot ID	Number of unique peptides identified Normal	CFS	nPTLS
IPI00795918	neural cell adhesion molecule 1 isoform 2	NCAM1		49	105	106
IPI00019591	Isoform 1 of Complement factor B precursor (Fragment)	CFB	P00751	49	155	145
IPI00290315	Chromogranin-A precursor	CHGA	P10645	49	99	103
IPI00291136	Collagen alpha-1(VI) chain precursor	COL6A1	P12109	48	64	54
IPI00021841	Apolipoprotein A-I precursor	APOA1	P02647	48	51	52
IPI00177543	peptidylglycine alpha-amidating monooxygenase isoform a, preproprotein	PAM	P19021	48	91	94
IPI00394655	Isoform 4 of Neurofascin precursor	NFASC	O94856	48	72	78
IPI00479514	Voltage-dependent calcium channel subunit alpha-2/delta-1 precursor	CACNA2D1	P54289	48	77	76
IPI00639937	B-factor, properdin	CFB		47	154	144
IPI00021000	Isoform A of Osteopontin precursor	SPP1	P10451	46	65	61
IPI00478809	Coagulation factor V precursor	F5	P12259	46	62	62
IPI00022937	Coagulation factor V	F5		46	61	62
IPI00032179	Antithrombin III variant	SERPINC1	P01008	46	133	127
IPI00023814	Isoform 1 of Neogenin precursor	NEO1	Q92859	45	67	65
IPI00220741	spectrin, alpha, erythrocytic 1	SPTA1	P02549	45		1
IPI00019580	Plasminogen precursor	PLG	P00747	44	102	92
IPI00294193	Isoform 1 of Inter-alpha-trypsin inhibitor heavy chain H4 precursor	ITIH4	Q14624	44	104	85
IPI00024966	Contactin-2 precursor	CNTN2	Q02246	44	90	89
IPI00218192	Isoform 2 of Inter-alpha-trypsin inhibitor heavy chain H4 precursor	ITIH4	Q14624	44	99	81
IPI00218875	Isoform C of Osteopontin precursor	SPP1	P10451	43	56	54
IPI00219042	Isoform 3 of Peptidyl-glycine alpha-amidating monooxygenase precursor	PAM	P19021	42	87	90
IPI00306339	secreted phosphoprotein 1 isoform b	SPP1		42	61	55
IPI00739237	similar to Complement C3 precursor	LOC653879		42	47	46
IPI00296608	Complement component C7 precursor	C7	P10643	42	107	126
IPI00009997	N-acetyllactosaminide beta-1,3-N-acetylglucosaminyltransferase	B3GNT1	O43505	41	112	114
IPI00305461	Inter-alpha-trypsin inhibitor heavy chain H2 precursor	ITIH2	P19823	41	105	91
IPI00334238	neuronal pentraxin receptor	NPTXR	O95502	41	79	76
IPI00032293	Cystatin-C precursor	CST3	P01034	41	107	94
IPI00220644	Isoform M1 of Pyruvate kinase isozymes M1/M2	PKM2	P14618	41	42	62
IPI00853525	Apolipoprotein A1	APOA1		40	42	44
IPI00218874	Isoform B of Osteopontin precursor	SPP1	P10451	40	58	52
IPI00215894	Isoform LMW of Kininogen-1 precursor	KNG1	P01042	39	111	94

Table S1.

IPI	Protein name	Gene symbol	Swiss Prot ID	Number of unique peptides identified<br>Normal	CFS	nPTLS
IPI00031030	Isoform 1 of Amyloid-like protein 2 precursor	APLP2	Q06481	39	69	67
IPI00025276	Isoform XB of Tenascin-X precursor	TNXB	P22105	39	20	33
IPI00032328	Isoform HMW of Kininogen-1 precursor	KNG1	P01042	37	106	88
IPI00376427	Neural cell adhesion molecule 2 precursor	NCAM2	O15394	37	57	53
IPI00456623	Isoform 1 of Brevican core protein precursor	BCAN	Q96GW7	37	62	61
IPI00019943	Afamin precursor	AFM	P43652	36	80	66
IPI00748312	protein tyrosine phosphatase, receptor-type, zeta1 precursor	PTPRZ1	P23471	36	42	45
IPI00291866	Plasma protease C1 inhibitor precursor	SERPING1	P05155	36	98	99
IPI00021885	Isoform 1 of Fibrinogen alpha chain precursor	FGA	P02671	35	40	35
IPI00022395	Complement component C9 precursor	C9	P02748	35	54	51
IPI00289831	Isoform PTPS of Receptor-type tyrosine-protein phosphatase S precursor	PTPRS	Q13332	35	51	49
IPI00027310	Isoform 1 of Multiple epidermal growth factor-like domains 8	MEGF8	Q7Z7M0	34	84	77
IPI00815926	IGHG1 protein	IGHG1		34	42	49
IPI00607580	multiple EGF-like-domains 8	MEGF8	Q7Z7M0	34	89	83
IPI00302641	Protocadherin Fat 2 precursor	FAT2	Q9NYQ8	34	36	38
IPI00784828	Putative uncharacterized protein DKFZp686C11235	-		34	39	42
IPI00784807	Putative uncharacterized protein	-		34	29	35
IPI00015260	Protein kinase C-binding protein NELL2 precursor	NELL2	Q99435	34	77	78
IPI00017696	Complement C1s subcomponent precursor	C1S	P09871	34	70	69
IPI00375547	Protein tyrosine phosphatase receptor type D	PTPRD	P23468	34	34	37
IPI00002714	Dickkopf-related protein 3 precursor	DKK3	Q9UBP4	33	128	126
IPI00426051	Putative uncharacterized protein DKFZp686C15213	-		33	31	34
IPI00219798	Isoform 1 of Roundabout homolog 1 precursor	ROBO1	Q9Y6N7	33	37	32
IPI00472345	IGHG3 protein	IGHG3	P01860	32	36	37
IPI00296537	Isoform C of Fibulin-1 precursor	FBLN1	P23142	32	52	49
IPI00384542	Isoform 2 of Nidogen-1 precursor	NID1	P14543	32	31	32
IPI00791343	261 kDa protein	-		32	76	70
IPI00025465	Mimecan precursor	OGN	P20774	32	56	63
IPI00796279	25 kDa protein	SERPINF1		32	64	62
IPI00107831	Receptor-type tyrosine-protein phosphatase F precursor	PTPRF	P10586	32	27	26

Table S1.

IPI	Protein name	Gene symbol	Swiss Prot ID	Number of unique peptides identified<br>Normal	CFS	nPTLS
IPI00761159	IGHM protein	IGHM		32	36	40
IPI00026944	Isoform 1 of Nidogen-1 precursor	NID1	P14543	32	31	32
IPI00784894	Putative uncharacterized protein	-		32	34	37
IPI00018219	Transforming growth factor-beta-induced protein ig-h3 precursor	TGFBI	Q15582	31	66	55
IPI00298497	Fibrinogen beta chain precursor	FGB	P02675	31	39	43
IPI00031121	Carboxypeptidase E precursor	CPE	P16870	31	58	73
IPI00003802	Alpha-mannosidase 2	MAN2A1	Q16706	31	31	30
IPI00025257	Semaphorin-7A precursor	SEMA7A	O75326	31	67	66
IPI00448925	IGHG1 protein	IGHG1		31	36	40
IPI00328829	inter-alpha trypsin inhibitor heavy chain precursor 5 isoform 1	ITIH5		31	23	28
IPI00022432	Transthyretin precursor	TTR	P02766	30	110	107
IPI00641737	Haptoglobin precursor	HP	P00738	30	39	34
IPI00816741	Complement component 5 variant (Fragment)	C5		30	53	55
IPI00784842	Putative uncharacterized protein DKFZp686G11190	IGHV4-31		30	33	41
IPI00296534	Isoform D of Fibulin-1 precursor	FBLN1	P23142	30	44	45
IPI00218725	laminin alpha 2 subunit isoform b precursor	LAMA2		30	27	43
IPI00027703	Isoform Long of Alpha-mannosidase IIx	MAN2A2	P49641	30	50	50
IPI00423463	Putative uncharacterized protein DKFZp686O01196	IGHG1		30	32	38
IPI00784810	IGHV4-31 protein	IGHV4-31		29	32	39
IPI00219029	Aspartate aminotransferase, cytoplasmic	GOT1	P17174	29	39	41
IPI00159927	Neurocan core protein precursor	NCAN	O14594	29	49	46
IPI00785084	Immunoglobulin heavy variable 4-31	IGHV4-31		29	32	36
IPI00295542	Nucleobindin-1 precursor	NUCB1	Q02818	29	43	51
IPI00744835	Isoform Sap-mu-9 of Proactivator polypeptide precursor	PSAP	P07602	29	75	75
IPI00012503	Isoform Sap-mu-0 of Proactivator polypeptide precursor	PSAP	P07602	29	77	76
IPI00418262	Fructose-bisphosphate aldolase C	ALDOC	P09972	29	38	35
IPI00011229	Cathepsin D precursor	CTSD	P07339	28	48	52
IPI00465439	Fructose-bisphosphate aldolase A	ALDOA	P04075	28	35	38
IPI00016334	Isoform 1 of Cell surface glycoprotein MUC18 precursor	MCAM	P43121	28	44	39
IPI00003362	HSPA5 protein	HSPA5	P11021	28	22	27
IPI00790899	55 kDa protein	-		28	43	50
IPI00853369	Plexin-B2 precursor	PLXNB2	O15031	28	45	44
IPI00872363	PTPRD protein	PTPRD		28	32	37

Table S1.

Number of unique peptides identified

IPI	Protein name	Gene symbol	Swiss Prot ID	Normal	CFS	nPTLS
IPI00423466	Putative uncharacterized protein DKFZp686H20196	IGHG1		28	31	37
IPI00334282	Protein FAM3C precursor	FAM3C	Q92520	27	52	63
IPI00019568	Prothrombin precursor (Fragment)	F2	P00734	27	126	107
IPI00296165	Complement C1r subcomponent precursor	C1R	P00736	27	48	43
IPI00829767	Uncharacterized protein IGHG2 (Fragment)	IGHG2		27	24	26
IPI00292950	Serpin peptidase inhibitor, clade D (Heparin cofactor), member 1	SERPIND1		27	47	47
IPI00292530	Inter-alpha-trypsin inhibitor heavy chain H1 precursor	ITIH1	P19827	27	61	63
IPI00023673	Galectin-3-binding protein precursor	LGALS3BP	Q08380	27	47	45
IPI00303963	Complement C2 precursor (Fragment)	C2	P06681	27	50	40
IPI00012545	Isoform TGN51 of Trans-Golgi network integral membrane protein 2 precursor	TGOLN2	O43493	26	15	18
IPI00016150	Neuroserpin precursor	SERPINI1	Q99574	26	46	42
IPI00000828	Proenkephalin A precursor	PENK	P01210	26	63	67
IPI00025252	Protein disulfide-isomerase A3 precursor	PDIA3	P30101	26	12	22
IPI00828205	IGHM protein	IGHM	P01871	26	26	24
IPI00004433	Contactin-6 precursor	CNTN6	Q9UQ52	26	19	25
IPI00451625	Isoform 2 of Cartilage acidic protein 1 precursor	CRTAC1	Q9NQ79	26	64	54
IPI00220117	Uncharacterized protein CD99	CD99	P14209	26	37	42
IPI00784942	Putative uncharacterized protein DKFZp686E23209	-		26	23	27
IPI00029658	Isoform 1 of EGF-containing fibulin-like extracellular matrix protein 1 precursor	EFEMP1	Q12805	26	37	43
IPI00300241	Leucine-rich repeat-containing protein 4B precursor	LRRC4B	Q9NT99	25	35	33
IPI00386879	CDNA FLJ14473 fis, clone MAMMA1001080, highly similar to Homo sapiens SNC73 protein (SNC73) mRNA	IGHV3OR16-13		25	36	24
IPI00022895	Alpha-1B-glycoprotein precursor	A1BG	P04217	25	89	81
IPI00394992	Isoform 2 of N-acetylmuramoyl-L-alanine amidase precursor	PGLYRP2	Q96PD5	25	82	74
IPI00550558	Protein O-linked-mannose beta-1,2-N-acetylglucosaminyltransferase 1	POMGNT1	Q8WZA1	25	34	33
IPI00298828	Beta-2-glycoprotein 1 precursor	APOH	P02749	25	87	73
IPI00163207	Isoform 1 of N-acetylmuramoyl-L-alanine amidase precursor	PGLYRP2	Q96PD5	25	82	74
IPI00009920	Complement component 6 precursor	C6	P13671	25	48	50

Table S1.

IPI	Protein name	Gene symbol	Swiss Prot ID	Number of unique peptides identified<br>Normal	CFS	nPTLS
IPI00382606	Factor VII active site mutant immunoconjugate	F7		25	28	31
IPI00439446	MAN1A1 protein	MAN1A1		24	29	36
IPI00015102	Isoform 1 of CD166 antigen precursor	ALCAM	Q13740	24	38	39
IPI00013976	Laminin subunit beta-1 precursor	LAMB1	P07942	24	26	40
IPI00788835	25 kDa protein	PENK		24	44	48
IPI00216171	Gamma-enolase	ENO2	P09104	24	27	29
IPI00029343	Isoform 1 of Contactin-associated protein-like 2 precursor	CNTNAP2	Q9UHC6	23	30	33
IPI00027780	72 kDa type IV collagenase precursor	MMP2	P08253	23	52	53
IPI00162735	Isoform 2 of Attractin precursor	ATRN	O75882	23	32	33
IPI00002147	Chitinase-3-like protein 1 precursor	CHI3L1	P36222	23	39	43
IPI00294395	Complement component C8 beta chain precursor	C8B	P07358	23	31	36
IPI00430842	IGHA1 protein	IGHV3OR16-13		23	28	22
IPI00008318	Ephrin type-A receptor 4 precursor	EPHA4	P54764	23	39	44
IPI00853454	200 kDa protein	LAMB1		23	26	41
IPI00744561	IGHA1 protein	IGHV3OR16-13		23	30	23
IPI00027087	Isoform 1 of Neural cell adhesion molecule L1 precursor	L1CAM	P32004	23	31	27
IPI00784998	Putative uncharacterized protein DKFZp686M24218	-		23	18	22
IPI00029863	SERPINF2 protein	SERPINF2		23	59	51
IPI00442294	Neurotrimin variant 3	HNT		22	32	29
IPI00296922	Laminin subunit beta-2 precursor	LAMB2	P55268	22	16	27
IPI00023845	Kallikrein-6 precursor	KLK6	Q92876	22	77	71
IPI00016915	Insulin-like growth factor-binding protein 7 precursor	IGFBP7	Q16270	22	55	51
IPI00374563	Agrin precursor	AGRN	O00468	22	29	33
IPI00002280	ProSAAS precursor	PCSK1N	Q9UHG2	22	55	56
IPI00844156	SERPINC1 protein	SERPINC1		22	63	48
IPI00166048	Isoform 1 of Cell adhesion molecule 3 precursor	CADM3	Q8N126	22	43	45
IPI00003351	Extracellular matrix protein 1 precursor	ECM1	Q16610	22	69	68
IPI00646281	L1 cell adhesion molecule	L1CAM		22	29	25
IPI00298971	Vitronectin precursor	VTN	P04004	21	36	44
IPI00220562	Neuronal pentraxin-1 precursor	NPTX1	Q15818	21	47	46
IPI00166622	similar to CG14446-PA	TMEM132C		21	16	17
IPI00216704	Isoform 2 of Spectrin beta chain, erythrocyte	SPTB	P11277	21		
IPI00029260	Monocyte differentiation antigen CD14 precursor	CD14	P08571	21	77	77
IPI00784950	Putative uncharacterized protein DKFZp686L19235	LOC100126583		21	24	19

Table S1.

IPI	Protein name	Gene symbol	Swiss Prot ID	Number of unique peptides identified<br>Normal	CFS	nPTLS
IPI00442297	Isoform 2 of Neurotrimin precursor	HNT	Q9P121	21	31	28
IPI00550731	Putative uncharacterized protein	-	P06310	21	34	28
IPI00830132	Uncharacterized protein IGHG4 (Fragment)	IGHG4		21	17	23
IPI00872555	cDNA FLJ76262, highly similar to Homo sapiens I factor (complement) (IF), mRNA	CFI		21	49	50
IPI00218733	Superoxide dismutase	SOD1	P00441	21	38	46
IPI00647704	CDNA FLJ41552 fis, clone COLON2004478, highly similar to Protein Tro alpha1 H,myeloma	IGHV3OR16-13		21	26	19
IPI00515041	Uncharacterized protein CFH	CFH		21	30	32
IPI00019157	Chondroitin sulfate proteoglycan 4 precursor	CSPG4	Q6UVK1	21	13	14
IPI00472249	protein tyrosine phosphatase, receptor type, N polypeptide 2 isoform 2 precursor	PTPRN2		21	23	18
IPI00465028	Isoform 1 of Triosephosphate isomerase	TPI1	P60174	21	31	37
IPI00787050	similar to neuronal pentraxin I precursor	NPTX1		21	43	43
IPI00654888	Plasma kallikrein precursor	KLKB1	P03952	20	34	31
IPI00792626	14 kDa protein	TF		20	20	14
IPI00171411	Golgi phosphoprotein 2	GOLM1		20	30	29
IPI00216697	Isoform Er1 of Ankyrin-1	ANK1	P16157	20		1
IPI00020996	Insulin-like growth factor-binding protein complex acid labile chain precursor	IGFALS	P35858	20	36	31
IPI00440577	IGKV2-24 protein	IGKV2-24		20	33	25
IPI00160552	Isoform 1 of Tenascin-R precursor	TNR	Q92752	20	28	21
IPI00783399	Isoform 1 of Transmembrane protein 132D precursor	TMEM132D	Q14C87	20	17	15
IPI00061977	IGHA1 protein	IGHV3OR16-13		20	28	15
IPI00294004	Vitamin K-dependent protein S precursor	PROS1	P07225	20	38	47
IPI00301865	Isoform 1 of Transmembrane protein 132A precursor	TMEM132A	Q24JP5	20	41	45
IPI00855725	Isoform 4 of Trans-Golgi network integral membrane protein 2 precursor	TGOLN2	O43493	20	12	12
IPI00784985	IGK@ protein	IGK@		19	33	24
IPI00020986	Lumican precursor	LUM	P51884	19	29	35
IPI00297646	Collagen alpha-1(I) chain precursor	COL1A1	P02452	19	20	18
IPI00845354	IGKC protein	IGKC		19	30	22
IPI00334667	Isoform 2 of Receptor-type tyrosine-protein phosphatase N2 precursor	PTPRN2	Q92932	19	24	18
IPI00784969	Putative uncharacterized protein	LOC100126583		19	22	16

Table S1.

				Number of unique peptides identified		
**IPI**	**Protein name**	**Gene symbol**	**Swiss Prot ID**	**Normal**	**CFS**	**nPTLS**
IPI00785067	IGH@ protein	IGH@		19	22	17
IPI00169383	Phosphoglycerate kinase 1	PGK1	P00558	19	16	13
IPI00004669	Polypeptide N-acetylgalactosaminyltransferase 2	GALNT2	Q10471	19	21	17
IPI00022426	AMBP protein precursor	AMBP	P02760	19	38	37
IPI00022284	Major prion protein precursor	PRNP	P04156	19	40	43
IPI00328609	Kallistatin precursor	SERPINA4	P29622	19	28	27
IPI00219365	Moesin	MSN	P26038	19	4	11
IPI00006662	Apolipoprotein D precursor	APOD	P05090	19	18	22
IPI00001662	Opioid-binding protein/cell adhesion molecule precursor	OPCML	Q14982	19	33	38
IPI00477747	Isoform 1 of Follistatin-related protein 4 precursor	FSTL4	Q6MZW2	19	23	25
IPI00004656	Beta-2-microglobulin	B2M	P61769	19	45	44
IPI00183445	Isoform 1 of Latrophilin-1 precursor	LPHN1	O94910	19	30	24
IPI00021891	Isoform Gamma-B of Fibrinogen gamma chain precursor	FGG	P02679	19	21	20
IPI00018136	Isoform 1 of Vascular cell adhesion protein 1 precursor	VCAM1	P19320	19	27	25
IPI00430808	Immunoblobulin light chain (Fragment)	IGKC		18	31	23
IPI00855824	dipeptidyl-peptidase 6 isoform 3	DPP6		18	14	21
IPI00289083	Isoform 1 of VWFA and cache domain-containing protein 1 precursor	CACHD1	Q5VU97	18	22	18
IPI00477597	Isoform 1 of Haptoglobin-related protein precursor	HPR	P00739	18	22	21
IPI00016422	Netrin receptor DCC precursor	DCC	P43146	18	14	15
IPI00000779	Isoform 1 of ADAM 22 precursor	ADAM22	Q9P0K1	18	30	28
IPI00301579	Epididymal secretory protein E1 precursor	NPC2	P61916	18	37	36
IPI00290085	Cadherin-2 precursor	CDH2	P19022	18	25	25
IPI00022371	Histidine-rich glycoprotein precursor	HRG	P04196	18	41	36
IPI00784830	CDNA FLJ41981 fis, clone SMINT2011888, highly similar to Protein Tro alpha1 H,myeloma	LOC100126583		18	21	15
IPI00784758	Putative uncharacterized protein DKFZp686M08189	LOC100126583		18	19	15
IPI00176221	Neuronal growth regulator 1 precursor	NEGR1	Q7Z3B1	18	40	30
IPI00480183	Protein	PTPRF		18	15	16
IPI00021854	Apolipoprotein A-II precursor	APOA2	P02652	17	23	16
IPI00298281	Laminin subunit gamma-1 precursor	LAMC1	P11047	17	20	29
IPI00012585	Beta-hexosaminidase beta chain precursor	HEXB	P07686	17	19	21
IPI00008787	Alpha-N-acetylglucosaminidase precursor	NAGLU	P54802	17	10	16

Table S1.

IPI	Protein name	Gene symbol	Swiss Prot ID	Number of unique peptides identified: Normal	CFS	nPTLS
IPI00784773	Putative uncharacterized protein	-		17	31	21
IPI00028911	Dystroglycan precursor	DAG1	Q14118	17	51	55
IPI00166729	alpha-2-glycoprotein 1, zinc	AZGP1	P25311	17	24	23
IPI00021304	Keratin, type II cytoskeletal 2 epidermal	KRT2	P35908	17	9	6
IPI00022417	Leucine-rich alpha-2-glycoprotein precursor	LRG1	P02750	17	15	16
IPI00022431	Alpha-2-HS-glycoprotein precursor	AHSG	P02765	17	59	51
IPI00010154	Rab GDP dissociation inhibitor alpha	GDI1	P31150	17	19	19
IPI00009028	Tetranectin precursor	CLEC3B	P05452	17	36	36
IPI00299024	Brain acid soluble protein 1	BASP1	P80723	17	15	12
IPI00746963	IGKC protein	IGKC		17	30	22
IPI00003590	Isoform 1 of Sulfhydryl oxidase 1 precursor	QSOX1	O00391	17	31	26
IPI00027827	Extracellular superoxide dismutase [Cu-Zn] precursor	SOD3	P08294	17	41	42
IPI00784865	IGK@ protein	IGK@		17	30	22
IPI00219217	L-lactate dehydrogenase B chain	LDHB	P07195	17	23	26
IPI00465248	Isoform alpha-enolase of Alpha-enolase	ENO1	P06733	17	19	18
IPI00879665	112 kDa protein	SEZ6L		17	41	36
IPI00792115	Putative uncharacterized protein DKFZp686H17246	CLEC3B		17	36	36
IPI00410600	Isoform 3 of Voltage-dependent calcium channel subunit alpha-2/delta-2 precursor	CACNA2D2	Q9NY47	17	21	26
IPI00008603	Actin, aortic smooth muscle	ACTA2	P62736	17	13	20
IPI00028908	Nidogen-2 precursor	NID2	Q14112	17	19	21
IPI00298237	Isoform 1 of Tripeptidyl-peptidase 1 precursor	TPP1	O14773	17	29	29
IPI00419722	seizure related 6 homolog (mouse)-like 2 isoform 2	SEZ6L2		17	30	34
IPI00252731	Isoform DPPX-S of Dipeptidyl aminopeptidase-like protein 6	DPP6	P42658	17	13	20
IPI00020599	Calreticulin precursor	CALR	P27797	16	32	31
IPI00026946	Neuronal pentraxin-2 precursor	NPTX2	P47972	16	19	23
IPI00472961	IGKC protein	IGKC		16	33	23
IPI00166766	hypothetical protein LOC146556 isoform 2	MGC45438		16	30	26
IPI00384952	Putative uncharacterized protein DKFZp686K04218 (Fragment)	-		16	15	9
IPI00011651	Isoform 1 of Receptor-type tyrosine-protein phosphatase gamma precursor	PTPRG	P23470	16	24	18
IPI00027851	Beta-hexosaminidase alpha chain precursor	HEXA	P06865	16	30	28
IPI00396423	Alcadein beta	CLSTN3		16	27	25

Table S1.

IPI	Protein name	Gene symbol	Swiss Prot ID	Number of unique peptides identified<br>Normal	CFS	nPTLS
IPI00154734	seizure related 6 homolog isoform 1	SEZ6		16	37	35
IPI00295741	Cathepsin B precursor	CTSB	P07858	16	27	24
IPI00853045	Anti-RhD monoclonal T125 kappa light chain precursor	IGKC		16	29	21
IPI00410210	Isoform 2 of Latrophilin-1 precursor	LPHN1	O94910	16	24	23
IPI00289819	Cation-independent mannose-6-phosphate receptor precursor	IGF2R	P11717	16	16	15
IPI00011252	Complement component C8 alpha chain precursor	C8A	P07357	16	33	24
IPI00015688	Glypican-1 precursor	GPC1	P35052	16	18	18
IPI00020091	Alpha-1-acid glycoprotein 2 precursor	ORM2	P19652	16	17	17
IPI00854806	IGKV1-5 protein	IGKV1-5		16	29	18
IPI00022429	Alpha-1-acid glycoprotein 1 precursor	ORM1	P02763	16	15	18
IPI00014572	SPARC precursor	SPARC	P09486	16	34	38
IPI00013303	Limbic system-associated membrane protein precursor	LSAMP	Q13449	16	25	26
IPI00022420	Plasma retinol-binding protein precursor	RBP4	P02753	16	41	33
IPI00298793	Beta-mannosidase precursor	MANBA	O00462	16	20	17
IPI00001952	Endonuclease domain-containing 1 protein precursor	ENDOD1	O94919	16	27	33
IPI00180240	thymosin-like 3	TMSL3		16	14	17
IPI00152540	Isoform 1 of CD109 antigen precursor	CD109	Q6YHK3	16	16	16
IPI00027848	Macrophage mannose receptor 1 precursor	MRC1	P22897	16	29	29
IPI00643034	Isoform 1 of Phospholipid transfer protein precursor	PLTP	P55058	16	31	26
IPI00008944	Isoform 1 of Neuroendocrine protein 7B2 precursor	SCG5	P05408	16	40	36
IPI00385264	Ig mu heavy chain disease protein	-	P04220	15	15	14
IPI00418471	Vimentin	VIM	P08670	15	10	6
IPI00176458	protocadherin 1 isoform 2 precursor	PCDH1		15	13	19
IPI00011218	Macrophage colony-stimulating factor 1 receptor precursor	CSF1R	P07333	15	29	26
IPI00292946	Thyroxine-binding globulin precursor	SERPINA7	P05543	15	39	29
IPI00329775	Isoform 1 of Carboxypeptidase B2 precursor	CPB2	Q96IY4	15	18	18
IPI00102543	SLIT and NTRK-like protein 1 precursor	SLITRK1	Q96PX8	15	37	41
IPI00178302	Isoform 4 of Semaphorin-6D precursor	SEMA6D	Q8NFY4	15	18	19
IPI00024046	Cadherin-13 precursor	CDH13	P55290	15	23	28
IPI00221224	Aminopeptidase N	ANPEP	P15144	15	12	12
IPI00550162	IGLV3-25 protein	IGLV3-25		15	21	12
IPI00165949	Isoform 2 of Endoplasmic reticulum aminopeptidase 1	ERAP1	Q9NZ08	15	10	14

Table S1.

Number of unique peptides identified

IPI	Protein name	Gene symbol	Swiss Prot ID	Normal	CFS	nPTLS
IPI00297124	Isoform 1 of Interleukin-6 receptor subunit beta precursor	IL6ST	P40189	15	35	38
IPI00056478	Isoform 1 of Immunoglobulin superfamily member 8 precursor	IGSF8	Q969P0	15	22	22
IPI00297284	Insulin-like growth factor-binding protein 2 precursor	IGFBP2	P18065	15	46	38
IPI00465016	Isoform 2 of Sulfhydryl oxidase 1 precursor	QSOX1	O00391	15	26	23
IPI00795013	149 kDa protein	LPHN3		14	18	17
IPI00385980	ROBO2 isoform a	ROBO2		14	13	18
IPI00104074	Isoform 1 of Scavenger receptor cysteine-rich type 1 protein M130 precursor	CD163	Q86VB7	14	26	31
IPI00218046	Heparan-sulfate 6-O-sulfotransferase 3	HS6ST3	Q8IZP7	14	17	13
IPI00012887	Cathepsin L1 precursor	CTSL1	P07711	14	17	17
IPI00219446	Phosphatidylethanolamine-binding protein 1	PEBP1	P30086	14	26	32
IPI00154742	IGL@ protein	IGL@		14	20	12
IPI00009950	Vesicular integral-membrane protein VIP36 precursor	LMAN2	Q12907	14	28	28
IPI00022337	Interphotoreceptor retinoid-binding protein precursor	RBP3	P10745	14	10	10
IPI00012303	Selenium-binding protein 1	SELENBP1	Q13228	14	17	17
IPI00829626	IGL@ protein	IGL@		14	20	12
IPI00005794	60 kDa protein	PGCP		14	18	16
IPI00295832	Oligodendrocyte-myelin glycoprotein precursor	OMG	P23515	14	28	29
IPI00009865	Keratin, type I cytoskeletal 10	KRT10	P13645	14	12	13
IPI00552905	Isoform 1 of Proline-rich transmembrane protein 3 precursor	PRRT3	Q5FWE3	14	24	25
IPI00004440	Receptor-type tyrosine-protein phosphatase-like N precursor	PTPRN	Q16849	14	23	21
IPI00030255	Procollagen-lysine,2-oxoglutarate 5-dioxygenase 3 precursor	PLOD3	O60568	14	9	11
IPI00027350	Peroxiredoxin-2	PRDX2	P32119	14	14	11
IPI00103175	Isoform 1 of Soluble calcium-activated nucleotidase 1	CANT1	Q8WVQ1	14	18	16
IPI00291005	Malate dehydrogenase, cytoplasmic	MDH1	P40925	14	13	16
IPI00292791	Contactin-3 precursor	CNTN3	Q9P232	14	15	15
IPI00027192	Procollagen-lysine,2-oxoglutarate 5-dioxygenase 1 precursor	PLOD1	Q02809	14	9	12
IPI00787853	Inositol monophosphatase 3	IMPAD1	Q9NX62	14	19	16
IPI00296141	Dipeptidyl-peptidase 2 precursor	DPP7	Q9UHL4	14	19	24
IPI00794070	CFI protein	CFI		14	35	33

Table S1.

IPI	Protein name	Gene symbol	Swiss Prot ID	Number of unique peptides identified<br>Normal	CFS	nPTLS
IPI00009802	Isoform V0 of Versican core protein precursor	VCAN	P13611	14	26	28
IPI00465184	Guanine deaminase	GDA	Q9Y2T3	14	12	17
IPI00293836	Isoform 3 of Cell adhesion molecule 2 precursor	CADM2	Q8N3J6	13	20	15
IPI00220327	Keratin, type II cytoskeletal 1	KRT1	P04264	13	16	15
IPI00022361	Band 3 anion transport protein	SLC4A1	P02730	13		
IPI00000130	Somatostatin precursor	SST	P61278	13	33	28
IPI00829877	IGL@ protein	IGL@		13	18	11
IPI00013698	Acid ceramidase precursor	ASAH1	Q13510	13	5	9
IPI00304925	Heat shock 70 kDa protein 1	HSPA1A	P08107	13	9	10
IPI00012989	Lysosomal alpha-mannosidase precursor	MAN2B1	O00754	13	3	2
IPI00784519	Putative uncharacterized protein	-		13	19	11
IPI00220334	Isoform 3 of Seizure 6-like protein precursor	SEZ6L	Q9BYH1	13	32	27
IPI00011264	Complement factor H-related protein 1 precursor	CFHR1	Q03591	13	24	19
IPI00152418	Decay-accelerating factor splicing variant 4	CD55		13	26	29
IPI00163563	PEBP family protein precursor	PEBP4	Q96S96	13	30	22
IPI00328391	N-acetylgalactosaminyltransferase 7	GALNT7	Q86SF2	13	20	16
IPI00026216	Puromycin-sensitive aminopeptidase	NPEPPS	P55786	13	7	8
IPI00176427	Cell adhesion molecule 4 precursor	CADM4	Q8NFZ8	13	19	25
IPI00783492	Isoform 2 of Latent-transforming growth factor beta-binding protein 4 precursor	LTBP4	Q8N2S1	13	37	32
IPI00816626	PLXNB2 protein	PLXNB2		13	20	19
IPI00001593	Lysosomal Pro-X carboxypeptidase precursor	PRCP	P42785	13	22	19
IPI00166392	Immunoglobulin superfamily member 4	CADM1	Q9BY67	13	29	35
IPI00382938	IGLV4-3 protein	IGLV4-3		13	17	9
IPI00785200	Putative uncharacterized protein	-		13	19	10
IPI00027230	Endoplasmin precursor	HSP90B1	P14625	13	24	28
IPI00410714	Hemoglobin subunit alpha	HBA2	P69905	13	15	9
IPI00290283	mannan-binding lectin serine protease 1 isoform 2 precursor	MASP1		13	13	17
IPI00376689	Isoform 1 of Protein KIAA1199 precursor	KIAA1199	Q8WUJ3	13	7	7
IPI00419724	semaphorin 4B precursor	SEMA4B	Q9NPR2	13	15	13
IPI00218732	Serum paraoxonase/arylesterase 1	PON1	P27169	13	18	14
IPI00021263	14-3-3 protein zeta/delta	YWHAZ	P63104	13	19	23
IPI00031461	Rab GDP dissociation inhibitor beta	GDI2	P50395	13	12	15
IPI00012440	Plasma alpha-L-fucosidase precursor	FUCA2	Q9BTY2	12	14	10
IPI00785196	Putative uncharacterized protein	-		12	17	10

Table S1.

IPI	Protein name	Gene symbol	Swiss Prot ID	Number of unique peptides identified<br>Normal	CFS	nPTLS
IPI00743302	intercellular adhesion molecule 5 precursor	ICAM5		12	16	15
IPI00157414	Ectonucleotide pyrophosphatase/phosphodiesterase family member 6 precursor	ENPP6	Q6UWR7	12	8	10
IPI00032063	Similar to Candidate tumor suppressor protein	LRP1B		12	4	8
IPI00414984	sarcoglycan, epsilon isoform 1	SGCE		12	20	22
IPI00289204	Reticulon-4 receptor precursor	RTN4R	Q9BZR6	12	18	19
IPI00418446	N-acylsphingosine amidohydrolase (acid ceramidase) 1 isoform b	ASAH1		12	4	8
IPI00443799	hypothetical protein LOC124565 isoform a	MGC15523		12	16	12
IPI00301395	Probable serine carboxypeptidase CPVL precursor	CPVL	Q9H3G5	12	14	16
IPI00654755	Hemoglobin subunit beta	HBB	P68871	12	17	9
IPI00791228	glutamate receptor, ionotrophic, AMPA 4 isoform 3 precursor	GRIA4		12	14	17
IPI00015881	Isoform 1 of Macrophage colony-stimulating factor 1 precursor	CSF1	P09603	12	20	26
IPI00787781	similar to Metalloproteinase inhibitor 2 precursor	TIMP2		12	20	24
IPI00026104	Isoform Long of Iduronate 2-sulfatase precursor	IDS	P22304	12	23	28
IPI00000816	14-3-3 protein epsilon	YWHAE	P62258	12	14	19
IPI00793166	15 kDa protein	SPARCL1		12	9	15
IPI00746623	Hyaluronan-binding protein 2 precursor	HABP2	Q14520	12	22	25
IPI00299738	Procollagen C-endopeptidase enhancer 1 precursor	PCOLCE	Q15113	12	35	36
IPI00022822	Isoform 2 of Collagen alpha-1(XVIII) chain precursor	COL18A1	P39060	12	22	22
IPI00218413	biotinidase precursor	BTD	P43251	12	25	29
IPI00162547	latrophilin 3 precursor	LPHN3	Q9HAR2	12	19	18
IPI00022608	Sortilin-related receptor precursor	SORL1	Q92673	12	25	16
IPI00019581	Coagulation factor XII precursor	F12	P00748	12	26	23
IPI00006967	Protocadherin-9 precursor	PCDH9	Q9HC56	12	16	18
IPI00024570	Semaphorin-3G precursor	SEMA3G	Q9NS98	12	15	26
IPI00019576	Coagulation factor X precursor	F10	P00742	12	21	21
IPI00028413	Isoform 1 of Inter-alpha-trypsin inhibitor heavy chain H3 precursor	ITIH3	Q06033	12	19	15
IPI00167093	complement factor H-related 1	CFHR1		12	22	16
IPI00018236	Ganglioside GM2 activator precursor	GM2A	P17900	12	32	32
IPI00877029	FGA protein	FGA		12	14	11
IPI00027166	Metalloproteinase inhibitor 2 precursor	TIMP2	P16035	12	25	25

Table S1.

IPI	Protein name	Gene symbol	Swiss Prot ID	Number of unique peptides identified Normal	CFS	nPTLS
IPI00829711	Uncharacterized protein IGHA2 (Fragment)	IGHA2		12	12	10
IPI00411680	Isoform 1 of Protein-L-isoaspartate(D-aspartate) O-methyltransferase	PCMT1	P22061	12	13	19
IPI00008994	Isoform 1 of Protein NDRG2	NDRG2	Q9UN36	12	24	25
IPI00852979	hypothetical protein LOC25758	C11orf41	Q6ZVL6	12	14	13
IPI00328746	Reticulon-4 receptor-like 2 precursor	RTN4RL2	Q86UN3	11	11	9
IPI00032532	Isoform 2 of Growth arrest-specific protein 6 precursor	GAS6	Q14393	11	19	18
IPI00220342	N(G),N(G)-dimethylarginine dimethylaminohydrolase 1	DDAH1	O94760	11	11	10
IPI00298388	Isoform 1 of Phosphoinositide-3-kinase-interacting protein 1 precursor	PIK3IP1	Q96FE7	11	32	32
IPI00643920	Transketolase	TKT	P29401	11	1	2
IPI00026154	Glucosidase 2 subunit beta precursor	PRKCSH	P14314	11	15	14
IPI00217966	Isoform 1 of L-lactate dehydrogenase A chain	LDHA	P00338	11	5	3
IPI00014048	Ribonuclease pancreatic precursor	RNASE1	P07998	11	46	37
IPI00012102	N-acetylglucosamine-6-sulfatase precursor	GNS	P15586	11	7	9
IPI00023728	Gamma-glutamyl hydrolase precursor	GGH	Q92820	11	17	16
IPI00015315	Extracellular matrix protein 2 precursor	ECM2	O94769	11	33	27
IPI00247243	31 kDa protein	-		11	19	16
IPI00718977	glutamate receptor, ionotrophic, AMPA 4 isoform 2 precursor	GRIA4		11	14	18
IPI00216318	Isoform Long of 14-3-3 protein beta/alpha	YWHAB	P31946	11	12	17
IPI00302181	Isoform 1 of Voltage-dependent calcium channel subunit alpha-2/delta-3 precursor	CACNA2D3	Q8IZS8	11	11	13
IPI00293748	Isoform 1 of Multiple inositol polyphosphate phosphatase 1 precursor	MINPP1	Q9UNW1	11	21	25
IPI00743766	Fetuin-B precursor	FETUB	Q9UGM5	11	25	18
IPI00000877	Hypoxia up-regulated protein 1 precursor	HYOU1	Q9Y4L1	11	11	10
IPI00829640	IGL@ protein	IGL@		11	18	12
IPI00465325	leucine-rich repeat neuronal 6A	LINGO1		11	13	11
IPI00027493	4F2 cell-surface antigen heavy chain	SLC3A2	P08195	11	16	16
IPI00423461	Putative uncharacterized protein DKFZp686C02220 (Fragment)	IGHA2		11	15	7
IPI00219526	Isoform 1 of Phosphoglucomutase-1	PGM1	P36871	11	4	4
IPI00337548	Cell growth regulator with EF hand domain protein 1	CGREF1	Q99674	11	14	15

Table S1.

Number of unique peptides identified

IPI	Protein name	Gene symbol	Swiss Prot ID	Normal	CFS	nPTLS
IPI00026199	Glutathione peroxidase 3 precursor	GPX3	P22352	11	18	18
IPI00783665	Laminin subunit alpha-5 precursor	LAMA5	O15230	11	10	16
IPI00003921	Isoform 1 of Protein 4.1	EPB41	P11171	11		
IPI00646304	peptidylprolyl isomerase B precursor	PPIB	P23284	11	11	12
IPI00029693	Isoform A22 of Neuropilin-2 precursor	NRP2	O60462	11	9	11
IPI00877169	calcium/calmodulin-dependent protein kinase IIA isoform 2	CAMK2A		11	10	10
IPI00010949	Isoform 1 of Sialate O-acetylesterase precursor	SIAE	Q9HAT2	11	11	12
IPI00815938	IGLV3-21 protein	IGLV3-21		10	18	10
IPI00011261	Complement component C8 gamma chain precursor	C8G	P07360	10	14	15
IPI00784156	Isoform 1 of AP-2 complex subunit beta-1	AP2B1	P63010	10	20	21
IPI00816555	IGLV2-14 protein	IGLV2-14		10	16	10
IPI00879084	20 kDa protein	CP		10	34	33
IPI00029235	Insulin-like growth factor-binding protein 6 precursor	IGFBP6	P24592	10	50	42
IPI00395488	Vasorin precursor	VASN	Q6EMK4	10	16	16
IPI00296176	Coagulation factor IX precursor	F9	P00740	10	11	13
IPI00163187	Fascin	FSCN1	Q16658	10	9	10
IPI00103597	Isoform 1 of VPS10 domain-containing receptor SorCS1 precursor	SORCS1	Q8WY21	10	16	19
IPI00176193	Isoform 1 of Collagen alpha-1(XIV) chain precursor	COL14A1	Q05707	10	7	7
IPI00298994	Talin-1	TLN1	Q9Y490	10		1
IPI00010471	Plastin-2	LCP1	P13796	10	20	22
IPI00298547	Protein DJ-1	PARK7	Q99497	10	17	13
IPI00220642	14-3-3 protein gamma	YWHAG	P61981	10	9	10
IPI00843910	Tissue alpha-L-fucosidase precursor	FUCA1	P04066	10	11	8
IPI00002211	Isoform 2 of Semaphorin-6A precursor	SEMA6A	Q9H2E6	10	8	9
IPI00015964	Neuromodulin	GAP43	P17677	10	9	6
IPI00304962	Collagen alpha-2(I) chain precursor	COL1A2	P08123	10	8	8
IPI00418531	Isoform 1 of Gliomedin	GLDN	Q6ZMI3	10	7	8
IPI00292550	Isoform 1 of Polypeptide N-acetylgalactosaminyltransferase 13	GALNT13	Q8IUC8	10	11	12
IPI00025426	Pregnancy zone protein precursor	PZP	P20742	10	20	14
IPI00022331	Phosphatidylcholine-sterol acyltransferase precursor	LCAT	P04180	10	21	22
IPI00010796	Protein disulfide-isomerase precursor	P4HB	P07237	10	12	10
IPI00456736	Isoform 1 of RGM domain family member B precursor	RGMB	Q6NW40	10	27	25
IPI00010381	VPS10 domain-containing receptor SorCS3 precursor	SORCS3	Q9UPU3	10	12	6

Table S1.

IPI	Protein name	Gene symbol	Swiss Prot ID	Normal	CFS	nPTLS
				Number of unique peptides identified		
IPI00003919	Glutaminyl-peptide cyclotransferase precursor	QPCT	Q16769	10	12	9
IPI00027497	Glucose-6-phosphate isomerase	GPI	P06744	10	5	3
IPI00299299	Stress 70 protein chaperone microsome-associated 60 kDa protein precursor	STCH	P48723	10	7	6
IPI00009793	Complement C1r-like protein	C1RL		10	14	13
IPI00007236	Isoform 2 of Neuroligin-1 precursor	NLGN1	Q8N2Q7	10	6	8
IPI00297263	Isoform 1 of Protein HEG homolog 1 precursor	HEG1	Q9ULI3	10	7	7
IPI00871139	92 kDa protein	MASP1		10	10	14
IPI00015902	Beta-type platelet-derived growth factor receptor precursor	PDGFRB	P09619	9	4	8
IPI00301459	1-O-acylceramide synthase precursor	LYPLA3	Q8NCC3	9	5	6
IPI00303071	Cat eye syndrome critical region protein 1 precursor	CECR1	Q9NZK5	9	5	7
IPI00290856	Lymphatic vessel endothelial hyaluronic acid receptor 1 precursor	LYVE1	Q9Y5Y7	9	7	11
IPI00470607	family with sequence similarity 20, member C	FAM20C	Q8IXL6	9	9	7
IPI00011454	Isoform 2 of Neutral alpha-glucosidase AB precursor	GANAB	Q14697	9	7	11
IPI00785079	Putative uncharacterized protein	-		9	14	9
IPI00029723	Follistatin-related protein 1 precursor	FSTL1	Q12841	9	22	17
IPI00297487	Cathepsin H precursor	CTSH	P09668	9	17	18
IPI00852577	IGLC1 protein	IGLC1		9	16	10
IPI00025110	Isoform 2 of Mesothelin precursor	MSLN	Q13421	9	6	7
IPI00027482	Corticosteroid-binding globulin precursor	SERPINA6	P08185	9	17	15
IPI00215983	Carbonic anhydrase 1	CA1	P00915	9	12	7
IPI00790473	12 kDa protein	SERPINF1		9	17	23
IPI00000137	N-acetylglucosamine-1-phosphotransferase subunit gamma precursor	GNPTG	Q9UJJ9	9	11	17
IPI00291175	Isoform 1 of Vinculin	VCL	P18206	9	3	4
IPI00477992	complement component 1, q subcomponent, B chain precursor	C1QB	P02746	9	12	14
IPI00015756	Isoform 1 of Receptor-type tyrosine-protein phosphatase kappa precursor	PTPRK	Q15262	9	8	8
IPI00220292	Isoform 1 of Ecto-ADP-ribosyltransferase 3 precursor	ART3	Q13508	9	21	16
IPI00002816	Cathepsin F precursor	CTSF	Q9UBX1	9	13	11
IPI00023014	von Willebrand factor precursor	VWF	P04275	9	10	10
IPI00745363	Immunglobulin heavy chain variable region (Fragment)	LOC652113		9	8	7

Table S1.

Number of unique peptides identified

IPI	Protein name	Gene symbol	Swiss Prot ID	Normal	CFS	nPTLS
IPI00021485	Leucine-rich repeat neuronal protein 1 precursor	LRRN1	Q6UXK5	9	7	6
IPI00106646	45 kDa calcium-binding protein precursor	SDF4	Q9BRK5	9	8	9
IPI00005292	Testican-1 precursor	SPOCK1	Q08629	9	33	38
IPI00292150	Latent-transforming growth factor beta-binding protein 2 precursor	LTBP2	Q14767	9	14	12
IPI00514676	myelin oligodendrocyte glycoprotein isoform beta2 precursor	MOG		9	14	11
IPI00293088	Lysosomal alpha-glucosidase precursor	GAA	P10253	9	8	5
IPI00020990	Osteomodulin precursor	OMD	Q99983	9	21	20
IPI00009123	Nucleobindin-2 precursor	NUCB2	P80303	9	8	13
IPI00745660	IGL@ protein	IGL@		9	15	9
IPI00376394	Sulfhydryl oxidase 2 precursor	QSOX2	Q6ZRP7	9	15	13
IPI00008087	Follistatin-related protein 5 precursor	FSTL5	Q8N475	9	5	15
IPI00414896	Isoform 1 of Ribonuclease T2 precursor	RNASET2	O00584	9	23	23
IPI00023019	Isoform 1 of Sex hormone-binding globulin precursor	SHBG	P04278	9	13	9
IPI00014592	Chondroadherin precursor	CHAD	O15335	9	1	
IPI00002732	EXTL2 protein (Fragment)	EXTL2	Q9UBQ6	9	18	20
IPI00007221	Plasma serine protease inhibitor precursor	SERPINA5	P05154	9	11	12
IPI00018769	Thrombospondin-2 precursor	THBS2	P35442	9	13	14
IPI00183487	Xylosyltransferase 1	XYLT1	Q86Y38	9	8	11
IPI00299571	Isoform 2 of Protein disulfide-isomerase A6 precursor	PDIA6	Q15084	9	6	8
IPI00788786	309 kDa protein	VWF		9	9	10
IPI00002406	Lutheran blood group glycoprotein precursor	BCAM	P50895	9	5	5
IPI00064607	Isoform 1 of Multiple epidermal growth factor-like domains 10 precursor	MEGF10	Q96KG7	9	36	36
IPI00216691	Profilin-1	PFN1	P07737	9	10	9
IPI00871326	plexin A1	PLXNA1		9	5	7
IPI00005142	Isoform 1 of Basic fibroblast growth factor receptor 1 precursor	FGFR1	P11362	9	20	18
IPI00024621	Isoform 1 of Olfactomedin-like protein 3 precursor	OLFML3	Q9NRN5	9	14	9
IPI00001611	Isoform 1 of Insulin-like growth factor II precursor	IGF2	P01344	9	13	14
IPI00000874	Peroxiredoxin-1	PRDX1	Q06830	9	6	11
IPI00001477	Isoform 1 of Epithelial discoidin domain-containing receptor 1 precursor	DDR1	Q08345	9	2	9
IPI00163446	IGHD protein	IGHD		9	14	15

Table S1.

Number of unique peptides identified

IPI	Protein name	Gene symbol	Swiss Prot ID	Normal	CFS	nPTLS
IPI00027038	Isoform 1 of V-set and immunoglobulin domain-containing protein 4 precursor	VSIG4	Q9Y279	9	10	7
IPI00795055	CDNA FLJ14022 fis, clone HEMBA1003538, weakly similar to COMPLEMENT C1R COMPONENT	C1RL		9	12	9
IPI00013682	Isoform 3 of Ecto-ADP-ribosyltransferase 3 precursor	ART3	Q13508	9	20	16
IPI00011654	Tubulin beta chain	TUBB	P07437	9	1	3
IPI00005707	Macrophage mannose receptor 2 precursor	MRC2	Q9UBG0	8	16	17
IPI00003269	hypothetical protein LOC345651	DKFZp686D0972		8	6	8
IPI00009904	Protein disulfide-isomerase A4 precursor	PDIA4	P13667	8	3	5
IPI00883772	acid alpha-glucosidase preproprotein	GAA		8	8	5
IPI00013475	Tubulin beta-2A chain	TUBB2A	Q13885	8	1	3
IPI00007702	Heat shock-related 70 kDa protein 2	HSPA2	P54652	8	7	6
IPI00302592	filamin A, alpha isoform 1	FLNA		8	5	5
IPI00015346	Cadherin EGF LAG seven-pass G-type receptor 2 precursor	CELSR2	Q9HCU4	8	14	13
IPI00329352	Nodal modulator 1 precursor	NOMO1	Q15155	8	5	3
IPI00023598	Tubulin beta-4 chain	TUBB4	P04350	8	1	3
IPI00007752	Tubulin beta-2C chain	TUBB2C	P68371	8	1	5
IPI00292304	Uncharacterized protein C9orf4	C9orf4	Q9P0K9	8	8	12
IPI00003865	Isoform 1 of Heat shock cognate 71 kDa protein	HSPA8	P11142	8	11	7
IPI00455667	hypothetical protein LOC402665	LOC402665		8	10	7
IPI00001895	Isoform 1 of Protocadherin-8 precursor	PCDH8	O95206	8	5	5
IPI00016870	Zona pellucida sperm-binding protein 2 precursor	ZP2	Q05996	8	7	6
IPI00003366	Isoform TrkB of BDNF/NT-3 growth factors receptor precursor	NTRK2	Q16620	8	13	12
IPI00021033	Isoform 1 of Collagen alpha-1(III) chain precursor	COL3A1	P02461	8	6	6
IPI00477804	Immunglobulin heavy chain variable region (Fragment)	-		8	11	8
IPI00854743	Uncharacterized protein ENSP00000375034	-		8	9	9
IPI00220739	Membrane-associated progesterone receptor component 1	PGRMC1	O00264	8	5	4
IPI00419585	Peptidyl-prolyl cis-trans isomerase A	PPIA	P62937	8	11	12
IPI00043756	Isoform 3 of Zinc transporter ZIP12	SLC39A12	Q504Y0	8	4	4
IPI00010295	Carboxypeptidase N catalytic chain precursor	CPN1	P15169	8	6	7
IPI00165438	Muscle type neuropilin 1	NRP1		8	15	17

Table S1.

IPI	Protein name	Gene symbol	Swiss Prot ID	Number of unique peptides identified<br>Normal	CFS	nPTLS
IPI00293303	Legumain precursor	LGMN	Q99538	8	9	9
IPI00219465	Transcobalamin-2 precursor	TCN2	P20062	8	14	13
IPI00328488	Isoform 1 of Epididymis-specific alpha-mannosidase precursor	MAN2B2	Q9Y2E5	8	1	2
IPI00002745	Cathepsin Z precursor	CTSZ	Q9UBR2	8	11	14
IPI00292732	fibromodulin precursor	FMOD		8	10	7
IPI00044369	Isoform 1 of Plexin domain-containing protein 2 precursor	PLXDC2	Q6UX71	8	17	17
IPI00827650	Isoform 3 of CD44 antigen precursor	CD44	P16070	8	9	7
IPI00007798	Thyrotropin-releasing hormone-degrading ectoenzyme	TRHDE	Q9UKU6	8	2	5
IPI00007778	Di-N-acetylchitobiase precursor	CTBS	Q01459	8	13	12
IPI00021552	UDP-GalNAc:beta-1,3-N-acetylgalactosaminyltransferase 1	B3GALNT1	O75752	8	11	9
IPI00022810	Dipeptidyl-peptidase 1 precursor	CTSC	P53634	8	13	12
IPI00217146	SLIT and NTRK-like protein 4 precursor	SLITRK4	Q8IW52	8	14	13
IPI00028614	erythrocyte membrane protein band 4.2 isoform 2	EPB42		8		
IPI00022296	Mast/stem cell growth factor receptor precursor	KIT	P10721	8	6	11
IPI00473011	Hemoglobin subunit delta	HBB	P02042	8	16	8
IPI00784119	Vacuolar ATP synthase subunit S1 precursor	ATP6AP1	Q15904	8	23	22
IPI00008290	Isoform 1 of Ephrin type-A receptor 5 precursor	EPHA5	P54756	8	14	11
IPI00297224	Sushi domain-containing protein 5	SUSD5	O60279	8	16	15
IPI00645206	Isoform 1 of Protocadherin-17 precursor	PCDH17	O14917	8	10	7
IPI00219757	Glutathione S-transferase P	GSTP1	P09211	8	11	9
IPI00004962	Golgi integral membrane protein 4	GOLIM4	O00461	8	10	12
IPI00152847	WAP, kazal, immunoglobulin, kunitz and NTR domain-containing protein 2 precursor	WFIKKN2	Q8TEU8	8	28	27
IPI00440580	Isoform 1 of Glycoprotein endo-alpha-1,2-mannosidase-like protein	MANEAL	Q5VSG8	8	7	9
IPI00294705	Papilin	PAPLN		8	18	13
IPI00019906	Isoform 2 of Basigin precursor	BSG	P35613	8	7	7
IPI00032292	Metalloproteinase inhibitor 1 precursor	TIMP1	P01033	8	31	29
IPI00375205	Isoform 1 of Polypeptide N-acetylgalactosaminyltransferase 10	GALNT10	Q86SR1	8	13	14
IPI00217493	Myoglobin	MB	P02144	8	12	2
IPI00432592	126 kDa protein	-		8	18	12
IPI00465436	Catalase	CAT	P04040	7	9	2
IPI00028448	Brain-specific angiogenesis inhibitor 3 precursor	BAI3	O60242	7	7	8

Table S1.

Number of unique peptides identified

IPI	Protein name	Gene symbol	Swiss Prot ID	Normal	CFS	nPTLS
IPI00025864	Cholinesterase precursor	BCHE	P06276	7	4	8
IPI00002236	Lactadherin precursor	MFGE8	Q08431	7	5	9
IPI00006644	Isoform 2 of Plexin-B1 precursor	PLXNB1	O43157	7	8	9
IPI00218493	Hypoxanthine-guanine phosphoribosyltransferase	HPRT1	P00492	7	5	4
IPI00246058	PDCD6IP protein	PDCD6IP	Q8WUM4	7	1	
IPI00022391	Serum amyloid P-component precursor	APCS	P02743	7	8	10
IPI00014964	Lymphocyte antigen 6H precursor	LY6H	O94772	7	12	10
IPI00026103	ACHE protein	ACHE	P22303	7	7	8
IPI00016679	SLIT and NTRK-like protein 5 precursor	SLITRK5	O94991	7	8	5
IPI00333140	Delta and Notch-like epidermal growth factor-related receptor precursor	DNER	Q8NFT8	7	18	20
IPI00027466	Carbonic anhydrase 4 precursor	CA4	P22748	7	12	11
IPI00168626	Isoform 1 of Putative polypeptide N-acetylgalactosaminyltransferase-like protein 4	GALNTL4	Q6P9A2	7	8	7
IPI00019038	Lysozyme C precursor	LYZ	P61626	7	5	7
IPI00031769	Esophageal cancer-related gene 4 protein precursor	C2orf40	Q9H1Z8	7	5	6
IPI00216298	Thioredoxin	TXN	P10599	7	7	9
IPI00029193	Hepatocyte growth factor activator precursor	HGFAC	Q04756	7	11	9
IPI00165972	Complement factor D preproprotein	CFD	P00746	7	17	11
IPI00745313	adipocyte enhancer binding protein 1 precursor	AEBP1		7	10	6
IPI00026270	Carboxypeptidase M precursor	CPM	P14384	7	4	5
IPI00103471	Thioredoxin-like selenoprotein M precursor	SELM	Q8WWX9	7	9	10
IPI00001734	Isoform 1 of Phosphoserine aminotransferase	PSAT1	Q9Y617	7	5	4
IPI00023648	Immunoglobulin superfamily containing leucine-rich repeat protein precursor	ISLR	O14498	7	14	18
IPI00746388	Ezrin	EZR		7		1
IPI00006128	Testican-2 precursor	SPOCK2	Q92563	7	15	19
IPI00290328	Receptor-type tyrosine-protein phosphatase eta precursor	PTPRJ	Q12913	7	3	3
IPI00299503	Isoform 1 of Phosphatidylinositol-glycan-specific phospholipase D precursor	GPLD1	P80108	7	8	6
IPI00830047	Uncharacterized protein ENSP00000374858 (Fragment)	-		7	11	6
IPI00030075	Fibroleukin precursor	FGL2	Q14314	7	9	9
IPI00296099	Thrombospondin-1 precursor	THBS1	P07996	7	5	10
IPI00029046	Uncharacterized protein KIAA0152 precursor	KIAA0152	Q14165	7	5	6

Table S1.

IPI	Protein name	Gene symbol	Swiss Prot ID	Number of unique peptides identified<br>Normal	CFS	nPTLS
IPI00022792	Microfibril-associated glycoprotein 4 precursor	MFAP4	P55083	7	16	12
IPI00328550	Thrombospondin-4 precursor	THBS4	P35443	7	16	12
IPI00000138	Alpha-1,3-mannosyl-glycoprotein 2-beta-N-acetylglucosaminyltransferase	MGAT1	P26572	7	6	6
IPI00003648	Isoform Delta of Poliovirus receptor-related protein 1 precursor	PVRL1	Q15223	7	13	15
IPI00791134	Calsyntenin 2	CLSTN2		7	16	12
IPI00478483	172 kDa protein	LAMC3		7	5	7
IPI00428511	Neurexin-1-beta precursor	NRXN1	P58400	7	8	8
IPI00171410	Isoform 1 of Uncharacterized protein C3orf21	C3orf21	Q8NBI6	7	6	5
IPI00013569	Isoform 1 of Pappalysin-2 precursor	PAPPA2	Q9BXP8	7	7	6
IPI00166892	DPPY splice variant c	DPP10	Q8N608	7	3	1
IPI00021903	Isoform Alpha of ADAM 23 precursor	ADAM23	O75077	7	6	7
IPI00004946	chemokine (C-X-C motif) ligand 16	CXCL16	Q9H2A7	7	8	10
IPI00045841	Isoform 1 of Low-density lipoprotein receptor-related protein 11 precursor	LRP11	Q86VZ4	7	9	12
IPI00029605	N-acetylgalactosamine-6-sulfatase precursor	GALNS	P34059	7	7	4
IPI00216983	Carbonic anhydrase 3	CA3	P07451	7	6	4
IPI00025818	Isoform 1 of Polypeptide N-acetylgalactosaminyltransferase 1	GALNT1	Q10472	7	12	13
IPI00178767	Acid sphingomyelinase-like phosphodiesterase 3a precursor	SMPDL3A	Q92484	7	3	3
IPI00171473	Spondin-1 precursor	SPON1	Q9HCB6	7	16	15
IPI00017569	Fas apoptotic inhibitory molecule 2	FAIM2	Q9BWQ8	7	8	9
IPI00167215	Isoform 1 of Hepatocyte cell adhesion molecule precursor	HEPACAM	Q14CZ8	7	3	2
IPI00216882	mannan-binding lectin serine protease 1 isoform 3	MASP1		7	10	12
IPI00789234	Immunoglobulin V-set domain containing protein	VSTM2A	Q8TAG5	7	24	20
IPI00019399	Serum amyloid A-4 protein precursor	SAA4	P35542	7	5	7
IPI00304865	transforming growth factor, beta receptor III	TGFBR3	Q03167	7	13	15
IPI00183321	Isoform 1 of N-acetylgalactosamine 4-sulfate 6-O-sulfotransferase	GALNAC4S-6ST	Q7LFX5	7	4	6
IPI00005908	ADAMTS-1 precursor	ADAMTS1	Q9UHI8	7	14	12
IPI00029273	Isoform 1 of Hepatocyte growth factor receptor precursor	MET	P08581	7	5	3
IPI00024825	Isoform A of Proteoglycan-4 precursor	PRG4	Q92954	7	10	9
IPI00412988	Isoform 1 of Netrin-G1 precursor	NTNG1	Q9Y2I2	7	9	12

Table S1.

				Number of unique peptides identified		
**IPI**	**Protein name**	**Gene symbol**	**Swiss Prot ID**	**Normal**	**CFS**	**nPTLS**
IPI00168479	Isoform 1 of Apolipoprotein A-I-binding protein precursor	APOA1BP	Q8NCW5	7	6	6
IPI00012283	Isoform 1 of Semaphorin-3B precursor	SEMA3B	Q13214	7	12	10
IPI00168884	Renin receptor precursor	ATP6AP2	O75787	7	19	21
IPI00398715	Neuropilin 1	NRP1		7	14	16
IPI00029275	Isoform 1 of Melanotransferrin precursor	MFI2	P08582	6	10	5
IPI00025846	Isoform 2A of Desmocollin-2 precursor	DSC2	Q02487	6	6	9
IPI00374065	similar to melanoma inhibitory activity 3 isoform 1	MIA3		6	6	12
IPI00011732	Isoform 1 of GDNF family receptor alpha-2 precursor	GFRA2	O00451	6	22	24
IPI00329482	Isoform 1 of Laminin subunit alpha-4 precursor	LAMA4	Q16363	6	3	6
IPI00016645	Isoform 1 of Ephrin type-A receptor 7 precursor	EPHA7	Q15375	6	12	13
IPI00102435	collagen, type XXI, alpha 1 precursor	COL21A1		6	2	2
IPI00745251	Mannosyl-oligosaccharide 1,2-alpha-mannosidase IC	MAN1C1	Q9NR34	6	7	9
IPI00015049	Isoform 2 of Repulsive guidance molecule A precursor	RGMA	Q96B86	6	20	16
IPI00004503	lysosomal-associated membrane protein 1	LAMP1		6	5	5
IPI00008207	Endoplasmic reticulum mannosyl-oligosaccharide 1,2-alpha-mannosidase	MAN1B1	Q9UKM7	6	8	3
IPI00306322	Collagen alpha-2(IV) chain precursor	COL4A2	P08572	6	8	4
IPI00030887	Tyrosine-protein kinase receptor TYRO3 precursor	TYRO3	Q06418	6	13	13
IPI00018206	Aspartate aminotransferase, mitochondrial precursor	GOT2	P00505	6	1	8
IPI00182438	Isoform 2 of Contactin-5 precursor	CNTN5	O94779	6	5	6
IPI00791479	CDNA FLJ90299 fis, clone NT2RP2000514, highly similar to Homo sapiens roundabout 2 (robo2) mRNA	ROBO2		6	5	7
IPI00299652	Isoform Long of ADAM 11 precursor	ADAM11	O75078	6	5	9
IPI00024601	Carbonic anhydrase-related protein 10	CA10	Q9NS85	6	10	7
IPI00719621	Isoform 1 of Plexin-A2 precursor	PLXNA2	O75051	6	3	3
IPI00024034	Cadherin-4 precursor	CDH4	P55283	6	6	8
IPI00015911	Dihydrolipoyl dehydrogenase, mitochondrial precursor	DLD	P09622	6	6	5
IPI00011140	Protein NOV homolog precursor	NOV	P48745	6	19	18
IPI00387168	Isoform 1 of Proprotein convertase subtilisin/kexin type 9 precursor	PCSK9	Q8NBP7	6	8	10

Table S1.

IPI	Protein name	Gene symbol	Swiss Prot ID	Number of unique peptides identified<br>Normal	CFS	nPTLS
IPI00011994	Ectonucleotide pyrophosphatase/phosphodiesterase family member 5 precursor	ENPP5	Q9UJA9	6	7	9
IPI00789954	7 kDa protein	TF		6	12	7
IPI00854709	Uncharacterized protein ENSP00000374799 (Fragment)	-		6	11	7
IPI00004413	Tumor necrosis factor receptor superfamily member 21 precursor	TNFRSF21	O75509	6	9	9
IPI00165975	Cysteine-rich flanking region, C-terminal domain containing protein	ISLR2		6	11	7
IPI00002925	Cocaine- and amphetamine-regulated transcript protein precursor	CARTPT	Q16568	6	13	9
IPI00011605	Cerebellin-1 precursor	CBLN1	P23435	6	11	11
IPI00028553	Isoform 2 of Multiple inositol polyphosphate phosphatase 1 precursor	MINPP1	Q9UNW1	6	9	9
IPI00029061	Selenoprotein P precursor	SEPP1	P49908	6	10	12
IPI00301143	Isoform 1 of Peptidase inhibitor 16 precursor	PI16	Q6UXB8	6	23	19
IPI00176424	Neuroligin-2 precursor	NLGN2	Q8NFZ4	6	9	9
IPI00060310	Phospholipase D4	PLD4	Q96BZ4	6	5	6
IPI00789847	Protein	-		6	3	7
IPI00295414	Collagen alpha-1(XV) chain precursor	COL15A1	P39059	6	6	10
IPI00006154	Isoform Long of Complement factor H-related protein 2 precursor	CFHR2	P36980	6	7	7
IPI00027078	Carboxypeptidase D precursor	CPD	O75976	6	4	4
IPI00337351	MAM domain-containing glycosylphosphatidylinositol anchor protein 2 precursor	MDGA2	Q7Z553	6	5	3
IPI00006971	Isoform 1 of Endosialin precursor	CD248	Q9HCU0	6	4	4
IPI00642861	CDNA FLJ37558 fis, clone BRCOC1000087	CXorf36		6	10	11
IPI00003907	Isoform 1 of Protocadherin gamma C5 precursor	PCDHGC5	Q9Y5F6	6	13	9
IPI00328257	Isoform A of AP-1 complex subunit beta-1	AP1B1	Q10567	6	4	4
IPI00168866	MAM domain containing glycosylphosphatidylinositol anchor 1	MDGA1	Q8NFP4	6	4	5
IPI00155729	Plexin-B3 precursor	PLXNB3	Q9ULL4	6	3	3
IPI00017704	Coactosin-like protein	COTL1	Q14019	6	6	6
IPI00060715	BTB/POZ domain-containing protein KCTD12	KCTD12	Q96CX2	6	1	6
IPI00019502	Myosin-9	MYH9	P35579	6		

Table S1.

Number of unique peptides identified

IPI	Protein name	Gene symbol	Swiss Prot ID	Normal	CFS	nPTLS
IPI00739827	Isoform LAMP-2B of Lysosome-associated membrane glycoprotein 2 precursor	LAMP2	P13473	6	9	9
IPI00025204	CD5 antigen-like precursor	CD5L	O43866	6	6	6
IPI00027462	Protein S100-A9	S100A9	P06702	6	1	
IPI00409640	Isoform 1 of Lipolysis-stimulated lipoprotein receptor	LSR	Q86X29	6	4	5
IPI00301255	Immunoglobulin superfamily member 21 precursor	IGSF21	Q96ID5	6	14	12
IPI00291807	C3 and PZP-like, alpha-2-macroglobulin domain containing 8	CPAMD8		6	4	1
IPI00402157	Cerebellin-3 precursor	CBLN3	Q6UW01	6	3	5
IPI00477611	184 kDa protein	COL5A1		6	4	3
IPI00216319	14-3-3 protein eta	YWHAH	Q04917	6	5	4
IPI00002525	Neudesin precursor	NENF	Q9UMX5	6	3	3
IPI00829590	Uncharacterized protein ENSP00000375044	-		6	7	5
IPI00002142	Protocadherin-10 precursor	PCDH10	Q9P2E7	6	5	6
IPI00295399	Cadherin-10 precursor	CDH10	Q9Y6N8	6	3	2
IPI00301961	Neuroendocrine convertase 1 precursor	PCSK1	P29120	6	20	16
IPI00382500	Ig heavy chain V-III region GAL	-	P01781	6	8	7
IPI00479116	Carboxypeptidase N subunit 2 precursor	CPN2	P22792	6	29	21
IPI00009030	Isoform LAMP-2A of Lysosome-associated membrane glycoprotein 2 precursor	LAMP2	P13473	6	9	8
IPI00453473	Histone H4	HIST2H4A	P62805	6		2
IPI00022892	Thy-1 membrane glycoprotein precursor	THY1	P04216	6	17	14
IPI00216602	Isoform 5 of Fibroblast growth factor receptor 2 precursor	FGFR2	P21802	6	7	9
IPI00013897	ADAM 10 precursor	ADAM10	O14672	6	4	5
IPI00303894	Protein FAM3A precursor	FAM3A	P98173	6	1	2
IPI00784258	latent transforming growth factor beta binding protein 1 isoform LTBP-1L	LTBP1		6	9	15
IPI00304840	Isoform 2C2 of Collagen alpha-2(VI) chain precursor	COL6A2	P12110	6	4	6
IPI00007750	Tubulin alpha-4A chain	TUBA4A	P68366	6		1
IPI00299086	Syntenin-1	SDCBP	O00560	6	2	4
IPI00555693	Isoform 3 of Testican-3 precursor	SPOCK3	Q9BQ16	6	12	15
IPI00018534	Histone H2B type 1-L	HIST1H2BL	Q99880	6	4	2
IPI00386630	TCN2 protein	TCN2		6	12	13
IPI00431738	X-linked interleukin-1 receptor accessory protein-like 1 precursor	IL1RAPL1	Q9NZN1	5	1	1
IPI00307592	ATP-binding cassette, sub-family A, member 2 isoform a	ABCA2		5	8	6

Table S1.

Number of unique peptides identified

IPI	Protein name	Gene symbol	Swiss Prot ID	Normal	CFS	nPTLS
IPI00259102	Mammalian ependymin-related protein 1 precursor	EPDR1	Q9UM22	5	5	6
IPI00006166	Probable G-protein coupled receptor 37 precursor	GPR37	O15354	5	17	15
IPI00385007	Putative uncharacterized protein DKFZp686A01208	AP1B1		5	2	4
IPI00410122	Isoform 1 of Plexin domain-containing protein 1 precursor	PLXDC1	Q8IUK5	5	6	7
IPI00017257	Cathepsin O precursor	CTSO	P43234	5	6	5
IPI00292300	contactin associated protein-like 5	CNTNAP5		5	2	2
IPI00550115	Isoform 1 of Acid sphingomyelinase-like phosphodiesterase 3b precursor	SMPDL3B	Q92485	5	6	9
IPI00023807	Semaphorin-4D precursor	SEMA4D	Q92854	5	9	9
IPI00419966	Isoform 2 of Target of Nesh-SH3 precursor	ABI3BP	Q7Z7G0	5	6	4
IPI00019533	Chitinase-3-like protein 2 precursor	CHI3L2	Q15782	5	4	6
IPI00186903	Isoform 2 of Apolipoprotein-L1 precursor	APOL1	O14791	5	7	7
IPI00020672	Isoform 1 of Dipeptidyl-peptidase 3	DPP3	Q9NY33	5	2	4
IPI00430291	Isoform Delta 2 of Calcium/calmodulin-dependent protein kinase type II delta chain	CAMK2D	Q13557	5	2	3
IPI00019954	Cystatin-M precursor	CST6	Q15828	5	7	6
IPI00027174	Isoform 1 of Fibroblast growth factor receptor 3 precursor	FGFR3	P22607	5	6	6
IPI00879309	Protein	NRXN2		5	8	8
IPI00419595	Isoform 1 of Podocalyxin-like protein 2 precursor	PODXL2	Q9NZ53	5	13	10
IPI00829701	Uncharacterized protein ENSP00000375014	-		5	5	3
IPI00006803	Carbohydrate sulfotransferase 10	CHST10	O43529	5	8	4
IPI00107886	Semaphorin 6B isoform 2	SEMA6B		5	5	8
IPI00008215	NADP-dependent malic enzyme	ME1	P48163	5	1	1
IPI00007240	Coagulation factor XIII B chain precursor	F13B	P05160	5	11	6
IPI00171928	Angiopoietin-related protein 7 precursor	ANGPTL7	O43827	5	3	4
IPI00304331	Galactosylgalactosylxylosylprotein 3-beta-glucuronosyltransferase 3	B3GAT3	O94766	5	3	3
IPI00328113	Fibrillin-1 precursor	FBN1	P35555	5	14	14
IPI00292218	Hepatocyte growth factor-like protein precursor	MST1	P26927	5	7	10
IPI00015351	Isoform 1 of UPF0424 protein C1orf128	C1orf128	Q9GZP4	5	5	6
IPI00007199	Protein Z-dependent protease inhibitor precursor	SERPINA10	Q9UK55	5	7	7
IPI00607655	Isoform 2 of Ephrin type-A receptor 7 precursor	EPHA7	Q15375	5	12	14

Table S1.

IPI	Protein name	Gene symbol	Swiss Prot ID	Number of unique peptides identified<br>Normal	CFS	nPTLS
IPI00032288	MANSC domain-containing protein 1 precursor	MANSC1	Q9H8J5	5	5	5
IPI00019190	Myocilin precursor	MYOC	Q99972	5	4	9
IPI00816799	Rheumatoid factor D5 light chain (Fragment)	-		5	3	2
IPI00021856	Apolipoprotein C-II precursor	APOC2	P02655	5	5	5
IPI00328745	Reticulon-4 receptor-like 1 precursor	RTN4RL1	Q86UN2	5	4	4
IPI00011730	EMILIN-3 precursor	EMILIN3	Q9NT22	5	4	5
IPI00056357	Uncharacterized protein C19orf10 precursor	C19orf10	Q969H8	5	4	7
IPI00783689	Immunglobulin heavy chain variable region (Fragment)	-		5	6	8
IPI00026237	Myelin-associated glycoprotein precursor	MAG	P20916	5	6	6
IPI00386133	Ig kappa chain V-IV region B17 precursor	-	P06314	5	7	6
IPI00329801	Annexin A5	ANXA5	P08758	5	1	2
IPI00020987	Prolargin precursor	PRELP	P51888	5	6	7
IPI00018305	Insulin-like growth factor-binding protein 3 precursor	IGFBP3	P17936	5	9	10
IPI00296558	Carboxypeptidase-like protein X2 precursor	CPXM2	Q8N436	5	3	3
IPI00029606	Isoform B of ADAM 17 precursor	ADAM17	P78536	5	2	4
IPI00413912	Transmembrane protein 132E precursor	TMEM132E	Q6IEE7	5	3	5
IPI00063048	Isoform 2 of Beta-galactoside alpha-2,6-sialyltransferase 2	ST6GAL2	Q96JF0	5	9	8
IPI00384391	Myosin-reactive immunoglobulin heavy chain variable region (Fragment)	-		5	7	5
IPI00018274	Isoform 1 of Epidermal growth factor receptor precursor	EGFR	P00533	5	12	11
IPI00003021	Sodium/potassium-transporting ATPase subunit alpha-2 precursor	ATP1A2	P50993	5	4	4
IPI00026050	Ceroid-lipofuscinosis neuronal protein 5	CLN5	O75503	5	8	6
IPI00216138	Transgelin	TAGLN	Q01995	5	6	7
IPI00027721	Isoform 1 of Alpha-type platelet-derived growth factor receptor precursor	PDGFRA	P16234	5	3	4
IPI00783287	Immunglobulin heavy chain variable region (Fragment)	-		5	7	6
IPI00024048	Cadherin-15 precursor	CDH15	P55291	5	3	6
IPI00294615	Fibulin-5 precursor	FBLN5	Q9UBX5	5	7	5
IPI00029236	Insulin-like growth factor-binding protein 5 precursor	IGFBP5	P24593	5	14	16
IPI00003176	Serine protease HTRA1 precursor	HTRA1	Q92743	5	8	7
IPI00031510	Semaphorin-3A precursor	SEMA3A	Q14563	5	1	4

Table S1.

				Number of unique peptides identified		
**IPI**	**Protein name**	**Gene symbol**	**Swiss Prot ID**	**Normal**	**CFS**	**nPTLS**
IPI00295386	Carbonyl reductase [NADPH] 1	CBR1	P16152	5	1	3
IPI00013096	Isoform 1 of Receptor-type tyrosine-protein phosphatase T precursor	PTPRT	O14522	5	5	5
IPI00642632	C7 protein	-		5	10	6
IPI00289924	Alpha-2,8-sialyltransferase 8E	ST8SIA5	O15466	5	4	3
IPI00016862	Isoform Mitochondrial of Glutathione reductase, mitochondrial precursor	GSR	P00390	5	4	7
IPI00021794	Lysosomal protective protein precursor	CTSA	P10619	5	6	7
IPI00219131	Isoform 1 of ICOS ligand precursor	ICOSLG	O75144	5	5	5
IPI00787265	similar to aminopeptidase puromycin sensitive	LOC729034		5	3	3
IPI00020906	Inositol monophosphatase	IMPA1	P29218	5	5	3
IPI00291006	Malate dehydrogenase, mitochondrial precursor	MDH2	P40926	5	2	2
IPI00021447	Alpha-amylase 2B precursor	AMY2B	P19961	5	5	5
IPI00289058	Ly-6/neurotoxin-like protein 1 precursor	LYNX1	Q9BZG9	5	12	9
IPI00032311	Lipopolysaccharide-binding protein precursor	LBP	P18428	5	4	4
IPI00740545	similar to Prostate, ovary, testis expressed protein on chromosome 2 isoform 2	LOC653269		5	2	3
IPI00010348	Deoxyribonuclease-2-alpha precursor	DNASE2	O00115	5	4	4
IPI00008780	Stanniocalcin-2 precursor	STC2	O76061	5	5	5
IPI00294834	Aspartyl/asparaginyl beta-hydroxylase	ASPH	Q12797	5	8	11
IPI00296197	Nucleotide exchange factor SIL1 precursor	SIL1	Q9H173	5	4	3
IPI00023824	Fibulin-2 precursor	FBLN2	P98095	5	10	11
IPI00829947	13 kDa protein	-		5	9	6
IPI00328243	Phospholipase D3	PLD3	Q8IV08	5	6	7
IPI00031821	Integral membrane protein 2B	ITM2B	Q9Y287	5	12	9
IPI00009276	Endothelial protein C receptor precursor	PROCR	Q9UNN8	5	5	4
IPI00009145	Mannosyl-oligosaccharide 1,2-alpha-mannosidase IB	MAN1A2	O60476	5	3	6
IPI00023751	Growth/differentiation factor 8 precursor	MSTN	O14793	5	6	6
IPI00007797	Fatty acid-binding protein, epidermal	FABP5	Q01469	5	5	5
IPI00375364	Isoform 3 of Chitotriosidase-1 precursor	CHIT1	Q13231	5	6	3
IPI00165044	Isoform 2 of Uncharacterized protein C4orf18	C4orf18	Q6UWH4	5	6	3
IPI00021275	Isoform 1 of Ephrin type-B receptor 2 precursor	EPHB2	P29323	5	2	4
IPI00478892	Leucine-rich repeats and immunoglobulin-like domains protein 2 precursor	LRIG2	O94898	5	1	1
IPI00556287	Putative uncharacterized protein	-		5	8	5

Table S1.

				Number of unique peptides identified		
**IPI**	**Protein name**	**Gene symbol**	**Swiss Prot ID**	**Normal**	**CFS**	**nPTLS**
IPI00220827	Thymosin beta-10	TMSB10	P63313	5	2	5
IPI00854841	Uncharacterized protein ENSP00000375033	-		5	9	8
IPI00005474	Phospholysine phosphohistidine inorganic pyrophosphate phosphatase	LHPP	Q9H008	5	3	5
IPI00043215	immunoglobulin superfamily, member 1 isoform 1	IGSF1		5	5	9
IPI00736885	Ig kappa chain V-II region TEW	LOC440786	P01617	5	9	5
IPI00412987	GMFB protein	GMFB	P60983	5	4	4
IPI00016467	SLIT and NTRK-like protein 3 precursor	SLITRK3	O94933	5	5	5
IPI00289870	Isoform C of Protocadherin-7 precursor	PCDH7	O60245	5	2	2
IPI00030205	Ig kappa chain V-III region HAH precursor	IGKV3-20	P18135	5	6	7
IPI00027377	aggrecan isoform 2 precursor	ACAN	P16112	5	2	2
IPI00302840	Sodium/potassium-transporting ATPase subunit alpha-3	ATP1A3	P13637	5	2	3
IPI00827892	VH87-2 protein (Fragment)	-		5	9	6
IPI00479997	Stathmin	STMN1	P16949	5	1	1
IPI00005126	Ephrin-B2 precursor	EFNB2	P52799	5	8	11
IPI00031564	Uncharacterized protein C7orf24	C7orf24	O75223	5	4	6
IPI00790775	Isoform 3 of Voltage-dependent calcium channel subunit alpha-2/delta-3 precursor	CACNA2D3	Q8IZS8	5	4	5
IPI00012075	C-type natriuretic peptide precursor	NPPC	P23582	5	7	5
IPI00034319	Isoform A of Protein CutA precursor	CUTA	O60888	5	18	20
IPI00029997	6-phosphogluconolactonase	PGLS	O95336	5		2
IPI00021857	Apolipoprotein C-III precursor	APOC3	P02656	5	5	7
IPI00736860	ELK2, member of ETS oncogene family, pseudogene 1	ELK2P1		5	6	6
IPI00003102	Ciliary neurotrophic factor receptor alpha precursor	CNTFR	P26992	5	12	10
IPI00022333	Brain-specific angiogenesis inhibitor 1 precursor	BAI1	O14514	5	8	8
IPI00017968	ADM precursor	ADM	P35318	5	5	5
IPI00867665	Similar to Protein disulfide-isomerase precursor	-		5	8	7
IPI00181174	Isoform 1 of Neuroligin-4, X-linked precursor	NLGN4X	Q8N0W4	5	3	5
IPI00329685	Putative uncharacterized protein DKFZp686G12235	ARSA		5	3	4
IPI00218570	Phosphoglycerate mutase 2	PGAM2	P15259	5	3	6
IPI00414467	collectin sub-family member 12	COLEC12		5	7	8
IPI00010790	Biglycan precursor	BGN	P21810	5	3	3
IPI00021983	Isoform 1 of Nicastrin precursor	NCSTN	Q92542	5	6	6

Table S1.

Number of unique peptides identified

IPI	Protein name	Gene symbol	Swiss Prot ID	Normal	CFS	nPTLS
IPI00044743	Isoform 1 of Transmembrane protein 132B	TMEM132B	Q14DG7	5	11	11
IPI00306710	Isoform 1 of Chordin precursor	CHRD	Q9H2X0	5	9	11
IPI00550363	Transgelin-2	TAGLN2	P37802	5	2	1
IPI00005981	Transgelin-3	TAGLN3	Q9UI15	5		
IPI00006482	Isoform Long of Sodium/potassium-transporting ATPase subunit alpha-1 precursor	ATP1A1	P05023	5	1	4
IPI00744692	Transaldolase	TALDO1	P37837	4	6	7
IPI00827940	Mu-chain precursor (Fragment)	-		4	6	6
IPI00829752	Uncharacterized protein ENSP00000375029	-		4	6	5
IPI00749328	hypothetical protein	LOC729085		4	6	4
IPI00871227	Isoform 1 of Hemicentin-1 precursor	HMCN1	Q96RW7	4	2	
IPI00794450	9 kDa protein	LYNX1		4	10	7
IPI00180707	Isoform 1 of FRAS1-related extracellular matrix protein 2 precursor	FREM2	Q5SZK8	4		1
IPI00442911	CDNA FLJ26266 fis, clone DMC05613	IGHV4-31		4	6	8
IPI00008494	Intercellular adhesion molecule 1 precursor	ICAM1	P05362	4	1	2
IPI00168520	Isoform 2 of Matrilin-2 precursor	MATN2	O00339	4	2	
IPI00401283	Multiple epidermal growth factor-like domains 9 precursor	MEGF9	Q9H1U4	4	4	3
IPI00005652	Isoform 1 of WSC domain-containing protein 2	WSCD2	Q2TBF2	4	1	
IPI00014439	Dihydropteridine reductase	QDPR	P09417	4	10	12
IPI00009111	Trophoblast glycoprotein precursor	TPBG	Q13641	4	3	3
IPI00023858	Fc-gamma receptor IIIb	FCGR3B	O75015	4	4	4
IPI00025840	Isoform 1 of Ephrin-A1 precursor	EFNA1	P20827	4	6	6
IPI00027223	Isocitrate dehydrogenase [NADP] cytoplasmic	IDH1	O75874	4	2	5
IPI00219301	Myristoylated alanine-rich C-kinase substrate	MARCKS	P29966	4	5	5
IPI00007709	Isoform 1 of ADAM 28 precursor	ADAM28	Q9UKQ2	4	3	
IPI00019209	Semaphorin-3C precursor	SEMA3C	Q99985	4	4	4
IPI00410585	Isoform 1 of Crumbs homolog 2 precursor	CRB2	Q5IJ48	4		
IPI00001506	Neuropeptide Y precursor	NPY	P01303	4	7	6
IPI00854667	Uncharacterized protein ENSP00000375015	-		4	5	5
IPI00827560	HRV Fab N27-VL (Fragment)	-		4	6	6
IPI00299724	Isoform 1 of Signal regulatory protein beta-1 precursor	SIRPB1	O00241	4	16	16

Table S1.

				Number of unique peptides identified		
**IPI**	**Protein name**	**Gene symbol**	**Swiss Prot ID**	**Normal**	**CFS**	**nPTLS**
IPI00007249	ectonucleotide pyrophosphatase/phosphodiesterase 4	ENPP4		4	2	7
IPI00031534	Alpha-N-acetylgalactosaminide alpha-2,6-sialyltransferase 1	ST6GALNAC1	Q9NSC7	4	6	5
IPI00167710	Isoform 1 of Fibulin-7 precursor	FBLN7	Q53RD9	4	8	8
IPI00218414	Carbonic anhydrase 2	CA2	P00918	4	3	3
IPI00296058	EGF-containing fibulin-like extracellular matrix protein 2 precursor	EFEMP2	O95967	4	11	8
IPI00153049	Isoform 2 of Matrix-remodeling-associated protein 8 precursor	MXRA8	Q9BRK3	4	3	4
IPI00465255	Isoform 1 of Proline-rich acidic protein 1 precursor	PRAP1	Q96NZ9	4	6	3
IPI00217466	Histone H1.3	HIST1H1D	P16402	4	1	
IPI00182944	Isoform 3 of Calcium/calmodulin-dependent protein kinase type II beta chain	CAMK2B	Q13554	4	5	5
IPI00643115	Stathmin 1/oncoprotein 18	STMN1		4	1	1
IPI00011302	CD59 glycoprotein precursor	CD59	P13987	4	23	19
IPI00021855	Apolipoprotein C-I precursor	APOC1	P02654	4	1	4
IPI00103871	Isoform 1 of Roundabout homolog 4 precursor	ROBO4	Q8WZ75	4	2	1
IPI00178926	immunoglobulin J chain	IGJ	P01591	4	2	4
IPI00432525	Sialic acid-binding Ig-like lectin 14 precursor	SIGLEC14	Q08ET2	4	7	8
IPI00217759	Isoform 1 of Alpha-(1,3)-fucosyltransferase 11	FUT11	Q495W5	4	4	4
IPI00293128	Exostosin-1	EXT1	Q16394	4	2	2
IPI00218345	Isoform 2 of Tubulin alpha-3C/D chain	TUBA3C	Q13748	4	2	
IPI00478890	Isoform 1 of Testican-3 precursor	SPOCK3	Q9BQ16	4	12	16
IPI00011518	Isoform A of Beta-secretase 1 precursor	BACE1	P56817	4	4	2
IPI00783184	Immunglobulin heavy chain variable region (Fragment)	-		4	6	6
IPI00152850	junctional adhesion molecule 3 precursor	JAM3	Q9BX67	4	5	4
IPI00251507	Isoform IB of Synapsin-1	SYN1	P17600	4		
IPI00217236	Tubulin-specific chaperone A	TBCA	O75347	4	1	1
IPI00015525	Multimerin-2 precursor	MMRN2	Q9H8L6	4	5	9
IPI00019501	Ephrin-B3 precursor	EFNB3	Q15768	4	4	5
IPI00383732	VH3 protein (Fragment)	-		4	6	6
IPI00296992	AXL receptor tyrosine kinase isoform 1	AXL	P30530	4	15	14
IPI00432723	Isoform 1 of Xylosyltransferase 2	XYLT2	Q9H1B5	4	1	1
IPI00414717	golgi apparatus protein 1	GLG1		4	7	5

Table S1.

Number of unique peptides identified

IPI	Protein name	Gene symbol	Swiss Prot ID	Normal	CFS	nPTLS
IPI00644472	Isoform 2 of Haloacid dehalogenase-like hydrolase domain-containing protein 2	HDHD2	Q9H0R4	4	1	1
IPI00217465	Histone H1.2	HIST1H1C	P16403	4		
IPI00012058	Brain-derived neurotrophic factor precursor	BDNF	P23560	4	9	11
IPI00217467	Histone H1.4	HIST1H1E	P10412	4		
IPI00847670	Similar to Phosphoglycerate mutase 1	LOC440043		4	2	2
IPI00217882	Sortilin precursor	SORT1	Q99523	4	7	9
IPI00219684	Fatty acid-binding protein, heart	FABP3	P05413	4	2	1
IPI00302944	Isoform 4 of Collagen alpha-1(XII) chain precursor	COL12A1	Q99715	4	1	
IPI00027341	Macrophage-capping protein	CAPG	P40121	4	1	3
IPI00021833	Isoform Long of Platelet-derived growth factor A chain precursor	PDGFA	P04085	4	5	3
IPI00218539	Isoform B of Collagen alpha-1(XI) chain precursor	COL11A1	P12107	4	2	1
IPI00008223	UV excision repair protein RAD23 homolog B	RAD23B	P54727	4	3	2
IPI00300838	Carbohydrate sulfotransferase 8	CHST8	Q9H2A9	4	3	3
IPI00006657	Protein FAM20B precursor	FAM20B	O75063	4	3	4
IPI00033560	Isoform Alpha of Receptor-type tyrosine-protein phosphatase R precursor	PTPRR	Q15256	4	7	5
IPI00026174	Cholecystokinins precursor	CCK	P06307	4	7	7
IPI00025809	Alpha-1,6-mannosyl-glycoprotein 2-beta-N-acetylglucosaminyltransferase	MGAT2	Q10469	4	6	5
IPI00026197	Similar to Ig kappa chain V-IV region precursor	IGKV4-1	P06312	4	7	7
IPI00023576	Leucine-rich repeat transmembrane neuronal protein 2 precursor	LRRTM2	O43300	4	1	
IPI00414909	Alpha-N-acetylgalactosaminidase precursor	NAGA	P17050	4	3	6
IPI00215767	Isoform Long of Beta-1,4-galactosyltransferase 1	B4GALT1	P15291	4	7	10
IPI00021817	Vitamin K-dependent protein C precursor	PROC	P04070	4	10	9
IPI00784430	Similar to Ig kappa chain V-III region VG precursor	IGKV3D-11	P04433	4	5	5
IPI00024094	Rhesus blood group-associated glycoprotein	RHAG	Q02094	4		
IPI00827510	HRV Fab 026-VL (Fragment)	-		4	3	4
IPI00829663	Uncharacterized protein ENSP00000374801	-		4	8	9

Table S1.

IPI	Protein name	Gene symbol	Swiss Prot ID	Number of unique peptides identified<br>Normal	CFS	nPTLS
IPI00883855	Similar to Hepatitis B virus receptor binding protein	-		4	5	5
IPI00437751	Isoform Somatic-1 of Angiotensin-converting enzyme, somatic isoform precursor	ACE	P12821	4	4	7
IPI00017567	Isoform Long of Endoglin precursor	ENG	P17813	4		
IPI00305380	Insulin-like growth factor-binding protein 4 precursor	IGFBP4	P22692	4	16	12
IPI00219025	Glutaredoxin-1	GLRX	P35754	4	4	5
IPI00029699	Ribonuclease 4 precursor	RNASE4	P34096	4	9	5
IPI00010706	Glutathione synthetase	GSS	P48637	4	2	3
IPI00257508	Dihydropyrimidinase-related protein 2	DPYSL2	Q16555	4		
IPI00456589	Isoform 1 of Polypeptide N-acetylgalactosaminyltransferase 11	GALNT11	Q8NCW6	4	1	1
IPI00293925	Isoform 1 of Ficolin-3 precursor	FCN3	O75636	4	4	5
IPI00031789	Isoform 1 of Interleukin-1 receptor accessory protein precursor	IL1RAP	Q9NPH3	4	1	
IPI00024036	Cadherin-8 precursor	CDH8	P55286	4	9	9
IPI00419720	Dermokine gamma-1	-		4	3	2
IPI00218474	Beta-enolase	ENO3	P13929	4	6	6
IPI00387118	Ig kappa chain V-III region WOL	-	P01623	4	4	5
IPI00101608	Isoform 2 of Cysteine-rich with EGF-like domain protein 1 precursor	CRELD1	Q96HD1	4	2	2
IPI00216457	Histone H2A type 2-A	HIST2H2AA3	Q6FI13	4	3	3
IPI00747849	Isoform 1 of Sodium/potassium-transporting ATPase subunit beta-1	ATP1B1	P05026	4	5	6
IPI00291395	fibronectin leucine rich transmembrane protein 1	FLRT1	Q9NZU1	4	6	6
IPI00022367	Isoform 2 of Astrotactin-1 precursor	ASTN1	O14525	4	4	3
IPI00297188	Brain-specific angiogenesis inhibitor 2 precursor	BAI2	O60241	4	5	5
IPI00002307	Isoform 1 of Neuroligin-3 precursor	NLGN3	Q9NZ94	4	2	1
IPI00549330	Myosin-reactive immunoglobulin light chain variable region	IGKV3D-15		4	4	5
IPI00007425	desmocollin 1 isoform Dsc1b preproprotein	DSC1		4	1	1
IPI00001893	Isoform A of Protocadherin-7 precursor	PCDH7	O60245	4	2	3
IPI00020984	Calnexin precursor	CANX	P27824	4	6	7
IPI00099670	carboxyl ester lipase precursor	CEL		4	5	3
IPI00001399	Adherens junction-associated protein 1	AJAP1	Q9UKB5	4	6	5
IPI00219930	Cellular retinoic acid-binding protein 1	CRABP1	P29762	4	2	
IPI00387115	Ig kappa chain V-III region SIE	-	P01620	4	5	7
IPI00006713	Isoform 1 of DnaJ homolog subfamily C member 3	DNAJC3	Q13217	4	4	5

Table S1.

Number of unique peptides identified

IPI	Protein name	Gene symbol	Swiss Prot ID	Normal	CFS	nPTLS
IPI00024129	Peptidyl-prolyl cis-trans isomerase C	PPIC	P45877	4	3	4
IPI00299699	Neural proliferation differentiation and control protein 1 precursor	NPDC1	Q9NQX5	4	16	19
IPI00554786	Thioredoxin reductase 1, cytoplasmic precursor	TXNRD1	Q16881	4	4	3
IPI00827482	Uncharacterized protein ENSP00000348964 (Fragment)	-		4	5	5
IPI00008107	Leucine-rich repeat and fibronectin type-III domain-containing protein 2 precursor	LRFN2	Q9ULH4	4	2	1
IPI00783024	Myosin-reactive immunoglobulin heavy chain variable region (Fragment)	-		4	10	6
IPI00012386	Cochlin precursor	COCH	O43405	4	5	5
IPI00413451	Putative uncharacterized protein DKFZp686I04222	SERPINB6		4	1	1
IPI00166339	Isoform 1 of Ephrin type-A receptor 10 precursor	EPHA10	Q5JZY3	4	13	14
IPI00162329	Isoform 1 of Transmembrane protein 25 precursor	TMEM25	Q86YD3	4	2	4
IPI00217345	Isoform 2 of UDP-GlcNAc:betaGal beta-1,3-N-acetylglucosaminyltransferase 2	B3GNT2	Q9NY97	4	9	10
IPI00019359	Keratin, type I cytoskeletal 9	KRT9	P35527	4	9	13
IPI00023643	Sema domain, transmembrane domain (TM), and cytoplasmic domain, (Semaphorin) 6C	SEMA6C	Q9H3T2	4	1	3
IPI00384404	Rheumatoid factor RF-ET9 (Fragment)	-		4	7	5
IPI00007102	Uncharacterized protein C17orf25	GLOD4		4	1	
IPI00218834	Low affinity immunoglobulin gamma Fc region receptor III-A precursor	FCGR3A	P08637	4	7	4
IPI00643348	80 kDa protein	COMP		4	15	14
IPI00000824	Isoform A of NT-3 growth factor receptor precursor	NTRK3	Q16288	4	4	2
IPI00075248	Calmodulin	CALM3	P62158	4	4	4
IPI00470484	Isoform 1 of EGF-like, fibronectin type-III and laminin G-like domain-containing protein precursor	EGFLAM	Q63HQ2	4	1	2
IPI00428967	Toll-like receptor adapter molecule 2	TICAM2		4	3	3
IPI00017841	Isoform 1 of Noelin precursor	OLFM1	Q99784	4	8	7
IPI00784154	60 kDa heat shock protein, mitochondrial precursor	HSPD1	P10809	4	3	6
IPI00006451	Vesicle-fusing ATPase	NSF	P46459	4	2	5
IPI00880120	Abhydrolase domain-containing protein 14A	ABHD14A	Q9BUJ0	4	2	3

Table S1.

IPI	Protein name	Gene symbol	Swiss Prot ID	Number of unique peptides identified<br>Normal	CFS	nPTLS
IPI00741710	Isoform 2 of Sushi, nidogen and EGF-like domain-containing protein 1 precursor	SNED1	Q8TER0	4	6	5
IPI00455739	Isoform 1 of Beta-1,3-N-acetylglucosaminyltransferase lunatic fringe	LFNG	Q8NES3	4	6	4
IPI00171412	Isoform 1 of Sulfatase-modifying factor 2 precursor	SUMF2	Q8NBJ7	4		2
IPI00025476	Pancreatic alpha-amylase precursor	AMY2A	P04746	4	4	5
IPI00005123	Ephrin-A3 precursor	EFNA3	P52797	3	2	2
IPI00383032	Isoform 2 of Hepatitis A virus cellular receptor 2 precursor	HAVCR2	Q8TDQ0	3	10	9
IPI00478414	Ventroptin (Fragment)	CHRDL1		3	4	5
IPI00297181	Cadherin-7 precursor	CDH7	Q9ULB5	3	2	2
IPI00387119	Ig kappa chain V-III region POM	-	P01624	3	3	2
IPI00019176	Retinoic acid receptor responder protein 2 precursor	RARRES2	Q99969	3	12	9
IPI00030882	Isoform Flop of Glutamate receptor 2 precursor	GRIA2	P42262	3	3	2
IPI00884080	Similar to Immunglobulin heavy chain variable region	-		3	4	3
IPI00411656	Isoform 1 of Protein piccolo	PCLO	Q9Y6V0	3		
IPI00219425	Isoform Beta of Poliovirus receptor precursor	PVR	P15151	3	8	6
IPI00012269	Multimerin-1 precursor	MMRN1	Q13201	3	2	1
IPI00375879	Uncharacterized protein KIAA1467	KIAA1467	A2RU67	3	6	8
IPI00788824	Light chain Fab	IGLV1-44		3	1	1
IPI00032050	WW domain-binding protein 2	WBP2	Q969T9	3	4	4
IPI00026285	Sia-alpha-2,3-Gal-beta-1,4-GlcNAc-R:alpha 2,8-sialyltransferase	ST8SIA3	O43173	3	6	4
IPI00022959	Isoform 1 of Poliovirus receptor-related protein 3 precursor	PVRL3	Q9NQS3	3	2	3
IPI00402293	Arylsulfatase G precursor	ARSG	Q96EG1	3	1	1
IPI00384402	Myosin-reactive immunoglobulin kappa chain variable region (Fragment)	-		3	2	3
IPI00477714	V3-4 protein	IGLV8-61		3	2	2
IPI00829740	V2-6 protein	-		3	2	2
IPI00387120	Ig kappa chain V-IV region Len	-	P01625	3	4	4
IPI00019449	Non-secretory ribonuclease precursor	RNASE2	P10153	3	4	3
IPI00382481	Ig heavy chain V-III region BUT	-	P01767	3	6	5
IPI00006444	Isoform 1 of Sodium/potassium/calcium exchanger 2 precursor	SLC24A2	Q9UI40	3	2	4
IPI00011662	Kunitz-type protease inhibitor 2 precursor	SPINT2	O43291	3	4	4

Table S1.

Number of unique peptides identified

IPI	Protein name	Gene symbol	Swiss Prot ID	Normal	CFS	nPTLS
IPI00024929	Adipocyte adhesion molecule precursor	ASAM	Q9H6B4	3	1	2
IPI00022977	Creatine kinase B-type	CKB	P12277	3	1	3
IPI00010182	Isoform a 1 of Acyl-CoA-binding protein	DBI	P07108	3	6	7
IPI00020747	Sodium channel subunit beta-3 precursor	SCN3B	Q9NY72	3	4	1
IPI00643667	C1q and tumor necrosis factor related protein 3 isoform b	C1QTNF3		3	3	4
IPI00829812	Uncharacterized protein ENSP00000375011	-		3	2	3
IPI00292496	Beta-tubulin 4Q	TUBB8		3	1	1
IPI00297655	Neurogenic locus notch homolog protein 2 precursor	NOTCH2	Q04721	3	2	4
IPI00016666	Metallothionein-3	MT3	P25713	3	15	12
IPI00028015	Isoform 2 of Leukocyte-associated immunoglobulin-like receptor 1 precursor	LAIR1	Q6GTX8	3	3	4
IPI00293971	Sodium/potassium-transporting ATPase subunit beta-2	ATP1B2	P14415	3	2	2
IPI00884092	Anti-HER3 scFv (Fragment)	-		3	5	4
IPI00385253	Ig kappa chain V-III region CLL precursor	-	P04207	3	4	4
IPI00022891	ADP/ATP translocase 1	SLC25A4	P12235	3		1
IPI00783393	Immunglobulin heavy chain variable region (Fragment)	-		3	3	5
IPI00179851	NDT80/PhoG like DNA-binding family protein	C11orf9		3	2	1
IPI00021347	Ubiquitin-conjugating enzyme E2 L3	UBE2L3	P68036	3		
IPI00176398	Isoform 1 of SLIT and NTRK-like protein 6 precursor	SLITRK6	Q9H5Y7	3	2	2
IPI00017562	Isoform 2 of Latrophilin-2 precursor	LPHN2	O95490	3	2	3
IPI00026991	Polypeptide N-acetylgalactosaminyltransferase 6	GALNT6	Q8NCL4	3	13	9
IPI00010148	Brain-specific polypeptide PEP-19	PCP4	P48539	3	1	2
IPI00883879	Similar to Anti-IFN-G scFv	-		3	2	2
IPI00219219	Galectin-1	LGALS1	P09382	3	5	4
IPI00293757	Isoform 1 of Netrin receptor UNC5C precursor	UNC5C	O95185	3	3	3
IPI00299399	Protein S100-B	S100B	P04271	3	2	1
IPI00029928	Elastin	ELN	P15502	3	1	
IPI00011094	Complement C1q tumor necrosis factor-related protein 4 precursor	C1QTNF4	Q9BXJ3	3	2	2
IPI00022649	Isoform 1 of Solute carrier family 12 member 2	SLC12A2	P55011	3	1	2

Table S1.

IPI	Protein name	Gene symbol	Swiss Prot ID	Number of unique peptides identified<br>Normal	CFS	nPTLS
IPI00001633	Leucine-rich repeat transmembrane protein FLRT2 precursor	FLRT2	O43155	3	1	1
IPI00401264	Thioredoxin domain-containing protein 4 precursor	TXNDC4	Q9BS26	3	3	8
IPI00018396	Cerebellin-4 precursor	CBLN4	Q9NTU7	3	3	3
IPI00171438	Thioredoxin domain-containing protein 5 precursor	TXNDC5	Q8NBS9	3	1	1
IPI00030111	Growth/differentiation factor 11 precursor	GDF11	O95390	3	2	3
IPI00465315	Cytochrome c	CYCS	P99999	3	13	5
IPI00015842	Reticulocalbin-1 precursor	RCN1	Q15293	3	2	1
IPI00005222	Ephrin type-B receptor 6 precursor	EPHB6	O15197	3	5	4
IPI00440932	Isoform 1 of ADAM 9 precursor	ADAM9	Q13443	3	5	4
IPI00376131	Similar to Leucine rich repeat neuronal 6C	LINGO3		3	3	4
IPI00005517	Ephrin-A5 precursor	EFNA5	P52803	3	4	2
IPI00026259	N	AGA	P20933	3	3	4
IPI00827581	Variable immnoglobulin anti-estradiol heavy chain (Fragment)	-		3	5	5
IPI00184019	Isoform 3 of Paired immunoglobulin-like type 2 receptor alpha precursor	PILRA	Q9UKJ1	3	2	3
IPI00013162	Isoform 1 of OX-2 membrane glycoprotein precursor	CD200	P41217	3	4	4
IPI00827637	K light chain variable region (Fragment)	-		3	4	4
IPI00017745	Metalloproteinase inhibitor 4 precursor	TIMP4	Q99727	3	3	4
IPI00298650	ADAMTS-8 precursor	ADAMTS8	Q9UP79	3	10	8
IPI00414676	Heat shock protein HSP 90-beta	HSP90AB1	P08238	3	5	6
IPI00873863	Brain-derived neurotrophic factor transcript variant 5	BDNF		3	3	6
IPI00030871	Pantetheinase precursor	VNN1	O95497	3	4	6
IPI00022674	Isoform 1 of Oncostatin-M specific receptor subunit beta precursor	OSMR	Q99650	3	2	1
IPI00000760	N(G),N(G)-dimethylarginine dimethylaminohydrolase 2	DDAH2	O95865	3	2	2
IPI00000044	Platelet-derived growth factor B chain precursor	PDGFB	P01127	3	9	6
IPI00102575	ATPase family, AAA domain containing 5	ATAD5		3		1
IPI00003799	Isoform 2 of Heme-binding protein 2	HEBP2	Q9Y5Z4	3		1
IPI00374732	similar to peptidylprolyl isomerase A isoform 1	PPIAP19		3	3	4
IPI00289876	Isoform 1 of Syntaxin-7	STX7	O15400	3	1	1
IPI00472754	Polycystic kidney disease 1-related protein	KIAA0319L		3		
IPI00013701	Nociceptin precursor	PNOC	Q13519	3	10	7

Table S1.

IPI	Protein name	Gene symbol	Swiss Prot ID	Number of unique peptides identified<br>Normal	CFS	nPTLS
IPI00300725	Keratin, type II cytoskeletal 6A	KRT6A	P02538	3	1	4
IPI00784368	Isoform 1 of Bifunctional heparan sulfate N-deacetylase/N-sulfotransferase 1	NDST1	P52848	3	1	3
IPI00018381	Isoform 1 of Tolloid-like protein 1 precursor	TLL1	O43897	3	2	2
IPI00022394	Complement C1q subcomponent subunit C precursor	C1QC	P02747	3	8	11
IPI00884389	Similar to Immunglobulin heavy chain variable region	-		3	4	3
IPI00854644	Uncharacterized protein ENSP00000374805	-		3	5	4
IPI00028082	Reversion-inducing cysteine-rich protein with Kazal motifs precursor	RECK	O95980	3	2	
IPI00009826	Carboxypeptidase B precursor	CPB1	P15086	3	2	2
IPI00220301	Peroxiredoxin-6	PRDX6	P30041	3	8	3
IPI00006130	Uncharacterized calcium-binding protein KIAA0494	KIAA0494	O75071	3	2	1
IPI00157454	Isoform 1 of Heparan-sulfate 6-O-sulfotransferase 2	HS6ST2	Q96MM7	3	1	2
IPI00062037	Dynein light chain 2, cytoplasmic	DYNLL2	Q96FJ2	3	8	5
IPI00016621	Adaptor-related protein complex 2, alpha 2 subunit variant (Fragment)	AP2A2	O94973	3	1	2
IPI00387116	Ig kappa chain V-III region NG9 precursor (Fragment)	-	P01621	3	2	3
IPI00019755	Glutathione transferase omega-1	GSTO1	P78417	3	4	2
IPI00024035	Isoform 1 of Cadherin-6 precursor	CDH6	P55285	3	8	5
IPI00384392	Myosin-reactive immunoglobulin heavy chain variable region (Fragment)	-		3	5	6
IPI00827876	Heavy chain Fab (Fragment)	-		3	4	8
IPI00022392	Complement C1q subcomponent subunit A precursor	C1QA	P02745	3	7	5
IPI00018941	Calcitonin gene-related peptide 2 precursor	CALCB	P10092	3	2	1
IPI00883765	Similar to Immunglobulin heavy chain variable region	-		3	4	3
IPI00299150	Cathepsin S precursor	CTSS	P25774	3	7	6
IPI00004114	Ribonuclease K6 precursor	RNASE6	Q93091	3	4	4
IPI00010896	Chloride intracellular channel protein 1	CLIC1	O00299	3	3	2
IPI00748265	Rheumatoid factor RF-ET13	-		3	4	3
IPI00220362	10 kDa heat shock protein, mitochondrial	HSPE1	P61604	3	1	1
IPI00006556	hypothetical protein LOC9865	KIAA0644		3	2	1
IPI00029131	Neuroendocrine convertase 2 precursor	PCSK2	P16519	3	16	11

Table S1.

IPI	Protein name	Gene symbol	Swiss Prot ID	Number of unique peptides identified<br>Normal	CFS	nPTLS
IPI00374590	cancer susceptibility candidate 4 isoform a	CASC4	Q6P4E1	3	7	7
IPI00739099	Collagen alpha-2(V) chain precursor	COL5A2	P05997	3	1	4
IPI00024273	Isoform Long of Very low-density lipoprotein receptor precursor	VLDLR	P98155	3	3	2
IPI00018860	NKG2D ligand 2 precursor	ULBP2	Q9BZM5	3		1
IPI00220766	Lactoylglutathione lyase	GLO1	Q04760	3	2	2
IPI00220361	Calbindin	CALB1	P05937	3	6	7
IPI00556391	Actin-like protein (Fragment)	-		3	1	1
IPI00552852	V2-19 protein	IGLV3-27		3	4	4
IPI00293723	Neurexophilin-4 precursor	NXPH4	O95158	3	5	4
IPI00883711	Similar to Anti-(ED-B) scFV	-		3	3	3
IPI00107731	Isoform 6 of Osteoclast associated immunoglobulin-like receptor precursor	OSCAR	Q8IYS5	3	5	7
IPI00441344	Beta-galactosidase precursor	GLB1	P16278	3		
IPI00293464	DNA damage-binding protein 1	DDB1	Q16531	3	2	4
IPI00018146	14-3-3 protein theta	YWHAQ	P27348	3	5	6
IPI00020977	Isoform 1 of Connective tissue growth factor precursor	CTGF	P29279	3	4	5
IPI00019862	butyrophilin, subfamily 2, member A1 isoform 2 precursor	BTN2A1		3	1	1
IPI00374914	hypothetical protein	LOC401115		3	7	10
IPI00019600	Ubiquitin-conjugating enzyme E2 variant 2	UBE2V2	Q15819	3	3	1
IPI00827829	HRV Fab N8-VL (Fragment)	-		3	6	4
IPI00829841	13 kDa protein	-		3	7	6
IPI00029817	Sialidase-1 precursor	NEU1	Q99519	3	5	4
IPI00031506	Potassium/sodium hyperpolarization-activated cyclic nucleotide-gated channel 1	HCN1	O60741	3		1
IPI00219129	Ribosyldihydronicotinamide dehydrogenase	NQO2	P16083	3		2
IPI00293539	Isoform 2 of Cadherin-11 precursor	CDH11	P55287	3	6	8
IPI00012011	Cofilin-1	CFL1	P23528	3	2	3
IPI00184094	UDP-GlcNAc:betaGal beta-1,3-N-acetylglucosaminyltransferase 8	B3GNT8	Q7Z7M8	3	3	2
IPI00010470	Isoform SNAP-25b of Synaptosomal-associated protein 25	SNAP25	P60880	3	1	1
IPI00219910	22 kDa protein	-		3	2	
IPI00398918	Putative uncharacterized protein DKFZp686I21167	DKFZp686O24166		3		1
IPI00552267	Similar to V2-13 protein	IGLV3-19		3	3	4
IPI00031708	Fumarylacetoacetase	FAH	P16930	3	5	3
IPI00028931	Desmoglein-2 precursor	DSG2	Q14126	3	3	1

Table S1.

IPI	Protein name	Gene symbol	Swiss Prot ID	Number of unique peptides identified: Normal	CFS	nPTLS
IPI00022774	Transitional endoplasmic reticulum ATPase	VCP	P55072	3	1	
IPI00011899	BMP and activin membrane-bound inhibitor homolog precursor	BAMBI	Q13145	3	3	5
IPI00024587	D1 dopamine receptor-interacting protein calcyon	CALY	Q9NYX4	3	3	6
IPI00026240	ADP-ribosyl cyclase 2 precursor	BST1	Q10588	3	4	7
IPI00024105	Complement C1q tumor necrosis factor-related protein 5 precursor	C1QTNF5	Q9BXJ0	3	2	3
IPI00005722	Tyrosine-protein kinase receptor	FLT3	P36888	3	1	1
IPI00005516	Leucine-rich repeat-containing protein 4 precursor	LRRC4	Q9HBW1	3	3	2
IPI00328703	Out at first protein homolog precursor	OAF	Q86UD1	3	7	8
IPI00030739	Apolipoprotein M	APOM	O95445	3	3	3
IPI00031549	Isoform 3A of Desmocollin-3 precursor	DSC3	Q14574	3	3	2
IPI00012510	EMILIN-2 precursor	EMILIN2	Q9BXX0	3	2	
IPI00470625	Neuritin precursor	NRN1	Q9NPD7	3	5	6
IPI00217376	Isoform 1 of Sodium channel subunit beta-4 precursor	SCN4B	Q8IWT1	3	3	3
IPI00553138	Vesicle-associated membrane protein 2	VAMP2	P63027	3		1
IPI00169285	Putative phospholipase B-like 2 precursor	P76	Q8NHP8	3	3	2
IPI00020396	Isoform PACE4A-I of Proprotein convertase subtilisin/kexin type 6 precursor	PCSK6	P29122	3	1	2
IPI00000832	Beta-neoendorphin-dynorphin precursor	PDYN	P01213	3	10	14
IPI00760721	13 kDa protein	-		3	7	9
IPI00293849	Receptor-type tyrosine-protein phosphatase mu precursor	PTPRM	P28827	3	2	3
IPI00465377	Isoform 1 of Matrix-remodeling-associated protein 7	MXRA7	P84157	3	6	4
IPI00396378	Isoform B1 of Heterogeneous nuclear ribonucleoproteins A2/B1	HNRNPA2B1	P22626	3	1	
IPI00828105	Anti-Mpl scFv (Fragment)	-		3	6	7
IPI00299758	Carbohydrate sulfotransferase 12	CHST12	Q9NRB3	3	3	4
IPI00743194	Kappa light chain variable region (Fragment)	-		3	6	6
IPI00307276	ADAMTS-4 precursor	ADAMTS4	O75173	3	7	7
IPI00002334	Neuron-specific protein family member 1	D4S234E	P42857	3	4	3
IPI00387024	Ig kappa chain V-I region CAR	-	P01596	3	3	2
IPI00444605	CDNA FLJ45296 fis, clone BRHIP3003340, moderately similar to Actin, alpha skeletal muscle 2	-		3	3	4

Table S1.

Number of unique peptides identified

IPI	Protein name	Gene symbol	Swiss Prot ID	Normal	CFS	nPTLS
IPI00304577	Isoform A of AP-2 complex subunit alpha-1	AP2A1	O95782	3	2	1
IPI00385252	Ig kappa chain V-III region GOL	-	P04206	3	5	7
IPI00182138	Isoform 2 of Granulins precursor	GRN	P28799	3	6	3
IPI00301364	Isoform 1 of S-phase kinase-associated protein 1A	SKP1	P63208	3	4	5
IPI00007960	Isoform 1 of Periostin precursor	POSTN	Q15063	3	2	3
IPI00030847	Transmembrane 9 superfamily member 3 precursor	TM9SF3	Q9HD45	3	3	4
IPI00008533	Isoform Long of Matrix metalloproteinase-17 precursor	MMP17	Q9ULZ9	3	7	5
IPI00387117	Ig kappa chain V-III region Ti	IGKV3D-20	P01622	3	3	3
IPI00657742	Major histocompatibility complex, class I, F	HLA-F		3	1	2
IPI00218667	Stathmin-2	STMN2	Q93045	3		1
IPI00005809	Serum deprivation-response protein	SDPR	O95810	3		
IPI00008997	WAP four-disulfide core domain protein 1 precursor	WFDC1	Q9HC57	3	4	3
IPI00008556	Isoform 1 of Coagulation factor XI precursor	F11	P03951	3	4	3
IPI00165125	Isoform 1 of Uncharacterized protein C14orf37 precursor	C14orf37	Q86TY3	3	2	2
IPI00027463	Protein S100-A6	S100A6	P06703	3	2	2
IPI00012048	Nucleoside diphosphate kinase A	NME2	P15531	3	3	2
IPI00789477	Similar to Lactotransferrin precursor	LTF		3	7	5
IPI00297252	Isoform 1 of Extracellular sulfatase Sulf-2 precursor	SULF2	Q8IWU5	3	3	6
IPI00418960	Isoform 3 of Protein NDRG4	NDRG4	Q9ULP0	3	8	6
IPI00303476	ATP synthase subunit beta, mitochondrial precursor	ATP5B	P06576	3	3	4
IPI00168847	Isoform 2 of Hyaluronidase-1 precursor	HYAL1	Q12794	3	3	5
IPI00219575	Bleomycin hydrolase	BLMH	Q13867	3	1	3
IPI00168921	Putative polypeptide N-acetylgalactosaminyltransferase-like protein 3	WBSCR17	Q6IS24	3	2	1
IPI00410487	Isoform 1 of Twisted gastrulation protein homolog 1 precursor	TWSG1	Q9GZX9	3	4	3
IPI00184851	Type 2 lactosamine alpha-2,3-sialyltransferase	ST3GAL6	Q9Y274	3	1	2
IPI00027507	Complement factor H-related protein 3 precursor	CFHR3	Q02985	3	5	4
IPI00025447	Elongation factor 1-alpha	EEF1A1		3		1
IPI00029168	Apolipoprotein	LPA	P08519	3	4	6
IPI00081836	Histone H2A type 1-H	HIST1H2AH	Q96KK5	3	2	3

Table S1.

IPI	Protein name	Gene symbol	Swiss Prot ID	Number of unique peptides identified Normal	CFS	nPTLS
IPI00397645	Isoform 2 of Matrix-remodeling-associated protein 7	MXRA7	P84157	3	5	3
IPI00299145	Keratin, type II cytoskeletal 6C	KRT6C	P48668	3	1	4
IPI00289862	Secernin-1	SCRN1	Q12765	2		
IPI00829827	Uncharacterized protein ENSP00000374804	-		2	2	2
IPI00219806	Protein S100-A7	S100A7	P31151	2	1	
IPI00005038	Ribonuclease UK114	HRSP12	P52758	2	2	2
IPI00873344	N8 protein long isoform (Fragment)	TPD52	P55327	2	1	1
IPI00013945	Isoform 1 of Uromodulin precursor	UMOD	P07911	2		
IPI00465261	Isoform 1 of Endoplasmic reticulum aminopeptidase 2	ERAP2	Q6P179	2	1	
IPI00552939	Isoform 1 of Complement C1q-like protein 3 precursor	C1QL3	Q5VWW1	2	5	4
IPI00827939	Anti-mucin1 light chain variable region (Fragment)	-		2	2	2
IPI00065312	N-acetyl-beta-glucosaminyl-glycoprotein 4-beta-N- acetylgalactosaminyltransferase 1	B4GALNT4	Q76KP1	2	1	1
IPI00152524	Isoform 3 of Neuropilin and tolloid-like protein 1 precursor	NETO1	Q8TDF5	2	1	
IPI00182194	Teneurin-2	ODZ2	Q9NT68	2	1	1
IPI00215997	CD9 antigen	CD9	P21926	2	2	2
IPI00009890	Glia-derived nexin precursor	SERPINE2	P07093	2	3	6
IPI00165936	Isoform A of Chloride intracellular channel 6	CLIC6	Q96NY7	2	1	1
IPI00783818	Immunglobulin heavy chain variable region (Fragment)	-		2	3	5
IPI00382476	Ig heavy chain V-III region WEA	-	P01763	2	3	4
IPI00242905	Uncharacterized protein ENSP00000344689 (Fragment)	-		2		
IPI00024307	Ephrin-B1 precursor	EFNB1	P98172	2	10	9
IPI00022890	Ig lambda chain V region 4A precursor	IGLV7-43	P04211	2		1
IPI00219468	Isoform IIa of Profilin-2	PFN2	P35080	2		1
IPI00294713	Isoform 1 of Mannan-binding lectin serine protease 2 precursor	MASP2	O00187	2		
IPI00026546	Platelet-activating factor acetylhydrolase IB subunit beta	PAFAH1B2	P68402	2	1	
IPI00554752	cAMP-dependent protein kinase type II-beta regulatory subunit	PRKAR2B	P31323	2		
IPI00413344	Cofilin-2	CFL2	Q9Y281	2		2
IPI00010402	Putative uncharacterized protein	SH3BGRL3		2	5	3
IPI00306844	Corticotropin-releasing factor-binding protein precursor	CRHBP	P24387	2	2	

Table S1.

IPI	Protein name	Gene symbol	Swiss Prot ID	Number of unique peptides identified<br>Normal	CFS	nPTLS
IPI00384016	Full-length cDNA 5-PRIME end of clone CS0DJ009YL13 of T cells (Jurkat cell line) of Homo sapiens (Fragment)	DLST		2	1	
IPI00477868	LAMA5 protein	LAMA5		2	3	2
IPI00022640	Neurogranin	NRGN	Q92686	2	1	1
IPI00644346	ADAMTS-like protein 2 precursor	ADAMTSL2	Q86TH1	2	4	4
IPI00027847	Lipoprotein lipase precursor	LPL	P06858	2	1	1
IPI00293460	ATP-binding cassette sub-family A member 1	ABCA1	O95477	2		
IPI00032227	Isoform 1 of Rabphilin-3A	RPH3A	Q9Y2J0	2	1	1
IPI00009867	Keratin, type II cytoskeletal 5	KRT5	P13647	2	1	4
IPI00064377	Tumor necrosis factor receptor superfamily member 19L precursor	RELT	Q969Z4	2	2	2
IPI00816775	F5-20 (Fragment)	-		2	2	2
IPI00002966	Heat shock 70 kDa protein 4	HSPA4	P34932	2		
IPI00298476	Isoform 1 of Gremlin-1 precursor	GREM1	O60565	2		
IPI00830035	Similar to Anti-streptococcal/anti-myosin immunoglobulin kappa light chain variable region	-		2	3	2
IPI00303161	Endothelial cell-selective adhesion molecule precursor	ESAM	Q96AP7	2	11	10
IPI00002320	Leucine-rich repeat transmembrane protein FLRT3 precursor	FLRT3	Q9NZU0	2	2	2
IPI00166613	Isoform 1 of Putative polypeptide N-acetylgalactosaminyltransferase-like protein 1	GALNTL1	Q8N428	2	5	7
IPI00001120	CDNA FLJ31810 fis, clone NT2RI2009289, weakly similar to CARBOXYPEPTIDASE N 83 KD CHAIN	LINGO2		2		1
IPI00382442	Ig lambda chain V-V region DEL	-	P01719	2	1	2
IPI00029756	Proto-oncogene tyrosine-protein kinase MER precursor	MERTK	Q12866	2	3	2
IPI00008202	Headcase protein homolog	HECA	Q9UBI9	2		
IPI00007853	Gamma-interferon-inducible lysosomal thiol reductase precursor	IFI30	P13284	2	7	5
IPI00220748	Isoform Alpha-7X1A of Integrin alpha-7 precursor	ITGA7	Q13683	2	1	2
IPI00465322	Uncharacterized protein BOC	BOC		2		1
IPI00024580	Methylcrotonoyl-CoA carboxylase subunit alpha, mitochondrial precursor	MCCC1	Q96RQ3	2	2	
IPI00328431	Isoform 1 of Netrin receptor UNC5B precursor	UNC5B	Q8IZJ1	2	3	4
IPI00829834	Ig kappa chain V-III region VH precursor	-	P04434	2	3	3

Table S1.

IPI	Protein name	Gene symbol	Swiss Prot ID	Number of unique peptides identified<br>Normal	CFS	nPTLS
IPI00008586	Isoform 1 of Chondroitin sulfate proteoglycan 5 precursor	CSPG5	O95196	2	3	4
IPI00006524	Uncharacterized protein KIAA0319 precursor	KIAA0319	Q5VV43	2	3	3
IPI00019771	Fractalkine precursor	CX3CL1	P78423	2	10	8
IPI00103755	Isoform 2 of Netrin receptor UNC5D precursor	UNC5D	Q6UXZ4	2	2	4
IPI00550533	Isoform 1 of Uncharacterized protein C1orf56 precursor	C1orf56	Q9BUN1	2	1	2
IPI00306576	Arylsulfatase B precursor	ARSB	P15848	2		
IPI00854624	Uncharacterized protein ENSP00000375043	-		2	2	3
IPI00003391	Teneurin-1	ODZ1	Q9UKZ4	2		
IPI00002283	Isoform 2 of Patched domain-containing protein 2	PTCHD2	Q9P2K9	2	1	1
IPI00178727	Novel protein	C10orf79		2		
IPI00383680	Ribophorin II	RPN2		2	1	3
IPI00026800	Scrapie-responsive protein 1 precursor	SCRG1	O75711	2	3	4
IPI00216461	Acylphosphatase-2	ACYP2	P14621	2	5	4
IPI00000871	Prolactin	PRL		2	2	2
IPI00419565	Isoform 1 of Stabilin-1 precursor	STAB1	Q9NY15	2	4	2
IPI00029623	Proteasome subunit alpha type-6	PSMA6	P60900	2		
IPI00171199	Isoform 2 of Proteasome subunit alpha type-3	PSMA3	P25788	2		
IPI00149097	Semaphorin-4A precursor	SEMA4A	Q9H3S1	2	1	1
IPI00021834	Isoform Alpha of Tissue factor pathway inhibitor precursor	TFPI	P10646	2		1
IPI00001610	Insulin-like growth factor IA precursor	IGF1	P01343	2	3	2
IPI00001592	Isoform 2 of Transmembrane glycoprotein NMB precursor	GPNMB	Q14956	2	6	4
IPI00382682	Putative matrix cell adhesion molecule-3	-		2	4	2
IPI00181079	Meteorin-like protein precursor	METRNL	Q641Q3	2	1	4
IPI00061354	Isoform 2 of Bromodomain adjacent to zinc finger domain protein 2B	BAZ2B	Q9UIF8	2	1	
IPI00020884	Plexin-A3 precursor	PLXNA3	P51805	2		
IPI00009054	Isoform BMP1-3 of Bone morphogenetic protein 1 precursor	BMP1	P13497	2	3	3
IPI00008148	Isoform 1 of GDNF family receptor alpha-1 precursor	GFRA1	P56159	2	1	1
IPI00018246	Isoform 1 of Hexokinase-1	HK1	P19367	2	2	1
IPI00827839	VK3 protein (Fragment)	-		2	2	2
IPI00640292	Isoform 1 of Protein G7c precursor	C6orf27	Q9Y334	2	2	3
IPI00396930	Uncharacterized protein ENSP00000353216 (Fragment)	-		2	1	2

Table S1.

Number of unique peptides identified

IPI	Protein name	Gene symbol	Swiss Prot ID	Normal	CFS	nPTLS
IPI00297779	T-complex protein 1 subunit beta	CCT2	P78371	2	1	
IPI00168920	collagen, type XXIV, alpha 1	COL24A1		2	1	
IPI00829810	Uncharacterized protein ENSP00000375027	-		2	3	3
IPI00001872	Isoform 1 of Protocadherin gamma C3 precursor	PCDHGC3	Q9UN70	2		
IPI00175989	Rho family guanine-nucleotide exchange factor	MCF2L2		2		
IPI00432766	Isoform 2 of Netrin-G2 precursor	NTNG2	Q96CW9	2	1	1
IPI00300623	Pro-MCH precursor	PMCH	P20382	2		
IPI00215980	Isoform Alpha of Poliovirus receptor-related protein 2 precursor	PVRL2	Q92692	2	3	2
IPI00011865	Isoform 2 of Platelet-derived growth factor D precursor	PDGFD	Q9GZP0	2		
IPI00735451	Uncharacterized protein ENSP00000375035	-		2	7	6
IPI00015479	UPF0454 protein C12orf49 precursor	C12orf49	Q9H741	2	11	7
IPI00294619	Protein TFG	TFG	Q92734	2		
IPI00100154	Toll-interacting protein	TOLLIP	Q9H0E2	2		
IPI00003441	Isoform 1 of Protein C1orf9 precursor	C1orf9	Q9UBS9	2	1	
IPI00043810	Isoform 1 of Proline-rich transmembrane protein 1	PRRT1	Q99946	2	2	1
IPI00022389	Isoform 1 of C-reactive protein precursor	CRP	P02741	2	3	3
IPI00021997	Protein CREG1 precursor	CREG1	O75629	2		1
IPI00418169	annexin A2 isoform 1	ANXA2		2	3	4
IPI00024976	Mitochondrial import receptor subunit TOM22 homolog	TOMM22	Q9NS69	2		
IPI00012315	Nucleoside diphosphate kinase 3	NME3	Q13232	2		
IPI00550949	Bone morphogenetic protein 7 precursor	BMP7	P18075	2		1
IPI00006034	Cysteine-rich protein 2	CRIP2	P52943	2	1	1
IPI00297180	Cadherin-9 precursor	CDH9	Q9ULB4	2	3	2
IPI00003527	Ezrin-radixin-moesin-binding phosphoprotein 50	SLC9A3R1	O14745	2		1
IPI00552943	V1-11 protein	IGLV1-36		2	1	
IPI00008726	Iron-responsive element-binding protein 2	IREB2	P48200	2	2	1
IPI00061520	hypothetical protein LOC84752	MGC4655		2	1	1
IPI00021727	C4b-binding protein alpha chain precursor	C4BPA	P04003	2	10	12
IPI00219420	Structural maintenance of chromosomes protein 3	SMC3	Q9UQE7	2	2	1
IPI00005774	Isoform 1 of Low-density lipoprotein receptor-related protein 8 precursor	LRP8	Q14114	2	2	2
IPI00411706	S-formylglutathione hydrolase	ESD	P10768	2	5	6

Table S1.

Number of unique peptides identified

IPI	Protein name	Gene symbol	Swiss Prot ID	Normal	CFS	nPTLS
IPI00879950	15 kDa protein	-		2		
IPI00027972	Isoform 1 of Leukocyte immunoglobulin-like receptor subfamily A member 2 precursor	LILRA2	Q8N149	2	4	2
IPI00004494	Semaphorin-3E precursor	SEMA3E	O15041	2	2	1
IPI00383951	Isoform 3 of Protein sidekick-1 precursor	SDK1	Q7Z5N4	2	2	1
IPI00099650	Protein jagged-1 precursor	JAG1	P78504	2	3	2
IPI00008756	Isoform 1 of Bullous pemphigoid antigen 1, isoforms 1/2/3/4/5/8 (Fragment)	DST	Q03001	2		
IPI00008164	Prolyl endopeptidase	PREP	P48147	2	2	3
IPI00002511	Cyclic AMP-dependent transcription factor ATF-6 alpha	ATF6	P18850	2		
IPI00396383	Isoform 1 of von Willebrand factor A domain-containing protein 1 precursor	VWA1	Q6PCB0	2	2	5
IPI00009901	Nuclear transport factor 2	NUTF2	P61970	2	7	7
IPI00009477	Intercellular adhesion molecule 2 precursor	ICAM2	P13598	2	4	3
IPI00454695	Histone H2B type 2-C	HIST2H2BC	Q6DN03	2		
IPI00031718	Isoform 1 of Ectonucleoside triphosphate diphosphohydrolase 4	ENTPD4	Q9Y227	2	1	
IPI00021119	Carbohydrate sulfotransferase 1	CHST1	O43916	2	1	1
IPI00012792	Cadherin-5 precursor	CDH5	P33151	2	1	2
IPI00004488	Vacuolar proton pump subunit F	ATP6V1F	Q16864	2		
IPI00075013	Complement C1q tumor necrosis factor-related protein 1 precursor	C1QTNF1	Q9BXJ1	2		1
IPI00514517	V4-1 protein	IGLV5-37		2	1	1
IPI00412492	Isoform 1 of Plexin-D1 precursor	PLXND1	Q9Y4D7	2	1	1
IPI00178352	Isoform 1 of Filamin-C	FLNC	Q14315	2	2	3
IPI00009439	Synaptotagmin-1	SYT1	P21579	2		
IPI00006009	Isoform 2 of Pleckstrin homology domain-containing family B member 1	PLEKHB1	Q9UF11	2	4	4
IPI00413587	Isoform 1 of BH3-interacting domain death agonist	BID	P55957	2	2	3
IPI00008504	Carbonic anhydrase 14 precursor	CA14	Q9ULX7	2		1
IPI00789259	V1-13 protein (Fragment)	IGLV1-40		2	1	1
IPI00017557	Secreted frizzled-related protein 4 precursor	SFRP4	Q6FHJ7	2	3	3
IPI00014223	Netrin-G1 ligand precursor	LRRC4C	Q9HCJ2	2	2	3
IPI00017334	Prohibitin	PHB	P35232	2		1
IPI00419442	IGLV6-57 protein	IGLV6-57		2	4	1
IPI00216699	Isoform 2 of Unc-112-related protein 2	FERMT3	Q86UX7	2		
IPI00004573	Polymeric immunoglobulin receptor precursor	PIGR	P01833	2	1	1

Table S1.

IPI	Protein name	Gene symbol	Swiss Prot ID	Number of unique peptides identified<br>Normal	CFS	nPTLS
IPI00044707	Hyaluronan and proteoglycan link protein 4 precursor	HAPLN4	Q86UW8	2	2	2
IPI00216348	Isoform 2C of Cytoplasmic dynein 1 intermediate chain 2	DYNC1I2	Q13409	2		
IPI00044600	VPS10 domain-containing receptor SorCS2 precursor	SORCS2	Q96PQ0	2	2	3
IPI00257882	Xaa-Pro dipeptidase	PEPD	P12955	2	4	4
IPI00176104	Isoform 1 of SLIT and NTRK-like protein 2 precursor	SLITRK2	Q9H156	2	2	1
IPI00012007	Adenosylhomocysteinase	AHCY	P23526	2		1
IPI00328826	Uncharacterized protein CADPS2	CADPS2		2		
IPI00151036	RING finger protein 13	RNF13	O43567	2	2	1
IPI00329332	Syntaxin-12	STX12	Q86Y82	2	3	4
IPI00020407	Alpha-1,6-mannosylglycoprotein 6-beta-N-acetylglucosaminyltransferase A	MGAT5	Q09328	2	3	
IPI00289802	Isoform 1 of CUB and sushi domain-containing protein 2	CSMD2	Q7Z408	2	2	2
IPI00441498	Folate receptor alpha precursor	FOLR1	P15328	2	3	5
IPI00004457	Membrane copper amine oxidase	AOC3	Q16853	2	2	2
IPI00328587	Enolase	ENO1P		2	2	1
IPI00387105	Ig kappa chain V-I region Mev	-	P01612	2	4	3
IPI00382493	Ig heavy chain V-III region WAS	-	P01776	2	1	2
IPI00384400	Myosin-reactive immunoglobulin heavy chain variable region (Fragment)	-		2	1	1
IPI00396961	Leucine-rich repeat and fibronectin type-III domain-containing protein 5 precursor	LRFN5	Q96NI6	2		
IPI00337612	Discoidin, CUB and LCCL domain-containing protein 1 precursor	DCBLD1	Q8N8Z6	2	1	1
IPI00024689	Aquaporin-1	AQP1	P29972	2		
IPI00741608	similar to eukaryotic translation initiation factor 5A	EIF5AL3		2		1
IPI00414294	hypothetical protein	LOC729956		2	2	3
IPI00024466	UDP-glucose ceramide glucosyltransferase-like 1 isoform 1	UGCGL1		2	1	1
IPI00019907	Glypican-3 precursor	GPC3	P51654	2	1	2
IPI00328680	Multiple coagulation factor deficiency protein 2 precursor	MCFD2	Q8NI22	2	6	5
IPI00012009	Isoform 1 of Granulocyte-macrophage colony-stimulating factor receptor alpha chain precursor	CSF2RA	P15509	2	4	5
IPI00026031	Uncharacterized protein C6orf72 precursor	C6orf72	Q9NU53	2		
IPI00219067	Glutathione S-transferase Mu 2	GSTM2	P28161	2		2
IPI00217963	Keratin, type I cytoskeletal 16	KRT16	P08779	2	3	2

Table S1. Number of unique peptides identified

IPI	Protein name	Gene symbol	Swiss Prot ID	Normal	CFS	nPTLS
IPI00382488	Ig heavy chain V-III region HIL	-	P01771	2	2	2
IPI00022445	Platelet basic protein precursor	PPBP	P02775	2	3	1
IPI00410079	Isoform 1 of Protein FAM82C	FAM82C	Q96TC7	2	2	1
IPI00827929	VH-3 family (VH26)D/J protein (Fragment)	-		2	4	4
IPI00019812	Serine/threonine-protein phosphatase 5	PPP5C	P53041	2		3
IPI00010343	Sodium/calcium exchanger 2 precursor	SLC8A2	Q9UPR5	2	1	1
IPI00006510	Tubulin beta-1 chain	TUBB1	Q9H4B7	2		1
IPI00023942	Isoform 2 of Syndecan-3	SDC3	O75056	2		
IPI00000914	Isoform 1 of Calcitonin precursor	CALCA	P01258	2	3	4
IPI00293276	Macrophage migration inhibitory factor	MIF	P14174	2	2	2
IPI00305833	Smu-1 suppressor of mec-8 and unc-52 protein homolog	SMU1	Q2TAY7	2		
IPI00025812	Carbonic anhydrase-related protein 11 precursor	CA11	O75493	2	2	
IPI00550746	Nuclear migration protein nudC	NUDC	Q9Y266	2		
IPI00003470	Ig kappa chain V-I region Wes	-	P01611	2	2	2
IPI00299116	Podocalyxin-like protein 1 precursor	PODXL	O00592	2		
IPI00028387	Isoform 1 of Uncharacterized protein C20orf116 precursor	C20orf116	Q96HY6	2		1
IPI00009396	Isoform 1 of Cannabinoid receptor 1	CNR1	P21554	2		
IPI00033466	C-type lectin domain family 11 member A precursor	CLEC11A	Q9Y240	2	2	3
IPI00828156	NANUC-1 heavy chain (Fragment)	-		2	2	4
IPI00400935	Isoform 1 of Collagen alpha-1(XVI) chain precursor	COL16A1	Q07092	2	1	1
IPI00181743	Isoform 1 of BAI1-associated protein 3	BAIAP3	O94812	2		
IPI00291488	Isoform 1 of WAP four-disulfide core domain protein 2 precursor	WFDC2	Q14508	2	5	6
IPI00382478	Ig heavy chain V-III region TIL	-	P01765	2	4	2
IPI00426727	Isoform 1 of Methyl-CpG-binding domain protein 4	MBD4	O95243	2		
IPI00552937	NHL repeat containing 3 isoform a	NHLRC3		2	5	6
IPI00787936	similar to cathepsin L-like protein	LOC644021		2	1	2
IPI00031907	Isoform 1 of Transmembrane protein 108 precursor	TMEM108	Q6UXF1	2		
IPI00007047	Protein S100-A8	S100A8	P05109	2		
IPI00018879	Alpha-L-iduronidase precursor	IDUA	P35475	2		
IPI00399089	Mesoderm development candidate 2	MESDC2	Q14696	2	2	1
IPI00387097	Ig kappa chain V-I region Lay	-	P01605	2	3	4
IPI00007812	Vacuolar ATP synthase subunit B, brain isoform	ATP6V1B2	P21281	2		
IPI00170692	Vesicle-associated membrane protein-associated protein A	VAPA	Q9P0L0	2	2	3

Table S1.

Number of unique peptides identified

IPI	Protein name	Gene symbol	Swiss Prot ID	Normal	CFS	nPTLS
IPI00413641	Aldose reductase	AKR1B1	P15121	2	2	3
IPI00854707	Immunglobulin heavy chain variable region (Fragment)	-		2	7	8
IPI00788258	similar to lysyl oxidase-like 1 preproprotein	LOXL1		2		
IPI00410675	Syntaxin-1B	STX1B	P61266	2	1	2
IPI00783156	Bone morphogenetic protein receptor type-2 precursor	BMPR2	Q13873	2	4	3
IPI00045536	Isoform 3 of Chitinase domain-containing protein 1 precursor	CHID1	Q9BWS9	2	2	1
IPI00019180	Glypican-5 precursor	GPC5	P78333	2	1	1
IPI00176581	Isoform 1 of Fanconi anemia group M protein	FANCM	Q8IYD8	2	1	1
IPI00023191	Target of myb1	TOM1		2	1	
IPI00217005	Ankyrin repeat domain-containing protein 18A	ANKRD18A	Q8IVF6	2	1	
IPI00219682	Erythrocyte band 7 integral membrane protein	STOM	P27105	2		
IPI00008085	Zinc transporter ZIP10 precursor	SLC39A10	Q9ULF5	2	3	2
IPI00329538	Prostasin precursor	PRSS8	Q16651	2		2
IPI00030634	Isoform 1 of Gamma-glutamyltransferase 4 precursor	GGTL3	Q9UJ14	2	3	4
IPI00027444	Leukocyte elastase inhibitor	SERPINB1	P30740	2	2	3
IPI00550677	WSC domain-containing protein 1	WSCD1	Q658N2	2	2	4
IPI00029050	Isoform 1 of Glycosyltransferase-like protein LARGE1	LARGE	O95461	2		1
IPI00328520	Isoform 2 of Proline-rich transmembrane protein 2	PRRT2	Q7Z6L0	2	7	7
IPI00749514	attractin-like 1	ATRNL1		2		
IPI00220281	Guanine nucleotide-binding protein G(o) subunit alpha 1	GNAO1	P09471	2	1	
IPI00299083	Junctional adhesion molecule B precursor	JAM2	P57087	2	4	3
IPI00000087	Sodium channel subunit beta-2 precursor	SCN2B	O60939	2	2	4
IPI00010810	Electron transfer flavoprotein subunit alpha, mitochondrial precursor	ETFA	P13804	2	1	1
IPI00301812	Isoform 1 of SPARC-related modular calcium-binding protein 1 precursor	SMOC1	Q9H4F8	2	9	8
IPI00027239	Isoform Alpha of Tumor necrosis factor ligand superfamily member 13 precursor	TNFSF12-TNFSF13	O75888	2	2	1
IPI00029700	Isoform Long of Down syndrome cell adhesion molecule precursor	DSCAM	O60469	2		1

Table S1.

Number of unique peptides identified

IPI	Protein name	Gene symbol	Swiss Prot ID	Normal	CFS	nPTLS
IPI00847652	CDNA FLJ46805 fis, clone TRACH3033535	LOC400891		2	1	1
IPI00024919	Thioredoxin-dependent peroxide reductase, mitochondrial precursor	PRDX3	P30048	2	2	4
IPI00250724	Protein kinase-like domain containing protein	C18orf51	Q0P6D2	2	12	13
IPI00022039	Isoform 3 of SLAM family member 5 precursor	CD84	Q9UIB8	2	1	2
IPI00004047	Isoform 1 of Exostosin-2	EXT2	Q93063	2	3	
IPI00387106	Ig kappa chain V-I region Ni	-	P01613	2	2	2
IPI00024704	Uronyl 2-sulfotransferase	UST	Q9Y2C2	2		
IPI00305975	Spondin-2 precursor	SPON2	Q9BUD6	2	2	3
IPI00440493	ATP synthase subunit alpha, mitochondrial precursor	ATP5A1	P25705	2		1
IPI00289926	leukocyte immunoglobulin-like receptor, subfamily B, member 4 isoform 2	LILRB4		2	2	3
IPI00419237	Isoform 1 of Cytosol aminopeptidase	LAP3	P28838	2		
IPI00646291	Integral membrane protein GPR180 precursor	GPR180	Q86V85	2	1	3
IPI00021199	Stathmin-3	STMN3	Q9NZ72	2		2
IPI00024067	Isoform 1 of Clathrin heavy chain 1	CLTC	Q00610	2	2	
IPI00016014	Isoform 1 of Integral membrane protein 2C	ITM2C	Q9NQX7	2	2	2
IPI00020692	Isoform 1 of Sodium channel protein type 3 subunit alpha	SCN3A	Q9NY46	2	1	
IPI00021091	Isoform 1 of Leucine-rich glioma-inactivated protein 1 precursor	LGI1	O95970	2		1
IPI00021695	Isoform D of Plasma membrane calcium-transporting ATPase 1	ATP2B1	P20020	2		
IPI00413781	chemokine (C-X-C motif) ligand 12 (stromal cell-derived factor 1) isoform gamma	CXCL12		2	1	1
IPI00480159	inositol polyphosphate-5-phosphatase F	INPP5F		2		
IPI00005690	Matrilin-3 precursor	MATN3	O15232	2	1	
IPI00289746	Isoform 2 of Serine/threonine-protein kinase PAK 1	PAK1	Q13153	2	1	1
IPI00295767	Noelin-2 precursor	OLFM2	O95897	2	2	
IPI00016371	Isoform JM-A of Receptor tyrosine-protein kinase erbB-4 precursor	ERBB4	Q15303	2	1	3
IPI00296441	Adenosine deaminase	ADA	P00813	2		3
IPI00827906	Anti-mucin1 light chain variable region (Fragment)	-		2	3	3
IPI00218795	L-selectin precursor	SELL	P14151	2	4	5

Table S1.

IPI	Protein name	Gene symbol	Swiss Prot ID	Number of unique peptides identified: Normal	CFS	nPTLS
IPI00382482	Ig heavy chain V-III region CAM	-	P01768	2	2	2
IPI00292657	NADP-dependent leukotriene B4 12-hydroxydehydrogenase	LTB4DH	Q14914	2		1
IPI00216694	plastin 3	PLS3	P13797	2	2	
IPI00383887	Immunoblobulin heavy chain (Fragment)	-		2	4	6
IPI00299547	Neutrophil gelatinase-associated lipocalin precursor	LCN2	P80188	2		2
IPI00385985	Ig lambda chain V-III region LOI	-	P80748	2	2	2
IPI00384395	Myosin-reactive immunoglobulin heavy chain variable region	-		2	4	1
IPI00064652	vascular endothelial growth factor A isoform e precursor	VEGFA	P15692	2	5	3
IPI00024572	aspartate beta-hydroxylase isoform e	ASPH		2	4	7
IPI00301180	Isoform 2 of Solute carrier family 12 member 5	SLC12A5	Q9H2X9	2	2	2
IPI00170814	PTK7 protein tyrosine kinase 7 isoform b precursor	PTK7		2	2	2
IPI00023754	Protein kinase C-binding protein NELL1 precursor	NELL1	Q92832	2	3	4
IPI00384407	Myosin-reactive immunoglobulin heavy chain variable region (Fragment)	-		2	4	2
IPI00030431	Isoform 1 of Anthrax toxin receptor 1 precursor	ANTXR1	Q9H6X2	2	1	1
IPI00220706	Hemoglobin subunit gamma-1	HBG1	P69891	2	5	1
IPI00028193	192 kDa protein	KNDC1		2		2
IPI00000775	Isoform 1 of Leucine-rich repeats and immunoglobulin-like domains protein 1 precursor	LRIG1	Q96JA1	2		1
IPI00221117	Acylphosphatase-1	C17orf13	P07311	2	1	1
IPI00646689	Thioredoxin domain-containing protein 17	TXNDC17	Q9BRA2	2	1	
IPI00019904	Isoform 1 of Beta-adducin	ADD2	P35612	2		
IPI00218319	Isoform 2 of Tropomyosin alpha-3 chain	TPM3	P06753	2	1	2
IPI00160369	PRKCA-binding protein	PICK1	Q9NRD5	1		
IPI00002478	Isoform B of Endothelin-converting enzyme 1	ECE1	P42892	1		
IPI00027438	Flotillin-1	FLOT1	O75955	1		
IPI00033030	Protein ADRM1	ADRM1	Q16186	1	1	
IPI00642645	Methylenetetrahydrofolate reductase	MTHFR		1		
IPI00386131	Ig kappa chain V-III region IARC/BL41 precursor	-	P06311	1	1	3
IPI00017964	Small nuclear ribonucleoprotein Sm D3	SNRPD3	P62318	1		

Table S1.

Number of unique peptides identified

IPI	Protein name	Gene symbol	Swiss Prot ID	Normal	CFS	nPTLS
IPI00163724	Potassium/sodium hyperpolarization-activated cyclic nucleotide-gated channel 3	HCN3	Q9P1Z3	1	1	1
IPI00478640	Isoform 1 of Transmembrane protein C17orf87	C17orf87	Q6UWF3	1		
IPI00013290	hepatoma-derived growth factor-related protein 2 isoform 1	HDGF2		1		
IPI00383953	VH4 heavy chain variable region precursor (Fragment)	-		1	2	4
IPI00011051	T-cell leukemia homeobox protein 1	TLX1	P31314	1		
IPI00154858	Platelet endothelial aggregation receptor 1 precursor	PEAR1	Q5VY43	1		1
IPI00830051	Similar to Immunolgoobulin heavy chain	LOC90925		1		
IPI00550720	Isoform 1 of Uncharacterized protein C19orf57	C19orf57	Q0VDD7	1		
IPI00103510	Relaxin receptor 2	RXFP2	Q8WXD0	1		
IPI00373872	polycystin 1-like 2 isoform a	PKD1L2		1		
IPI00295172	Ninjurin-1	NINJ1	Q92982	1		1
IPI00328522	KTEL motif-containing protein 1	KTELC1		1		
IPI00000811	Proteasome subunit beta type-6 precursor	PSMB6	P28072	1		
IPI00251596	Isoform 1 of Collagen alpha-1(XXIII) chain	COL23A1	Q86Y22	1		
IPI00550906	Cleavage stimulation factor 64 kDa subunit, tau variant	CSTF2T	Q9H0L4	1		
IPI00550263	Isoform 5 of Serine/threonine-protein kinase MRCK alpha	CDC42BPA	Q5VT25	1		1
IPI00045219	Sorting nexin-18	SNAG1	Q96RF0	1		
IPI00179473	Isoform 1 of Sequestosome-1	SQSTM1	Q13501	1	4	4
IPI00221235	nucleoporin 160kDa	NUP160	Q12769	1		
IPI00167560	PAP-associated domain-containing protein 4	PAPD4		1		
IPI00005719	Isoform 1 of Ras-related protein Rab-1A	RAB1A	P62820	1		
IPI00013933	Isoform DPI of Desmoplakin	DSP	P15924	1		
IPI00098902	2-oxoglutarate dehydrogenase E1 component, mitochondrial precursor	OGDH	Q02218	1		
IPI00000959	Isoform 1 of VIP peptides precursor	VIP	P01282	1	1	1
IPI00289271	Liprin-alpha-2	PPFIA2	O75334	1		
IPI00216470	Isoform 1 of Phosphatidylinositol-5-phosphate 4-kinase type-2 beta	PIP4K2B	P78356	1		
IPI00879575	71 kDa protein	-		1	2	
IPI00744825	Conserved hypothetical protein	-		1		
IPI00217264	Isoform 3 of MAP7 domain-containing protein 3	MAP7D3	Q8IWC1	1		1

Table S1.

Number of unique peptides identified

IPI	Protein name	Gene symbol	Swiss Prot ID	Normal	CFS	nPTLS
IPI00005675	NF-kappa-B-repressing factor	NKRF	O15226	1		
IPI00218628	Isoform 2 of Integrin alpha-IIb precursor	ITGA2B	P08514	1		
IPI00217989	Isoform 1 of Protein-associating with the carboxyl-terminal domain of ezrin	SCYL3	Q8IZE3	1		
IPI00217405	Isoform 1 of E3 ubiquitin-protein ligase UBR1	UBR1	Q8IWV7	1		
IPI00829836	Uncharacterized protein ENSP00000374797	-		1	2	1
IPI00185661	Ubiquitin carboxyl-terminal hydrolase 32	USP32	Q8NFA0	1	1	
IPI00554521	Ferritin heavy chain	FTH1	P02794	1	1	1
IPI00217258	CCDC100 protein	CCDC100		1		
IPI00307729	ADAMTS-3 precursor	ADAMTS3	O15072	1	2	3
IPI00604430	Isoform 2 of Receptor expression-enhancing protein 2	REEP2	Q9BRK0	1		
IPI00456670	Isoform 13 of Peroxisomal N(1)-acetyl-spermine/spermidine oxidase	PAOX	Q6QHF9	1		
IPI00186621	Orofacial clefting chromosomal breakpoint region 1	OFCC1		1		
IPI00000160	Proopiomelanocortin preproprotein	POMC	P01189	1	2	1
IPI00002535	FK506-binding protein 2 precursor	FKBP2	P26885	1	2	2
IPI00295098	Signal recognition particle receptor subunit beta	SRPRB	Q9Y5M8	1		
IPI00071824	Isoform 1 of Cytoskeleton-associated protein 2	CKAP2	Q8WWK9	1		
IPI00747494	Glutamate receptor delta-2 subunit precursor	GRID2	O43424	1		
IPI00302962	Amphiphysin I variant CT4 (Fragment)	AMPH		1	2	1
IPI00879409	28 kDa protein	-		1		
IPI00005153	Isoform Aa of Odorant-binding protein 2a precursor	OBP2A	Q9NY56	1	3	1
IPI00169259	Small VCP/p97-interacting protein	SVIP	Q8NHG7	1		
IPI00106506	Isoform 1 of Evolutionarily conserved signaling intermediate in Toll pathway, mitochondrial precursor	ECSIT	Q9BQ95	1		
IPI00006900	Something about silencing protein 10	UTP3	Q9NQZ2	1		
IPI00472332	similar to polyhomeotic 1-like isoform 4	LOC653441		1		
IPI00789245	Isoform 2 of Probable organic cation transporter protein C6orf85	SLC22A23	A1A5C7	1		
IPI00291922	Proteasome subunit alpha type-5	PSMA5	P28066	1		
IPI00012044	Isoform 1 of Pro-neuregulin-3, membrane-bound isoform precursor	NRG3	P56975	1	2	2
IPI00290744	Fibronectin type-III domain-containing protein C5orf40	C5orf40	Q8TBE3	1	1	1
IPI00152145	Protein odd-skipped-related 1	OSR1	Q8TAX0	1		

Table S1.

IPI	Protein name	Gene symbol	Swiss Prot ID	Number of unique peptides identified<br>Normal	CFS	nPTLS
IPI00021770	Isoform 1 of 3-hydroxy-3-methylglutaryl-coenzyme A reductase	HMGCR	P04035	1		
IPI00465045	DIP2 disco-interacting protein 2 homolog B	DIP2B		1		
IPI00031131	Adipocyte plasma membrane-associated protein	C20orf3	Q9HDC9	1	1	2
IPI00018803	homeobox D12	HOXD12		1		
IPI00828083	Heavy chain Fab (Fragment)	-		1	1	1
IPI00827580	Immunogobulin kappa, VJ region (Fragment)	-		1	2	1
IPI00021594	Isoform 1 of Glycosylphosphatidylinositol anchor attachment 1 protein	GPAA1	O43292	1		
IPI00010360	Isoform 1 of Collagen alpha-3(IV) chain precursor	COL4A3	Q01955	1		
IPI00028381	Isoform 1 of Delta-like protein 2 precursor	DLK2	Q6UY11	1	2	2
IPI00017163	Isoform 1 of E3 ubiquitin-protein ligase HECW2	HECW2	Q9P2P5	1		
IPI00465044	Protein RCC2	RCC2	Q9P258	1		
IPI00013847	Cytochrome b-c1 complex subunit 1, mitochondrial precursor	UQCRC1	P31930	1		
IPI00024248	Sodium/iodide cotransporter	SLC5A5	Q92911	1		1
IPI00026665	Glutaminyl-tRNA synthetase	QARS	P47897	1		
IPI00180386	Isoform GN-1L of Glycogenin-1	GYG1	P46976	1		
IPI00001793	Beta-1,3-N-acetylglucosaminyltransferase radical fringe	RFNG	Q9Y644	1	5	5
IPI00167638	Isoform 1 of GTP-binding protein 10	GTPBP10	A4D1E9	1		
IPI00004367	FXYD domain-containing ion transport regulator 6 precursor	FXYD6	Q9H0Q3	1	1	3
IPI00003406	Isoform 1 of Drebrin	DBN1	Q16643	1		
IPI00827891	Cold agglutinin FS-2 H-chain (Fragment)	-		1	2	4
IPI00218292	Isoform Short of Ubiquitin fusion degradation protein 1 homolog	UFD1L	Q92890	1	1	
IPI00384861	Isoform 1 of ARF GTPase-activating protein GIT1	GIT1	Q9Y2X7	1		
IPI00018980	Sodium channel subunit beta-1 precursor	SCN1B	Q07699	1	1	1
IPI00020008	NEDD8 precursor	NEDD8	Q15843	1		1
IPI00028714	Matrix Gla protein precursor	MGP	P08493	1	2	2
IPI00387110	Ig kappa chain V-II region MIL	-	P01616	1	3	1
IPI00830044	Uncharacterized protein ENSP00000374806	-		1	1	1

Table S1.

IPI	Protein name	Gene symbol	Swiss Prot ID	Number of unique peptides identified<br>Normal	CFS	nPTLS
IPI00024012	Frizzled-7 precursor	FZD7	O75084	1	2	3
IPI00478860	Glycoprotein endo-alpha-1,2-mannosidase	MANEA	Q5SRI9	1		1
IPI00552735	V2-8 protein	IGLV3-12		1	1	1
IPI00000104	Isoform 1 of mRNA-capping enzyme	RNGTT	O60942	1	1	
IPI00296219	Glutaminase liver isoform, mitochondrial precursor	GLS2	Q9UI32	1		
IPI00005564	Stanniocalcin-1 precursor	STC1	P52823	1		1
IPI00376087	putative binding protein 7a5	7A5		1		
IPI00387113	Ig kappa chain V-III region B6	-	P01619	1	3	3
IPI00014850	Astrocytic phosphoprotein PEA-15	PEA15	Q15121	1		1
IPI00794679	Major histocompatibility complex, class I, B	MICA		1		
IPI00008282	Isoform 1 of Calcium/calmodulin-dependent 3',5'-cyclic nucleotide phosphodiesterase 1A	PDE1A	P54750	1		
IPI00007277	Isoform 1 of Leucine-rich repeat flightless-interacting protein 2	LRRFIP2	Q9Y608	1		
IPI00294519	Isoform 1 of Telomerase protein component 1	TEP1	Q99973	1		
IPI00179589	Myotrophin	MTPN	P58546	1		
IPI00744811	Low-density lipoprotein receptor-related protein 5 precursor	LRP5	O75197	1		
IPI00031485	Mitochondrial ribosomal protein 63	MRP63	Q9BQC6	1		
IPI00784044	Isoform 1 of Methylcrotonoyl-CoA carboxylase beta chain, mitochondrial precursor	MCCC2	Q9HCC0	1		1
IPI00514893	Disheveled-associated activator of morphogenesis 2	DAAM2	Q86T65	1	1	
IPI00429191	Eukaryotic peptide chain release factor subunit 1	ETF1	P62495	1		
IPI00514594	Isoform 1 of Protein FAM5B precursor	FAM5B	Q9C0B6	1		
IPI00853376	additional sex combs like 3	ASXL3		1		
IPI00216914	Vitelline membrane outer layer protein 1 homolog precursor	VMO1	Q7Z5L0	1		
IPI00400967	KIAA1843 protein (Fragment)	KIAA1843		1		1
IPI00166010	Isoform 1 of CCR4-NOT transcription complex subunit 1	CNOT1	A5YKK6	1		
IPI00793576	7 kDa protein	PRH2		1		1
IPI00301631	Isoform 1 of Torsin-3A precursor	TOR3A	Q9H497	1	1	1
IPI00399252	Isoform 1 of Protein Jade-1	PHF17	Q6IE81	1		
IPI00396077	Isoform 1 of E3 ubiquitin-protein ligase Topors	TOPORS	Q9NS56	1		1

Table S1.

IPI	Protein name	Gene symbol	Swiss Prot ID	Number of unique peptides identified Normal	CFS	nPTLS
IPI00144243	Human immunodeficiency virus type I enhancer-binding protein 2	HIVEP2	P31629	1		
IPI00186004	hypothetical protein LOC57730	KIAA1641		1		1
IPI00465178	Isoform 1 of Vacuolar proton translocating ATPase 116 kDa subunit a isoform 1	ATP6V0A1	Q93050	1		
IPI00291463	Radical S-adenosyl methionine domain-containing protein 2	RSAD2	Q8WXG1	1		
IPI00007040	Zinc finger protein 222	ZNF222	Q9UK12	1		
IPI00220578	Guanine nucleotide-binding protein G	GNAI3	P08754	1		
IPI00740191	similar to Forkhead box protein L1	LOC651986		1		
IPI00044607	Protein phosphatase inhibitor 2-like protein 1	PPP1R2P1	Q96PQ5	1		
IPI00301923	Isoform 1 of Cell division protein kinase 9	CDK9	P50750	1		
IPI00000070	Low-density lipoprotein receptor precursor	LDLR	P01130	1	2	1
IPI00255145	hypothetical protein	LOC342346		1		
IPI00552874	V1-3 protein	IGLV2-11		1	2	4
IPI00010737	Thrombomodulin precursor	THBD	P07204	1		
IPI00059395	Kinesin-like protein KIFC2	KIFC2	Q96AC6	1		1
IPI00030319	Forkhead box protein F2	FOXF2	Q12947	1		
IPI00168806	Isoform 1 of Myeloid/lymphoid or mixed-lineage leukemia protein 3 homolog	MLL3	Q8NEZ4	1		
IPI00018275	Prion-like protein doppel precursor	PRND	Q9UKY0	1		
IPI00025363	Isoform 1 of Glial fibrillary acidic protein	GFAP	P14136	1	1	
IPI00015285	Ethanolamine-phosphate cytidylyltransferase	PCYT2	Q99447	1		
IPI00302850	Small nuclear ribonucleoprotein Sm D1	SNRPD1	P62314	1		
IPI00554799	shadow of prion protein	SPRN		1	1	1
IPI00478124	61 kDa protein	UCKL1		1		
IPI00013681	N-terminally extended type 3 canonical transient receptor potential channel	TRPC3		1		
IPI00019208	Similar to 60S ribosomal protein L29	-		1		
IPI00304596	Non-POU domain-containing octamer-binding protein	NONO	Q15233	1		1
IPI00012895	Isoform 1 of Carbonic anhydrase 12 precursor	CA12	O43570	1		
IPI00299627	Dual oxidase 2 precursor	DUOX2	Q9NRD8	1		1
IPI00023513	Isoform 1 of E3 ubiquitin-protein ligase CHFR	CHFR	Q96EP1	1		
IPI00394870	Brorin precursor	VWC2	Q2TAL6	1	5	4
IPI00063827	Isoform 1 of Abhydrolase domain-containing protein 14B	ABHD14B	Q96IU4	1		2

Table S1.

Number of unique peptides identified

IPI	Protein name	Gene symbol	Swiss Prot ID	Normal	CFS	nPTLS
IPI00025019	Proteasome subunit beta type-1 precursor	PSMB1	P20618	1		
IPI00166817	Zinc finger protein 561	ZNF561	Q8N587	1		
IPI00375174	Ankyrin repeat and sterile alpha motif domain-containing protein 1B	ANKS1B		1		
IPI00296866	interphotoreceptor matrix proteoglycan 2	IMPG2		1		
IPI00040730	protocadherin 21 precursor	PCDH21		1		
IPI00013508	Alpha-actinin-1	ACTN1	P12814	1		
IPI00829980	Myosin-reactive immunoglobulin light chain variable region (Fragment)	-		1	4	4
IPI00028561	Kinesin heavy chain isoform 5C	KIF5C	O60282	1	1	2
IPI00743898	Uncharacterized protein ENSP00000357890 (Fragment)	-		1	1	
IPI00470766	Isoform 1 of Olfactomedin-like protein 2B precursor	OLFML2B	Q68BL8	1	1	1
IPI00644231	Isoform 1 of Cytoplasmic FMR1-interacting protein 1	CYFIP1	Q7L576	1		
IPI00007193	Isoform 2 of Ankyrin repeat domain-containing protein 26	ANKRD26	Q9UPS8	1		1
IPI00306959	Keratin, type II cytoskeletal 7	KRT7	P08729	1	1	2
IPI00816737	Rheumatoid factor D5 heavy chain (Fragment)	-		1	1	1
IPI00013466	Arsenical pump-driving ATPase	ASNA1	O43681	1		
IPI00016605	Uncharacterized protein C1orf123	C1orf123	Q9NWV4	1		
IPI00872861	PTD016 protein	LOC51136		1		
IPI00298285	Isoform 1 of Receptor tyrosine-protein kinase erbB-3 precursor	ERBB3	P21860	1	2	2
IPI00166619	Isoform 2 of Putative transporter SVOPL	SVOPL	Q8N434	1		
IPI00023359	Isoform 1 of Malonyl CoA-acyl carrier protein transacylase, mitochondrial precursor	MCAT	Q8IVS2	1	1	
IPI00029722	Kinesin heavy chain isoform 5A	KIF5A	Q12840	1		1
IPI00031696	FAST kinase domain-containing protein 3	FASTKD3	Q14CZ7	1		1
IPI00005128	Isoform 1 of Angiopoietin-2 precursor	ANGPT2	O15123	1	1	2
IPI00796647	HIG1 domain family, member 1C	HIGD1C		1		
IPI00852758	Similar to Ankyrin repeat domain-containing protein 26. Isoform 2	ANKRD18B		1		
IPI00023087	Ubiquitin-conjugating enzyme E2 T	UBE2T	Q9NPD8	1		
IPI00297444	Isoform 1 of CD177 antigen precursor	CD177	Q8N6Q3	1	3	6
IPI00161119	Isoform 1 of NF-kappa-B inhibitor beta	NFKBIB	Q15653	1		
IPI00016577	CDNA: FLJ22814 fis, clone KAIA3004	CD22		1		
IPI00852633	16 kDa protein	RABL2B		1		

Table S1.

Number of unique peptides identified

IPI	Protein name	Gene symbol	Swiss Prot ID	Normal	CFS	nPTLS
IPI00163391	Isoform 1 of Putative methyltransferase METT10D	METT10D	Q86W50	1		
IPI00299679	Isoform B of Ral guanine nucleotide dissociation stimulator-like 1	RGL1	Q9NZL6	1		
IPI00797699	20 kDa protein	-		1		
IPI00333126	Leucine-rich repeat-containing protein 56	LRRC56	Q8IYG6	1	1	1
IPI00794119	13 kDa protein	ABCC8		1		
IPI00816274	Chemokine-like factor superfamily 1 transcript variant 26	CMTM1		1		
IPI00038378	Isoform 1 of Enolase-phosphatase E1	ENOPH1	Q9UHY7	1		1
IPI00032830	Isoform 1 of Oligoribonuclease, mitochondrial precursor (Fragment)	REXO2	Q9Y3B8	1	1	1
IPI00171737	Isoform 2 of Leucine-rich repeat and death domain-containing protein	LRDD	Q9HB75	1		
IPI00385003	Putative transposase	TIGD1		1		
IPI00029123	Isoform A of Endothelin B receptor precursor	EDNRB	P24530	1		
IPI00218487	Gap junction alpha-1 protein	GJA1	P17302	1	1	1
IPI00158992	snRNA-activating protein complex subunit 4	SNAPC4	Q5SXM2	1		
IPI00029819	Neurogenic locus notch homolog protein 3 precursor	NOTCH3	Q9UM47	1	6	4
IPI00394712	Granulocyte inhibitory protein	-		1	2	
IPI00003933	hydroxyacyl glutathione hydrolase isoform 1	HAGH		1	3	3
IPI00184997	cDNA FLJ78771, highly similar to Homo sapiens discs, large homolog 7 (Drosophila), mRNA	DLG7		1		
IPI00001869	Pappalysin-1 precursor	PAPPA	Q13219	1		
IPI00027834	heterogeneous nuclear ribonucleoprotein L isoform a	HNRNPL	P14866	1		
IPI00062730	Uncharacterized protein C16orf45	C16orf45	Q96MC5	1		
IPI00004409	Discoidin domain-containing receptor 2 precursor	DDR2	Q16832	1	1	1
IPI00298258	UNC13B protein	UNC13B		1		
IPI00018352	Ubiquitin carboxyl-terminal hydrolase isozyme L1	UCHL1	P09936	1	2	1
IPI00744366	Conserved hypothetical protein	-		1		
IPI00556643	Semaphorin 3F variant	SEMA3F		1	2	5
IPI00000792	Quinone oxidoreductase	CRYZ	Q08257	1		
IPI00008905	UDP-glucuronosyltransferase 2B15 precursor	UGT2B15	P54855	1		

Table S1.

IPI	Protein name	Gene symbol	Swiss Prot ID	Number of unique peptides identified<br>Normal	CFS	nPTLS
IPI00031411	Cadherin-related tumor suppressor homolog precursor	FAT	Q14517	1		3
IPI00377077	Isoform 3 of Astrotactin-2 precursor	ASTN2	O75129	1		1
IPI00009294	Cysteine-rich motor neuron 1 protein precursor	CRIM1	Q9NZV1	1	1	2
IPI00186581	amplified in osteosarcoma isoform 2 precursor	OS9		1		
IPI00445364	CDNA FLJ44171 fis, clone THYMU2036058	-		1		
IPI00021985	transmembrane 9 superfamily protein member 4	TM9SF4	Q92544	1		1
IPI00166071	B-cell CLL/lymphoma 6 member B protein	BCL6B	Q8N143	1		
IPI00217023	MMAA protein	MMAA		1		1
IPI00218130	Glycogen phosphorylase, muscle form	PYGM	P11217	1		
IPI00455521	similar to transmembrane protein 46	C22:CTA-250D10.9		1	2	2
IPI00034006	Tyrosine-protein phosphatase non-receptor type 23	PTPN23	Q9H3S7	1		
IPI00021274	Ephrin type-A receptor 8 precursor	EPHA8	P29322	1	1	
IPI00394820	Olfactomedin-like protein 1 precursor	OLFML1	Q6UWY5	1	1	1
IPI00419253	Isoform 1 of Nck-associated protein 5	NAP5	O14513	1		
IPI00292393	Sodium channel protein type 4 subunit alpha	SCN4A	P35499	1		
IPI00029012	Eukaryotic translation initiation factor 3 subunit A	EIF3A	Q14152	1		
IPI00043201	Centromere protein J	CENPJ	Q9HC77	1		
IPI00385042	Nucleolar GTP-binding protein 1	GTPBP4	Q9BZE4	1		
IPI00410013	Isoform 1 of Zinc finger CCCH domain-containing protein 3	ZC3H3	Q8IXZ2	1	1	
IPI00402144	Isoform 1 of Zinc finger protein 555	ZNF555	Q8NEP9	1		
IPI00478986	Similar to 40S ribosomal protein S4	LOC126235		1		
IPI00478521	Isoform 1 of UPF0475 protein	RILPL1	Q5EBL4	1		
IPI00221332	Uncharacterized protein DNM3	DNM3		1	1	1
IPI00046057	Isoform 2 of Syntaxin-binding protein 1	STXBP1	P61764	1		
IPI00420071	microtubule-associated protein 6 isoform 1	MAP6		1	2	1
IPI00555614	Heat shock protein 90Bc	HSP90AB3P		1	2	2
IPI00026530	Protein ERGIC-53 precursor	LMAN1	P49257	1	9	7
IPI00021364	Properdin precursor	CFP	P27918	1	2	
IPI00025700	Isoform CD6A of T-cell differentiation antigen CD6 precursor	CD6	P30203	1		
IPI00061507	Isoform 3 of Ester hydrolase C11orf54	C11orf54	Q9H0W9	1		
IPI00382499	Ig heavy chain V-III region JON	-	P01780	1	1	1

Table S1.

IPI	Protein name	Gene symbol	Swiss Prot ID	Number of unique peptides identified<br>Normal	CFS	nPTLS
IPI00386754	Isoform 2 of Cysteine-rich with EGF-like domain protein 2 precursor	CRELD2	Q6UXH1	1		1
IPI00016949	Isoform 4 of Electrogenic sodium bicarbonate cotransporter 1	SLC4A4	Q9Y6R1	1		
IPI00297040	Serine protease inhibitor Kazal-type 6 precursor	SPINK6	Q6UWN8	1	4	4
IPI00454910	Serine/threonine-protein kinase MRCK gamma	CDC42BPG	Q6DT37	1	1	
IPI00031547	Desmoglein-3 precursor	DSG3	P32926	1		
IPI00384722	Isoform 2 of UPF0510 protein C19orf63 precursor	C19orf63	Q5UCC4	1	1	2
IPI00382756	Isoform 2 of Pleiotropic regulator 1	PLRG1	O43660	1		
IPI00604599	Transmembrane emp24 domain-containing protein 3 precursor	TMED3	Q9Y3Q3	1		1
IPI00743284	Methionine synthase	MTR	Q99707	1		
IPI00021363	Histone demethylase JARID1A	JARID1A	P29375	1		
IPI00749440	Uncharacterized protein ENSP00000368180	-		1		1
IPI00216683	M-phase inducer phosphatase 3	CDC25C	P30307	1		
IPI00737969	microtubule associated monoxygenase, calponin and LIM domain containing 3	MICAL3		1		
IPI00015522	Growth/differentiation factor 5 precursor	GDF5	P43026	1		
IPI00877084	Isoform 1 of Coiled-coil domain-containing protein 144C	CCDC144C	Q8IYA2	1		
IPI00060265	Zinc finger protein 775	ZNF775	Q96BV0	1		
IPI00024802	TATA-binding protein-associated factor 172	BTAF1	O14981	1		
IPI00374039	Conserved hypothetical protein	C1orf189	Q5VU69	1		
IPI00302133	Transient receptor potential cation channel subfamily V member 5	TRPV5	Q9NQA5	1	1	
IPI00028786	Isoform 3 of Polycystin-1 precursor	PKD1	P98161	1	2	
IPI00167254	Isoform 4 of Inactive phospholipase D5	PLD5	Q8N7P1	1		1
IPI00442121	delta-aminolevulinic acid dehydratase isoform a	ALAD		1		
IPI00013455	CLIP1 protein	CLIP1		1		
IPI00026299	Isoform Glycophorin C of Glycophorin-C	GYPC	P04921	1		
IPI00845508	BAH domain and coiled-coil containing 1	BAHCC1	Q9P281	1		
IPI00745300	31 kDa protein	NAT11		1		
IPI00009148	Diphosphoinositol polyphosphate phosphohydrolase 1	NUDT3	O95989	1		1
IPI00186826	Ephrin receptor	EPHB4		1		
IPI00787932	similar to zinc finger protein 10	hCG_1646157		1		
IPI00166161	Protein SIX6OS1	C14orf39	Q8N1H7	1		

Table S1.

Number of unique peptides identified

IPI	Protein name	Gene symbol	Swiss Prot ID	Normal	CFS	nPTLS
IPI00180426	Isoform 3 of G protein-coupled receptor kinase 4	GRK4	P32298	1		
IPI00013219	Integrin-linked protein kinase	ILK	Q13418	1		
IPI00010207	Ubiquitin-fold modifier 1 precursor	UFM1	P61960	1	1	
IPI00382420	Ig lambda chain V-I region HA	-	P01700	1	3	1
IPI00166553	Isoform 1 of Protein FAM19A2 precursor	FAM19A2	Q8N3H0	1	1	1
IPI00004315	Sialic acid-binding Ig-like lectin 9 precursor	SIGLEC9	Q9Y336	1	1	
IPI00796906	8 kDa protein	ABCF3		1		
IPI00028932	Microtubule-associated serine/threonine-protein kinase 3	MAST3	O60307	1		
IPI00018708	Isoform 2 of Centrosomal protein of 63 kDa	CEP63	Q96MT8	1	1	
IPI00011578	Isoform 1 of Neuroplastin precursor	NPTN	Q9Y639	1	6	3
IPI00387159	Isoform 1 of Inhibitor of growth protein 3	ING3	Q9NXR8	1		
IPI00737429	Teneurin-4	ODZ4	Q6N022	1		1
IPI00004480	ADAM DEC1 precursor	ADAMDEC1	O15204	1	2	1
IPI00645089	Kv channel interacting protein 1 isoform 3	KCNIP1		1		
IPI00167619	Leucine-rich repeat and transmembrane domain-containing protein 2 precursor	LRTM2	Q8N967	1	4	4
IPI00412216	vacuolar protein sorting 13C protein isoform 2B	VPS13C	Q709C8	1		
IPI00374862	Isoform 1 of Kelch-like protein 5	KLHL5	Q96PQ7	1		
IPI00009377	HSPC212	C3orf19		1		
IPI00005969	F-actin-capping protein subunit alpha-1	CAPZA1	P52907	1		
IPI00300244	zinc finger, CW type with PWWP domain 1	ZCWPW1	Q9H0M4	1		
IPI00060308	Isoform 6 of PDZ and LIM domain protein 7	PDLIM7	Q9NR12	1		1
IPI00333410	Isoform 1 of Ubiquitin-conjugating enzyme E2 Q1	UBE2Q1	Q7Z7E8	1		
IPI00018311	Isoform 2 of Neuroplastin precursor	NPTN	Q9Y639	1	7	3
IPI00168862	Conserved hypothetical protein	PXT1		1		1
IPI00025647	Isoform 1 of F-box only protein 21	FBXO21	O94952	1		1
IPI00387101	Ig kappa chain V-I region Scw	-	P01609	1	3	2
IPI00027429	Putative uncharacterized protein DKFZp547J2313	FABP7		1	1	2
IPI00218075	Protein FAM9B	FAM9B	Q8IZU0	1		
IPI00738499	Ferritin light chain	FTL	P02792	1		1
IPI00013281	Fukutin-related protein	FKRP	Q9H9S5	1	1	
IPI00002790	Isoform 1 of Protein sel-1 homolog 1 precursor	SEL1L	Q9UBV2	1	1	2
IPI00252845	SYT9 protein	SYT9		1		

Table S1.

Number of unique peptides identified

IPI	Protein name	Gene symbol	Swiss Prot ID	Normal	CFS	nPTLS
IPI00171611	Histone H3.2	HIST2H3C	Q71DI3	1	1	1
IPI00477361	10 kDa protein	SDHALP1		1		
IPI00397578	135 kDa protein	PPFIA4		1		
IPI00031019	Cystatin-8 precursor	CST8	O60676	1	1	1
IPI00221034	Transcription factor RelB	RELB	Q01201	1	1	
IPI00215899	Isoform 2 of Sushi repeat-containing protein SRPX precursor	SRPX	P78539	1	1	2
IPI00017659	Protein kinase substrate CapZIP	RCSD1		1		
IPI00744706	282 kDa protein	SPTAN1		1	1	1
IPI00025094	CDNA: FLJ22037 fis, clone HEP08868 (Fragment)	MYH16		1		
IPI00830057	Uncharacterized protein ENSP00000374791	-		1	2	1
IPI00023315	Bone morphogenetic protein 3b precursor	GDF10	P55107	1		
IPI00019372	Serglycin precursor	SRGN	P10124	1	1	1
IPI00215979	Bisphosphoglycerate mutase	BPGM	P07738	1	1	
IPI00216963	Isoform 9 of CASP8 and FADD-like apoptosis regulator precursor	CFLAR	O15519	1		
IPI00010895	Tubby-related protein 2	TULP2	O00295	1		
IPI00248596	similar to slit homolog 1	ELFN1		1	1	1
IPI00012391	Isoform Long of Adenomatous polyposis coli protein	APC	P25054	1	1	
IPI00739106	similar to ribosomal protein L5 isoform 1	LOC647436		1		
IPI00022606	Isoform 1 of Proline-serine-threonine phosphatase-interacting protein 1	PSTPIP1	O43586	1		
IPI00296337	Isoform 1 of DNA-dependent protein kinase catalytic subunit	PRKDC	P78527	1	1	1
IPI00550232	cardiomyopathy associated 3 isoform 1	XIRP2		1		1
IPI00299147	Small ubiquitin-related modifier 3 precursor	SUMO3	P55854	1		1
IPI00295502	Isoform 1 of Protein Wiz	WIZ	O95785	1		
IPI00435925	PP14214	IGFBP3		1		
IPI00216106	Isoform 3 of Obg-like ATPase 1	OLA1	Q9NTK5	1		1
IPI00010118	Isoform 1 of Prostate tumor overexpressed gene 1 protein	PTOV1	Q86YD1	1		
IPI00300990	Isoform 1 of Uncharacterized protein C1orf77	C1orf77	Q9Y3Y2	1		
IPI00008274	Adenylyl cyclase-associated protein 1	CAP1	Q01518	1	1	
IPI00741780	similar to CG4845-PA	LOC652559		1		
IPI00454858	similar to alpha 3 type VI collagen isoform 1 precursor	LOC344875		1		
IPI00399328	similar to jumonji domain containing 2D	LOC390245		1		
IPI00410588	ADAMTS-like protein 3 precursor	ADAMTSL3	P82987	1	1	2

Table S1.

IPI	Protein name	Gene symbol	Swiss Prot ID	Number of unique peptides identified: Normal	CFS	nPTLS
IPI00479083	Isoform 2 of Erythroid differentiation-related factor 1	C10orf137	Q3B7T1	1	1	
IPI00168885	Isoform 1 of Putative ATP-dependent RNA helicase DHX57	DHX57	Q6P158	1	1	
IPI00004534	Phosphoribosylformylglycinamidine synthase	PFAS	O15067	1		
IPI00010575	KIAA1466 protein	KIAA1466		1		
IPI00785015	Isoform 1 of Uncharacterized protein KIAA2030	FLJ25778	Q6ZU65	1		
IPI00106502	Kelch-like ECH-associated protein 1	KEAP1	Q14145	1	1	
IPI00830025	Uncharacterized protein ENSP00000375021	-		1	4	4
IPI00187143	Isoform 2 of Ras-related protein Rab-4B	RAB4B	P61018	1		1
IPI00554474	Hypothetical LOC284297	LOC284297		1	5	9
IPI00103630	Isoform 2 of Protein phosphatase 1E	PPM1E	Q8WY54	1		
IPI00479669	Isoform 1 of Uncharacterized protein KIAA0701	UHRF1BP1L	A0JNW5	1		
IPI00017940	LMBR1 domain-containing protein 2	LMBRD2	Q68DH5	1		
IPI00032405	Endothelin B receptor-like protein 2 precursor	GPR37L1	O60883	1	5	4
IPI00216508	Isoform 2 of Sorting nexin-3	SNX3	O60493	1		
IPI00011400	T-lymphoma invasion and metastasis-inducing protein 1	TIAM1	Q13009	1		1
IPI00299485	Complement component C1q receptor precursor	CD93	Q9NPY3	1	3	4
IPI00060146	Isoform 1 of Smith-Magenis syndrome chromosome region candidate gene 7 protein	SMCR7	Q96C03	1		
IPI00400986	hypothetical protein LOC85459	KIAA1731		1	2	1
IPI00329688	Protein YIPF3	YIPF3	Q9GZM5	1	3	3
IPI00011643	Isoform 2 of Kunitz-type protease inhibitor 1 precursor	SPINT1	O43278	1	3	3
IPI00022989	Isoform Beta-1 of Retinoic acid receptor beta	RARB	P10826	1		
IPI00152072	hypothetical protein LOC387758	FIBIN		1		
IPI00748955	platelet glycoprotein Ib alpha polypeptide precursor	GP1BA	P07359	1	1	
IPI00060546	Uncharacterized protein C10orf35	C10orf35	Q96D05	1		
IPI00412541	Probable G-protein coupled receptor 158 precursor	GPR158	Q5T848	1	2	2
IPI00217781	Similar to expressed sequence AI593442	LOC399947		1	4	1
IPI00043978	Isoform 1 of Partitioning-defective 3 homolog B	PARD3B	Q8TEW8	1		

Table S1.

IPI	Protein name	Gene symbol	Swiss Prot ID	Number of unique peptides identified<br>Normal	CFS	nPTLS
IPI00440221	Putative uncharacterized protein (Fragment)	CDC2L5		1		
IPI00011564	Syndecan-4 precursor	SDC4	P31431	1		
IPI00031008	Isoform 1 of Tenascin precursor	TNC	P24821	1	3	5
IPI00423683	Isoform 2 of EMI domain-containing protein 1 precursor	EMID1	Q96A84	1		
IPI00026612	Isoform Beta-1 of Protein phosphatase 1B	PPM1B	O75688	1		
IPI00103552	Mucin-16	MUC16	Q8WXI7	1	1	1
IPI00167137	Isoform 3 of SLAM family member 7 precursor	SLAMF7	Q9NQ25	1		
IPI00155447	MMP28 protein	MMP28		1		
IPI00001434	Protocadherin beta 14 precursor	PCDHB14	Q9Y5E9	1		
IPI00443909	Isoform 1 of Protein canopy homolog 2 precursor	CNPY2	Q9Y2B0	1	2	3
IPI00854745	Uncharacterized protein ENSP00000375019	-		1	6	4
IPI00043731	CDNA FLJ30671 fis, clone FCBBF1000687, moderately similar to Mus musculus Rap2 interacting protein 8 (RPIP8) mRNA	RUNDC3B		1		
IPI00024853	Isoform 1 of Periaxin	PRX	Q9BXM0	1	1	
IPI00645814	Isoform 2 of MAP7 domain-containing protein 1	MAP7D1	Q3KQU3	1		
IPI00003353	Neuronal protein 3.1	C5orf13	Q16612	1		
IPI00007402	Importin-7	IPO7	O95373	1		
IPI00152470	Prokineticin receptor 1	PROKR1	Q8TCW9	1		
IPI00006094	Regulating synaptic membrane exocytosis protein 3	RIMS3	Q9UJD0	1		
IPI00169426	Isoform 2 of Cytosolic 5'-nucleotidase 1B	NT5C1B	Q96P26	1	1	1
IPI00101927	Leucine zipper putative tumor suppressor 2	LZTS2	Q9BRK4	1		
IPI00816794	REV25-2 (Fragment)	-		1	1	2
IPI00010346	Neurolysin, mitochondrial precursor	NLN	Q9BYT8	1		
IPI00013216	Origin recognition complex subunit 2	ORC2L	Q13416	1	1	1
IPI00216572	BarH-like homeobox 2	BARX2	Q9UMQ3	1		
IPI00644025	Isoform 1 of Synaptic vesicle glycoprotein 2A	SV2A	Q7L0J3	1		1
IPI00171647	Isoform 1 of Sialic acid-binding Ig-like lectin 8 precursor	SIGLEC8	Q9NYZ4	1		
IPI00470838	Isoform 1 of DENN domain-containing protein 2C	DENND2C	Q68D51	1	1	1
IPI00020058	Isoform 1 of Copper-transporting ATPase 2	ATP7B	P35670	1		

Table S1.

Number of unique peptides identified

IPI	Protein name	Gene symbol	Swiss Prot ID	Normal	CFS	nPTLS
IPI00026570	Cytochrome c oxidase polypeptide VIIa-liver/heart, mitochondrial precursor	COX7A2	P14406	1		
IPI00027726	Isoform 1 of Krueppel-like factor 3	KLF3	P57682	1		
IPI00064241	Isoform 1 of Zinc finger protein Eos	IKZF4	Q9H2S9	1		
IPI00295503	Isoform 2 of Probable ATP-dependent RNA helicase DDX58	DDX58	O95786	1		
IPI00307591	Zinc finger protein 609	ZNF609	O15014	1	1	
IPI00290292	Rhomboid 5 homolog 1	RHBDF1		1		
IPI00008580	Antileukoproteinase precursor	SLPI	P03973	1		
IPI00003348	Guanine nucleotide-binding protein G(I)/G(S)/G(T) subunit beta-2	GNB2	P62879	1		
IPI00299435	apolipoprotein F precursor	APOF		1	1	1
IPI00029533	Integrin beta-8 precursor	ITGB8	P26012	1		
IPI00815786	Hexokinase 1 (Fragment)	HK1		1		
IPI00375881	Polycystic kidney disease 1-like protein 3	PKD1L3		1	1	
IPI00008998	Protein tyrosine phosphatase-like protein PTPLAD1	PTPLAD1	Q9P035	1		
IPI00658112	32 kDa protein	SPEG		1		
IPI00479361	Isoform 1 of UDP-GlcNAc:betaGal beta-1,3-N-acetylglucosaminyltransferase 4	B3GNT4	Q9C0J1	1		
IPI00448465	Isoform 1 of Ankyrin repeat domain-containing protein 12	ANKRD12	Q6UB98	1		
IPI00025869	Alpha-galactosidase A precursor	GLA	P06280	1	1	1
IPI00745122	Conserved hypothetical protein	MGC33894		1		
IPI00783471	Immunglobulin heavy chain variable region (Fragment)	-		1	3	2
IPI00005531	Isoform 1 of Probable DNA dC->dU-editing enzyme APOBEC-3B	APOBEC3B	Q9UH17	1		
IPI00023542	transmembrane emp24 protein transport domain containing 9	TMED9	Q9BVK6	1		1
IPI00783313	Glycogen phosphorylase, liver form	PYGL	P06737	1		1
IPI00383832	Protein kinase C-binding protein RACK8	DVL3		1		
IPI00027685	C-C chemokine receptor type 1	CCR1	P32246	1		
IPI00009335	Brain protein 16	C8orf30A	Q9BTY7	1		
IPI00432755	PPRR6495	FAM124A		1		
IPI00796777	17 kDa protein	CRYAA		1		
IPI00008894	Carboxypeptidase A4 precursor	CPA4	Q9UI42	1		
IPI00387095	Ig kappa chain V-I region Ka	-	P01603	1	3	3
IPI00847759	DENN domain-containing protein 4B	DENND4B	O75064	1		
IPI00008315	Isoform 1 of Ephrin type-B receptor 1 precursor	EPHB1	P54762	1		1
IPI00031005	Protein kinase-like protein SgK196	FLJ23356	Q9H5K3	1		

Table S1.

Number of unique peptides identified

IPI	Protein name	Gene symbol	Swiss Prot ID	Normal	CFS	nPTLS
IPI00787414	Uncharacterized protein ENSP00000381388	MGC34829		1		
IPI00019988	N-sulphoglucosamine sulphohydrolase precursor	SGSH	P51688	1		
IPI00013860	3-hydroxyisobutyrate dehydrogenase, mitochondrial precursor	HIBADH	P31937	1	1	1
IPI00059164	Galactose-3-O-sulfotransferase 3	GAL3ST3	Q96A11	1		1
IPI00175654	Probable mast cell antigen 32 homolog precursor	C17orf60	Q7Z6M3	1	1	1
IPI00873740	Uncharacterized protein ENSP00000383832 (Fragment)	-		1		
IPI00032416	Isoform Long of Protein jagged-2 precursor	JAG2	Q9Y219	1		
IPI00335589	RNA methyltransferase-like protein 1	RNMTL1	Q9HC36	1		
IPI00377045	Alpha3A	LAMA3		1	1	1
IPI00797310	14 kDa protein	CLSTN3		1	1	1
IPI00843819	Similar to Dual specificity protein kinase CLK2	-		1		
IPI00065276	Isoform 2 of Tether containing UBX domain for GLUT4	ASPSCR1	Q9BZE9	1		
IPI00644522	PNKP protein	PNKP		1		
IPI00045511	Isoform 1 of Chloride channel CLIC-like protein 1 precursor	CLCC1	Q96S66	1		
IPI00604763	Transmembrane protein 66 precursor	TMEM66	Q96BY9	1		
IPI00829759	Uncharacterized protein ENSP00000375040	-		1	1	2
IPI00235647	similar to fibrillarin	LOC345630		1		
IPI00294910	Protein PARM-1 precursor	DKFZP564O0823	Q6UWI2	1		
IPI00008433	40S ribosomal protein S5	RPS5	P46782	1		
IPI00217652	Isoform 1 of Glycosyltransferase 8 domain-containing protein 3	GLT8D3	Q4G148	1	1	
IPI00219525	6-phosphogluconate dehydrogenase, decarboxylating	PGD	P52209	1	1	
IPI00470805	Isoform 2 of Mediator of DNA damage checkpoint protein 1	MDC1	Q14676	1		
IPI00167089	Isoform 2 of Activated CDC42 kinase 1	TNK2	Q07912	1		
IPI00385143	Microfibrillar protein 2 (Fragment)	-		1	2	3
IPI00783753	UPF0235 protein C15orf40	C15orf40	Q8WUR7	1		
IPI00445716	Isoform 1 of GDNF family receptor alpha-3 precursor	GFRA3	O60609	1	4	1
IPI00020501	Myosin-11	MYH11	P35749	1		1
IPI00030919	Mitogen-activated protein kinase kinase 1-interacting protein 1	MAP2K1IP1	Q9UHA4	1		
IPI00827745	Isoform 1 of RNA-binding protein 24	RBM24	Q9BX46	1		

Table S1.

Number of unique peptides identified

IPI	Protein name	Gene symbol	Swiss Prot ID	Normal	CFS	nPTLS
IPI00005776	Nucleotide-binding oligomerization domain-containing protein 1	NOD1	Q9Y239	1		
IPI00412264	Pleiotrophin precursor	PTN	P21246	1	1	4
IPI00394879	Leucine-rich repeat-containing protein 9	LRRC9		1		
IPI00401852	Conserved hypothetical protein	DKFZP434L187		1		
IPI00442865	CDNA FLJ26488 fis, clone KDN05770, highly similar to Bumetanide- sensitive sodium-(potassium)-chloride cotransporter 2	SLC12A1		1		
IPI00306884	CDNA FLJ11867 fis, clone HEMBA1006976, weakly similar to H.sapiens Gal-beta(1-3/1-4)GlcNAc alpha-2.3-sialyltransferase	ST3GAL4		1	1	
IPI00748891	hypothetical protein LOC283635 isoform 1	C14orf24		1		
IPI00414320	Annexin A11	ANXA11	P50995	1		
IPI00874023	Uncharacterized protein ENSP00000379699	-		1		
IPI00827584	similar to kinesin family member 27	KIF27		1		
IPI00020199	Alpha-2,8-sialyltransferase 8B	ST8SIA2	Q92186	1		
IPI00297714	Gamma-synuclein	SNCG	O76070	1	1	3
IPI00005158	Lon protease homolog, mitochondrial precursor	LONP1	P36776	1	1	
IPI00295618	Isoform Long of Platelet endothelial cell adhesion molecule precursor	PECAM1	P16284	1	1	1
IPI00791513	CDNA FLJ16614 fis, clone TESTI4013365	CTA-216E10.6		1		
IPI00013319	Isoform 2 of 43 kDa receptor-associated protein of the synapse	RAPSN	Q13702	1	1	
IPI00828037	Heavy chain Fab (Fragment)	-		1	2	2
IPI00020201	CMP-N-acetylneuraminate-poly-alpha-2,8-sialyltransferase	ST8SIA4	Q92187	1		1
IPI00217537	Isoform 1 of Putative Polycomb group protein ASXL1	ASXL1	Q8IXJ9	1	1	1
IPI00010133	Coronin-1A	CORO1A	P31146	1	2	1
IPI00002884	CDNA: FLJ22222 fis, clone HRC01658	FLJ22222		1		
IPI00025092	Myosin-binding protein C, slow-type	MYBPC1	Q00872	1		1
IPI00003363	Isoform 1 of Protein phosphatase 1 regulatory subunit 1B	PPP1R1B	Q9UD71	1		
IPI00013991	Isoform 1 of Tropomyosin beta chain	TPM2	P07951	1	1	2
IPI00005705	Isoform Gamma-1 of Serine/threonine-protein phosphatase PP1-gamma catalytic subunit	PPP1CC	P36873	1		
IPI00853073	Protein	GPX3		1	2	3

Table S1.

Number of unique peptides identified

IPI	Protein name	Gene symbol	Swiss Prot ID	Normal	CFS	nPTLS
IPI00022078	Protein NDRG1	NDRG1	Q92597	1		1
IPI00290857	Keratin, type II cytoskeletal 3	KRT3	P12035	1	1	
IPI00164949	Isoform NELF-C of Negative elongation factor C/D	TH1L	Q8IXH7	1		
IPI00029647	Zymogen granule membrane protein 16 precursor	ZG16	O60844	1		
IPI00414481	GTF3C1 protein	GTF3C1		1		
IPI00303882	Isoform B of Mannose-6-phosphate receptor-binding protein 1	M6PRBP1	O60664	1	1	2
IPI00640818	Isoform 3 of Neuropathy target esterase	PNPLA6	Q8IY17	1		1
IPI00154528	Isoform 1 of Structural maintenance of chromosomes protein 6	SMC6	Q96SB8	1		
IPI00164861	Isoform 3 of Kinesin-like protein KIF13A	KIF13A	Q9H1H9	1		
IPI00411674	Isoform 1 of Zinc finger protein 254	ZNF254	O75437	1		
IPI00025365	Isoform Long of Endothelin-3 precursor	EDN3	P14138	1	3	3
IPI00020131	Son of sevenless homolog 1	SOS1	Q07889	1	1	1
IPI00828191	NANUC-2 heavy chain (Fragment)	-		1	2	2
IPI00249982	Isoform 1 of Death-inducer obliterator 1	DIDO1	Q9BTC0	1		
IPI00025473	Beta-1,4 N-acetylgalactosaminyltransferase 1	B4GALNT1	Q00973	1	3	2
IPI00328270	Neuronal PAS domain-containing protein 2	NPAS2	Q99743	1		
IPI00024887	Bone morphogenetic protein 6 precursor	BMP6	P22004	1	3	1
IPI00432707	Caspase-12	CASP12		1	1	
IPI00442544	CDNA FLJ27034 fis, clone SLV07984	-		1		
IPI00175019	similar to Temporarily Assigned Gene name family member	LOC643677		1		
IPI00027898	Isoform A of Uncharacterized protein C21orf70	C21orf70	Q9NSI2	1		
IPI00216049	Isoform 1 of Heterogeneous nuclear ribonucleoprotein K	HNRPK	P61978	1	1	
IPI00019485	Isoform 2 of Enoyl-CoA hydratase domain-containing protein 2, mitochondrial precursor	ECHDC2	Q86YB7	1	2	2
IPI00023340	Histone acetyltransferase MYST3	MYST3	Q92794	1		
IPI00300052	Keratin type II cuticular Hb4	KRT84	Q9NSB2	1		
IPI00383594	melanoma ubiquitous mutated protein	MUM1	Q2TAK8	1	1	
IPI00003111	Ig kappa chain V-I region AU	LOC652694	P01594	1	4	3
IPI00003031	Isoform 2 of Isochorismatase domain-containing protein 2, mitochondrial precursor	ISOC2	Q96AB3	1	1	1
IPI00019158	ADAM metallopeptidase domain 8 precursor	ADAM8	P78325	1		
IPI00005605	Isoform 1 of Protein NDRG3	NDRG3	Q9UGV2	1	1	1

Table S1.

Number of unique peptides identified

IPI	Protein name	Gene symbol	Swiss Prot ID	Normal	CFS	nPTLS
IPI00045928	Sodium/hydrogen exchanger 7	SLC9A7	Q96T83	1		
IPI00009532	4-aminobutyrate aminotransferase, mitochondrial precursor	ABAT	P80404	1		
IPI00306332	60S ribosomal protein L24	RPL24	P83731	1		
IPI00015047	8D6 antigen (Fragment)	CD320		1		
IPI00643937	Methylenetetrahydrofolate dehydrogenase (NADP+ dependent) 1-like	MTHFD1L		1		
IPI00001786	Isoform 2 of Ubiquitin carboxyl-terminal hydrolase 36	USP36	Q9P275	1		
IPI00745103	similar to melanoma associated antigen (mutated) 1-like 1	LOC728307		1		1
IPI00030876	diaphanous 1 isoform 2	DIAPH1		1		
IPI00827846	Anti-mucin1 heavy chain variable region (Fragment)	-		1	3	3
IPI00385791	Serologically defined breast cancer antigen NY-BR-87 (Fragment)	MRPS26		1		
IPI00031627	DNA-directed RNA polymerase II subunit RPB1	POLR2A	P24928	1		
IPI00145805	Isoform 1 of TRAF2 and NCK-interacting protein kinase	TNIK	Q9UKE5	1		
IPI00296374	Zinc finger protein-like 1	ZFPL1	O95159	1		
IPI00021733	Bifunctional heparan sulfate N-deacetylase/N-sulfotransferase 4	NDST4	Q9H3R1	1	1	2
IPI00184884	Non-structural maintenance of chromosomes element 1 homolog	NSMCE1	Q8WV22	1		
IPI00103874	Isoform 1 of Zinc finger FYVE domain-containing protein 1	ZFYVE1	Q9HBF4	1		
IPI00737920	similar to dynein, axonemal, heavy polypeptide 1	DNAH3		1		
IPI00218914	Retinal dehydrogenase 1	ALDH1A1	P00352	1	2	1
IPI00243221	nardilysin (N-arginine dibasic convertase) isoform a	NRD1	O43847	1		2
IPI00220791	Amphiphysin I variant CT2	AMPH		1	1	2
IPI00015983	Sphingosine 1-phosphate receptor Edg-3	EDG3	Q99500	1		
IPI00289965	Potassium voltage-gated channel subfamily C member 3	KCNC3	Q14003	1		
IPI00374129	NLR family, pyrin domain containing 3 isoform b	NLRP3	Q96P20	1		
IPI00007617	Olfactory receptor 52A1	OR52A1	Q9UKL2	1	1	1
IPI00297288	Cdc42 GTPase-activating protein	CDGAP		1	1	1
IPI00456578	LOC441054 protein	LOC441054		1		

Table S1.

IPI	Protein name	Gene symbol	Swiss Prot ID	Number of unique peptides identified<br>Normal	CFS	nPTLS
IPI00748890	Isoform 1 of Leucine zipper protein 2 precursor	LUZP2	Q86TE4	1	2	2
IPI00013302	ADAM 15 precursor	ADAM15	Q13444	1	1	2
IPI00376587	Uncharacterized protein ENSP00000345065	LOC728780		1		
IPI00333197	Isoform 2 of GRIP and coiled-coil domain-containing protein 2	GCC2	Q8IWJ2	1		1
IPI00004346	C-C chemokine receptor type 10	CCR10	P46092	1		
IPI00297251	Isoform 2 of Probable E3 ubiquitin-protein ligase MGRN1	MGRN1	O60291	1		1
IPI00218131	Protein S100-A12	S100A12	P80511	1		
IPI00438170	Isoform 1 of Sorting nexin-12	SNX12	Q9UMY4	1		
IPI00440153	68 kDa protein	XRRA1		1	1	1
IPI00373823	Cytochrome P450 26C1	CYP26C1	Q6V0L0	1		
IPI00550917	Twinfilin-2	TWF2	Q6IBS0	1		
IPI00014516	Isoform 1 of Caldesmon	CALD1	Q05682	1		
IPI00301294	Protein FAM134A	FAM134A	Q8NC44	1	3	2
IPI00021951	Uncharacterized protein KIAA0247 precursor	KIAA0247	Q92537	1		
IPI00815893	Isoform 1 of Chromodomain-helicase-DNA-binding protein 2	CHD2	O14647	1		
IPI00002243	Isoform 1 of Gamma-glutamyltransferase 5 precursor	GGTLA1	P36269	1		
IPI00159049	SET-binding protein	SETBP1		1		
IPI00005837	Angiopoietin-related protein 1 precursor	ANGPTL1	O95841	1		
IPI00297277	Isoform 1 of RING finger protein 150 precursor	RNF150	Q9ULK6	1		1
IPI00031765	Isoform 2 of Protocadherin gamma C4 precursor	PCDHGC4	Q9Y5F7	1	1	1
IPI00297208	similar to Myosin-10	KIAA1276		1	2	1
IPI00016685	Enamelin precursor	ENAM	Q9NRM1	1		
IPI00002191	Putative uncharacterized protein FLJ12684	-		1		
IPI00007928	Pre-mRNA-processing-splicing factor 8	PRPF8	Q6P2Q9	1		
IPI00022542	Rho-associated protein kinase 1	ROCK1	Q13464	1	1	1
IPI00028053	Gap junction alpha-9 protein	GJA9	P57773	1		
IPI00005732	Isoform 1 of Activin receptor type-1B precursor	ACVR1B	P36896	1	2	3
IPI00024138	Uncharacterized protein ENSP00000374816	-		1	1	1
IPI00293095	Isoform 1 of Coiled-coil domain-containing protein 83	CCDC83	Q8IWF9	1		
IPI00747142	Centaurin-gamma-like family member 6	CTGLF6	Q5VTM2	1		

Table S1. Number of unique peptides identified

IPI	Protein name	Gene symbol	Swiss Prot ID	Normal	CFS	nPTLS
IPI00040900	Isoform 2 of Heparan sulfate 2-O-sulfotransferase 1	HS2ST1	Q7LGA3	1		
IPI00011515	Protein kinase C and casein kinase substrate in neurons protein 1	PACSIN1	Q9BY11	1		1
IPI00164066	Isoform 4 of Coiled-coil domain-containing protein 136	CCDC136	Q96JN2	1		
IPI00307702	H53_GS1 (Fragment)	-		1		
IPI00025084	Calpain small subunit 1	CAPNS1	P04632	1		
IPI00014899	CDNA FLJ20744 fis, clone HEP06585	BRPF3		1		
IPI00218398	Matrix metalloproteinase-14 precursor	MMP14	P50281	1	2	3
IPI00307611	Isoform 1 of Microtubule-associated serine/threonine-protein kinase 4	MAST4	O15021	1		
IPI00014444	Isoform 1 of Protein SERAC1	SERAC1	Q96JX3	1		
IPI00479217	Isoform Short of Heterogeneous nuclear ribonucleoprotein U	HNRNPU	Q00839	1		1
IPI00183206	Isoform 1 of RIM-binding protein 2	RIMBP2	O15034	1		
IPI00220070	6-phosphofructo-2-kinase/fructose-2,6-biphosphatase 4	PFKFB4	Q16877	1		
IPI00023322	Zinc finger protein ubi-d4	DPF2	Q92785	1		
IPI00013978	Speckle-type POZ protein	SPOP	O43791	1	1	
IPI00303318	Protein FAM49B	FAM49B	Q9NUQ9	1		1
IPI00014398	Four and a half LIM domains 1 variant	FHL1		1	1	
IPI00784739	Uncharacterized protein C14orf43	C14orf43	Q6PJG2	1		
IPI00335946	Family with sequence similarity 120B	FAM120B		1	1	
IPI00738920	similar to CG3104-PA, isoform A	LOC642574		1		
IPI00056314	Pre-rRNA-processing protein TSR2 homolog	TSR2	Q969E8	1		
IPI00874156	Isoform 1 of Ubiquitin thioesterase OTUB1	OTUB1	Q96FW1	1	1	1
IPI00797694	3 kDa protein	-		1		
IPI00294210	DNA-binding protein inhibitor ID-2	ID2	Q02363	1		
IPI00004373	Mannose-binding protein C precursor	MBL2	P11226	1		1
IPI00787020	similar to Dynamin-1	LOC644153		1		
IPI00827522	Anti-streptococcal/anti-myosin immunoglobulin lambda light chain variable region (Fragment)	IGLV1-44		1	3	2
IPI00167941	Midasin	MDN1	Q9NU22	1		
IPI00807609	Aberrant LSLCL	CLEC11A		1	1	1
IPI00016701	P2Y purinoceptor 14	P2RY14	Q15391	1		
IPI00007010	Lysozyme-like protein 6 precursor	LYZL6	O75951	1		
IPI00166039	Isoform 1 of Scotin precursor	SCOTIN	Q8N114	1	5	4
IPI00009899	Uncharacterized protein C5orf5	C5orf5	Q9NYF5	1		
IPI00005129	Isoform 1 of Secretory carrier-associated membrane protein 1	SCAMP1	O15126	1	2	2

Table S1.

Number of unique peptides identified

IPI	Protein name	Gene symbol	Swiss Prot ID	Normal	CFS	nPTLS
IPI00217948	FRMD4B protein	FRMD4B		1		
IPI00002412	Palmitoyl-protein thioesterase 1 precursor	PPT1	P50897	1	3	2
IPI00027457	C1q-related factor precursor	C1QL1	O75973	1		1
IPI00002993	Transcription initiation factor TFIID subunit 9	TAF9	Q16594	1		
IPI00555600	Solute carrier family 26, member 1 isoform a variant (Fragment)	IDUA		1		
IPI00419221	Membrane-bound O-acyltransferase domain-containing protein 2	MBOAT2	Q6ZWT7	1		
IPI00299778	Serum paraoxonase/lactonase 3	PON3	Q15166	1	2	
IPI00021634	Kinesin light chain 2	KLC2	Q9H0B6	1		
IPI00451429	NIF3L1 isoform gamma	NIF3L1		1		
IPI00384225	Meteorin precursor	METRN	Q9UJH8	1	1	3
IPI00375803	Isoform 1 of GON-4-like protein	GON4L	Q3T8J9	1		
IPI00023184	Isoform 1 of Poly [ADP-ribose] polymerase 3	PARP3	Q9Y6F1	1		
IPI00872739	Uncharacterized protein C18orf2	C18orf2		1		
IPI00241409	hypothetical protein LOC55747	FAM21B		1		
IPI00090764	Toll-like receptor 1 precursor	TLR1	Q15399	1		
IPI00141938	H2A histone family, member V isoform 2	H2AFV		1	2	
IPI00024253	Isoform 1 of Fibroblast growth factor 14	FGF14	Q92915	1		
IPI00387096	Ig kappa chain V-I region Kue	-	P01604	1	2	3
IPI00218637	Major histocompatibility complex, class II, DQ beta 2	HLA-DQB2		1		
IPI00218407	Fructose-bisphosphate aldolase B	ALDOB	P05062	1	3	5
IPI00216921	Isoform 2 of Stathmin-4	STMN4	Q9H169	1		
IPI00011416	Delta(3,5)-Delta(2,4)-dienoyl-CoA isomerase, mitochondrial precursor	ECH1	Q13011	1		
IPI00260755	similar to Rho GTPase activating protein 18	C20orf95		1		
IPI00337385	Isoform 1 of Pre-mRNA-processing factor 40 homolog A	PRPF40A	O75400	1		
IPI00304527	Protein FAM83B	FAM83B	Q5T0W9	1		
IPI00000459	Transmembrane gamma-carboxyglutamic acid protein 1 precursor	PRRG1	O14668	1		1
IPI00030741	Uncharacterized protein C21orf13	LCA5L	O95447	1		
IPI00399296	hypothetical protein LOC390110	LOC390110		1		
IPI00233358	islet cell autoantigen 1,69kDa-like isoform 2	ICA1L		1		
IPI00879842	6 kDa protein	-		1		
IPI00021476	Eukaryotic translation initiation factor 4E-binding protein 3	EIF4EBP3	O60516	1		

Table S1.

Number of unique peptides identified

IPI	Protein name	Gene symbol	Swiss Prot ID	Normal	CFS	nPTLS
IPI00028383	Uncharacterized protein C16orf24	C16orf24	Q9BQD7	1		
IPI00010369	Testis-expressed sequence 15 protein	TEX15	Q9BXT5	1	1	1
IPI00001863	Wnt inhibitory factor 1 precursor	WIF1	Q9Y5W5	1	1	
IPI00012948	Proheparin-binding EGF-like growth factor precursor	HBEGF	Q99075	1		1
IPI00298337	cDNA FLJ77671	SLC14A1	Q13336	1		
IPI00219005	FK506-binding protein 4	FKBP4	Q02790	1		1
IPI00044842	Isoform 2 of RAB3A-interacting protein	RAB3IP	Q96QF0	1		
IPI00021900	Tumor necrosis factor ligand superfamily member 12	TNFSF12	O43508	1	3	6
IPI00013004	Isoform 1 of Pyridoxal kinase	PDXK	O00764	1		
IPI00022055	Histone acetyltransferase PCAF	PCAF	Q92831	1		1
IPI00328260	Protein FAN	NSMAF	Q92636	1		
IPI00807418	Isoform 9 of Lymphoid-specific helicase	HELLS	Q9NRZ9	1		
IPI00183002	Isoform 1 of Protein phosphatase 1 regulatory subunit 12A	PPP1R12A	O14974	1		
IPI00163601	Putative uncharacterized protein FLJ10213	FLJ10213		1		
IPI00428741	LP2477	FLJ35348		1		
IPI00289334	Isoform 1 of Filamin-B	FLNB	O75369	1	1	4
IPI00006252	Multisynthetase complex auxiliary component p43	SCYE1	Q12904	1		
IPI00021907	Isoform 1 of Myelin basic protein	MBP	P02686	1		
IPI00007321	Isoform 1 of Acyl-protein thioesterase 1	LYPLA1	O75608	1		
IPI00217617	palmitoylated membrane protein 7	MPP7		1		
IPI00008303	Isoform 1 of N-acetylglucosamine-1-phosphodiester alpha-N-acetylglucosaminidase precursor	NAGPA	Q9UK23	1		1
IPI00306850	EGF-like-domain, multiple 3	MEGF6		1	1	1
IPI00064935	Alpha-protein kinase 3	ALPK3	Q96L96	1	1	1
IPI00300407	Syndecan-2 precursor	SDC2	P34741	1	1	1
IPI00061448	13 kDa protein	-		1	2	3
IPI00024662	Chromobox protein homolog 5	CBX5	P45973	1		
IPI00550364	Phosphoglucomutase-2	PGM2	Q96G03	1		
IPI00169331	Phosphatidylcholine:ceramide cholinephosphotransferase 2	SGMS2	Q8NHU3	1		
IPI00028450	Isoform 1 of Sodium/calcium exchanger 1 precursor	SLC8A1	P32418	1		
IPI00783464	dynein heavy chain domain 3	DNAH2		1		1
IPI00298702	solute carrier family 39 (zinc transporter), member 6 isoform 1	SLC39A6	Q13433	1	3	4
IPI00185088	immunoglobulin superfamily, member 11 isoform b	IGSF11		1	1	

Table S1.

Number of unique peptides identified

IPI	Protein name	Gene symbol	Swiss Prot ID	Normal	CFS	nPTLS
IPI00010405	Isoform Long of Tyrosine-protein kinase transmembrane receptor ROR1 precursor	ROR1	Q01973	1		
IPI00873774	Uncharacterized protein ENSP00000383488 (Fragment)	-		1		
IPI00847335	FLJ45422 protein	-		1		
IPI00218465	Phospholipase A-2-activating protein	PLAA	Q9Y263	1	1	
IPI00217740	C20orf12 protein	C20orf12		1		
IPI00877800	32 kDa protein	-		1		2
IPI00008497	Ornithine decarboxylase	ODC1	P11926	1		
IPI00026358	Gamma-aminobutyric acid receptor-associated protein-like 2	GABARAPL2	P60520	1	1	1
IPI00010808	Interferon-gamma receptor alpha chain precursor	IFNGR1	P15260	1		
IPI00872550	Uncharacterized protein PRDM2	PRDM2		1		
IPI00827485	BRE (Fragment)	-		1	3	1
IPI00514622	Ran-binding protein 6	RANBP6	O60518	1		
IPI00382421	Ig lambda chain V-I region NEW	-	P01701	1	1	1
IPI00030877	15 kDa selenoprotein isoform 1 precursor	SEP15	O60613	1	2	2
IPI00009203	Sorting nexin-7	SNX7	Q9UNH6	1		
IPI00790021	Zinc finger protein 652	ZNF652	Q9Y2D9	1		
IPI00000027	Pituitary adenylate cyclase-activating polypeptide precursor	ADCYAP1	P18509	1	3	4
IPI00289861	Isoform 1 of Zinc finger CCHC domain-containing protein 11	ZCCHC11	Q5TAX3	1	1	1
IPI00855918	mucin 5, subtype B, tracheobronchial	MUC5B	Q9HC84	1		
IPI00033600	Isoform 1 of Protein phosphatase 1 regulatory subunit 7	PPP1R7	Q15435	1	1	
IPI00375746	Isoform 1 of Guanylate-binding protein 6	GBP6	Q6ZN66	1	1	1
IPI00180384	dynein, axonemal, heavy chain 7	DNAH7		1		1
IPI00176920	Nephrocystin-4	NPHP4	O75161	1	1	1
IPI00045839	Isoform 3 of Prolyl 3-hydroxylase 1 precursor	LEPRE1	Q32P28	1	1	
IPI00008404	Isoform Long of Segment polarity protein dishevelled homolog DVL-1-like	DVL1L1	P54792	1		
IPI00658025	Putative novel transcript	-		1		
IPI00022958	PRO0149	C16orf72		1		
IPI00032313	Protein S100-A4	S100A4	P26447	1		
IPI00006987	ATP-dependent RNA helicase DDX24	DDX24	Q9GZR7	1	1	1
IPI00027626	T-complex protein 1 subunit zeta	CCT6A	P40227	1		
IPI00001960	Chloride intracellular channel protein 4	CLIC4	Q9Y696	1	4	5
IPI00027009	Isoform 1 of Protein kinase C and casein kinase substrate in neurons protein 2	PACSIN2	Q9UNF0	1		

Table S1.

				Number of unique peptides identified		
**IPI**	**Protein name**	**Gene symbol**	**Swiss Prot ID**	**Normal**	**CFS**	**nPTLS**
IPI00023162	UDP-N-acetylglucosamine 2-epimerase/N-acetylmannosamine kinase	GNE	Q9Y223	1		
IPI00019146	Isoform 1 of Coxsackievirus and adenovirus receptor precursor	CXADR	P78310	1	2	3
IPI00789181	115 kDa protein	PLCL1		1		
IPI00005132	Guanine nucleotide-binding protein-like 3-like protein	GNL3L	Q9NVN8	1		
IPI00742725	Conserved hypothetical protein	LOC388564		1		
IPI00878755	43 kDa protein	-		1		
IPI00152050	ataxin 2-binding protein 1 isoform 3	A2BP1		1		
IPI00385480	Caskin-1	CASKIN1	Q8WXD9	1		
IPI00465123	KIAA0415 gene product	KIAA0415	O43299	1		
IPI00013495	Isoform 2 of ATP-binding cassette sub-family F member 1	ABCF1	Q8NE71	1	1	1
IPI00398992	Isoform 1 of Chromodomain-helicase-DNA-binding protein 8	CHD8	Q9HCK8	1		
IPI00827724	Rheumatoid factor Vh I region precursor (Fragment)	-		1	1	1
IPI00012441	Isoform 1 of Synaptojanin-1	SYNJ1	O43426	1		
IPI00006054	Syntaphilin	SNPH	O15079	1		
IPI00215746	Fatty acid-binding protein, adipocyte	FABP4	P15090	1		
IPI00216780	Cartilage intermediate layer protein 2 precursor	CILP2	Q8IUL8	1		1
IPI00385918	CDNA FLJ90582 fis, clone PLACE1000442, moderately similar to ZINC FINGER PROTEIN ZFP-36	ZNF627		1		
IPI00008438	40S ribosomal protein S10	RPS10	P46783	1		
IPI00152849	Isoform 1 of G2/mitotic-specific cyclin-B3	CCNB3	Q8WWL7	1		
IPI00029556	Uncharacterized protein C1orf105	C1orf105	O95561	1		
IPI00217012	pleckstrin and Sec7 domain containing	PSD		1		
IPI00218730	Rod cGMP-specific 3',5'-cyclic phosphodiesterase subunit alpha	PDE6A	P16499	1	1	
IPI00215914	ADP-ribosylation factor 1	ARF1	P84077	1		
IPI00290826	Transmembrane protein 157 precursor	TMEM157	Q8TBP5	1	1	1
IPI00553092	V3-3 protein	IGLV7-46		1	1	2
IPI00218820	Isoform 3 of Tropomyosin beta chain	TPM2	P07951	1	1	2
IPI00015980	Isoform 2 of Multiple PDZ domain protein	MPDZ	O75970	1		
IPI00830122	A30	-		1	5	5
IPI00383603	Anti-thyroglobulin light chain variable region (Fragment)	-		1	1	1
IPI00641251	CD320 antigen precursor	CD320	Q9NPF0	1	1	1
IPI00103853	Isoform 1 of Putative ribosome-binding factor A, mitochondrial precursor	C18orf22	Q8N0V3	1		1

Table S1.

Number of unique peptides identified

IPI	Protein name	Gene symbol	Swiss Prot ID	Normal	CFS	nPTLS
IPI00301465	14-3-3-associated AKT substrate	HJURP	Q8NCD3	1	1	
IPI00102678	Isoform 1 of Pecanex-like protein 1	PCNX	Q96RV3	1		
IPI00020356	331 kDa protein	MAP1A		1		
IPI00008226	73 kDa protein	THSD3		1		
IPI00219622	Proteasome subunit alpha type-2	PSMA2	P25787	1		
IPI00010903	Dopey family member 1	DOPEY1		1		
IPI00735934	similar to capicua homolog	LOC646070		1		
IPI00031086	Insulin-like growth factor-binding protein 1 precursor	IGFBP1	P08833	1		1
IPI00465234	Cytokine receptor common beta chain precursor	CSF2RB	P32927	1		
IPI00010303	Serpin B4	SERPINB4	P48594	1		
IPI00022462	Transferrin receptor protein 1	TFRC	P02786	1	2	3
IPI00008091	Putative DNA helicase INO80 complex homolog 1	INOC1	Q9ULG1	1		
IPI00016112	peroxidasin homolog	PXDN		1		
IPI00010193	Isoform 1 of Interferon-alpha/beta receptor beta chain precursor	IFNAR2	P48551	1		2
IPI00329791	cDNA FLJ78679, highly similar to Homo sapiens DEAD (Asp-Glu-Ala-Asp) box polypeptide 46 (DDX46), mRNA	DDX46	Q7L014	1		
IPI00152344	Pyridoxal phosphate phosphatase PHOSPHO2	PHOSPHO2	Q8TCD6	1		
IPI00307612	Cadherin-20 precursor	CDH20	Q9HBT6	1	2	
IPI00465363	Histone H2B type 1-A	HIST1H2BA	Q96A08	1		1
IPI00028601	Putative metallothionein C20orf127	MT1P3	Q9BQN2	1		
IPI00011781	Ankyrin repeat-containing protein C20orf86 precursor	C20orf86	Q9BZ19	1	1	
IPI00419908	Uncharacterized protein GPR179	GPR179	Q6PRD1	1	1	2
IPI00479125	SLIT-ROBO Rho GTPase-activating protein 2	SRGAP2	O75044	1		
IPI00455852	Isoform 1 of Rho guanine nucleotide exchange factor 15	ARHGEF15	O94989	1		
IPI00174976	Isoform 1 of MAGUK p55 subfamily member 5	MPP5	Q8N3R9	1	1	1
IPI00550876	Protein maestro	MRO	Q9BYG7	1		
IPI00795481	Isoform 1 of Ly6/PLAUR domain-containing protein 1 precursor	LYPD1	Q8N2G4	1	1	1
IPI00064296	PRO0633	-		1		
IPI00002459	annexin VI isoform 2	ANXA6		1	1	
IPI00025311	Isoform 1 of Breast carcinoma-amplified sequence 1	BCAS1	O75363	1	1	
IPI00021048	Isoform 1 of Myoferlin	FER1L3	Q9NZM1	1	1	

Table S1.

Number of unique peptides identified

IPI	Protein name	Gene symbol	Swiss Prot ID	Normal	CFS	nPTLS
IPI00216651	Isoform 1 of Interleukin-28 receptor alpha chain precursor	IL28RA	Q8IU57	1		
IPI00216774	Cerebellin-2	CBLN2	Q8IUK8	1	1	
IPI00792945	38 kDa protein	CHFR		1		
IPI00027464	Calcineurin subunit B isoform 1	PPP3R1	P63098	1	1	
IPI00149375	Isoform 2 of Uncharacterized protein C11orf56	C11orf56	Q8N612	1		
IPI00025318	SH3 domain-binding glutamic acid-rich-like protein	SH3BGRL	O75368	1	5	2
IPI00015913	5,6-dihydroxyindole-2-carboxylic acid oxidase precursor	TYRP1	P17643	1		
IPI00383016	Immunglobulin light chain variable region (Fragment)	-		1	1	1
IPI00293530	C3a anaphylatoxin chemotactic receptor	C3AR1	Q16581	1	2	1
IPI00399180	Serine/threonine-protein kinase SBK1	SBK1	Q52WX2	1		
IPI00007512	Glutathione transferase omega-2	GSTO2	Q9H4Y5	1		
IPI00016576	Isoform 1 of Grainyhead-like protein 2 homolog	GRHL2	Q6ISB3	1		
IPI00290308	Tribbles homolog 1	TRIB1	Q96RU8	1		
IPI00005859	Keratin, type II cytoskeletal 75	KRT75	O95678	1		1
IPI00025622	AN1-type zinc finger protein 5	ZFAND5	O76080	1		
IPI00019901	Isoform 1 of Alpha-adducin	ADD1	P35611	1		
IPI00102808	Isoform 1 of Dual specificity protein phosphatase 19	DUSP19	Q8WTR2	1		
IPI00382486	Ig heavy chain V-III region NIE	-	P01770	1	2	3
IPI00073763	Semaphorin-4C precursor	SEMA4C	Q9C0C4	1	3	3
IPI00018208	Tetratricopeptide repeat protein 33	TTC33	Q6PID6	1		
IPI00166865	CDGSH iron sulfur domain-containing protein 2	CISD2	Q8N5K1	1		
IPI00004533	Kinesin-like protein KIF3B	KIF3B	O15066	1		
IPI00020019	Adiponectin precursor	ADIPOQ	Q15848	1	4	2
IPI00477468	RNA polymerase-associated protein CTR9 homolog	CTR9	Q6PD62	1		
IPI00005668	Aldo-keto reductase family 1 member C2	AKR1C2	P52895	1		
IPI00018098	Isoform 1 of Pre-mRNA-splicing factor 38B	PRPF38B	Q5VTL8	1		
IPI00026241	Bone marrow stromal antigen 2 precursor	BST2	Q10589	1		
IPI00243338	24 kDa protein	KRT23		1		
IPI00786937	similar to deleted in malignant brain tumors 1 isoform b precursor	LOC731940		1		
IPI00010863	Copper transport protein ATOX1	ATOX1	O00244	1	3	1
IPI00218918	Annexin A1	ANXA1	P04083	1		

Table S1.

Number of unique peptides identified

IPI	Protein name	Gene symbol	Swiss Prot ID	Normal	CFS	nPTLS
IPI00295577	Receptor-type tyrosine-protein phosphatase beta precursor	PTPRB	P23467	1		
IPI00027744	Isoform 1 of Mineralocorticoid receptor	NR3C2	P08235	1	1	
IPI00718806	arylhydrocarbon receptor repressor	AHRR		1		1
IPI00029372	Uncharacterized protein C4orf15	C4orf15	Q68CZ6	1		
IPI00029591	P-selectin glycoprotein ligand 1 precursor	SELPLG	Q14242	1	1	
IPI00024032	TBC1 domain family, member 29	TBC1D29		1	1	1
IPI00744226	Conserved hypothetical protein	-		1		
IPI00792229	20 kDa protein	TATDN1		1		
IPI00000144	Oxytocin-neurophysin 1 precursor	OXT	P01178	1	2	2
IPI00220271	Alcohol dehydrogenase	AKR1A1	P14550	1		
IPI00786893	similar to LYRIC/3D3	LOC730296		1		1
IPI00015954	GTP-binding protein SAR1a	SAR1A	Q9NR31	1		
IPI00099838	Isoform 1 of Lysophosphatidic acid phosphatase type 6 precursor	ACP6	Q9NPH0	1	2	1
IPI00397949	G protein-coupled receptor 56 isoform b	GPR56		1		2
IPI00027547	Dermcidin precursor	DCD	P81605	1		
IPI00306853	Carbohydrate sulfotransferase 3	CHST3	Q7LGC8	1		2
IPI00418735	hypothetical protein LOC400566	LOC400566		1		
IPI00019530	Tyrosine-protein kinase receptor Tie-1 precursor	TIE1	P35590	1	7	4
IPI00306413	Tubulin polymerization-promoting protein family member 3	TPPP3	Q9BW30	1		
IPI00018755	High mobility group protein 1-like 10	HMG1L10	Q9UGV6	1		
IPI00385543	Isoform 3 of UPF0469 protein KIAA0907	KIAA0907	Q7Z7F0	1		
IPI00456683	Isoform 3 of Transcription elongation factor SPT6	SUPT6H	Q7KZ85	1	2	
IPI00299076	Receptor-binding cancer antigen expressed on SiSo cells (Fragment)	EBAG9	O00559	1		
IPI00386393	CDNA FLJ13729 fis, clone PLACE3000121, weakly similar to VESICULAR TRAFFIC CONTROL PROTEIN SEC15	EXOC6B		1		
IPI00878511	45 kDa protein	-		1		
IPI00816155	Isoform 2 of Complement C1q-like protein 3 precursor	C1QL3	Q5VWW1	1	5	4
IPI00442564	CDNA FLJ26948 fis, clone RCT08241	-		1		
IPI00412408	Breast cancer type 2 susceptibility protein	BRCA2	P51587	1		
IPI00005614	Isoform Long of Spectrin beta chain, brain 1	SPTBN1	Q01082	1		
IPI00021831	cAMP-dependent protein kinase type I-alpha regulatory subunit	PRKAR1A	P10644	1		

Table S1.

Number of unique peptides identified

IPI	Protein name	Gene symbol	Swiss Prot ID	Normal	CFS	nPTLS
IPI00552578	Serum amyloid A protein precursor	SAA1	P02735	1		3
IPI00008422	Isoform 2 of SWI/SNF-related matrix-associated actin-dependent regulator of chromatin subfamily A containing DEAD/H box 1	SMARCAD1	Q9H4L7	1		
IPI00413778	Peptidyl-prolyl cis-trans isomerase	FKBP1A		1		1
IPI00007682	Vacuolar ATP synthase catalytic subunit A	ATP6V1A	P38606	1		
IPI00024502	Ubiquilin-4	UBQLN4	Q9NRR5	1		
IPI00746177	similar to Tubulin alpha-2 chain	LOC730222		1	1	
IPI00550792	Isoform 1 of Bridging integrator 2	BIN2	Q9UBW5	1		1
IPI00029175	Strumpellin	KIAA0196	Q12768	1		
IPI00045360	Capicua-like protein/double homeodomain 4 fusion protein	CIC	Q96RK0	1		
IPI00022314	Superoxide dismutase [Mn], mitochondrial precursor	SOD2	P04179	1	5	4
IPI00001755	Glypican-6 precursor	GPC6	Q9Y625	1	1	
IPI00749171	Conserved hypothetical protein	LOC340184		1		
IPI00033419	Protein Fem-1 homolog b	FEM1B		1		1
IPI00170635	Secreted and transmembrane protein 1 precursor	SECTM1	Q8WVN6	1	1	2
IPI00830018	Uncharacterized protein ENSP00000374807	-		1	2	3
IPI00298738	DNA-directed RNA polymerase, mitochondrial precursor	POLRMT		1		
IPI00026230	Heterogeneous nuclear ribonucleoprotein H2	HNRPH2	P55795	1	1	
IPI00009365	COX16-like protein C14orf112, mitochondrial precursor	C14orf112	Q9P0S2	1	1	
IPI00445278	CDNA FLJ44033 fis, clone TESTI4028062	-		1		
IPI00335541	Isoform 1 of Protein timeless homolog	TIMELESS	Q9UNS1	1		
IPI00644840	Hypothetical protein	LOC255783		1		1
IPI00301098	Uncharacterized protein C1orf187 precursor	C1orf187	Q8NBI3	1	2	1
IPI00009771	Lamin-B2	LMNB2		1		1
IPI00444842	CDNA FLJ45125 fis, clone BRAWH3036561	-		1		
IPI00023152	Isoform 1 of N-acetylated-alpha-linked acidic dipeptidase-like protein	NAALADL1	Q9UQQ1	1		
IPI00296727	Kinesin-like protein KIF2B	KIF2B	Q8N4N8	1		
IPI00005347	Zinc finger Ran-binding domain-containing protein 1	ZRANB1	Q9UGI0	1		

Table S1.

IPI	Protein name	Gene symbol	Swiss Prot ID	Number of unique peptides identified Normal	CFS	nPTLS
IPI00470913	RANBP2-like and GRIP domain containing 1	RGPD1	Q68DN6	1		
IPI00444331	Isoform 4 of Histone-lysine N-methyltransferase NSD3	WHSC1L1	Q9BZ95	1		
IPI00023407	Nck-associated protein 1-like	NCKAP1L	P55160	1		
IPI00306046	Isoform 1 of EGF-like repeat and discoidin I-like domain-containing protein 3 precursor	EDIL3	O43854	1		1
IPI00000265	Uncharacterized protein C10orf38 precursor	C10orf38	Q5VUB5	1	4	3
IPI00028600	Isoform 1 of Kallikrein-7 precursor	KLK7	P49862	1	1	2
IPI00301288	polydom	SVEP1		1		1
IPI00456635	Isoform 1 of Protein unc-13 homolog D	UNC13D	Q70J99	1		
IPI00445315	Protein FAM47C	FAM47C		1		
IPI00221178	Isoform 2 of Tumor protein D54	TPD52L2	O43399	1	1	1
IPI00065931	Isoform 2 of A-kinase anchor protein 13	AKAP13	Q12802	1		1
IPI00149044	Isoform 2 of Suppressor of hairy wing homolog 4	ZNF280D	Q6N043	1		
IPI00007834	Isoform 1 of Ankyrin-2	ANK2	Q01484	1		
IPI00003971	Isoform RTN1-A of Reticulon-1	RTN1	Q16799	1	1	1
IPI00746681	Similar to Bcl-2-related ovarian killer protein	-		1		
IPI00000977	Mitogen-activated protein kinase kinase kinase 11	MAP3K11	Q16584	1		
IPI00383808	Ig kappa chain V-IV region STH (Fragment)	-	P83593	1	3	3
IPI00871556	107 kDa protein	ZFYVE28		1		
IPI00746666	hypothetical protein	LOC728262		1		
IPI00003814	Isoform 1 of Dual specificity mitogen-activated protein kinase kinase 6	MAP2K6	P52564	1		
IPI00290854	A-kinase anchor protein 3	AKAP3	O75969	1		
IPI00301058	Vasodilator-stimulated phosphoprotein	VASP	P50552	1		
IPI00245940	immunoglobulin superfamily 5 like	IGSF5		1		
IPI00657699	Protein	MUC19		1		
IPI00290094	Splicing factor, arginine/serine-rich 8	SFRS8	Q12872	1		
IPI00413826	similar to H3 histone, family 3B	LOC644914		1	1	2
IPI00030706	Activator of 90 kDa heat shock protein ATPase homolog 1	AHSA1	O95433	1		
IPI00853516	dynein, axonemal, heavy chain 17	DNAH17		1		
IPI00410093	coiled-coil domain containing 69	CCDC69		1		
IPI00168404	Zinc finger and BTB domain containing 34	ZBTB34	Q8NCN2	1		

Table S1.

IPI	Protein name	Gene symbol	Swiss Prot ID	Number of unique peptides identified<br>Normal	CFS	nPTLS
IPI00644766	cDNA FLJ78048, highly similar to Homo sapiens torsin A interacting protein 1, mRNA	TOR1AIP1		1		
IPI00167154	Uncharacterized protein MAPKBP1	MAPKBP1		1		
IPI00871533	Uncharacterized protein C3orf48 (Fragment)	C3orf48		1		
IPI00877615	15 kDa protein	-		1		
IPI00030352	Isoform 2 of Growth inhibition and differentiation-related protein 88	C10orf28	Q7Z5L2	1		
IPI00384051	Uncharacterized protein PSME2	PSME2	Q9UL46	1		1
IPI00221080	Isoform 2 of Parathyroid hormone-related protein precursor	PTHLH	P12272	1	3	2
IPI00386575	Ig lambda chain V-I region EPS	-	P06888	1	1	1
IPI00006146	serum amyloid A2	SAA1		1		2
IPI00032338	kelch-like 20	KLHL20	Q9Y2M5	1		1
IPI00293679	Isoform 1 of Potassium voltage-gated channel subfamily KQT member 4	KCNQ4	P56696	1		
IPI00000190	CD81 antigen	CD81	P60033	1	3	4
IPI00152769	Isoform 1 of Trpc4-associated protein	TRPC4AP	Q8TEL6	1	1	
IPI00748682	Pheromone shutdown-related, TraB family protein	-		1		
IPI00019888	Succinate-semialdehyde dehydrogenase, mitochondrial precursor	ALDH5A1	P51649	1		
IPI00291987	Insulin-like growth factor-binding protein-like 1 precursor	IGFBPL1	Q8WX77	1	2	1
IPI00170766	Isoform 2 of Protein CASC5	CASC5	Q8NG31	1	1	
IPI00032597	RNA-binding motif protein, X-linked 2	RBMX2	Q9Y388	1		
IPI00783855	neighbor of BRCA1 gene 1	NBR1	Q14596	1		
IPI00746987	Ribosomal protein S1 family protein	-		1		
IPI00150881	Isoform 1 of Coiled-coil domain-containing protein C6orf204	C6orf204	Q5SZL2	1	1	2
IPI00295469	Copine-6	CPNE6	O95741	1		
IPI00044326	Carbohydrate sulfotransferase 14	CHST14	Q8NCH0	1	1	2
IPI00884004	Rheumatoid factor RF-ET12 (Fragment)	-		1	4	3
IPI00478997	V5-6 protein	IGLV4-69		1	1	1
IPI00456599	hypothetical protein LOC84792	MGC12966		1		
IPI00607831	PRAME family member 3	PRAMEF3	Q5TYW8	1		
IPI00399254	Isoform 1 of OTU domain-containing protein 4	OTUD4	Q01804	1		
IPI00171230	Isoform 2 of ELKS/RAB6-interacting/CAST family member 1	ERC1	Q8IUD2	1	1	1
IPI00719505	RABL2A protein	RABL2A		1		
IPI00784880	Cancer/testis antigen 75	LOC440934	Q6PK30	1		

Table S1.

Number of unique peptides identified

IPI	Protein name	Gene symbol	Swiss Prot ID	Normal	CFS	nPTLS
IPI00741005	similar to MAX-interacting protein isoform 4	MGA		1	1	
IPI00884353	Ets-1 transcript variant ets-1 delta	-		1		
IPI00852725	Isoform 7 of Prolactin receptor precursor	PRLR	P16471	1		
IPI00005600	Isoform 1 of Bifunctional heparan sulfate N-deacetylase/N-sulfotransferase 2	NDST2	P52849	1		
IPI00791593	8 kDa protein	GLYCAM1		1		
IPI00024346	snRNA-activating protein complex subunit 3	SNAPC3	Q92966	1		
IPI00293396	adaptor-related protein complex 1, gamma 1 subunit isoform a	AP1G1		1		
IPI00018914	Tyrosine-protein phosphatase non-receptor type 14	PTPN14	Q15678	1		
IPI00296913	ADP-sugar pyrophosphatase	NUDT5	Q9UKK9	1		
IPI00185146	Importin-9	IPO9	Q96P70	1		
IPI00018342	Adenylate kinase isoenzyme 1	AK1	P00568	1	3	
IPI00143753	Isoform 1 of U2-associated protein SR140	SR140	O15042	1		1
IPI00217791	Coiled-coil domain-containing protein 105	CCDC105	Q8IYK2	1		
IPI00166075	Leucine-rich repeat LGI family member 3 precursor	LGI3	Q8N145	1	1	2
IPI00167006	Uncharacterized protein C13orf26	C13orf26	Q8N6G2	1		
IPI00028481	Ras-related protein Rab-8A	RAB8A	P61006	1		
IPI00294215	Uncharacterized protein KIAA0232	KIAA0232	Q92628	1		
IPI00027806	Cysteine-rich secretory protein LCCL domain-containing 1 precursor	CRISPLD1	Q9H336	1		1
IPI00645194	integrin beta 1 isoform 1A precursor	ITGB1		1	1	1
IPI00071185	Isoform SV1 of PITSLRE serine/threonine-protein kinase CDC2L1	CDC2L1	P21127	1		
IPI00166776	Protein CREG2 precursor	CREG2	Q8IUH2	1		
IPI00853400	Isoform 1 of FK506-binding protein 15	FKBP15	Q5T1M5	1	1	
IPI00473033	Isoform 1 of Zinc finger protein 69	ZNF69	Q9UC07	1	1	1
IPI00749245	Secreted frizzled-related protein 1 precursor	SFRP1	Q8N474	1	1	1
IPI00152182	Isoform 1 of Kelch domain-containing protein 4	KLHDC4	Q8TBB5	1	1	
IPI00783604	EPH receptor A6 isoform a	EPHA6	Q9UF33	1	2	1
IPI00456969	Dynein heavy chain, cytosolic	DYNC1H1	Q14204	1		1
IPI00013299	Neuroblastoma, suppression of tumorigenicity 1	NBL1		1	11	12
IPI00001796	Tumor necrosis factor receptor superfamily, member 18 (Fragment)	TNFRSF18	Q9Y5U5	1		
IPI00549972	LIM domain-containing protein 2	LIMD2	Q9BT23	1		

Table S1.

IPI	Protein name	Gene symbol	Swiss Prot ID	Number of unique peptides identified<br>Normal	CFS	nPTLS
IPI00604551	Isoform 1 of Cell division cycle-associated protein 7	CDCA7	Q9BWT1	1		
IPI00029107	Werner syndrome ATP-dependent helicase	WRN	Q14191	1		
IPI00867509	Coronin-1C_i3 protein	CORO1C	Q9ULV4	1	1	
IPI00470468	Isoform 3 of Protein EFR3 homolog A	EFR3A	Q14156	1		
IPI00743963	Ig kappa chain V-I region HK101 precursor (Fragment)	IGKC	P01601	1	5	4
IPI00382515	CDNA FLJ30384 fis, clone BRACE2008114	-		1		
IPI00853312	Uncharacterized protein ENSP00000324580	-		1		
IPI00217871	Delta-1-pyrroline-5-carboxylate dehydrogenase, mitochondrial precursor	ALDH4A1	P30038	1		
IPI00552771	V2-11 protein	IGLV3-16		1	2	2
IPI00644191	70 kDa protein	ZNF90		1		1
IPI00470490	Isoform 1 of Nuclear receptor coactivator 1	NCOA1	Q15788	1		
IPI00410488	Isoform 1 of CD276 antigen precursor	CD276	Q5ZPR3	1		1
IPI00001433	Protocadherin beta 15 precursor	PCDHB15	Q9Y5E8	1		
IPI00220156	Isoform B of Transforming growth factor beta-2 precursor	TGFB2	P61812	1		
IPI00024818	Isoform 1 of Ubiquitin-specific peptidase-like protein 1	USPL1	Q5W0Q7	1		
IPI00335437	Ankyrin repeat and zinc finger domain-containing protein 1	ANKZF1	Q9H8Y5	1		
IPI00000076	Beta-nerve growth factor precursor	NGFB	P01138	1		
IPI00290078	keratin 4	KRT4	P19013	1	1	
IPI00100980	EH domain-containing protein 2	EHD2	Q9NZN4	1		
IPI00382474	Ig heavy chain V-III region TRO	-	P01762	1	1	2
IPI00215610	55 kDa erythrocyte membrane protein	MPP1	Q00013	1		
IPI00015148	Ras-related protein Rap-1b precursor	RAP1B	P61224	1		
IPI00020431	Isoform 1 of TGF-beta receptor type-2 precursor	TGFBR2	P37173	1	1	1
IPI00790122	27 kDa protein	TNFSF12		1	2	3
IPI00410657	Isoform 2 of mRNA cap guanine-N7 methyltransferase	RNMT	O43148	1		
IPI00328361	Seryl-tRNA synthetase, mitochondrial precursor	SARS2	Q9NP81	1		
IPI00552591	V1-20 protein	IGLV10-54		1		
IPI00022277	Coiled-coil domain-containing protein 56	CCDC56	Q9Y2R0	1		
IPI00216288	Isoform 3 of Lethal	L3MBTL	Q9Y468	1		

Table S1.

Number of unique peptides identified

IPI	Protein name	Gene symbol	Swiss Prot ID	Normal	CFS	nPTLS
IPI00025499	Isoform Tau-F of Microtubule-associated protein tau	MAPT	P10636	1		
IPI00030757	Isoform LpNPI of ADAMTS-2 precursor	ADAMTS2	O95450	1	3	2
IPI00787083	similar to peptidylprolyl isomerase A isoform 1	LOC256374		1		
IPI00856012	collagen type VI alpha 6	COL6A6		1		
IPI00001712	Isoform 1 of Catenin alpha-3	CTNNA3	Q9UI47	1		
IPI00026125	Deoxyribonuclease I-like 1 precursor	DNASE1L1	P49184	1	1	1
IPI00216592	Isoform C1 of Heterogeneous nuclear ribonucleoproteins C1/C2	HNRNPC	P07910	1	1	
IPI00217831	Ankyrin repeat domain-containing protein 13A	ANKRD13A	Q8IZ07	1		
IPI00335168	Isoform Non-muscle of Myosin light polypeptide 6	MYL6	P60660	1		
IPI00030037	Agouti-signaling protein precursor	ASIP	P42127	1		
IPI00013749	Protein kinase C zeta type	PRKCZ	Q05513	1		
IPI00002491	Isoform 9 of Sorbin and SH3 domain-containing protein 1	SORBS1	Q9BX66	1		
IPI00645078	Ubiquitin-like modifier-activating enzyme 1	UBA1	P22314	1	1	2
IPI00395866	SCUBE1 protein	SCUBE1		1	3	2
IPI00240793	Probable phospholipid-transporting ATPase IF	ATP11B	Q9Y2G3	1		
IPI00005159	Actin-related protein 2	ACTR2	P61160	1	1	
IPI00376237	Isoform 2 of Transcription factor LBX2	LBX2	Q6XYB7	1		
IPI00847723	Similar to VH4 heavy chain variable region precursor	-		1	1	3
IPI00169115	Olfactory receptor OR9-8	OR13C3	Q8NGS6	1		1
IPI00020966	Isoform 1 of Phosphatidylinositol N-acetylglucosaminyltransferase subunit A	PIGA	P37287	1		
IPI00456827	Protein FAM22G precursor	FAM22G	Q5VZR2	1		
IPI00747657	Similar to Rod cGMP-specific 3',5'-cyclic phosphodiesterase subunit beta precursor	PDE6B		1		
IPI00215777	Isoform B of Phosphate carrier protein, mitochondrial precursor	SLC25A3	Q00325	1		
IPI00028912	zinc finger protein 161	VEZF1	Q14119	1		
IPI00003807	Lysosomal acid phosphatase precursor	ACP2	P11117	1	2	1
IPI00830107	V4-2 protein	IGLV5-45		1		1
IPI00019242	Matrix metalloproteinase-15 precursor	MMP15	P51511	1		
IPI00647217	Superkiller viralicidic activity 2-like 2	SKIV2L2	P42285	1	1	
IPI00002818	Isoform 1 of Kallikrein-11 precursor	KLK11	Q9UBX7	1	5	4
IPI00017529	Isoform 1 of Lymphocyte function-associated antigen 3 precursor	CD58	P19256	1	1	

Table S1.

Number of unique peptides identified

IPI	Protein name	Gene symbol	Swiss Prot ID	Normal	CFS	nPTLS
IPI00024664	Isoform Long of Ubiquitin carboxyl-terminal hydrolase 5	USP5	P45974	1		
IPI00786946	similar to Tektin-3	LOC642249		1		
IPI00166257	CDNA FLJ37614 fis, clone BRCOC2011769	-		1		
IPI00004798	cDNA FLJ75207	CRISP3	P54108	1	2	3
IPI00012877	Isoform 1 of Interferon-alpha/beta receptor alpha chain precursor	IFNAR1	P17181	1		1
IPI00011592	Cytoplasmic dynein 1 light intermediate chain 2	DYNC1LI2	O43237	1	1	
IPI00442745	CDNA FLJ26780 fis, clone PRS03837	-		1		
IPI00478816	Serine protease inhibitor Kazal-type 5 precursor	SPINK5	Q9NQ38	1	1	1
IPI00017256	Ras suppressor protein 1	RSU1	Q15404	1		
IPI00374301	hypothetical protein	-		1		
IPI00023217	Isoform 1 of Ryanodine receptor 2	RYR2	Q92736	1		
IPI00025992	Hepcidin precursor	HAMP	P81172	1	1	1
IPI00024920	ATP synthase subunit delta, mitochondrial precursor	ATP5D	P30049	1		
IPI00026262	Isoform 1 of Ras GTPase-activating protein 1	RASA1	P20936	1		1
IPI00018843	Isoform 1 of D(3) dopamine receptor	DRD3	P35462	1		
IPI00477616	Protein phosphatase 2A activator, regulatory subunit 4	PPP2R4	Q15257	1		1
IPI00027782	Stromelysin-1 precursor	MMP3	P08254	1		
IPI00030009	Isoform A of Bifunctional 3'-phosphoadenosine 5'-phosphosulfate synthetase 2	PAPSS2	O95340	1		
IPI00479722	Proteasome activator complex subunit 1	PSME1	Q06323	1		
IPI00177878	Isoform 3 of Transmembrane protein 16D	TMEM16D	Q32M45	1		
IPI00104907	Isoform 1 of Uncharacterized potential DNA-binding protein C14orf106	C14orf106	Q6P0N0	1		
IPI00827978	VL4 protein (Fragment)	-		1	2	1
IPI00005107	Niemann-Pick C1 protein precursor	NPC1	O15118	1		
IPI00006470	Neuron-specific protein family member 2	HMP19	Q9Y328	1	1	
IPI00152216	Isoform 1 of Protein RIC-3 precursor	RIC3	Q7Z5B4	1	1	1
IPI00028520	Isoform 1 of NADH dehydrogenase [ubiquinone] flavoprotein 1, mitochondrial precursor	NDUFV1	P49821	1		
IPI00290358	Putative uncharacterized protein gs103	LOC283951		1	1	2
IPI00013272	Isoform 1 of Golgin subfamily A member 4	GOLGA4	Q13439	1	1	
IPI00009070	Isoform 1 of HBS1-like protein	HBS1L	Q9Y450	1		

Table S1.

Number of unique peptides identified

IPI	Protein name	Gene symbol	Swiss Prot ID	Normal	CFS	nPTLS
IPI00386576	Ig lambda chain V-IV region MOL	-	P06889	1		
IPI00022295	Platelet factor 4 variant precursor	PF4V1	P10720	1		1
IPI00747420	Melanoma-derived protein (Fragment)	-		1		
IPI00432226	AVLL5809	-		1		
IPI00171874	Ras guanyl-releasing protein 3	RASGRP3	Q8IV61	1		1
IPI00165229	proprotein convertase subtilisin/kexin type 5 preproprotein	PCSK5		1	1	
IPI00006746	Ermin	ERMN	Q8TAM6	1		
IPI00178894	Zinc finger and BTB domain-containing protein 20	ZBTB20	Q9HC78	1		
IPI00878436	24 kDa protein	SLC2A11		1		
IPI00640810	6 kDa protein	CTDP1		1		
IPI00845229	Isoform 2 of DEP domain-containing protein 2	DEPDC2	Q70Z35	1	1	
IPI00306549	CDNA FLJ11065 fis, clone PLACE1004868, weakly similar to MALE STERILITY PROTEIN 2	MLSTD1		1		
IPI00003392	Transmembrane protein 5	TMEM5	Q9Y2B1	1		
IPI00293361	Isoform 2 of Small G protein signaling modulator 2	SGSM2	O43147	1		
IPI00021753	Kinesin-like protein KIF13B	KIF13B	Q9NQT8	1		
IPI00827788	VH-3 family (VH26)D/J protein (Fragment)	-		1	2	1
IPI00253281	Isoform 2 of Epidermal growth factor receptor kinase substrate 8-like protein 1	EPS8L1	Q8TE68	1		
IPI00005607	Isoform 1 of Deleted in bladder cancer protein 1 precursor	DBC1	O60477	1		
IPI00027984	Putative uncharacterized protein	DNAJC11		1		
IPI00014371	Cadherin-18 precursor	CDH18	Q13634	1	1	1
IPI00329605	DNA mismatch repair protein Msh3	MSH3	P20585	1		1
IPI00032425	Receptor activity-modifying protein 3 precursor	RAMP3	O60896	1		
IPI00027875	Synaptotagmin-11	SYT11	Q9BT88	1	1	1
IPI00300020	Excitatory amino acid transporter 2	SLC1A2	P43004	1	1	1
IPI00001568	Vacuolar proton pump subunit D	ATP6V1D	Q9Y5K8	1		
IPI00221255	Isoform 2 of Myosin light chain kinase, smooth muscle	MYLK	Q15746	1	1	
IPI00300207	Isoform 1 of Uncharacterized protein FLJ44066	LOC91431	Q6ZU11	1	1	1
IPI00171678	Dopamine beta-hydroxylase	DBH	P09172	1	1	2
IPI00020470	Isoform 1 of Glycosyltransferase 8 domain-containing protein 1	GLT8D1	Q68CQ7	1		

Table S1.

Number of unique peptides identified

IPI	Protein name	Gene symbol	Swiss Prot ID	Normal	CFS	nPTLS
IPI00718821	Isoform 1 of Uncharacterized protein C19orf55	C19orf55	Q2NL68	1		
IPI00018387	Furin precursor	FURIN	P09958	1		
IPI00018909	trefoil factor 3 precursor	TFF3	Q07654	1	1	1
IPI00398229	similar to deubiquitinating enzyme 3	LOC392197		1		
IPI00027248	Tumor suppressor candidate 2	TUSC2	O75896	1		
IPI00163851	Isoform 1 of Eukaryotic translation initiation factor 2-alpha kinase 4	EIF2AK4	Q9P2K8	1		
IPI00438286	Isoform 1 of Protein LAP2	ERBB2IP	Q96RT1	1		2
IPI00023780	Isoform 2 of DnaJ homolog subfamily C member 5	DNAJC5	Q9H3Z4	1		
IPI00018027	Isoform 1 of Angiogenic factor with G patch and FHA domains 1	AGGF1	Q8N302	1		
IPI00010442	Phospholemman precursor	FXYD1	O00168	1		
IPI00434711	Putative uncharacterized protein FP6679	-		1		
IPI00009881	Neuroendocrine secretory protein 55	GNAS	O95467	1	1	1
IPI00020329	Potassium voltage-gated channel subfamily S member 2	KCNS2	Q9ULS6	1		1
IPI00243451	Liver-specific organic anion transporter 3TM12	SLCO1B3		1	1	1
IPI00184650	Class B basic helix-loop-helix protein 4	BHLHB4	Q8NDY6	1		
IPI00328298	Isoform 2 of Structural maintenance of chromosomes protein 4	SMC4	Q9NTJ3	1		
IPI00014340	Isoform 1 of Protein phosphatase 1 regulatory subunit 12C	PPP1R12C	Q9BZL4	1		
IPI00386284	olfactory receptor, family 2, subfamily AK, member 2	OR2AK2	Q8NG84	1		
IPI00289329	Ephrin type-B receptor 3 precursor	EPHB3	P54753	1	2	3
IPI00018429	Paired mesoderm homeobox protein 2	PRRX2	Q99811	1		
IPI00297550	Coagulation factor XIII A chain precursor	F13A1	P00488	1	1	
IPI00032904	Beta-synuclein	SNCB	Q16143	1	2	4
IPI00424119	Frizzled-3 precursor	FZD3	Q9NPG1	1	1	1
IPI00387004	PNAS-146	MCM7		1		
IPI00218946	Potassium/sodium hyperpolarization-activated cyclic nucleotide-gated channel 2	HCN2	Q9UL51	1	1	1
IPI00024766	Plexin-C1 precursor	PLXNC1	O60486	1	1	1
IPI00009471	WD repeat-containing protein 3	WDR3	Q9UNX4	1		
IPI00455967	Uncharacterized protein ENSP00000353619 (Fragment)	-		1		1
IPI00299263	ADP-ribosylation factor GTPase-activating protein 3	ARFGAP3	Q9NP61	1		
IPI00186966	Isoform IIA of Myc box-dependent-interacting protein 1	BIN1	O00499	1		

Table S1.

IPI	Protein name	Gene symbol	Swiss Prot ID	Number of unique peptides identified<br>Normal	CFS	nPTLS
IPI00045939	2-aminoethanethiol dioxygenase	ADO	Q96SZ5	1		
IPI00014537	Isoform 1 of Calumenin precursor	CALU	O43852	1		
IPI00165009	Isoform 3 of MBT domain-containing protein 1	MBTD1	Q05BQ5	1		
IPI00374670	hypothetical protein isoform 2	LOC388588		1		
IPI00289837	Coiled-coil domain-containing protein 85A	CCDC85A	Q96PX6	1		
IPI00027264	Calretinin	CALB2	P22676	1	1	1
IPI00878962	10 kDa protein	-		1		
IPI00103891	Putative uncharacterized protein	MTMR14		1		
IPI00328709	Gremlin-2 precursor	GREM2	Q9H772	1		1
IPI00398154	actin filament associated protein 1	AFAP1		1		
IPI00291939	Structural maintenance of chromosomes protein 1A	SMC1A	Q14683	1		
IPI00444272	Leukemia inhibitory factor receptor precursor	LIFR	P42702	1		1
IPI00641181	MARCKS-related protein	MARCKSL1	P49006	1		
IPI00418125	Isoform 1 of Layilin precursor	LAYN	Q6UX15	1	3	3
IPI00151990	Isoform 1 of Thioredoxin domain-containing protein 15 precursor	TXNDC15	Q96J42	1		
IPI00243995	Serine/threonine-protein kinase Nek5	NEK5	Q6P3R8	1	1	1
IPI00171196	keratin 13 isoform b	KRT13		1	4	2
IPI00021327	Isoform 1 of Growth factor receptor-bound protein 2	GRB2	P62993	1	2	6
IPI00024272	Integral membrane protein DGCR2/IDD precursor	DGCR2	P98153	1	6	9
IPI00023505	Low affinity immunoglobulin gamma Fc region receptor II-a precursor	FCGR2A	P12318	1	5	3
IPI00003384	Isoform 1 of Cadherin EGF LAG seven-pass G-type receptor 1 precursor	CELSR1	Q9NYQ6	1		
IPI00015988	HLA class I histocompatibility antigen, alpha chain G precursor	HLA-G	P17693	1		1
IPI00021812	Neuroblast differentiation-associated protein AHNAK	AHNAK	Q09666	1	1	1
IPI00219077	Isoform 1 of Leukotriene A-4 hydrolase	LTA4H	P09960	1	2	2
IPI00657936	collagen, type XXVIII precursor	COL28A1		1	3	3
IPI00004084	Isoform 2 of Cyclic AMP-dependent transcription factor ATF-6 beta	CREBL1	Q99941	1	1	1
IPI00642259	Dystonin	DST		1	2	
IPI00296259	Transmembrane emp24 domain-containing protein 4 precursor	TMED4	Q7Z7H5	1	3	1
IPI00789398	Isoform 3 of Lymphocyte antigen 75 precursor	LY75	O60449	1	1	2
IPI00032826	Hsc70-interacting protein	ST13	P50502	1		1

Table S1.

Number of unique peptides identified

IPI	Protein name	Gene symbol	Swiss Prot ID	Normal	CFS	nPTLS
IPI00152326	glutathione S-transferase M1 isoform 2	GSTM1		1		1
IPI00044751	Isoform 1 of M-phase phosphoprotein 1	MPHOSPH1	Q96Q89	1		1
IPI00329593	Isoform 2 of ADP-dependent glucokinase	ADPGK	Q9BRR6	1		2
IPI00296461	Isoform 1 of Sphingomyelin phosphodiesterase precursor	SMPD1	P17405	1		1
IPI00009943	Tumor protein, translationally-controlled 1	TPT1		1	1	1
IPI00376383	Centrosomal protein 110kDa	CEP110		1		
IPI00015973	Band 4.1-like protein 2	EPB41L2	O43491	1		
IPI00166807	Isoform 3 of Oxidation resistance protein 1	OXR1	Q8N573	1		1
IPI00304789	27 kDa protein	TTLL9		1	1	
IPI00032187	nischarin	NISCH		1		1
IPI00792759	73 kDa protein	THSD4		1	1	
IPI00446588	Isoform 2 of Plexin-A4 precursor	PLXNA4	Q9HCM2	1	1	
IPI00419836	Isoform 1 of Discoidin, CUB and LCCL domain-containing protein 2 precursor	DCBLD2	Q96PD2	1	1	
IPI00420014	Isoform 1 of U5 small nuclear ribonucleoprotein 200 kDa helicase	ASCC3L1	O75643	1		
IPI00386314	FLJ00064 protein (Fragment)	CNDP2		1		
IPI00024107	Isoform 1 of Alpha-synuclein	SNCA	P37840	1	2	2
IPI00217435	Signal peptide, CUB and EGF-like domain-containing protein 1 precursor	SCUBE1	Q8IWY4	1	2	2
IPI00021766	Isoform 1 of Reticulon-4	RTN4	Q9NQC3	1		
IPI00221006	Isoform 4 of Transcription factor 7-like 2	TCF7L2	Q9NQB0	1		1
IPI00005567	Isoform 3A of Voltage-dependent L-type calcium channel subunit beta-3	CACNB3	P54284		1	
IPI00852669	Zinc finger protein 516	ZNF516	Q92618			1
IPI00017297	Matrin-3	MATR3	P43243			1
IPI00185038	Isoform 1 of Dual oxidase 1 precursor	DUOX1	Q9NRD9		1	
IPI00385799	erv1-like growth factor	GFER			2	1
IPI00470812	Putative uncharacterized protein DKFZp781E21107	LOC339977				1
IPI00556381	Putative uncharacterized protein (Fragment)	TRIM16				1
IPI00004569	Isoform MZF1A of Myeloid zinc finger 1	MZF1	P28698			1
IPI00005537	39S ribosomal protein L12, mitochondrial precursor	MRPL12	P52815		1	
IPI00240059	Transmembrane and coiled-coil domains protein 3	TMCC3	Q9ULS5			1
IPI00795575	39 kDa protein	-			1	
IPI00221240	Isoform 2 of Leucyl-cystinyl aminopeptidase	LNPEP	Q9UIQ6		1	

Table S1.

IPI	Protein name	Gene symbol	Swiss Prot ID	Number of unique peptides identified		
				Normal	CFS	nPTLS
IPI00655865	Isoform 2 of Protein diaphanous homolog 3	DIAPH3	Q9NSV4		1	
IPI00442474	CDNA FLJ27204 fis, clone SYN03230	COG7			1	1
IPI00019270	Tankyrase-2	TNKS2	Q9H2K2		1	
IPI00004838	Isoform Crk-II of Proto-oncogene C-crk	CRK	P46108			1
IPI00026646	IgG receptor FcRn large subunit p51 precursor (Fragment)	FCGRT	P55899		1	2
IPI00748733	Similar to Slit homolog 3 protein precursor	-				1
IPI00023919	26S protease regulatory subunit 8	PSMC5	P62195			1
IPI00010277	Isoform 1 of Tumor necrosis factor receptor superfamily member 12A precursor	TNFRSF12A	Q9NP84		1	2
IPI00302614	V-set domain containing T cell activation inhibitor 1	VTCN1			1	
IPI00007423	Isoform 1 of Acidic leucine-rich nuclear phosphoprotein 32 family member B	ANP32B	Q92688			1
IPI00553215	V1-5 protein	IGLV2-18				2
IPI00020005	Arylsulfatase E precursor	ARSE	P51690			1
IPI00176976	Novel protein similar to mitochondrial ribosome recycling factor isoform 1	RP1-57A13.2			1	
IPI00786931	similar to fatty acid amide hydrolase	LOC729037			1	
IPI00015181	Integrin alpha-9 precursor	ITGA9	Q13797			1
IPI00387100	Ig kappa chain V-I region Roy	-	P01608		2	3
IPI00063878	Isoform 1 of Multidrug resistance-associated protein 9	ABCC12	Q96J65		1	
IPI00029473	Protein Njmu-R1	C17orf75	Q9HAS0		1	
IPI00847415	Isoform 4 of Dual specificity protein kinase CLK3	CLK3	P49761			1
IPI00884076	ENV polyprotein (coat polyprotein) family protein	-			1	
IPI00719690	Isoform 1 of Sterile alpha motif domain-containing protein 9-like	SAMD9L	Q8IVG5		1	1
IPI00064429	Isoform 2 of Inactive serine protease RAMP precursor	DKFZP586H2123	Q6UXH9		2	1
IPI00449923	Isoform 1 of Retinoic acid-induced protein 1	RAI1	Q7Z5J4		1	
IPI00294187	Protein-arginine deiminase type-2	PADI2	Q9Y2J8		1	
IPI00045473	Similar to C. Elegans protein F17C8.5	LOC339123			1	
IPI00167433	CDNA FLJ39703 fis, clone SMINT2012195	FDX1L			1	
IPI00063245	Uncharacterized protein FUBP3	FUBP3	Q96I24			1
IPI00017480	Sonic hedgehog protein precursor	SHH	Q15465		2	
IPI00012079	Eukaryotic translation initiation factor 4B	EIF4B	P23588			1

Table S1.

Number of unique peptides identified

IPI	Protein name	Gene symbol	Swiss Prot ID	Normal	CFS	nPTLS
IPI00183572	Isoform 2 of Dedicator of cytokinesis protein 7	DOCK7	Q96N67		1	
IPI00001835	Zinc finger and BTB domain-containing protein 4	ZBTB4	Q9P1Z0		1	
IPI00148768	TRIO and F-actin binding protein isoform 1	TRIOBP	Q9H2D6		1	
IPI00170548	Isoform 1 of ATPase family AAA domain-containing protein 2	ATAD2	Q6PL18		1	
IPI00024134	Ig kappa chain V-I region Walker precursor	IGKC	P04431		3	3
IPI00005793	AP-3 complex subunit beta-2	AP3B2	Q13367			1
IPI00410391	Isoform 1 of Major facilitator superfamily domain-containing protein 2	MFSD2	Q8NA29			1
IPI00220257	Isoform 3 of Probable tubulin polyglutamylase	TTLL1	O95922		1	
IPI00441952	Isoform 1 of Ninein	NIN	Q8N4C6			1
IPI00016940	Phospholipid scramblase 2	PLSCR2	Q9NRY7			1
IPI00298058	Isoform 1 of Transcription elongation factor SPT5	SUPT5H	O00267		1	
IPI00829915	similar to PR domain zinc finger protein 16	LOC647868				1
IPI00023843	Deoxyribonuclease I-like 2 precursor	DNASE1L2	Q92874		1	1
IPI00455731	Protein FAM26F	FAM26F	Q5R3K3			1
IPI00102281	Retroviral-like aspartic protease 1 precursor	SASP	Q53RT3		1	
IPI00030355	Protein phosphatase 1 regulatory subunit 11	PPP1R11	O60927		1	
IPI00002649	Isoform 2 of Pinin	PNN	Q9H307		1	2
IPI00217915	Isoform 1 of Transmembrane protein 136	TMEM136	Q6ZRR5		1	
IPI00414927	Transmembrane and immunoglobulin domain-containing protein C1orf32	C1orf32	Q71H61			1
IPI00871458	39 kDa protein	C10orf4				1
IPI00101299	C1GALT1-specific chaperone 1	C1GALT1C1	Q96EU7			1
IPI00176778	KIAA1841 protein	KIAA1841			1	1
IPI00220986	Isoform 3 of ADAMTS-9 precursor	ADAMTS9	Q9P2N4		1	
IPI00175169	Isoform 1 of ADP-ribosylation factor GTPase-activating protein 1	ARFGAP1	Q8N6T3		1	
IPI00760941	Uncharacterized protein ENSP00000371687	DKFZp434J1015				1
IPI00514966	Recombining binding protein suppressor of hairless	RBPJL				1
IPI00550862	hypothetical protein LOC387911	LOC387911			1	
IPI00746459	CDNA FLJ34517 fis, clone HLUNG2006781	-			1	

Table S1.

Number of unique peptides identified

IPI	Protein name	Gene symbol	Swiss Prot ID	Normal	CFS	nPTLS
IPI00444234	CDNA FLJ45743 fis, clone KIDNE2016464	FLJ45743			1	
IPI00412982	Neurogenic locus notch homolog protein 1 precursor	NOTCH1	P46531		2	2
IPI00021772	S-adenosylmethionine synthetase isoform type-1	MAT1A	Q00266			1
IPI00002534	Isoform 1 of Purine-rich element-binding protein gamma	PURG	Q9UJV8		1	
IPI00556253	Isoform 1 of Coiled-coil domain-containing protein C6orf199	C6orf199	Q5TCS8		1	
IPI00017592	Leucine zipper-EF-hand-containing transmembrane protein 1, mitochondrial precursor	LETM1	O95202		1	
IPI00016007	Myosin-Vc	MYO5C	Q9NQX4		1	
IPI00216969	Isoform IA of Proto-oncogene tyrosine-protein kinase ABL1	ABL1	P00519		1	1
IPI00830059	Uncharacterized protein ENSP00000375013	-			1	1
IPI00240909	similar to eukaryotic translation initiation factor 3, subunit 5 epsilon, 47kDa isoform 1	hCG_15200				1
IPI00004671	Golgin subfamily B member 1	GOLGB1	Q14789			2
IPI00744927	gastrotropin isoform 1	FABP6			1	
IPI00024319	Forkhead box protein J1	FOXJ1	Q92949		1	
IPI00217899	RING finger protein 168	RNF168	Q8IYW5			1
IPI00339297	Polypeptide N-acetylgalactosaminyltransferase 4	GALNT4	Q8N4A0		1	
IPI00451450	Inactive serine protease 35 precursor	PRSS35	Q8N3Z0		1	1
IPI00152462	Ciliary dynein heavy chain 3	DNAH3				1
IPI00254408	bromodomain PHD finger transcription factor isoform 1	BPTF	Q12830		1	
IPI00304324	Histone deacetylase 11	HDAC11	Q96DB2		1	
IPI00477693	fatty acid binding protein	LOC646486			1	
IPI00004509	Leukotriene C4 synthase	LTC4S	Q16873			1
IPI00013418	Baculoviral IAP repeat-containing protein 2	BIRC2	Q13490		1	1
IPI00884371	Uncharacterized protein ENSP00000383215	-				1
IPI00002881	Bcl-2-related protein A1	BCL2A1	Q16548			1
IPI00063523	similar to Temporarily Assigned Gene name family member	FLJ40176			1	1
IPI00853133	DEAD (Asp-Glu-Ala-Asp) box polypeptide 60-like	DDX60L			2	

Table S1.

Number of unique peptides identified

IPI	Protein name	Gene symbol	Swiss Prot ID	Normal	CFS	nPTLS
IPI00065352	Isoform 1 of Uncharacterized serine/threonine-protein kinase SgK494	FLJ25006	Q96LW2			1
IPI00382424	Ig lambda chain V-II region NEI	-	P01705		1	2
IPI00060423	Isoform 1 of Collagen triple helix repeat-containing protein 1 precursor	CTHRC1	Q96CG8		1	1
IPI00000388	Zinc finger protein 175	ZNF175	Q9Y473			1
IPI00240345	C-type lectin domain family 14 member A precursor	CLEC14A	Q86T13		1	1
IPI00447328	Isoform 5 of Coiled-coil domain-containing protein 40	CCDC40	Q4G0X9		1	1
IPI00394807	Regenerating islet-derived protein 3 gamma precursor	REG3G	Q6UW15			1
IPI00022830	Isoform 2 of NSFL1 cofactor p47	NSFL1C	Q9UNZ2			1
IPI00025310	Zinc finger protein 217	ZNF217	O75362		1	
IPI00334845	Isoform 3 of Uncharacterized protein KIAA1946 precursor	KIAA1946	Q6P995		1	1
IPI00008816	Isoform 1 of DNA repair protein REV1	REV1	Q9UBZ9		1	1
IPI00465345	Isoform 3 of Pre-mRNA-processing factor 40 homolog B	PRPF40B	Q6NWY9			2
IPI00427808	Isoform 1 of Uncharacterized protein C10orf71	C10orf71	Q711Q0		1	
IPI00065388	Isoform 1 of Coiled-coil domain-containing protein 17	CCDC17	Q96LX7			1
IPI00735313	similar to Peptidyl-prolyl cis-trans isomerase NIMA-interacting 4	-			1	
IPI00384013	Polypeptide N-acetylgalactosaminyltransferase-like protein 2	GALNTL2	Q8N3T1		1	
IPI00410631	Protein FAM45A	FAM45A	Q8TCE6		1	1
IPI00289540	Isoform 2 of Usherin precursor	USH2A	O75445			1
IPI00382495	Ig heavy chain V-III region ZAP	-	P01778		1	
IPI00748216	CDNA FLJ20187 fis, clone COLF0433	GTF3C5				1
IPI00001674	Isoform 1 of Neurokinin-B precursor	TAC3	Q9UHF0		2	1
IPI00477820	Isoform 1 of FRAS1-related extracellular matrix protein 1 precursor	FREM1	Q5H8C1		1	
IPI00024776	Calmegin precursor	CLGN	O14967		1	
IPI00022143	Isoform 1 of Extended-synaptotagmin-1	FAM62A	Q9BSJ8			1
IPI00023663	Putative transcription factor Ovo-like 1	OVOL1	O14753			1
IPI00015602	Mitochondrial precursor proteins import receptor	TOMM70A	O94826			1
IPI00072377	Isoform 1 of Protein SET	SET	Q01105			1
IPI00011604	Glycine cleavage system H protein, mitochondrial precursor	GCSH	P23434			1
IPI00401586	Chromosome 18 open reading frame 62	C18orf62				1

Table S1.

IPI	Protein name	Gene symbol	Swiss Prot ID	Normal	CFS	nPTLS
				Number of unique peptides identified		
IPI00012760	Leptin precursor	LEP	P41159		1	1
IPI00023110	Potassium channel subfamily K member 3	KCNK3	O14649		1	
IPI00182833	Coiled-coil alpha-helical rod protein 1	CCHCR1	Q8TD31		1	
IPI00167280	CDNA FLJ40447 fis, clone TESTI2040642	FLJ25439			1	1
IPI00026492	2-amino-3-ketobutyrate coenzyme A ligase, mitochondrial precursor	GCAT	O75600			1
IPI00394994	Isoform 1 of Nesprin-3	C14orf49	Q6ZMZ3		1	
IPI00644529	Isoform 2 of Zinc finger protein 615	ZNF615	Q8N8J6			1
IPI00028880	Putative DNA polymerase	POLN				1
IPI00014878	Uncharacterized protein PRKD1	PRKD1				1
IPI00794791	Isoform 2 of IQ domain-containing protein D	IQCD	Q96DY2			1
IPI00009853	Vomeronasal type-1 receptor 1	VN1R1	Q9GZP7			1
IPI00022250	Lymphocyte antigen 96 precursor	LY96	Q9Y6Y9			1
IPI00332371	Isoform 1 of 6-phosphofructokinase, liver type	PFKL	P17858			1
IPI00004337	Zinc finger and BTB domain-containing protein 11	ZBTB11	O95625			1
IPI00419992	Isoform 1 of Acyl-CoA synthetase family member 4	AASDH	Q4L235		1	
IPI00004445	Gamma-2-syntrophin	SNTG2	Q9NY99		1	
IPI00878015	12 kDa protein	-			1	
IPI00455927	Uncharacterized protein ENSP00000366638 (Fragment)	-			2	2
IPI00328094	Isoform 1 of Forkhead box protein P3	FOXP3	Q9BZS1		1	
IPI00873348	63 kDa protein	A1CF			1	
IPI00005708	Heparan sulfate glucosamine 3-O-sulfotransferase 3B1	HS3ST3B1	Q9Y662			1
IPI00169430	Isoform 1 of Spermatid perinuclear RNA-binding protein	STRBP	Q96SI9			1
IPI00011092	Ubiquitin carboxyl-terminal hydrolase 26	USP26	Q9BXU7		1	1
IPI00007057	Rabenosyn-5	ZFYVE20	Q9H1K0			1
IPI00307016	Metallothionein 1 pseudogene 2	MT1P2			1	1
IPI00386822	Putative uncharacterized protein (Fragment)	TGFBI				1
IPI00175649	Leucine-rich repeat serine/threonine-protein kinase 2	LRRK2	Q5S007		1	1
IPI00003894	RING finger protein 11	RNF11	Q9Y3C5		1	1
IPI00015614	Isoform A of Trypsin-3 precursor	PRSS3	P35030		7	6
IPI00009524	Isoform Alpha of Tripartite motif-containing protein 10	TRIM10	Q9UDY6		1	1
IPI00477539	Uncharacterized protein C14orf102	C14orf102	Q9H7Z3			1

Table S1.

Number of unique peptides identified

IPI	Protein name	Gene symbol	Swiss Prot ID	Normal	CFS	nPTLS
IPI00640240	59 kDa protein	SPTLC3			1	
IPI00002824	Cysteine and glycine-rich protein 2	CSRP2	Q16527		1	
IPI00398272	Isoform 1 of Collagen alpha-1(XVII) chain	COL17A1	Q9UMD9		1	
IPI00103595	Centrosome-associated protein 350	CEP350	Q5VT06		3	
IPI00006648	IDN4-GGTR14 protein	RABGAP1L				1
IPI00220002	Isoform 2 of Paralemmin	PALM	O75781		1	1
IPI00302176	Isoform 1 of H/ACA ribonucleoprotein complex subunit 1	NOLA1	Q9NY12			1
IPI00003564	Lymphocyte antigen 86 precursor	LY86	O95711		1	
IPI00061009	Isoform 1 of WD repeat-containing protein 67	WDR67	Q96DN5		1	
IPI00299301	Isoform 1 of Desmuslin	DMN	O15061		1	
IPI00152011	PTPL1-associated RhoGAP 1	ARHGAP29			1	
IPI00478586	Isoform 2 of Vacuolar protein sorting-associated protein 13A	VPS13A	Q96RL7			1
IPI00060439	KIAA0226 protein	KIAA0226				1
IPI00847790	Protein of unknown function DUF1725 domain containing protein	-			1	
IPI00303343	Splicing factor, arginine/serine-rich 19	SCAF1	Q9H7N4			1
IPI00065121	Isoform 2 of Coiled-coil domain-containing protein 36	CCDC36	Q8IYA8		1	
IPI00373867	glycolipid transfer protein domain containing 2	GLTPD2			1	
IPI00419180	CDNA FLJ41429 fis, clone BRHIP2005354	CC2D2B			1	
IPI00872354	armadillo repeat containing 2	ARMC2	Q8NEN0		1	
IPI00869070	Ankyrin repeat containing protein	-				1
IPI00783186	Isoform 2 of Ankyrin repeat domain-containing protein 17	ANKRD17	O75179			1
IPI00152975	Isoform 1 of Coiled-coil domain-containing protein 90A, mitochondrial precursor	CCDC90A	Q96AQ8		1	
IPI00303152	collagen, type XXII, alpha 1	COL22A1				1
IPI00374804	Isoform 1 of Diacylglycerol kinase delta	DGKD	Q16760		1	
IPI00292894	Pre-rRNA-processing protein TSR1 homolog	TSR1	Q2NL82		1	
IPI00028232	Putative uncharacterized protein	FGF4			1	
IPI00423379	Putative uncharacterized protein DKFZp686G24192	C20orf96				1
IPI00027269	E3 ubiquitin-protein ligase CBL	CBL	P22681		1	
IPI00025308	Homeobox protein prophet of Pit-1	PROP1	O75360			1
IPI00219358	Isoform 1 of Mannose-6-phosphate isomerase	MPI	P34949			1
IPI00797567	9 kDa protein	GNAI2			1	

Table S1.

IPI	Protein name	Gene symbol	Swiss Prot ID	Number of unique peptides identified<br>Normal	CFS	nPTLS
IPI00307757	Isoform 1 of Membrane-associated phosphatidylinositol transfer protein 3	PITPNM3	Q9BZ71			1
IPI00032958	Isoform 2 of Actin-binding protein anillin	ANLN	Q9NQW6		1	
IPI00166738	Zinc-binding alcohol dehydrogenase domain-containing protein 2	ZADH2	Q8N4Q0			1
IPI00412977	Prothymosin alpha	-				3
IPI00457109	Isoform 2 of ATP-binding cassette sub-family A member 12	ABCA12	Q86UK0		1	2
IPI00446355	CDNA FLJ41803 fis, clone NHNPC2002749	IER5L				1
IPI00167861	Protein SMG5	SMG5	Q9UPR3			1
IPI00005530	FMRFamide-related peptides precursor	NPVF	Q9HCQ7			1
IPI00387132	CDNA FLJ32661 fis, clone TESTI1000055, weakly similar to HOMEOBOX PROTEIN SIX1	hCG_2007354			2	
IPI00057815	intestinal facilitative glucose transporter 7	SLC2A7			1	
IPI00472171	RPL7 protein	RPL7				1
IPI00398007	ubiquitin specific protease 40	USP40	Q9NVE5		1	1
IPI00187091	Isoform 2 of MAP kinase-interacting serine/threonine-protein kinase 1	MKNK1	Q9BUB5			1
IPI00292471	Centaurin-delta-1	CENTD1	Q8WZ64		1	1
IPI00014367	Isoform 1 of Non-SMC element 4 homolog A	NSMCE4A	Q9NXX6		1	
IPI00398421	Isoform 1 of Potassium channel subfamily T member 2	KCNT2	Q6UVM3			1
IPI00884022	Similar to Immunglobulin heavy chain variable region	-			1	
IPI00878369	12 kDa protein	-			2	
IPI00465319	Chondroitin sulfate synthase 2	CHPF	Q8IZ52		1	
IPI00549844	Isoform 1 of Protein FAM134B	FAM134B	Q9H6L5		1	
IPI00027422	Isoform Beta-4C of Integrin beta-4 precursor	ITGB4	P16144		2	2
IPI00178150	Isoform 1 of Chromosome-associated kinesin KIF4A	KIF4A	O95239		1	3
IPI00457114	Isoform 1 of IQ motif and Sec7 domain-containing protein 1	IQSEC1	Q6DN90		1	
IPI00002773	Isoform 2 of Tyrosine-protein kinase JAK3	JAK3	P52333		1	
IPI00005667	NEDD4-binding protein 1	N4BP1	O75113		1	
IPI00232571	Glypican-4 precursor	GPC4	O75487		4	3
IPI00300594	CDNA FLJ35435 fis, clone SMINT2002620	SPATA13			1	
IPI00103142	NudC domain-containing protein 2	NUDCD2	Q8WVJ2		1	

Table S1.

Number of unique peptides identified

IPI	Protein name	Gene symbol	Swiss Prot ID	Normal	CFS	nPTLS
IPI00022228	Vigilin	HDLBP	Q00341			1
IPI00290785	Isoform 1 of SET domain-containing protein 6	SETD6	Q8TBK2		1	
IPI00382492	Ig heavy chain V-III region LAY	-	P01775		2	
IPI00185892	Isoform 1 of Actin-binding LIM protein 3	ABLIM3	O94929		1	1
IPI00297763	Retinal-specific ATP-binding cassette transporter	ABCA4	P78363		1	
IPI00000861	Isoform 1 of LIM and SH3 domain protein 1	LASP1	Q14847		1	
IPI00168255	hypothetical protein LOC400451	LOC400451			1	
IPI00159652	Isoform 2 of Protein furry homolog-like	FRYL	O94915		1	1
IPI00034277	Isoform A of Probable cation-transporting ATPase 13A1	ATP13A1	Q9HD20			1
IPI00152853	Isoform 1 of Phostensin	KIAA1949	Q6NYC8			1
IPI00015990	Isoform 1 of Eyes absent homolog 3	EYA3	Q99504		1	
IPI00015802	Isoform 1 of Zinc finger protein ZFPM2	ZFPM2	Q8WW38		1	1
IPI00334492	Isoform 3 of Coiled-coil domain-containing protein 144A	LOC731479	A2RUR9		2	
IPI00446785	CDNA FLJ41116 fis, clone BRACE1000572	-				1
IPI00784272	Putative uncharacterized protein	-				1
IPI00171500	Uncharacterized protein C14orf45	C14orf45	Q8ND07		1	
IPI00411452	Uncharacterized protein DOCK11	DOCK11				1
IPI00170765	Isoform 3 of Alanine--glyoxylate aminotransferase 2-like 2	AGXT2L2	Q8IUZ5		1	
IPI00099977	platelet-derived growth factor C precursor	PDGFC			1	
IPI00444395	CDNA FLJ45615 fis, clone BRTHA3026180	-				1
IPI00019278	Thiamine transporter 2	SLC19A3	Q9BZV2			1
IPI00292953	Isoform 2 of Ankycorbin	RAI14	Q9P0K7		1	
IPI00159072	57 kDa protein	ROD1			1	
IPI00305692	Thioredoxin-like protein 1	TXNL1	O43396		1	
IPI00216132	Isoform Alpha I of Ribosomal protein S6 kinase beta-1	RPS6KB1	P23443			1
IPI00006705	Uteroglobin precursor	SCGB1A1	P11684		1	1
IPI00216313	Visinin-like protein 1	VSNL1	P62760		1	1
IPI00002897	Gamma-aminobutyric acid receptor subunit alpha-3 precursor	GABRA3	P34903		1	1
IPI00009891	Isoform 1 of Transcription initiation factor TFIID subunit 1	TAF1	P21675		1	1
IPI00328115	Isoform 3 of 5'-3' exoribonuclease 1	XRN1	Q8IZH2			1
IPI00031630	Melanocyte protein Pmel 17 precursor	SILV	P40967		1	

Table S1.

Number of unique peptides identified

IPI	Protein name	Gene symbol	Swiss Prot ID	Normal	CFS	nPTLS
IPI00167446	Isoform 2 of Uncharacterized protein C17orf78	C17orf78	Q8N4C9		1	
IPI00479625	Isoform 1 of ELAV-like protein 4	ELAVL4	P26378		1	
IPI00022048	Prostaglandin F2 receptor negative regulator precursor	PTGFRN	Q9P2B2			1
IPI00103055	Isoform 1 of Solute carrier family 35 member F5	SLC35F5	Q8WV83			1
IPI00167194	CDNA FLJ25694 fis, clone TST04471	FLJ25694				1
IPI00745395	Conserved hypothetical protein	KIAA0508			1	
IPI00152536	Isoform 1 of Transmembrane channel-like protein 2	TMC2	Q8TDI7			1
IPI00783271	Leucine-rich PPR motif-containing protein, mitochondrial precursor	LRPPRC	P42704			1
IPI00150057	Isoform 2 of SWI/SNF-related matrix-associated actin-dependent regulator of chromatin subfamily C member 2	SMARCC2	Q8TAQ2			1
IPI00456996	Conserved hypothetical protein	PVT1				1
IPI00023501	Isoform 1 of Tumor necrosis factor receptor superfamily member 1B precursor	TNFRSF1B	P20333		1	1
IPI00232891	Isoform 3 of Fibronectin type III domain-containing protein 1	FNDC1	Q4ZHG4			1
IPI00795119	Protein	LTBP1			1	1
IPI00018402	Tubulin-specific chaperone E	TBCE	Q15813			1
IPI00043069	Ankyrin repeat domain-containing protein 30A	ANKRD30A	Q9BXX3		1	
IPI00014316	Cyclin-dependent kinase 5 activator 1 precursor	CDK5R1	Q15078		1	
IPI00301726	Isoform 1 of Uncharacterized protein C3orf20	C3orf20	Q8ND61		1	
IPI00030404	Transcriptional repressor NF-X1	NFX1	Q12986			1
IPI00185361	ATP-dependent RNA helicase DDX55	DDX55	Q8NHQ9		1	
IPI00293867	D-dopachrome decarboxylase	DDT	P30046		1	
IPI00219661	Isoform 1 of Myelin proteolipid protein	PLP1	P60201			1
IPI00161055	Putative 2-oxo-4-hydroxy-4-carboxy-5-ureidoimidazoline decarboxylase	PRHOXNB	A6NGE7		1	1
IPI00619927	NAALAD2 protein	NAALAD2			1	1
IPI00794779	DNA polymerase theta	POLQ	O75417		1	
IPI00186853	pecanex-like 2	PCNXL2				1
IPI00478354	Putative uncharacterized protein CXorf31	CXorf31	Q5VT33		1	
IPI00395376	Isoform 1 of Semaphorin-5B	SEMA5B	Q9P283			1
IPI00555621	p21-activated kinase 2 variant (Fragment)	-				1
IPI00166414	Coiled-coil domain-containing protein 96	CCDC96	Q2M329		1	

Table S1.

IPI	Protein name	Gene symbol	Swiss Prot ID	Number of unique peptides identified		
				**Normal**	**CFS**	**nPTLS**
IPI00746232	coiled-coil domain containing 112 isoform 2	CCDC112				1
IPI00385578	HDCMC04P	MLL5			1	1
IPI00022020	Type II inositol-3,4-bisphosphate 4-phosphatase	INPP4B	O15327			1
IPI00300053	Keratin type II cuticular Hb2	KRT82	Q9NSB4		1	
IPI00749062	Conserved hypothetical protein	-			1	
IPI00382483	Ig heavy chain V-III region GA	-	P01769		2	3
IPI00020406	Beta-1,4-mannosyl-glycoprotein 4-beta-N-acetylglucosaminyltransferase	MGAT3	Q09327		2	
IPI00418236	Factor in the germline alpha	FIGLA	Q6QHK4			1
IPI00328762	Isoform 1 of ATP-binding cassette sub-family A member 13	ABCA13	Q86UQ4			1
IPI00748342	Beclin-1	BECN1	Q14457			1
IPI00739423	similar to c11.1 CG12132-PA isoform 11	LOC377711				1
IPI00446753	SCO-spondin precursor	SSPO	A2VEC9		1	1
IPI00452161	Isoform 1 of Mucolipin-1	MCOLN1	Q9GZU1		1	
IPI00026970	FACT complex subunit SPT16	SUPT16H	Q9Y5B9			1
IPI00010130	Glutamine synthetase	GLUL	P15104			2
IPI00384546	CDNA FLJ25883 fis, clone CBR02735	-			1	
IPI00382439	Ig lambda chain V-IV region X	-	P01716		1	1
IPI00549384	Isoform 1 of Bromodomain-containing protein 9	BRD9	Q9H8M2			1
IPI00479893	Isoform 2 of Uncharacterized protein C10orf18	C10orf18	Q5VWN6			1
IPI00412272	SH3 domain-binding glutamic acid-rich-like protein 2	SH3BGRL2	Q9UJC5		1	
IPI00876910	Isoform 1 of Ankyrin repeat and SOCS box-containing protein 18	ASB18	Q6ZVZ8			1
IPI00477766	Isoform 3 of Protein PTHB1	BBS9	Q3SYG4			1
IPI00006298	Isoform 1 of Peptidyl-prolyl cis-trans isomerase G	PPIG	Q13427		1	
IPI00031497	Conserved hypothetical protein	C7orf49			1	
IPI00006631	Synaptic vesicle glycoprotein 2B	SV2B	Q7L1I2		1	2
IPI00872208	122 kDa protein	KIAA1856			1	1
IPI00301503	Isoform 1 of Splicing factor, arginine/serine-rich 10	SFRS10	P62995			1
IPI00022061	Sodium/hydrogen exchanger 6	SLC9A6	Q92581		1	
IPI00425688	Isoform 1 of RUN and SH3 domain-containing protein 1	RUSC1	Q9BVN2			1
IPI00787427	ankyrin repeat domain 36	ANKRD36				1
IPI00167241	Zinc finger protein 283	ZNF283	Q8N7M2			1
IPI00220325	Isoform Short of Insulin receptor precursor	INSR	P06213			1

Table S1.

Number of unique peptides identified

IPI	Protein name	Gene symbol	Swiss Prot ID	Normal	CFS	nPTLS
IPI00217418	Isoform 2 of Rho GTPase-activating protein 12	ARHGAP12	Q8IWW6		1	
IPI00604549	Isoform 2 of Phosphatidylinositol-3,4,5-trisphosphate 5-phosphatase 1	LOC646743	Q92835		1	1
IPI00032541	Keratin type II cuticular Hb5	KRT85	P78386			1
IPI00743545	Conserved hypothetical protein	-			1	
IPI00328156	Amine oxidase [flavin-containing] B	MAOB	P27338		1	
IPI00384874	Isoform 1 of ATP-binding cassette sub-family A member 10	ABCA10	Q8WWZ4			1
IPI00644785	Protein	PLXNB3			1	
IPI00019690	death-inducing-protein	DIP				1
IPI00744816	Conserved hypothetical protein	-			1	1
IPI00654693	Protein FAM44C	FAM44C	Q8IYS8		1	
IPI00027723	Elastase-2B precursor	ELA2B	P08218			1
IPI00880011	116 kDa protein	-			1	
IPI00335711	Ciliary dynein heavy chain 11	DNAH11	Q96DT5		1	
IPI00022820	Transcription initiation factor IIB	GTF2B	Q00403		1	1
IPI00018583	Isoform 1 of Intracellular hyaluronan-binding protein 4	HABP4	Q5JVS0		1	
IPI00152432	Isoform 1 of Alanine aminotransferase 2	GPT2	Q8TD30		1	1
IPI00015737	15 kDa protein	DCAKD				1
IPI00549189	Thimet oligopeptidase	THOP1	P52888		1	1
IPI00030986	Isoform Long of Kelch repeat and BTB domain-containing protein 10	KBTBD10	O60662		1	
IPI00641954	22 kDa protein	TMED5				1
IPI00027220	Isoform 1 of Cell adhesion molecule-related/down-regulated by oncogenes precursor	CDON	Q4KMG0		2	1
IPI00739676	Isoform 2 of Protein DDX26B	DDX26B	Q5JSJ4			1
IPI00045503	Isoform 2 of Protein phosphatase 1 regulatory subunit 14A	PPP1R14A	Q96A00		1	
IPI00793408	6 kDa protein	ADAMTS9			1	
IPI00477763	Serine/threonine-protein kinase MRCK beta	CDC42BPB	Q9Y5S2			2
IPI00021302	Sushi domain-containing protein 2 precursor	SUSD2	Q9UGT4		1	1
IPI00024255	G patch domain and KOW motifs-containing protein	GPKOW	Q92917		1	
IPI00028262	67 kDa protein	KIAA1754			1	
IPI00402006	Similar to DNA-binding protein	-				1
IPI00001528	Isoform C of Interleukin-18-binding protein precursor	IL18BP	O95998		3	3
IPI00477949	Isoform 1 of Zinc finger MYM-type protein 4	ZMYM4	Q5VZL5			1

Table S1.

Number of unique peptides identified

IPI	Protein name	Gene symbol	Swiss Prot ID	Normal	CFS	nPTLS
IPI00027212	Insulin receptor-related protein precursor	INSRR	P14616		1	
IPI00009724	Isoform 1 of EF-hand calcium-binding domain-containing protein 6	EFCAB6	Q5THR3		1	
IPI00737448	similar to heat shock 70kD protein binding protein	LOC338805				1
IPI00868835	Similar to Heterogeneous nuclear ribonucleoproteins C1/C2 (hnRNP C1 / hnRNP C2). Isoform 4	-			1	
IPI00395444	calmodulin regulated spectrin-associated protein 1	CAMSAP1			1	1
IPI00176574	similar to large subunit ribosomal protein L36a	LOC284230			1	1
IPI00396658	Isoform 2 of Protein ITFG3	ITFG3	Q9H0X4		1	
IPI00383261	Peroxin Pex6p	PEX6				1
IPI00742127	Similar to Heterogeneous nuclear ribonucleoprotein A1 (Helix-destabilizing protein) (Single-strand RNA-binding protein) (hnRNP core protein A1). Isoform A1-A	-			1	
IPI00398586	hypothetical protein LOC199800	LOC199800			1	
IPI00418164	Novel protein	RP4-692D3.1			1	
IPI00022735	Synaptotagmin-4	SYT4	Q9H2B2			1
IPI00033019	Potassium voltage-gated channel subfamily B member 1	KCNB1	Q14721		1	
IPI00032598	Isoform 1 of ETS translocation variant 3	ETV3	P41162		1	
IPI00217494	Isoform 1 of Protein SMG7	SMG7	Q92540		1	
IPI00000837	Metabotropic glutamate receptor 3 precursor	GRM3	Q14832		1	
IPI00296535	2-hydroxyacyl-CoA lyase 1	HACL1	Q9UJ83		1	
IPI00026058	Isoform 1 of Leucine zipper putative tumor suppressor 1	LZTS1	Q9Y250			1
IPI00062882	ATP-binding domain-containing protein 3	ATPBD3	Q7Z7A3		1	
IPI00000436	Cadherin-22 precursor	CDH22	Q9UJ99		2	2
IPI00479789	Isoform 1 of Uncharacterized protein C1orf103	C1orf103	Q5T3J3			1
IPI00411637	Galactose-3-O-sulfotransferase 4	GAL3ST4	Q96RP7			1
IPI00445167	CDNA FLJ44499 fis, clone UTERU3000665, highly similar to Homo sapiens Snf2-related CBP activator protein	SRCAP			1	
IPI00018880	Tumor necrosis factor receptor superfamily member 1A precursor	TNFRSF1A	P19438		2	

Table S1.

Number of unique peptides identified

IPI	Protein name	Gene symbol	Swiss Prot ID	Normal	CFS	nPTLS
IPI00154834	Isoform 1 of Neighbor of punc e11 precursor	NOPE	Q8TDY8			1
IPI00442582	CDNA FLJ26893 fis, clone RCT00305	-			1	
IPI00011274	Isoform 1 of Heterogeneous nuclear ribonucleoprotein D-like	HNRPDL	O14979		1	
IPI00007360	Zinc finger protein 238	ZNF238	Q99592			1
IPI00217787	Isoform 1 of Uncharacterized protein C12orf53 precursor	C12orf53	Q8IYJ0		2	3
IPI00384280	Prenylcysteine oxidase 1 precursor	PCYOX1	Q9UHG3			3
IPI00152733	FLJ00237 protein (Fragment)	OLFML2A				1
IPI00002791	Isoform Wnt-16b of Protein Wnt-16 precursor	WNT16	Q9UBV4		1	
IPI00290432	Natural resistance-associated macrophage protein 1	SLC11A1	P49279		1	
IPI00032876	Cytokine-like protein 1 precursor	CYTL1	Q9NRR1		2	2
IPI00793585	57 kDa protein	OC90				1
IPI00012540	Prominin-1 precursor	PROM1	O43490		1	
IPI00477396	Uncharacterized protein ZNF324B (Fragment)	ZNF584			1	
IPI00029133	ATP synthase subunit b, mitochondrial precursor	ATP5F1	P24539			1
IPI00032939	Uncharacterized protein WIPF3	WIPF3			1	1
IPI00853149	RW1 protein	TMEM131	Q92545		1	
IPI00168977	Olfactory receptor 6J1	OR6J1	Q8NGC5		1	
IPI00395010	hypothetical protein LOC65250	C5orf42			1	1
IPI00218271	Isoform 3 of MAGUK p55 subfamily member 2	MPP2	Q14168			1
IPI00003949	Ubiquitin-conjugating enzyme E2 N	UBE2N	P61088			1
IPI00165955	Isoform 1 of Mitogen-activated protein kinase 15	MAPK15	Q8TD08		1	
IPI00009504	Solute carrier family 20 member 2	SLC20A2			1	1
IPI00304992	Isoform 1 of Zinc finger protein 503	ZNF503	Q96F45			1
IPI00377211	Isoform 3 of Cadherin-like protein 26 precursor	CDH26	Q8IXH8		1	
IPI00100247	Thioredoxin domain-containing protein 13 precursor	TXNDC13	Q9H1E5		1	
IPI00292134	Epidermal growth factor receptor substrate 15	EPS15	P42566		1	1
IPI00006612	Isoform 1 of Clathrin coat assembly protein AP180	SNAP91	O60641			1
IPI00642305	MCM8 protein	MCM8				1
IPI00736558	THAP domain containing 4	THAP4	Q8WY91		1	
IPI00470518	Mitotic spindle assembly checkpoint protein MAD1	MAD1L1	Q9Y6D9		1	

Table S1.

Number of unique peptides identified

IPI	Protein name	Gene symbol	Swiss Prot ID	Normal	CFS	nPTLS
IPI00005087	Tropomodulin-3	TMOD3	Q9NYL9		1	
IPI00026320	E3 ubiquitin-protein ligase UBR5	UBR5	O95071			1
IPI00010142	Ubiquitin-conjugating enzyme E2 G2	UBE2G2	P60604			1
IPI00029227	LOC93622 protein	LOC93622			1	
IPI00029769	Isoform p59-HCK of Tyrosine-protein kinase HCK	HCK	P08631			1
IPI00446138	CDNA FLJ42768 fis, clone BRAWH3003522	-				1
IPI00218839	Cytochrome P450 1A1	CYP1A1	P04798		1	
IPI00006356	Isoform 1 of Patatin-like phospholipase domain-containing protein 5	PNPLA5	Q7Z6Z6		1	
IPI00449071	Isoform 5a of Paired box protein Pax-6	PAX6	P26367			1
IPI00328268	EIF4G3 protein	EIF4G3				2
IPI00005616	Isoform 1 of Trace amine-associated receptor 2	TAAR2	Q9P1P5		1	
IPI00789494	8 kDa protein	TUSC4			1	
IPI00305477	Cystatin-SN precursor	CST1	P01037			2
IPI00011773	Uncharacterized protein C20orf177	C20orf177	Q9NTX9		1	2
IPI00745346	Isoform 11 of Peroxisomal N(1)-acetyl-spermine/spermidine oxidase	PAOX	Q6QHF9		1	
IPI00432893	INPP5F protein (Fragment)	INPP5F				1
IPI00220039	Isoform 1 of Transmembrane protein 16K	TMEM16K	Q9NW15			1
IPI00027596	Secreted frizzled-related protein 2 precursor	SFRP2	Q96HF1		1	
IPI00783950	Isoform 6 of Titin	TTN	Q8WZ42		1	1
IPI00008455	Isoform 2 of Myosin-VI	MYO6	Q9UM54		1	
IPI00217850	Isoform 1 of GRAM domain-containing protein 1C	GRAMD1C	Q8IYS0		1	
IPI00020771	Isoform 3 of Ensconsin	MAP7	Q14244		1	
IPI00170800	Isoform 1 of Otoancorin precursor	OTOA	Q7RTW8		1	
IPI00000270	Ribonuclease 7 precursor	RNASE7	Q9H1E1			1
IPI00031171	Isoform 1 of Interleukin-6 receptor alpha chain precursor	IL6R	P08887		2	2
IPI00479430	Isoform 2 of Complement C1q tumor necrosis factor-related protein 9 precursor	C1QTNF9	Q5VX65			1
IPI00063181	CDKN2A interacting protein N-terminal like	CDKN2AIPNL			1	
IPI00328225	Isoform 1 of T-box transcription factor TBX20	TBX20	Q9UMR3		1	
IPI00296798	Isoform 1 of Fibrinogen C domain-containing protein 1	FIBCD1	Q8N539			1
IPI00784385	Adenylate cyclase type 1	ADCY1	Q08828		2	

Table S1.

IPI	Protein name	Gene symbol	Swiss Prot ID	Normal	CFS	nPTLS
				Number of unique peptides identified		
IPI00152189	Putative uncharacterized protein	-			1	1
IPI00166331	Ankyrin repeat domain-containing protein 35	ANKRD35	Q8N283		1	
IPI00294386	Myosin-Ia	MYO1A	Q9UBC5		1	
IPI00061680	SAGA-associated factor 29 homolog	CCDC101	Q96ES7		1	
IPI00220901	TBC1D4 protein	TBC1D4	O60343		1	
IPI00059139	Vacuolar proton pump subunit E 2	ATP6V1E2	Q96A05		1	
IPI00743335	myosin IC isoform a	MYO1C			1	1
IPI00003483	Isoform 1 of Neuralized-like protein 1	NEURL	O76050			1
IPI00059711	LRP11 protein (Fragment)	LRP11			1	
IPI00010893	Left-right determination factor 2 precursor	LEFTY2	O00292		1	
IPI00026185	Isoform 1 of F-actin-capping protein subunit beta	CAPZB	P47756		1	2
IPI00419575	Protein of unknown function DUF410 family protein	C7orf20	Q7L5D6			1
IPI00019090	Collagen alpha-1	COL19A1	Q14993			1
IPI00011756	Homeobox protein Meis1	MEIS1	O00470			1
IPI00746222	ATP-binding cassette, sub-family B (MDR/TAP), member 5	ABCB5	Q2M3G0		1	
IPI00010843	Isoform 1 of Amyloid beta A4 precursor protein-binding family B member 1	APBB1	O00213		1	
IPI00012687	27 kDa protein	C1orf83				1
IPI00332106	Isoform 1 of Pre-B-cell leukemia transcription factor-interacting protein 1	PBXIP1	Q96AQ6			1
IPI00012353	Mitochondrial 39S ribosomal protein L3	MRPL3	P09001		1	
IPI00418426	Metal transporter CNNM4	CNNM4	Q6P4Q7			1
IPI00002524	ATP-sensitive inward rectifier potassium channel 14	KCNJ14	Q9UNX9			1
IPI00000110	Zinc finger protein 337	ZNF337	Q9Y3M9			1
IPI00297767	Casein kinase I isoform gamma-2	CSNK1G2	P78368			1
IPI00385065	Isoform 1 of Coiled-coil domain-containing protein 46	CCDC46	Q8N8E3		1	
IPI00007207	Isoform 1 of Lysosomal acid lipase/cholesteryl ester hydrolase precursor	LIPA	P38571			1
IPI00413930	Elongation of very long chain fatty acids protein 2	ELOVL2	Q9NXB9		1	
IPI00006715	Double-strand-break repair protein rad21 homolog	RAD21	O60216		1	
IPI00787189	similar to thioredoxin domain containing 4	LOC727931			1	
IPI00023868	Canalicular multispecific organic anion transporter 1	ABCC2	Q92887			1

Table S1.

Number of unique peptides identified

IPI	Protein name	Gene symbol	Swiss Prot ID	Normal	CFS	nPTLS
IPI00056309	Liver-expressed antimicrobial peptide 2 precursor	LEAP2	Q969E1		1	1
IPI00031522	Trifunctional enzyme subunit alpha, mitochondrial precursor	HADHA	P40939			1
IPI00215965	Isoform A1-B of Heterogeneous nuclear ribonucleoprotein A1	HNRNPA1	P09651		1	
IPI00030237	Isoform 1 of Uncharacterized protein C1orf49	C1orf49	Q5T0J7		1	
IPI00070070	Novel protein	FLJ32784				2
IPI00088953	Phosphoinositide 3-kinase regulatory subunit 6	C17orf38	Q5UE93		1	
IPI00329142	Glutamate-rich protein 1	ERICH1	Q86X53			1
IPI00169253	Olfactory receptor OR1-45	OR5AT1				1
IPI00031490	Isoform 1 of Collectin-11 precursor	COLEC11	Q9BWP8		1	
IPI00001516	Isoform Long of Protocadherin alpha C2 precursor	PCDHAC2	Q9Y5I4		1	1
IPI00045801	Flt3-interacting zinc finger protein 1	FIZ1	Q96SL8			1
IPI00017802	Isoform 1 of Methylglutaconyl-CoA hydratase, mitochondrial precursor	AUH	Q13825			1
IPI00012573	Peroxisome assembly protein 12	PEX12	O00623		1	1
IPI00384549	TTC31 protein (Fragment)	TTC31				1
IPI00012756	Interferon-induced protein with tetratricopeptide repeats 5	IFIT5	Q13325			1
IPI00418573	Isoform 2 of Netrin receptor UNC5A precursor	UNC5A	Q6ZN44		2	3
IPI00430079	Isoform 2 of PHD finger protein 8	PHF8	Q9UPP1		1	
IPI00141933	Mitotic checkpoint serine/threonine-protein kinase BUB1 beta	BUB1B	O60566		1	
IPI00297985	Uncharacterized protein C6orf103	C6orf103	Q8N7X0			1
IPI00296942	Cadherin-12 precursor	CDH12	P55289		1	
IPI00514394	Family with sequence similarity 120A opposite strand	FAM120AOS				1
IPI00852688	Isoform 1 of Low density lipoprotein receptor-related protein 5-like protein	LRP5L	A4QPB2		1	
IPI00034088	Interleukin-27 beta chain precursor	EBI3	Q14213			1
IPI00060440	Zinc finger protein GLI4	GLI4	P10075		1	
IPI00845299	Putative uncharacterized protein LOC285733	LOC285733			1	
IPI00305856	Homeobox protein Hox-D11	HOXD11	P31277			1
IPI00003897	Isoform 1 of Protocadherin gamma B6 precursor	PCDHGB6	Q9Y5F9		1	
IPI00797859	10 kDa protein	LOC643711				1
IPI00395769	Isoform Heart of ATP synthase subunit gamma, mitochondrial precursor	ATP5C1	P36542		1	1

Table S1.

Number of unique peptides identified

IPI	Protein name	Gene symbol	Swiss Prot ID	Normal	CFS	nPTLS
IPI00385737	similar to Nonhistone chromosomal protein HMG-17	LOC646853				1
IPI00152257	Enkurin	C10orf63	Q8TC29		1	
IPI00043526	Uncharacterized protein C14orf44	C14orf44	Q96MY7		1	
IPI00015195	Cleavage stimulation factor 77 kDa subunit	CSTF3	Q12996			1
IPI00006158	Lymphoid-restricted membrane protein	LRMP	Q12912			1
IPI00008554	Angiogenin precursor	ANG	P03950			1
IPI00028158	Neurotensin/neuromedin N precursor	NTS	P30990			1
IPI00013373	Orexin precursor	HCRT	O43612		1	
IPI00294650	Secreted frizzled-related protein 3 precursor	FRZB	Q92765		6	5
IPI00384393	Myosin-reactive immunoglobulin heavy chain variable region (Fragment)	-				1
IPI00021129	Isoform 1 of AP-3 complex subunit beta-1	AP3B1	O00203			1
IPI00829925	Uncharacterized protein ENSP00000365090	ELA2A			1	
IPI00013871	Ribonucleoside-diphosphate reductase large subunit	RRM1	P23921			1
IPI00029628	Reticulocalbin-2 precursor	RCN2	Q14257		2	3
IPI00020039	Cylicin-1	CYLC1	P35663			1
IPI00658167	Isoform 1 of Protein cramped-like	CRAMP1L	Q96RY5		1	1
IPI00009333	Transmembrane protein 9 precursor	TMEM9	Q9P0T7		1	
IPI00470917	Isoform 2 of Uncharacterized protein KIAA1529	KIAA1529	Q9P1Z9		1	
IPI00013269	Isoform 1 of Uncharacterized protein C6orf106	C6orf106	Q9H6K1		1	
IPI00737712	CCDC125 protein	CCDC125				1
IPI00219036	dicer1	DICER1	Q9UPY3			1
IPI00008752	Isoform 1 of Metallothionein-1G	MT1G	P13640		6	3
IPI00306343	Isoform 1 of RNA-binding protein 41	RBM41	Q96IZ5		1	1
IPI00295461	Isoform 1 of Seprase	FAP	Q12884		1	2
IPI00022433	Heat-shock protein beta-6	HSPB6	O14558		1	
IPI00179694	Beta-klotho	KLB	Q86Z14		1	
IPI00027642	Isoform 1 of JmjC domain-containing histone demethylation protein 3C	JMJD2C	Q9H3R0		1	
IPI00797832	Isoform 3 of Acyl-CoA dehydrogenase family member 10	ACAD10	Q6JQN1		1	
IPI00289849	Leucine-rich repeat and fibronectin type-III domain-containing protein 6 precursor	ELFN2	Q5R3F8		1	2
IPI00410464	Isoform 1 of Zinc finger protein 324B	ZNF324B	Q6AW86		1	
IPI00297212	Paladin	KIAA1274	Q9ULE6			1

Table S1.

Number of unique peptides identified

IPI	Protein name	Gene symbol	Swiss Prot ID	Normal	CFS	nPTLS
IPI00166863	Isoform 2 of Coiled-coil domain-containing protein 123, mitochondrial precursor	CCDC123	Q96ST8		1	
IPI00418185	Putative uncharacterized protein FLJ20097	CCDC132				1
IPI00008832	Growth arrest-specific protein 1 precursor	GAS1	P54826		1	
IPI00423562	Isoform 1 of Ubiquitin carboxyl-terminal hydrolase 6	USP6	P35125			1
IPI00014958	Isoform 1 of Serum paraoxonase/arylesterase 2	PON2	Q15165		1	
IPI00004101	Betaine--homocysteine S-methyltransferase 1	BHMT	Q93088			1
IPI00719669	Myosin regulatory light chain	MRLC2			1	
IPI00299084	Transmembrane protein 33	TMEM33	P57088		1	
IPI00465168	Isoform 1 of Serine/threonine-protein kinase TAO2	TAOK2	Q9UL54		2	
IPI00028213	Semaphorin-3D precursor	SEMA3D	O95025		2	
IPI00008475	Hydroxymethylglutaryl-CoA synthase, cytoplasmic	HMGCS1	Q01581			1
IPI00023604	Thyroliberin precursor	TRH	P20396		2	1
IPI00029737	Isoform Long of Long-chain-fatty-acid--CoA ligase 4	ACSL4	O60488		1	
IPI00005661	Isoform Gamma of Max-like protein X	MLX	Q9UH92		1	
IPI00412107	FLJ43980 protein	FLJ43980				1
IPI00328793	Sterol regulatory element-binding protein 2	SREBF2	Q12772			1
IPI00217683	A-kinase anchor protein 12 isoform 2	AKAP12			1	
IPI00023555	Isoform 1a of Oxysterol-binding protein-related protein 3	OSBPL3	Q9H4L5		1	1
IPI00004563	Protein FAM105A	FAM105A	Q9NUU6			1
IPI00291662	LON peptidase N-terminal domain and ring finger 1	LONRF1			1	1
IPI00418966	hypothetical protein LOC123688 isoform 2	LOC123688			1	
IPI00013257	Single-stranded DNA-binding protein 4	SSBP4	Q9BWG4			1
IPI00328409	Armadillo repeat-containing protein 4	ARMC4	Q5T2S8		1	
IPI00002707	Isoform 1 of Spastin	SPAST	Q9UBP0			2
IPI00065457	OTTHUMP00000018353	FLJ32682			1	
IPI00444502	CDNA FLJ45482 fis, clone BRTHA2001953	FLJ45482			1	
IPI00158804	basic, immunoglobulin-like variable motif containing	BIVM				1
IPI00001780	Ubiquitin carboxyl-terminal hydrolase 16	USP16	Q9Y5T5		1	

Table S1.

IPI	Protein name	Gene symbol	Swiss Prot ID	Normal	CFS	nPTLS
				Number of unique peptides identified		
IPI00552840	Probable G-protein coupled receptor 112	GPR112	Q8IZF6		1	
IPI00016373	Ras-related protein Rab-13	RAB13	P51153		1	
IPI00552434	Myosin, heavy chain 7B, cardiac muscle, beta	MYH7B			1	
IPI00166606	KIAA0913 protein (Fragment)	KIAA0913				1
IPI00329014	Isoform 1 of Ankyrin repeat domain-containing protein 46	ANKRD46	Q86W74			1
IPI00306402	Lysyl oxidase homolog 4 precursor	LOXL4	Q96JB6		1	1
IPI00876962	Isoform 2 of Inverted formin-2	INF2	Q27J81		1	
IPI00026512	Isoform HERA-A of GTP-binding protein era homolog	ERAL1	O75616		1	
IPI00395603	Isoform 3 of Uncharacterized protein C18orf34	C18orf34	Q5BJE1			1
IPI00180375	Isoform 1 of Beta-arrestin-2	ARRB2	P32121		1	
IPI00852619	Isoform A of Probable phospholipid-transporting ATPase VB	ATP10B	O94823			2
IPI00303980	Isoform 1 of Uncharacterized protein C14orf93 precursor	C14orf93	Q9H972			1
IPI00433499	rhomboid, veinlet-like 6 isoform 1	RHBDF2				1
IPI00006196	Isoform 2 of Nuclear mitotic apparatus protein 1	NUMA1	Q14980			1
IPI00297407	Solute carrier family 45 member 4	SLC45A4			1	
IPI00829979	Uncharacterized protein ENSP00000375008	-			3	2
IPI00004446	Sushi repeat-containing protein SRPX2 precursor	SRPX2	O60687			1
IPI00247295	Isoform 4 of Nesprin-1	SYNE1	Q8NF91			3
IPI00647124	A1 chain of type XIX collagen	COL19A1			1	1
IPI00152879	Monocarboxylate transporter 10	SLC16A10	Q8TF71		1	
IPI00290155	66 kDa protein	ZNF148			1	
IPI00010448	Isoform 2 of Rho GTPase-activating protein 24	ARHGAP24	Q8N264			1
IPI00747017	Putative uncharacterized protein DKFZp686D06121	NEK1			1	
IPI00793836	11 kDa protein	ARHGDIB			1	
IPI00737392	similar to proline-rich protein BstNI subfamily 3 precursor	LOC646352				1
IPI00382422	Ig lambda chain V-I region NEWM	-	P01703		1	
IPI00328842	ARHGAP4 protein	ARHGAP4			1	
IPI00219503	Isoform 1 of F-box only protein 24	FBXO24	O75426			1
IPI00414591	Flavoprotein oxidoreductase	MICAL2	O94851			1
IPI00008632	Isoform 1 of Putative homeodomain transcription factor 1	PHTF1	Q9UMS5		1	

Table S1.

Number of unique peptides identified

IPI	Protein name	Gene symbol	Swiss Prot ID	Normal	CFS	nPTLS
IPI00018240	Isoform 1 of Protein SDA1 homolog	SDAD1	Q9NVU7			1
IPI00296421	EH domain-binding protein 1-like protein 1	EHBP1L1	Q8N3D4		2	
IPI00657892	Factor VIII	F8				1
IPI00386651	LOC404266 protein	LOC404266				1
IPI00142538	Isoform 1 of Probable helicase senataxin	SETX	Q7Z333		2	1
IPI00065253	Uncharacterized protein C3orf30	C3orf30	Q96M34		1	
IPI00166933	Uncharacterized protein C10orf12	C10orf12	Q8N655			1
IPI00005254	Isoform 1 of E3 ubiquitin-protein ligase parkin	PARK2	O60260			1
IPI00883751	RNA-directed DNA polymerase (Reverse transcriptase), related domain containing protein	-			1	
IPI00290903	Chromosome 2 open reading frame 65	C2orf65			1	1
IPI00005721	Neutrophil defensin 1 precursor	DEFA1	P59665		2	3
IPI00216137	Synaptonemal complex protein 1	SYCP1	Q15431		1	
IPI00005621	7h3 protein (Fragment)	SYDE1			1	
IPI00003053	CDNA: FLJ23510 fis, clone LNG03216	ARMC9			1	1
IPI00005563	Isoform 1 of Tubulointerstitial nephritis antigen-like precursor	TINAGL1	Q9GZM7			2
IPI00001933	Coiled-coil domain-containing protein 113	CCDC113	Q9H0I3		1	
IPI00411291	Peroxisome biogenesis factor 1	PEX1	O43933		1	
IPI00383294	Putative uncharacterized protein pp7518	MNT			1	
IPI00016861	102 kDa protein	GTF3C2			1	
IPI00065378	Isoform 1 of DC-STAMP domain-containing protein 1	DCST1	Q5T197			1
IPI00016848	Uncharacterized protein C20orf103 precursor	C20orf103	Q9UJQ1		1	1
IPI00163718	Isoform 4 of Vacuolar protein sorting-associated protein 54	VPS54	Q9P1Q0			1
IPI00219817	Isoform 2 of C-jun-amino-terminal kinase-interacting protein 3	MAPK8IP3	Q9UPT6		1	1
IPI00414819	Helicase SKI2W	SKIV2L	Q15477			1
IPI00015343	endothelial differentiation, sphingolipid G-protein-coupled receptor, 1	EDG1	P21453		2	2
IPI00020903	Isoform 1 of AF4/FMR2 family member 2	AFF2	P51816		1	
IPI00465247	Isoform 1 of Stimulated by retinoic acid gene 6 protein homolog	STRA6	Q9BX79		1	
IPI00382394	Isoform 5 of Cytosolic phospholipase A2 beta	PLA2G4B	O95712		1	
IPI00041588	Ral guanine nucleotide exchange factor RalGPS1A	RALGPS1				1

Table S1.

Number of unique peptides identified

IPI	Protein name	Gene symbol	Swiss Prot ID	Normal	CFS	nPTLS
IPI00100787	RANBP2-like and GRIP domain-containing protein 7	RGPD5	Q9H0B2			1
IPI00167913	Isoform 1 of Spermatid-associated protein	SPERT	Q8NA61		1	
IPI00743696	Uncharacterized protein COL4A1	COL4A1			2	1
IPI00874276	Putative uncharacterized protein DKFZp761N1814	EPB41L4B			1	
IPI00873759	Uncharacterized protein ENSP00000381675 (Fragment)	-				1
IPI00012390	Homeobox protein Hox-D4	HOXD4	P09016			1
IPI00446767	Isoform 3 of Transducin-like enhancer protein 4	TLE4	Q04727		1	
IPI00027694	Isoform 3 of Homeobox protein Hox-A1	HOXA1	P49639			1
IPI00020754	PRO1751	-			1	
IPI00514551	OTTHUMP00000016936	SCML4				1
IPI00747264	Gamma-aminobutyric-acid receptor subunit rho-1 precursor	GABRR1	P24046			1
IPI00386574	Ig lambda chain V-I region MEM	-	P06887		1	1
IPI00027667	Gastrin/cholecystokinin type B receptor	CCKBR	P32239		1	1
IPI00296063	Isoform 1 of Sodium channel protein type 11 subunit alpha	SCN11A	Q9UI33		1	1
IPI00337544	Isoform 2 of Myomegalin	PDE4DIP	Q5VU43			1
IPI00300060	WD repeat-containing protein 70	WDR70	Q9NW82		1	
IPI00021979	Peroxisomal membrane protein 11A	PEX11A	O75192		1	
IPI00031768	Hook homolog 3	HOOK3	Q86VS8		1	
IPI00171160	Isoform 1 of Leucine-rich repeat-containing protein 17 precursor	LRRC17	Q8N6Y2		1	1
IPI00029039	Regenerating islet-derived protein 3 alpha precursor	REG3A	Q06141		1	1
IPI00216986	Isoform C of Complement receptor type 2 precursor	CR2	P20023		1	
IPI00470771	SAMD4B protein	SAMD4B			1	
IPI00020795	R33083_1 (Fragment)	DPP9				1
IPI00010740	Isoform Long of Splicing factor, proline- and glutamine-rich	SFPQ	P23246			1
IPI00021143	Class B basic helix-loop-helix protein 2	BHLHB2	O14503		1	
IPI00297931	Isoform 1 of AP1 subunit gamma-binding protein 1	AP1GBP1	Q9UMZ2		1	
IPI00019243	Isoform Long of Matrix metalloproteinase-16 precursor	MMP16	P51512			1
IPI00018691	28S ribosomal protein S18a, mitochondrial precursor	MRPS18A	Q9NVS2		1	
IPI00000203	Low-density lipoprotein receptor-related protein 6 precursor	LRP6	O75581			2

Table S1.

Number of unique peptides identified

IPI	Protein name	Gene symbol	Swiss Prot ID	Normal	CFS	nPTLS
IPI00749338	Hypothetical gene supported by BC063892	hCG_1651889				1
IPI00221009	Isoform 7 of Transcription factor 7-like 2	TCF7L2	Q9NQB0			1
IPI00010604	Isoform 1 of 1-phosphatidylinositol-4,5-bisphosphate phosphodiesterase epsilon-1	PLCE1	Q9P212		1	
IPI00022438	Isoform Long of Transcription factor Maf	MAF	O75444		1	
IPI00012402	Carbohydrate sulfotransferase 6	CHST6	Q9GZX3		1	2
IPI00396527	Isoform 1 of Coiled-coil domain-containing protein 128	CCDC128	Q6ZMI0		1	
IPI00398725	Isoform 1 of Zinc finger protein 644	ZNF644	Q9H582			1
IPI00291840	Isoform 1 of Zinc finger protein 90 homolog	ZFP90	Q8TF47		1	
IPI00387098	Ig kappa chain V-I region OU	-	P01606		1	
IPI00022479	guanine nucleotide exchange factor p532	HERC1			2	
IPI00745832	IQ calmodulin-binding region domain containing protein	-				1
IPI00167228	CDNA FLJ40966 fis, clone UTERU2012407	CHCHD7				1
IPI00646410	Isoform 2 of Flavin-containing amine oxidase domain-containing protein 1	AOF1	Q8NB78		1	
IPI00451413	Intersectin 1 isoform 7	ITSN1			1	
IPI00007067	Golgi-associated plant pathogenesis-related protein 1	C9orf19	Q9H4G4			1
IPI00787410	similar to Polypeptide N-acetylgalactosaminyltransferase 9	GALNT9			1	
IPI00410616	Isoform 1 of Endonuclease VIII-like 2	NEIL2	Q969S2			1
IPI00005978	Splicing factor, arginine/serine-rich 2	SFRS2	Q01130			1
IPI00021828	Cystatin-B	CSTB	P04080		2	1
IPI00844512	RHOV protein (Fragment)	RHOV				1
IPI00552601	Putative uncharacterized protein DKFZp686M12165 (Fragment)	-			1	
IPI00879389	20 kDa protein	ZMYND11				1
IPI00329251	Isoform 1 of Transmembrane protein 146 precursor	TMEM146	Q86XM0			1
IPI00293588	Isoform 1 of Tomoregulin-1 precursor	TMEFF1	Q8IYR6		3	4
IPI00166833	LAG1 longevity assurance homolog 5	LASS5	Q8N5B7		1	
IPI00183425	CDKN1A interacting zinc finger protein 1	CIZ1			1	
IPI00465087	Ubiquitin carboxyl-terminal hydrolase BAP1	BAP1	Q92560			1
IPI00027228	Probable glutamyl-tRNA(Gln) amidotransferase subunit B, mitochondrial precursor	PET112L	O75879		1	

Table S1.

Number of unique peptides identified

IPI	Protein name	Gene symbol	Swiss Prot ID	Normal	CFS	nPTLS
IPI00007917	Collectin-10 precursor	COLEC10	Q9Y6Z7		1	
IPI00103812	PWWP domain-containing protein 2B	PWWP2B	Q6NUJ5			1
IPI00552787	similar to Matrin-3	RBM20			1	
IPI00010953	CDNA FLJ11251 fis, clone PLACE1008813	EXOC6				1
IPI00639924	97 kDa protein	LOC374920				1
IPI00065484	CDNA FLJ32471 fis, clone SKNMC2000322, weakly similar to MAJOR CENTROMERE AUTOANTIGEN B	PTRH2			1	
IPI00384447	Sarcoma antigen NY-SAR-22 (Fragment)	NEXN			1	
IPI00830073	Isoform 1 of RING finger protein 207	RNF207	Q6ZRF8		1	
IPI00292579	Stabilin-2 precursor	STAB2	Q8WWQ8			1
IPI00012411	Zinc finger protein 32	ZNF32	P17041		1	
IPI00303335	Nebulin	NEB	P20929		1	1
IPI00067920	FXYD domain-containing ion transport regulator 7	FXYD7	P58549			1
IPI00879162	Uncharacterized protein ENSP00000374866	-				1
IPI00760993	IQ motif and Sec7 domain 2 isoform 2	IQSEC2			1	
IPI00004527	Uncharacterized protein KIAA0355	KIAA0355	O15063		1	
IPI00009804	DNA-binding protein SATB1	SATB1	Q01826			1
IPI00018956	ALX homeobox protein 1	ALX1	Q15699		1	
IPI00154553	Centrosomal protein of 76 kDa	CEP76	Q8TAP6		1	
IPI00215925	Glycine N-methyltransferase	GNMT	Q14749			1
IPI00006675	Multidrug resistance-associated protein 4	ABCC4	O15439			1
IPI00442551	CDNA FLJ26989 fis, clone SLV03395	ATP9B				1
IPI00027436	Tumor necrosis factor receptor superfamily member 16 precursor	NGFR	P08138			1
IPI00444208	CDNA FLJ45771 fis, clone NETRP2003103	RNF44			1	
IPI00166190	Isoform 1 of Uncharacterized protein C19orf19	C19orf19	Q3SX64			1
IPI00398709	Isoform 1 of Cation channel sperm-associated protein 4	CATSPER4	Q7RTX7		1	1
IPI00012869	Isoform A of Mothers against decapentaplegic homolog 6	SMAD6	O43541		1	1
IPI00827643	HRV Fab 027-VL (Fragment)	-			1	1
IPI00001641	Origin recognition complex subunit 6	ORC6L	Q9Y5N6		1	
IPI00216484	Isoform 2 of LisH domain-containing protein ARMC9	ARMC9	Q7Z3E5		1	

Table S1.

Number of unique peptides identified

IPI	Protein name	Gene symbol	Swiss Prot ID	Normal	CFS	nPTLS
IPI00170961	Isoform 2 of WD repeat-containing protein 16	WDR16	Q8N1V2		1	
IPI00877627	nuclear factor of activated T-cells 5 isoform d	NFAT5			1	1
IPI00154567	Isoform 2 of Spermatogenesis-associated protein 20 precursor	SPATA20	Q8TB22			1
IPI00169276	Trypsinogen C	-			5	5
IPI00786880	Myosin-XVB	-	Q96JP2		1	
IPI00152722	Protein CCDC35	CCDC35	Q8TEE6		1	
IPI00298447	Probable methyltransferase TARBP1	TARBP1	Q13395		1	
IPI00008495	NADH-ubiquinone oxidoreductase chain 4	MT-ND4	P03905			1
IPI00179172	Isoform 2 of Liprin-beta-1	PPFIBP1	Q86W92			1
IPI00009375	3-hydroxyanthranilate 3,4-dioxygenase	HAAO	P46952		1	1
IPI00293200	OTTHUMP00000016423	PARC			1	
IPI00220736	sialidase 3	NEU3	Q9UQ49		1	1
IPI00872928	Several ankyrin repeat protein transcript variant 2 (Fragment)	ANKRD42				1
IPI00412647	CDNA: FLJ21792 fis, clone HEP00441	THADA				1
IPI00456604	Protein FAM19A1 precursor	FAM19A1	Q7Z5A9		1	1
IPI00032929	Isoform 1 of Thrombospondin type-1 domain-containing protein 1 precursor	THSD1	Q9NS62			1
IPI00550272	Integrator complex subunit 10	INTS10	Q9NVR2		1	
IPI00154451	MMS19 nucleotide excision repair homolog	MMS19	Q96T76			1
IPI00186145	Isoform 1 of Protein phosphatase 1L	PPM1L	Q5SGD2			1
IPI00514197	similar to Nonhistone chromosomal protein HMG-17	LOC648822			1	
IPI00374293	similar to high-mobility group box 3	LOC729595			1	1
IPI00386364	Olfactory receptor 2L2	OR2L2	Q8NH16			1
IPI00013438	Immunoglobulin lambda-like polypeptide 1 precursor	IGLL1	P15814		2	2
IPI00218823	Isoform 1 of WW domain-binding protein 7	MLL4	Q9UMN6			1
IPI00156804	cDNA FLJ77550, highly similar to Homo sapiens zinc finger protein 665 (ZNF665), mRNA	ZNF665			1	
IPI00787587	similar to Temporarily Assigned Gene name family member	LOC729549			2	
IPI00384140	Full-length cDNA clone CS0DA005YA22 of Neuroblastoma of Homo sapiens (Fragment)	ESR2			1	
IPI00414784	Isoform 1 of CMRF35-H antigen precursor	CD300A	Q9UGN4		1	1

Table S1.

Number of unique peptides identified

IPI	Protein name	Gene symbol	Swiss Prot ID	Normal	CFS	nPTLS
IPI00410564	CDNA FLJ45248 fis, clone BRHIP2006819	FLJ45248			1	
IPI00023586	Isoform 1 of Sentrin-specific protease 6	SENP6	Q9GZR1		1	
IPI00028579	Transcription initiation factor TFIID subunit 6	TAF6	P49848		1	
IPI00060379	Integrator complex subunit 12	INTS12	Q96CB8			1
IPI00788118	similar to phosphodiesterase 4D interacting protein isoform 2	LOC727942			1	1
IPI00444706	CDNA FLJ45007 fis, clone BRAWH3012005	KALRN				1
IPI00021458	EH domain-containing protein 3	EHD3	Q9NZN3		1	1
IPI00216710	Isoform 1 of Nuclear receptor ROR-gamma	RORC	P51449			1
IPI00382490	Ig heavy chain V-III region BUR	-	P01773		1	2
IPI00411690	Isoform 3 of La-related protein 1	LARP1	Q6PKG0			1
IPI00295519	Isoform 1 of Uncharacterized protein C3orf17	C3orf17	Q6NW34			1
IPI00514153	transmembrane and tetratricopeptide repeat containing 4 isoform 1	TMTC4	Q5T4D3			1
IPI00398728	Isoform 1 of Retinitis pigmentosa 1-like 1 protein	RP1L1	Q8IWN7			1
IPI00253323	Ankyrin repeat domain-containing protein 57	ANKRD57	Q53LP3		1	
IPI00299554	Kinesin-like protein KIF14	KIF14	Q15058		1	
IPI00166361	Putative uncharacterized protein FLJ33590	FLJ33590			1	
IPI00021473	59 kDa protein	CBFA2T3				2
IPI00745775	Similar to PR domain containing 4	-				1
IPI00016703	24-dehydrocholesterol reductase precursor	DHCR24	Q15392			2
IPI00013880	Semaphorin-5A precursor	SEMA5A	Q13591			1
IPI00010157	S-adenosylmethionine synthetase isoform type-2	MAT2A	P31153			1
IPI00048230	Neurexophilin-1 precursor	NXPH1	P58417		3	4
IPI00794307	hypothetical protein	LOC729505				1
IPI00015782	UPF0171 protein C16orf35	C16orf35	Q12980			1
IPI00011652	Isoform Efs1 of Embryonal Fyn-associated substrate	EFS	O43281			1
IPI00294810	CDNA FLJ10824 fis, clone NT2RP4001086 (Fragment)	FAM21A			1	
IPI00418408	Cytochrome P450	CYP2D7P1			1	
IPI00028881	NADH dehydrogenase [ubiquinone] 1 alpha subcomplex subunit 3	NDUFA3	O95167			2
IPI00168627	Uncharacterized protein CXorf20	CXorf20	Q8NDZ0		1	

Table S1.

IPI	Protein name	Gene symbol	Swiss Prot ID	Number of unique peptides identified: Normal	CFS	nPTLS
IPI00413385	Inter-alpha-trypsin inhibitor heavy chain H5-like protein precursor	ITIH5L	Q6UXX5			1
IPI00026952	Plakophilin-3	PKP3	Q9Y446		1	
IPI00747758	Uncharacterized protein HSPG2 (Fragment)	HSPG2			4	3
IPI00014852	Isoform 1 of Phosphoglucomutase-like protein 5	PGM5	Q15124		1	
IPI00022334	Ornithine aminotransferase, mitochondrial precursor	OAT	P04181			1
IPI00023617	Zinc finger protein 197	ZNF197	O14709			1
IPI00332628	Otopetrin-3	OTOP3	Q7RTS5		1	
IPI00376317	Isoform 1 of Enhancer of mRNA-decapping protein 4	EDC4	Q6P2E9		1	1
IPI00642798	Nucleolar autoantigen No55	SC65	Q92791			1
IPI00383895	CDNA FLJ40559 fis, clone THYMU2002910	PRDM11				1
IPI00152157	Isoform 1 of Zinc finger protein 509	ZNF509	Q6ZSB9			2
IPI00296803	30 kDa protein	TCP11L2				1
IPI00296830	Isoform 1 of Leucine zipper protein 1	LUZP1	Q86V48		1	
IPI00168056	Zinc finger and BTB domain-containing protein 38	ZBTB38	Q8NAP3			1
IPI00306127	THUMP domain-containing protein 3	THUMPD3	Q9BV44		1	
IPI00026105	Isoform SCPx of Non-specific lipid-transfer protein	SCP2	P22307			1
IPI00385255	Ig lambda chain V-II region NIG-84	-	P04209			1
IPI00022881	Isoform 1 of Clathrin heavy chain 2	CLTCL1	P53675		1	
IPI00328178	Isoform 3 of TOM1-like protein 2	TOM1L2	Q6ZVM7			1
IPI00397015	A.T hook DNA-binding motif-containing protein 1	AHDC1	Q5TGY3		1	
IPI00029184	Hyaluronan and proteoglycan link protein 2 precursor	HAPLN2	Q9GZV7		1	
IPI00795917	126 kDa protein	CXorf45			1	
IPI00027144	Cytochrome b561	CYB561	P49447			1
IPI00815976	Transmembrane protein 120B	TMEM120B	A0PK00		1	
IPI00382671	Isoform 6 of C-type lectin domain family 4 member M	CLEC4M	Q9H2X3		1	
IPI00217688	Uncharacterized protein C12orf60	C12orf60	Q5U649			1
IPI00018303	Isoform 2 of Protocadherin-11 X-linked precursor	PCDH11X	Q9BZA7		1	
IPI00176210	hypothetical protein LOC133558	FLJ40243			1	
IPI00220126	Isoform EP2B of Sperm-associated antigen 11B precursor	SPAG11B	Q08648			1
IPI00166153	Protein KIAA0082	KIAA0082	Q8N1G2		1	
IPI00007757	Isoform 1 of Zinc finger protein Helios	IKZF2	Q9UKS7			1

Table S1.

Number of unique peptides identified

IPI	Protein name	Gene symbol	Swiss Prot ID	Normal	CFS	nPTLS
IPI00300621	Isoform 2B2 of Synaptojanin-2	SYNJ2	O15056		1	
IPI00328400	Zygote arrest protein 1	ZAR1	Q86SH2			1
IPI00032358	Nuclear envelope pore membrane protein POM 121	POM121	Q9Y2N3		1	1
IPI00306166	germ cell associated 1 isoform 2	GSG1			1	
IPI00465147	Isoform 1 of Uncharacterized protein C9orf97	C9orf97	Q5T7W7			1
IPI00401002	Zinc finger protein 740	ZNF740	Q8NDX6		1	
IPI00607829	Isoform 1 of Ret finger protein-like 2	RFPL2	O75678		1	
IPI00402280	Isoform 1 of Small leucine-rich proteoglycan family member LOC150356 precursor	CHADL	Q6NUI6		1	
IPI00333016	Isoform 3 of DnaJ homolog subfamily C member 11	DNAJC11	Q9NVH1			1
IPI00006800	Isoform Long of Autophagy protein 5	ATG5	Q9H1Y0		1	
IPI00014897	Isoform 2 of 1-phosphatidylinositol-4,5-bisphosphate phosphodiesterase beta-4	PLCB4	Q15147			1
IPI00181279	hypothetical protein	LRRC37A4				1
IPI00294398	Isoform 1 of Hydroxyacyl-coenzyme A dehydrogenase, mitochondrial precursor	HADH	Q16836		1	1
IPI00218559	Gamma-sarcoglycan	SGCG	Q13326		1	
IPI00028980	Uncharacterized protein KIAA0133	KIAA0133	Q14146		2	
IPI00011894	MRNA clone with similarity to L-glycerol-3-phosphate:NAD oxidoreductase and albumin gene sequences	-				1
IPI00794248	47 kDa protein	-				1
IPI00479897	Chromosome 9 open reading frame 75	C9orf75			1	
IPI00008868	Microtubule-associated protein 1B	MAP1B	P46821		1	1
IPI00216003	Cullin-5	CUL5	Q93034		1	
IPI00445796	CDNA FLJ43383 fis, clone OCBBF2006058, highly similar to Homo sapiens acyl-Coenzyme A dehydrogenase-8 mRNA	ACAD8			1	
IPI00514774	Isoform 1 of PAB-dependent poly(A)-specific ribonuclease subunit 3	PAN3	Q58A45			1
IPI00307733	Isoform 1 of Histone-lysine N-methyltransferase SETD2	SETD2	Q9BYW2			1
IPI00742114	similar to tripartite motif protein 11	LOC653978			1	
IPI00007127	Isoform 1 of Dipeptidase 2 precursor	DPEP2	Q9H4A9		2	2
IPI00218896	Alcohol dehydrogenase 1A	ADH1A	P07327			1
IPI00414973	FAST kinase domain-containing protein 5	FASTKD5	Q7L8L6			1

Table S1.

IPI	Protein name	Gene symbol	Swiss Prot ID	Number of unique peptides identified		
				**Normal**	**CFS**	**nPTLS**
IPI00002441	Syndecan-1 precursor	SDC1	P18827			1
IPI00413272	Isoform 3 of Mediator of RNA polymerase II transcription subunit 23	MED23	Q9ULK4			1
IPI00023164	Potassium/sodium hyperpolarization-activated cyclic nucleotide-gated channel 4	HCN4	Q9Y3Q4		1	
IPI00299853	Protocadherin beta 16 precursor	PCDHB16	Q9NRJ7			1
IPI00012480	FKSG42	RACGAP1P			1	
IPI00004538	similar to nascent polypeptide-associated complex alpha polypeptide isoform 1	NACAD	O15069		1	
IPI00297462	Uncharacterized protein C1orf65	C1orf65	Q8N715		1	
IPI00735857	similar to ATP-binding cassette sub-family D member 1	LOC651123				1
IPI00515034	87 kDa protein	KIFAP3			1	
IPI00430792	Isoform 1 of Protein ERGIC-53-like precursor	LMAN1L	Q9HAT1		2	
IPI00793454	7 kDa protein	USP19			1	
IPI00554701	Cytochrome b-c1 complex subunit 9	UCRC	Q9UDW1		1	1
IPI00647254	7 kDa protein	-				1
IPI00015877	Transmembrane protein 59-like precursor	TMEM59L	Q9UK28		3	5
IPI00028392	specifically androgen-regulated protein isoform 1	C1orf116			1	
IPI00387025	Ig kappa chain V-I region DEE	-	P01597		2	2
IPI00385495	Transmembrane protein 153	LMF2			1	
IPI00328118	Sperm-associated antigen 5	SPAG5	Q96R06			1
IPI00017726	Isoform 1 of 3-hydroxyacyl-CoA dehydrogenase type-2	HSD17B10	Q99714		1	
IPI00094740	Isoform 1 of RING finger protein 31	RNF31	Q96EP0			1
IPI00383318	PRO2272	-				1
IPI00292470	dapper, antagonist of beta-catenin, homolog 2	DACT2				1
IPI00791509	Neural precursor cell expressed, developmentally down-regulated 1	NEDD1			1	
IPI00215884	Isoform ASF-1 of Splicing factor, arginine/serine-rich 1	SFRS1	Q07955			1
IPI00250716	Isoform B of Bromodomain and WD repeat-containing protein 1	BRWD1	Q9NSI6		1	1
IPI00289931	Isoform 1 of Mucosal addressin cell adhesion molecule 1 precursor	MADCAM1	Q13477		1	
IPI00013488	Isoform Alpha-2 of N-chimaerin	CHN1	P15882			1
IPI00218687	Isoform 10 of Voltage-dependent T-type calcium channel subunit alpha-1G	CACNA1G	O43497			1

Table S1.

Number of unique peptides identified

IPI	Protein name	Gene symbol	Swiss Prot ID	Normal	CFS	nPTLS
IPI00442601	CDNA FLJ26817 fis, clone PRS06446	-			1	
IPI00002899	Lymphocyte antigen 6E precursor	LY6E	Q16553		1	1
IPI00012891	Phosphorylase b kinase gamma catalytic chain, testis/liver isoform	PHKG2	P15735		1	
IPI00021338	Dihydrolipoyllysine-residue acetyltransferase component of pyruvate dehydrogenase complex, mitochondrial precursor	DLAT	P10515		1	
IPI00030909	BB1 family protein	-				1
IPI00552213	Isoform 6 of Disks large homolog 1	DLG1	Q12959			1
IPI00455083	Uncharacterized protein ENSP00000371558	-			1	
IPI00168877	helicase (DNA) B	HELB			1	
IPI00397358	Similar to ribosomal protein S27	-				1
IPI00021786	RAF proto-oncogene serine/threonine-protein kinase	RAF1	P04049			1
IPI00641214	177 kDa protein	ADCY10			1	
IPI00003438	DnaJ homolog subfamily C member 8	DNAJC8	O75937			1
IPI00290837	Isoform 2 of F-box/WD repeat-containing protein 10	FBXW10	Q5XX13		1	
IPI00398900	Homeobox protein HMX2	HMX2	A2RU54			1
IPI00031960	polymerase (RNA) I polypeptide A, 194kDa	POLR1A	O95602			1
IPI00006173	Isoform 1 of Cholesteryl ester transfer protein precursor	CETP	P11597		3	
IPI00298870	Transmembrane protein 1	TMEM1	P48553			1
IPI00009213	Isoform 1 of B-cell lymphoma/leukemia 11B	BCL11B	Q9C0K0		1	
IPI00018335	Isoform Flt1 of Vascular endothelial growth factor receptor 1 precursor	FLT1	P17948		2	
IPI00012902	synaptotagmin VII	SYT7	O43581		1	3
IPI00217051	Isoform 1 of Neuron navigator 3	NAV3	Q8IVL0		1	1
IPI00017991	jumonji domain containing 2D	JMJD2D	Q6B0I6			1
IPI00151366	Isoform TRPM6a of Transient receptor potential cation channel subfamily M member 6	TRPM6	Q9BX84			1
IPI00003421	T-brain-1 protein	TBR1	Q16650		1	
IPI00396314	Isoform 3 of CCR4-NOT transcription complex subunit 10	CNOT10	Q9H9A5		1	
IPI00328825	Isoform 1 of NEDD4-binding protein 2	N4BP2	Q86UW6			2
IPI00011488	Isoform 1 of Serine/threonine-protein kinase 4	STK4	Q13043			1
IPI00000106	Signal transducer and activator of transcription 4	STAT4	Q14765		1	

Table S1.

				Number of unique peptides identified		
**IPI**	**Protein name**	**Gene symbol**	**Swiss Prot ID**	**Normal**	**CFS**	**nPTLS**
IPI00339217	Ovochymase-1 precursor	OVCH1	Q7RTY7		1	
IPI00395631	Isoform 1 of E3 ubiquitin-protein ligase TRAF7	TRAF7	Q6Q0C0		1	
IPI00060569	Isoform 2 of Abhydrolase domain-containing protein 12	ABHD12	Q8N2K0			2
IPI00101405	Farnesyl diphosphate synthase	FDPS	P14324			1
IPI00044608	Isoform 1 of Protein KIAA1881	KIAA1881	Q96Q06		1	
IPI00455397	DMWD protein	DMWD			1	
IPI00786867	Dual Intracellular Von Willebrand factor domain A	DIVA				1
IPI00006904	Cell death regulator Aven	AVEN	Q9NQS1			1
IPI00827486	Rheumatoid factor light chain variable region precursor (Fragment)	-				2
IPI00853079	Isoform 1 of Uncharacterized protein C14orf166B	C14orf166B	Q0VAA2			1
IPI00444138	CDNA FLJ46074 fis, clone TESTI2001915, highly similar to Homo sapiens actin filament associated protein	AFAP1			1	
IPI00744557	kazrin isoform E	RP1-21O18.1			1	
IPI00152692	D-tyrosyl-tRNA(Tyr) deacylase 1	DTD1	Q8TEA8		1	
IPI00376213	Isoform 1 of G-protein coupled receptor 120	GPR120	Q5NUL3			1
IPI00478921	myelin protein zero	MPZ	P25189		1	2
IPI00397526	Isoform 1 of Myosin-10	MYH10	P35580		1	
IPI00010420	ADP/ATP translocase 4	SLC25A31	Q9H0C2			1
IPI00782966	Zinc finger protein 106 homolog	ZFP106	Q9H2Y7		1	1
IPI00640654	RAP1 GTPase activating protein	RAP1GAP			1	
IPI00795736	Fer-1-like 4	-			2	
IPI00791831	10 kDa protein	ADRBK1			1	
IPI00004419	Zinc finger protein 646	ZNF646	O15015			1
IPI00479904	Uncharacterized protein COL27A1	COL27A1			1	
IPI00847409	hypothetical protein	LOC440338				1
IPI00013256	Isoform 1 of Cleavage stimulation factor 64 kDa subunit	CSTF2	P33240		1	
IPI00005024	Isoform 1 of Myb-binding protein 1A	MYBBP1A	Q9BQG0			1
IPI00177890	54 kDa protein	SMARCD2				1
IPI00023635	Isoform 1 of Inositol monophosphatase 2	IMPA2	O14732			1
IPI00296563	GTP-binding protein GUF1 homolog	GUF1	Q8N442		1	1
IPI00816409	V<gamma>1 protein (Fragment)	-			1	2
IPI00008403	Carbohydrate sulfotransferase 7	CHST7	Q9NS84			1
IPI00412298	Isoform 1 of Serine/threonine-protein kinase ATR	ATR	Q13535			2
IPI00550069	Ribonuclease inhibitor	RNH1	P13489		1	3

Table S1.

IPI	Protein name	Gene symbol	Swiss Prot ID	Number of unique peptides identified		
				Normal	CFS	nPTLS
IPI00059279	Exocyst complex component 4	EXOC4	Q96A65		1	
IPI00299402	Pyruvate carboxylase, mitochondrial precursor	PC	P11498		1	
IPI00167639	Vacuolar proton pump subunit d 2	ATP6V0D2	Q8N8Y2		1	
IPI00304885	Centromere protein C 1	CENPC1	Q03188			1
IPI00784013	janus kinase 1	JAK1	P23458			1
IPI00216139	Isoform I of Septin-6	SEPT6	Q14141			1
IPI00289954	Heparan sulfate glucosamine 3-O-sulfotransferase 6	HS3ST6	Q96QI5			1
IPI00025861	Epithelial cadherin precursor	CDH1	P12830		2	3
IPI00018134	Isoform 4 of Protein SOLO	FLJ10357	Q8TER5			1
IPI00855742	sterile alpha motif domain containing 12 isoform a	SAMD12				1
IPI00415037	Isoform 12L of ADAM 12 precursor	ADAM12	O43184			1
IPI00411298	Connector enhancer of kinase suppressor of ras 3	CNKSR3	Q6P9H4			1
IPI00170855	tetratricopeptide repeat domain 19	TTC19	Q6DKK2		1	
IPI00031288	Claudin-14	CLDN14	O95500			1
IPI00445563	Putative uncharacterized protein MGC21675/FLJ43787	C4orf42	Q0VAR9		1	
IPI00026941	Serine protease 23 precursor	PRSS23	O95084		1	1
IPI00867582	Similar to SCAN domain-containing protein 2. Isoform 2	ZNF496				1
IPI00418885	Epididymal-specific lipocalin-10 precursor	LCN10	Q6JVE6			1
IPI00006152	Isoform 1 of Sphingosine 1-phosphate receptor Edg-8	EDG8	Q9H228		1	1
IPI00166465	hypothetical protein LOC221416	C6orf223				2
IPI00795992	Hypothetical short protein	-				1
IPI00884334	Conserved hypothetical protein	LOC729968			1	
IPI00293613	Serine/threonine-protein kinase TBK1	TBK1	Q9UHD2		1	1
IPI00007910	Isoform 1 of Sodium-dependent phosphate transport protein 2B	SLC34A2	O95436		1	
IPI00083708	BAT2-iso	BAT2D1			1	
IPI00847373	hypothetical protein LOC54627	KIAA1383	Q9P2G4			1
IPI00030070	Gap junction beta-5 protein	GJB5	O95377			1
IPI00418238	Isoform 1 of HIV-1 Rev-binding protein-like protein	HRBL	O95081			1
IPI00328715	Protein LYRIC	MTDH	Q86UE4		1	
IPI00293409	Zinc finger protein 81	ZNF81	P51508			1
IPI00003927	40 kDa peptidyl-prolyl cis-trans isomerase	PPID	Q08752			1
IPI00024801	E3 ubiquitin-protein ligase NRDP1	RNF41	Q9H4P4			1
IPI00060969	Isoform 1 of Protein FAM55B	FAM55B	Q96DL1		1	1

Table S1.

Number of unique peptides identified

IPI	Protein name	Gene symbol	Swiss Prot ID	Normal	CFS	nPTLS
IPI00290035	Isoform 1 of Protocadherin-15 precursor	PCDH15	Q96QU1		1	
IPI00103356	Uncharacterized protein ITGB2	ITGB2				1
IPI00385511	Uncharacterized protein TNRC6B	TNRC6B			1	
IPI00604798	Bitter taste receptor T2R2 (Fragment)	-				1
IPI00306929	Isoform 2 of Myosin-XVIIIb	MYO18B	Q8IUG5		2	1
IPI00179415	Isoform 1 of Serine/threonine-protein phosphatase 2B catalytic subunit alpha isoform	PPP3CA	Q08209		2	1
IPI00419215	alpha-2-macroglobulin-like 1	A2ML1			1	1
IPI00006561	Isoform 1 of Protein KIAA0317	KIAA0317	O15033		1	
IPI00375294	Laminin subunit alpha-1 precursor	LAMA1	P25391		1	
IPI00013887	Beta-galactoside alpha-2,6-sialyltransferase 1	ST6GAL1	P15907			1
IPI00006653	Calcium-transporting ATPase type 2C member 2	ATP2C2	O75185		1	1
IPI00375325	Keratin-associated protein 10-10	KRTAP10-10	P60014		1	
IPI00640293	23 kDa protein	TIMP3			1	
IPI00028122	Isoform 1 of PC4 and SFRS1-interacting protein	PSIP1	O75475		1	1
IPI00235003	Tumor necrosis factor receptor superfamily, member 6 isoform 1 variant (Fragment)	FAS			1	
IPI00221101	Sucrase-isomaltase, intestinal	SI	P14410		1	1
IPI00000656	Isoform 1 of Uncharacterized protein KIAA0892 precursor	KIAA0892	Q9Y6X3		2	
IPI00010085	Zinc finger protein PLAGL2	PLAGL2	Q9UPG8		1	1
IPI00216702	Isoform 1 of ATP-binding cassette sub-family A member 9	ABCA9	Q8IUA7		1	
IPI00786926	Myosin-reactive immunoglobulin heavy chain variable region (Fragment)	IGHV1-69			5	5
IPI00384971	hypothetical protein LOC643155	DKFZP686E2158				1
IPI00807406	hypothetical protein isoform 1	LOC285346			1	
IPI00167930	ring finger protein 190	MARCH10			1	
IPI00157820	Thioredoxin reductase 2 isoform 1 variant	TXNRD2			1	1
IPI00020017	Adipose most abundant gene transcript 2 protein	C10orf116	Q15847		1	1
IPI00470657	Anti-colorectal carcinoma heavy chain	-			1	
IPI00413868	hypothetical protein LOC79632 isoform 1	C6orf60			1	
IPI00066367	Endothelial cells scavenger receptor precursor	SCARF1	Q14162		1	
IPI00025721	COP9 signalosome complex subunit 3	COPS3	Q9UNS2			1
IPI00020228	Frizzled-6 precursor	FZD6	O60353		1	1

Table S1.

				Number of unique peptides identified		
**IPI**	**Protein name**	**Gene symbol**	**Swiss Prot ID**	**Normal**	**CFS**	**nPTLS**
IPI00442002	Isoform 3 of Signal peptide, CUB and EGF-like domain-containing protein 2 precursor	SCUBE2	Q9NQ36		5	2
IPI00397740	zinc finger protein 749	ZNF749	O43361		1	
IPI00300426	Isoform 1 of Tetratricopeptide repeat protein 29	TTC29	Q8NA56		1	
IPI00291878	Pulmonary surfactant-associated protein D precursor	SFTPD	P35247		2	1
IPI00855747	hypothetical protein LOC57481	RP13-347D8.3	Q9ULL0			1
IPI00307259	DnaJ homolog subfamily C member 13	DNAJC13	O75165		1	
IPI00445054	CDNA FLJ44715 fis, clone BRACE3021430	FLJ44715				1
IPI00030360	RUN domain-containing protein 2A	RUNDC2A	Q9HA26			1
IPI00220503	dynactin 2	DCTN2	Q13561			1
IPI00550571	Keratinocytes-associated transmembrane protein 2 precursor	C5orf15	Q8NC54		1	1
IPI00386113	CDNA: FLJ23412 fis, clone HEP20516	C20orf59			1	
IPI00153060	Angiopoietin-related protein 4 precursor	ANGPTL4	Q9BY76		1	
IPI00020354	Interleukin-13 receptor alpha-1 chain precursor	IL13RA1	P78552		1	
IPI00328159	Isoform 1 of Uncharacterized protein C1orf88	C1orf88	Q8TCI5			1
IPI00177888	Isoform 1 of Leukocyte receptor cluster member 8	LENG8	Q96PV6		1	
IPI00003881	Heterogeneous nuclear ribonucleoprotein F	HNRPF	P52597			1
IPI00101267	Isoform 1 of Uncharacterized protein C19orf60	C19orf60	Q96EN9		1	
IPI00003990	Isoform 2 of Valacyclovir hydrolase precursor	BPHL	Q86WA6			1
IPI00847485	Similar to PFIV protein	-				1
IPI00739927	similar to vasoactive intestinal peptide receptor 2	LOC645464			1	
IPI00010869	Agouti-related protein precursor	AGRP	O00253		1	1
IPI00782965	huntingtin interacting protein 1	HIP1	O00291		1	
IPI00103749	Isoform 2 of Triggering receptor expressed on myeloid cells 2 precursor	TREM2	Q9NZC2		1	3
IPI00293616	ATP-dependent RNA helicase DDX3Y	DDX3Y	O15523		2	1
IPI00007300	BTB/POZ domain-containing protein 3	BTBD3	Q9Y2F9			1
IPI00019517	Wilms tumor 1 isoform D	WT1	P19544			2
IPI00387109	Ig kappa chain V-II region FR	-	P01615		1	1
IPI00217490	Isoform 1 of Fibronectin type III domain-containing protein 3B	FNDC3B	Q53EP0			1
IPI00848330	Conserved hypothetical protein	LOC285144				1

Table S1.

IPI	Protein name	Gene symbol	Swiss Prot ID	Number of unique peptides identified<br>Normal	CFS	nPTLS
IPI00444375	Rh blood group, CcEe antigens	RHCE			1	
IPI00000787	Isoform LMP2.L of Proteasome subunit beta type-9 precursor	PSMB9	P28065			1
IPI00004379	Homeobox protein HMX1	HMX1	Q9NP08			1
IPI00794880	Isoform 1 of Chromodomain-helicase-DNA-binding protein 7	CHD7	Q9P2D1		1	
IPI00419791	Isoform 1 of Arginine/serine-rich coiled-coil protein 2	RSRC2	Q7L4I2		1	
IPI00375820	Interferon-induced transmembrane protein 5	IFITM5	A6NNB3			1
IPI00100106	resistance to inhibitors of cholinesterase 8 homolog A	RIC8A	Q9NPQ8		1	
IPI00302647	Isoform 1 of Coiled-coil and C2 domain-containing protein 1A	CC2D1A	Q6P1N0		1	2
IPI00216593	Isoform NOH-1LV of NADPH oxidase homolog 1	NOX1	Q9Y5S8		1	
IPI00411635	180 kDa protein	-				1
IPI00395667	Interferon-related IFRD2 (PC4-B) protein	NAT6	Q12894		1	
IPI00402291	Uncharacterized protein ENSP00000308976	LOC402117			1	1
IPI00008708	Ribosomal L1 domain-containing protein 1	RSL1D1	O76021		1	
IPI00795566	6 kDa protein	-				1
IPI00217117	Isoform 1 of Glycerate kinase	GLYCTK	Q8IVS8			1
IPI00300086	Nicotinate-nucleotide pyrophosphorylase	QPRT	Q15274			1
IPI00235167	Profilin-3	PFN3	P60673			1
IPI00026126	Mammaglobin-B precursor	SCGB2A1	O75556			1
IPI00748649	25 kDa protein	-			1	
IPI00100362	erythrocyte membrane protein band 4.1 like 4B isoform 1	EPB41L4B	Q9H329		1	
IPI00829845	Uncharacterized protein ENSP00000375026	-			2	2
IPI00219898	Isoform 2 of Nephrin precursor	NPHS1	O60500		1	
IPI00220167	Isoform 1 of Putative methyltransferase NSUN7	NSUN7	Q8NE18			1
IPI00872098	Zinc finger protein Pegasus	IKZF5	Q9H5V7		1	
IPI00155168	Leukocyte common antigen precursor (EC 3.1.3.48) (L-CA) (T200) (CD45 antigen). Isoform 2	PTPRC				1
IPI00043294	Isoform 1 of Uncharacterized protein C7orf26	C7orf26	Q96N11		1	
IPI00152535	Chromodomain-helicase-DNA-binding protein 5	CHD5	Q8TDI0		1	

Table S1.

Number of unique peptides identified

IPI	Protein name	Gene symbol	Swiss Prot ID	Normal	CFS	nPTLS
IPI00384268	XRCC6BP1 protein	XRCC6BP1				1
IPI00217507	Neurofilament medium polypeptide	NEFM	P07197		1	
IPI00015159	Ephrin-A2 precursor	EFNA2	O43921		1	2
IPI00216356	Isoform 2 of RasGAP-activating-like protein 1	RASAL1	O95294		1	
IPI00299507	Condensin complex subunit 2	NCAPH	Q15003			1
IPI00290562	Nuclear factor interleukin-3-regulated protein	NFIL3	Q16649		1	
IPI00645729	62 kDa protein	C1QTNF8			1	
IPI00445643	CDNA FLJ43684 fis, clone TBAES2001492	-				1
IPI00165591	Isoform 4 of Transmembrane channel-like protein 6	TMC6	Q7Z403			1
IPI00010334	Isoform 3 of Inactive phospholipase C-like protein 2	PLCL2	Q9UPR0			1
IPI00006003	CD83 antigen precursor	CD83	Q01151		1	
IPI00166528	Isoform 3 of Rapamycin-insensitive companion of mTOR	RICTOR	Q6R327		1	
IPI00221325	E3 SUMO-protein ligase RanBP2	RANBP2	P49792		1	
IPI00152149	CENP-B, N-terminal DNA-binding domain containing protein	-				1
IPI00029643	Isoform 2 of Mitogen-activated protein kinase kinase kinase MLT	ZAK	Q9NYL2			1
IPI00084684	similar to Zinc finger protein 469	ZNF469			1	
IPI00000875	Elongation factor 1-gamma	EEF1G	P26641			1
IPI00215948	Isoform 1 of Catenin alpha-1	CTNNA1	P35221			1
IPI00217975	Lamin-B1	LMNB1	P20700		2	2
IPI00643809	Protein	CNTNAP3B			1	
IPI00022918	Isoform 2 of Protein FAM125B	FAM125B	Q9H7P6			1
IPI00164776	Similar to RIKEN cDNA A230078I05 gene	TMEM198			1	
IPI00787765	similar to solute carrier family 35, member F4	SLC35F4				1
IPI00023410	Integrin alpha-8 precursor	ITGA8	P53708		1	
IPI00787593	similar to CG33300-PA	LOC730407			1	
IPI00007208	Probable ATP-dependent RNA helicase DDX41	DDX41	Q9UJV9			1
IPI00180781	Isoform 1 of Mixed lineage kinase domain-like protein	MLKL	Q8NB16		1	
IPI00339309	Isoform 1 of Proto-oncogene DBL	MCF2	P10911		1	
IPI00855922	Isoform 4 of DENN domain-containing protein 3	DENND3	A2RUS2			1
IPI00843923	Phosphodiesterase PDE7B2	PDE7B			1	
IPI00640865	Integrin beta-like protein 1 precursor	ITGBL1			1	

Table S1.

IPI	Protein name	Gene symbol	Swiss Prot ID	Normal	CFS	nPTLS
				Number of unique peptides identified		
IPI00029144	Isoform PR130 of Serine/threonine-protein phosphatase 2A regulatory subunit B" subunit alpha	PPP2R3A	Q06190		1	
IPI00328840	THO complex subunit 4	THOC4	Q86V81		1	3
IPI00007074	Tyrosyl-tRNA synthetase, cytoplasmic	YARS	P54577			2
IPI00031056	Zinc finger MYND domain-containing protein 15	ZMYND15	Q9H091			1
IPI00175439	F-box only protein 43	FBXO43	Q4G163		1	
IPI00646485	Protein	EFCAB2			1	
IPI00641614	47 kDa protein	AKAP8L				1
IPI00007729	Isoform 1 of Nucleolar protein 7	NOL7	Q9UMY1		1	1
IPI00002134	26S proteasome non-ATPase regulatory subunit 5	PSMD5	Q16401			1
IPI00023105	Peptidylglycine alpha-amidating monooxygenase COOH-terminal interactor	PAMCI	O75901		1	
IPI00056040	Neuritin 1-like	NRN1L				1
IPI00856045	AHNAK nucleoprotein 2	AHNAK2				1
IPI00217955	H1 histone family, member O, oocyte-specific	H1FOO			1	1
IPI00009329	Utrophin	UTRN	P46939			1
IPI00218235	dehydrogenase/reductase member 2 isoform 2	DHRS2	Q13268			1
IPI00003375	Isoform HCC-1 of C-C motif chemokine 14 precursor	CCL14	Q16627		2	1
IPI00301144	Isoform 1 of Sulfatase-modifying factor 1 precursor	SUMF1	Q8NBK3		1	
IPI00297315	Alpha-2B adrenergic receptor	ADRA2B	P18089			1
IPI00439935	UDP-glucuronosyltransferase 1-4 precursor	UGT1A4	P22310		1	
IPI00001753	Myosin-4	MYH4	Q9Y623		1	
IPI00107855	Hephaestin precursor	HEPH	Q9BQS7			1
IPI00401829	Putative uncharacterized protein ENST00000281581	-	P0C221		1	
IPI00217662	Uncharacterized protein C11orf35	C11orf35	Q8IXW0			1
IPI00386119	Isoform 5 of Splicing factor 1	SF1	Q15637			1
IPI00025489	Serine/threonine-protein kinase MAK	MAK	P20794			1
IPI00027774	THAP domain-containing protein 2	THAP2	Q9H0W7			1
IPI00027415	Isoform 1 of Probable ATP-dependent RNA helicase DHX36	DHX36	Q9H2U1			1
IPI00056324	Isoform 1 of Hermansky-Pudlak syndrome 3 protein	HPS3	Q969F9		1	
IPI00220493	U6 snRNA-associated Sm-like protein LSm5	LSM5	Q9Y4Y9		1	

Table S1.

				Number of unique peptides identified		
**IPI**	**Protein name**	**Gene symbol**	**Swiss Prot ID**	**Normal**	**CFS**	**nPTLS**
IPI00169377	Isoform 1 of GAS2-like protein 2	GAS2L2	Q8NHY3		2	
IPI00386390	Olfactory receptor 6N2	OR6N2	Q8NGY6		1	
IPI00176125	Collagen XXIX alpha 1	COL29A1				1
IPI00044761	Pseudouridylate synthase 7 homolog	PUS7	Q96PZ0		1	
IPI00032342	TRIP12 protein	TRIP12			1	
IPI00016600	Tetraspanin-7	TSPAN7	P41732		1	1
IPI00418614	67 kDa protein	-				1
IPI00016046	Protein MGR2 homolog	C20orf52	P60602			1
IPI00307536	Isoform 1 of Testis-expressed sequence 11 protein	TEX11	Q8IYF3		1	
IPI00244812	Tetratricopeptide repeat protein 6	TTC6	Q86TZ1		1	
IPI00030487	CDNA FLJ30993 fis, clone HLUNG1000064, weakly similar to KARYOGAMY PROTEIN KAR4	KIAA1627			1	
IPI00787443	similar to Collagen alpha-2(XI) chain precursor	LOC730037			1	
IPI00013371	Protein sprouty homolog 3	SPRY3	O43610		1	
IPI00414259	Isoform 2 of Pregnancy-specific beta-1-glycoprotein 4 precursor	PSG4	Q00888		1	
IPI00002841	Sodium/hydrogen exchanger 2	SLC9A2	Q9UBY0			1
IPI00410590	Isoform 2 of LSM14 protein homolog A	LSM14A	Q8ND56		1	
IPI00472712	Coiled-coil domain containing 7	CCDC7				1
IPI00306851	Low-density lipoprotein receptor-related protein 4 precursor	LRP4	O75096		2	2
IPI00061355	Isoform 3 of Protein ZNF673	ZNF673	Q5JUW0			1
IPI00445804	Uncharacterized protein ENSP00000374264	DKFZp761B107				1
IPI00790813	8 kDa protein	RAD51AP1				1
IPI00061780	Isoform 1 of E3 ubiquitin-protein ligase Itchy homolog	ITCH	Q96J02			1
IPI00789856	tubulin tyrosine ligase-like family, member 3	TTLL3			1	
IPI00005806	ST7 form 2 splice variant a	ST7			1	1
IPI00023330	Isoform 2 of LisH domain and HEAT repeat-containing protein KIAA1468	KIAA1468	Q9P260		1	
IPI00426267	Isoform 1 of Leucine-rich repeat-containing protein 7	LRRC7	Q96NW7		1	1
IPI00645614	Isoform 2 of Cadherin-3 precursor	CDH3	P22223		1	
IPI00744574	Similar to Tropomyosin alpha-4 chain	-			1	
IPI00737735	similar to cell division cycle 10 isoform 1	LOC646913			1	
IPI00020729	Insulin receptor substrate 4	IRS4	O14654		1	1
IPI00030099	Adenylate cyclase type 9	ADCY9	O60503			1
IPI00030648	Isoform 1 of Zinc finger FYVE domain-containing protein 9	ZFYVE9	O95405		2	

Table S1.

Number of unique peptides identified

IPI	Protein name	Gene symbol	Swiss Prot ID	Normal	CFS	nPTLS
IPI00514533	Uncharacterized protein SYCP2L	SYCP2L				2
IPI00016095	Transcription termination factor, mitochondrial precursor	MTERF	Q99551			1
IPI00065415	Isoform 1 of Coiled-coil domain-containing protein 138	CCDC138	Q96M89		1	
IPI00004521	Ewing's tumor-associated antigen 1	ETAA1	Q9NY74			1
IPI00171525	Sentrin-specific protease 3	SENP3	Q9H4L4			1
IPI00376206	Isoform 2 of 17-beta hydroxysteroid dehydrogenase 13 precursor	HSD17B13	Q7Z5P4		1	
IPI00011589	Isoform A of Septin-4	SEPT4	O43236		1	
IPI00373824	Testis serine protease 1 precursor	TESSP1				1
IPI00847644	structural maintenance of chromosomes flexible hinge domain containing 1	SMCHD1			1	1
IPI00032158	Isoform 2 of NMDA receptor-regulated protein 1	NARG1	Q9BXJ9		1	
IPI00013468	Mitotic checkpoint protein BUB3	BUB3	O43684		1	
IPI00003084	Isoform 1 of Dr1-associated corepressor	DRAP1	Q14919		1	1
IPI00017231	Neurensin-2	NRSN2	Q9GZP1			1
IPI00745955	EBNA1 binding protein 2	EBNA1BP2	Q99848		1	
IPI00005465	Testis-specific Y-encoded-like protein 2	TSPYL2	Q9H2G4		1	1
IPI00256974	Osteocrin precursor	OSTN	P61366		1	1
IPI00299729	Transcobalamin-1 precursor	TCN1	P20061			1
IPI00157237	Isoform 1 of MAGUK p55 subfamily member 4	MPP4	Q96JB8		1	
IPI00009040	Ornithine decarboxylase antizyme 3	OAZ3	Q9UMX2		1	
IPI00383774	Mih1/Tx protein	CASP4			1	
IPI00022290	Beta-defensin 1 precursor	DEFB1	P60022			1
IPI00007444	Epiphycan precursor	EPYC	Q99645			1
IPI00167014	EID2 protein (Fragment)	EID2				1
IPI00182774	SH3YL1 protein	SH3YL1			1	
IPI00007327	Isoform 1 of Tapasin precursor	TAPBP	O15533			1
IPI00244346	Troponin I, cardiac muscle	TNNI3	P19429			1
IPI00217691	Isoform 1 of EF-hand domain-containing protein C17orf57	C17orf57	Q8IY85		1	
IPI00009066	similar to protein tyrosine phosphatase, receptor type, Q isoform 1 precursor	PTPRQ				1
IPI00303195	Syncoilin-1	SYNC1	Q9H7C4			1
IPI00012622	Isoform 1 of Rho GTPase-activating protein 20	ARHGAP20	Q9P2F6		1	1
IPI00015473	Excitatory amino acid transporter 1	SLC1A3	P43003		1	1
IPI00514843	KIAA0082	KIAA0082			1	
IPI00216346	Isoform 2 of Nucleolar protein 3	NOL3	O60936		1	
IPI00031812	Nuclease sensitive element-binding protein 1	YBX1	P67809			1

Table S1.

Number of unique peptides identified

IPI	Protein name	Gene symbol	Swiss Prot ID	Normal	CFS	nPTLS
IPI00644968	Similar to Heterogeneous nuclear ribonucleoprotein A1	-			1	
IPI00018854	Coiled-coil domain-containing protein 68	CCDC68	Q9H2F9			1
IPI00084582	Isoform 7 of Collagen alpha-1(XIII) chain	COL13A1	Q5TAT6			1
IPI00216815	Isoform 2 of DNA topoisomerase 3-beta-1	TOP3B	O95985			1
IPI00873544	Uncharacterized protein ENSP00000380627 (Fragment)	-			6	4
IPI00062599	71 kDa protein	-				1
IPI00414122	coiled-coil domain containing 141	CCDC141			1	
IPI00060549	G-protein coupled receptor-associated sorting protein 1	GPRASP1	Q5JY77			1
IPI00795943	7 kDa protein	-				1
IPI00884216	RNA-directed DNA polymerase (Reverse transcriptase), related domain containing protein	-			1	
IPI00746034	NAB2 protein	NAB2			1	2
IPI00642204	Thioredoxin domain-containing protein 16 precursor	TXNDC16	Q9P2K2			1
IPI00397759	similar to ribosomal protein L21	LOC388621			1	
IPI00646199	Zinc finger, MYM-type 2	ZMYM2				1
IPI00292086	HLCDGP1	CSNK1A1			1	1
IPI00013895	Protein S100-A11	S100A11	P31949		1	3
IPI00791447	Pseudogene candidate	LOC339929				1
IPI00156871	Isoform 2 of Ubiquitin carboxyl-terminal hydrolase 49	USP49	Q70CQ1		1	
IPI00014230	Complement component 1 Q subcomponent-binding protein, mitochondrial precursor	C1QBP	Q07021		1	6
IPI00745433	eukaryotic translation initiation factor 2C, 2	EIF2C2	Q9UKV8			1
IPI00168692	Isoform 2 of Zinc finger protein 318	ZNF318	Q5VUA4		1	
IPI00187082	Isoform 3 of Multiple C2 and transmembrane domain-containing protein 1	MCTP1	Q6DN14		1	
IPI00419675	hypothetical protein LOC150763	LOC150763			2	
IPI00103090	Isoform 1 of RNA polymerase-associated protein LEO1	LEO1	Q8WVC0			1
IPI00746600	44 kDa protein	B4GALT3			2	1
IPI00018805	Uncharacterized protein C14orf105	C14orf105	Q9NVL8		1	
IPI00101186	Isoform 1 of RRP12-like protein	RRP12	Q5JTH9			1
IPI00013404	Mothers against decapentaplegic homolog 4	SMAD4	Q13485			1

Table S1.

Number of unique peptides identified

IPI	Protein name	Gene symbol	Swiss Prot ID	Normal	CFS	nPTLS
IPI00446685	CDNA FLJ41345 fis, clone BRAWH2002761	-			1	
IPI00220038	Isoform B of Arsenite-resistance protein 2	ARS2	Q9BXP5			1
IPI00470515	Isoform 1 of Uncharacterized protein C1orf173	C1orf173	Q5RHP9		1	
IPI00385683	Ig heavy chain V-III region GAR	-	P80419			1
IPI00001317	Rho-related BTB domain-containing protein 1	RHOBTB1	O94844		1	
IPI00024523	Isoform A of DnaJ homolog subfamily B member 6	DNAJB6	O75190		1	
IPI00030814	Fibroblast growth factor 22 precursor	FGF22	Q9HCT0		1	
IPI00441064	Isoform 1 of Uncharacterized protein C15orf26	C15orf26	Q6P656		1	
IPI00867744	Isoform 3 of Pleckstrin homology domain-containing family H member 2	PLEKHH2	Q8IVE3		1	
IPI00006030	Dynein intermediate chain 1, axonemal	DNAI1	Q9UI46			1
IPI00784027	Rod cGMP-specific 3',5'-cyclic phosphodiesterase subunit beta precursor	PDE6B	P35913		1	
IPI00024714	Isoform 1 of Regulator of G-protein signaling 12	RGS12	O14924			1
IPI00879277	436 kDa protein	TRRAP			1	
IPI00011631	Centromere/kinetochore protein zw10 homolog	ZW10	O43264		1	
IPI00009851	PCDH12 protein	PCDH12	Q9NPG4		2	1
IPI00014186	Isoform A of Zinc finger homeobox protein 3	ZFHX3	Q15911		1	
IPI00852699	4 kDa protein	-			1	
IPI00022621	Microfibrillar-associated protein 2 precursor	MFAP2	P55001		1	1
IPI00400834	Mediator of RNA polymerase II transcription subunit 13-like	MED13L	Q71F56		1	
IPI00018203	Isoform SRP55-2 of Splicing factor, arginine/serine-rich 6	SFRS6	Q13247		1	
IPI00003469	Ig kappa chain V-I region WEA	-	P01610		2	1
IPI00259671	Taste receptor type 2 member 44	TAS2R44	P59538			1
IPI00007648	Sodium- and chloride-dependent GABA transporter 3	SLC6A11	P48066		1	1
IPI00551062	Isoform 1 of Protein canopy homolog 3 precursor	CNPY3	Q9BT09			3
IPI00166323	LOC152485 protein	LOC152485			1	1
IPI00335581	Isoform 1 of E3 ubiquitin-protein ligase UBR3	UBR3	Q6ZT12			1

Table S1.

IPI	Protein name	Gene symbol	Swiss Prot ID	Number of unique peptides identified<br>Normal	CFS	nPTLS
IPI00386418	Isoform 2 of Myelin expression factor 2	MYEF2	Q9P2K5		1	2
IPI00335259	deleted in liver cancer 1 isoform 1	DLC1			1	
IPI00004416	Charged multivesicular body protein 2a	CHMP2A	O43633			1
IPI00239405	Isoform 1 of Nesprin-2	SYNE2	Q8WXH0		1	
IPI00788010	similar to 60S ribosomal protein L21	RPL21			1	
IPI00296286	Isoform 1 of Tomoregulin-2 precursor	TMEFF2	Q9UIK5		1	2
IPI00470606	Isoform 1 of tRNA guanosine-2'-O-methyltransferase TRM11 homolog	TRMT11	Q7Z4G4		1	1
IPI00028828	Isoform Beta of Zinc finger protein 202	ZNF202	O95125			1
IPI00027487	Creatine kinase M-type	CKM	P06732		1	
IPI00167419	Isoform 2 of Ankyrin repeat domain-containing protein 44	ANKRD44	Q8N8A2		1	1
IPI00029662	Isoform 1 of Potassium voltage-gated channel subfamily H member 2	KCNH2	Q12809		1	
IPI00292130	Dermatopontin precursor	DPT	Q07507		1	2
IPI00651669	Putative uncharacterized protein	-			1	
IPI00029446	Isoform 1 of Myotubularin-related protein 5	SBF1	O95248		1	
IPI00883929	Conserved hypothetical protein	-			1	
IPI00514795	Isoform 1 of Tubulin--tyrosine ligase-like protein 7	TTLL7	Q6ZT98			1
IPI00101987	Uncharacterized protein C19orf62	C19orf62	Q9NWV8			1
IPI00853224	START domain containing 7	STARD7	Q9NQZ5			1
IPI00054042	Isoform 1 of General transcription factor II-I	GTF2I	P78347		1	1
IPI00641155	20 kDa protein	HIRIP3			1	
IPI00001141	Mitochondrial import inner membrane translocase subunit Tim22	TIMM22	Q9Y584			1
IPI00030307	Solute carrier family 41 member 2	SLC41A2	Q96JW4		1	
IPI00465070	Histone H3.1	HIST1H3E	P68431		1	1
IPI00398162	Isoform 2 of Nebulin-related-anchoring protein	NRAP	Q86VF7		1	
IPI00158506	Isoform 3 of Zinc finger protein 688	ZNF688	Q8WV14		1	
IPI00514975	Chromosome 1 open reading frame 133	C1orf133				1
IPI00003768	Isoform 1 of Pescadillo homolog 1	PES1	O00541		1	
IPI00744029	Similar to Zinc finger, FYVE domain containing 21	-				1
IPI00399320	Transmembrane protein 59 precursor	TMEM59	Q9BXS4			1
IPI00815662	Rheumatoid factor G9 heavy chain (Fragment)	-			2	3
IPI00100910	Isoform 2 of Armadillo repeat-containing protein 5	ARMC5	Q96C12		1	
IPI00445894	CDNA FLJ42978 fis, clone BRTHA2004821	-			1	

Table S1.

Number of unique peptides identified

IPI	Protein name	Gene symbol	Swiss Prot ID	Normal	CFS	nPTLS
IPI00022325	Isoform 4 of Pro-neuregulin-2, membrane-bound isoform precursor	NRG2	O14511		1	
IPI00217687	Isoform 1 of Coiled-coil domain-containing protein 135	CCDC135	Q8IY82			1
IPI00829745	Cellular titin isoform PEVK variant 2 (Fragment)	-				1
IPI00216544	Isoform 2 of Protein hairless	HR	O43593			1
IPI00747271	zinc finger protein SBZF3	ZNF695			1	
IPI00646323	Isoform 1 of Uncharacterized protein C10orf76	C10orf76	Q5T2E6			1
IPI00006178	Y6 encoding protein	NPY6R			1	
IPI00386797	Isoform 1 of Folliculin-interacting protein 1	FNIP1	Q8TF40			1
IPI00304589	Isoform 1 of 182 kDa tankyrase 1-binding protein	TNKS1BP1	Q9C0C2			1
IPI00796745	22 kDa protein	PPP1R14D			1	
IPI00002857	Isoform CSBP2 of Mitogen-activated protein kinase 14	MAPK14	Q16539			1
IPI00299880	Isoform 2 of Calcium-activated potassium channel subunit beta-3	KCNMB3	Q9NPA1		1	
IPI00027356	Adenylate cyclase type 8	ADCY8	P40145		1	1
IPI00413293	Isoform 1 of Torsin-1A precursor	TOR1A	O14656		1	1
IPI00328885	eEF1A2 binding protein	DKFZp434B1231			1	
IPI00009305	Glucosamine-6-phosphate isomerase	GNPDA1	P46926		1	
IPI00465054	Putative uncharacterized protein DKFZp686C1054	THUMPD1			1	
IPI00005171	HLA class II histocompatibility antigen, DR alpha chain precursor	HLA-DRA	P01903		1	1
IPI00017630	Nuclear fragile X mental retardation-interacting protein 1	NUFIP1	Q9UHK0			1
IPI00019992	Myb-related protein A	MYBL1	P10243			1
IPI00168848	UDP-GlcNAc:betaGal beta-1,3-N-acetylglucosaminyltransferase 7	B3GNT7	Q8NFL0			1
IPI00456676	Isoform 1 of F-box only protein 38	FBXO38	Q6PIJ6		1	
IPI00031101	Natriuretic peptides B precursor	NPPB	P16860		2	1
IPI00328089	Kappa B and V(D)J recombination signal sequences binding protein	HIVEP3			1	
IPI00641920	N-terminal EF-hand calcium-binding protein 2	NECAB2	Q7Z6G3		1	1
IPI00419815	Growth/differentiation factor 6 precursor	GDF6	Q6KF10		1	1
IPI00002506	Dolichyl-phosphate beta-glucosyltransferase	ALG5	Q9Y673		1	
IPI00011832	Secreted phosphoprotein 24 precursor	SPP2	Q13103		2	1

Table S1.

				Number of unique peptides identified		
**IPI**	**Protein name**	**Gene symbol**	**Swiss Prot ID**	**Normal**	**CFS**	**nPTLS**
IPI00410325	Low-density lipoprotein receptor class A domain-containing protein 3 precursor	LDLRAD3	Q86YD5		1	
IPI00450358	Putative uncharacterized protein	TOP			1	
IPI00216917	Isoform 1 of Uncharacterized protein C22orf9	C22orf9	Q6ICG6			1
IPI00043499	Probable urocanate hydratase	UROC1	Q96N76			1
IPI00219953	cytidine monophosphate (UMP-CMP) kinase 1, cytosolic	CMPK1	P30085		1	1
IPI00401776	mucin 6, gastric	MUC6	Q6W4X9		1	1
IPI00004472	Isoform 1 of Serine/threonine-protein kinase WNK1	WNK1	Q9H4A3		1	
IPI00025862	Isoform 1 of C4b-binding protein beta chain precursor	C4BPB	P20851		1	1
IPI00025753	Desmoglein-1 precursor	DSG1	Q02413		1	1
IPI00056507	Isoform 1 of F-box/WD repeat-containing protein 5	FBXW5	Q969U6			1
IPI00017451	Splicing factor 3 subunit 1	SF3A1	Q15459			1
IPI00465273	UHRF1-binding protein 1	UHRF1BP1	Q6BDS2			1
IPI00428677	Putative olfactory receptor (Fragment)	-			1	
IPI00738999	similar to ubiquitin-conjugating enzyme E2 variant 1	LOC643227				1
IPI00031047	CDNA: FLJ23571 fis, clone LNG12303	IQCG	Q9H095			1
IPI00788014	similar to Opioid growth factor receptor	LOC729015			1	
IPI00442208	CDNA FLJ16344 fis, clone TESTI2032643, moderately similar to ADENYLATE CYCLASE, TYPE IV	ADCY4			1	
IPI00022256	AP-2 complex subunit mu-1	AP2M1	Q96CW1			1
IPI00019932	ubiquitin-conjugating enzyme E2D 2 isoform 2	UBE2D2			1	
IPI00869224	Pseudogene candidate	-			1	
IPI00000102	Progonadoliberin-1 precursor	GNRH1	P01148		1	
IPI00151141	Isoform 1 of Serine/threonine-protein kinase WNK4	WNK4	Q96J92			1
IPI00470896	Isoform 2 of Zinc finger FYVE domain-containing protein 26	ZFYVE26	Q68DK2		1	
IPI00027232	Insulin-like growth factor 1 receptor precursor	IGF1R	P08069			1
IPI00329488	Isoform IB of Tyrosine-protein kinase ABL2	ABL2	P42684		1	
IPI00219604	Dual specificity mitogen-activated protein kinase kinase 1	MAP2K1	Q02750		1	
IPI00879915	15 kDa protein	C6			3	3
IPI00328748	Protein ARMET precursor	ARMET	P55145			1

Table S1.

Number of unique peptides identified

IPI	Protein name	Gene symbol	Swiss Prot ID	Normal	CFS	nPTLS
IPI00000030	Isoform Delta-1 of Serine/threonine-protein phosphatase 2A 56 kDa regulatory subunit delta isoform	PPP2R5D	Q14738		1	
IPI00017538	Isoform 2 of Separin	ESPL1	Q14674		1	
IPI00012759	Complexin-2	CPLX2	Q6PUV4		1	1
IPI00741524	Thrombospondin type-1 domain-containing protein 7A precursor	THSD7A	Q9UPZ6		1	1
IPI00787099	similar to SR protein related family member	LOC728676			1	
IPI00553006	Probable prolyl-tRNA synthetase, mitochondrial precursor	PARS2	Q7L3T8			1
IPI00297235	cDNA FLJ77204	CCPG1	Q9ULG6		1	
IPI00102803	Ankyrin repeat domain-containing protein 22	ANKRD22	Q5VYY1			1
IPI00013743	Isoform 1 of BUD13 homolog	BUD13	Q9BRD0		1	1
IPI00002405	2'-5'-oligoadenylate synthetase 3	OAS3	Q9Y6K5		1	
IPI00465173	Isoform 1 of Multidrug and toxin extrusion protein 1	SLC47A1	Q96FL8		1	1
IPI00060866	Isoform 1 of AP-1 complex subunit sigma-3	AP1S3	Q96PC3		1	1
IPI00816289	V2-1 protein	IGLV3-1			2	2
IPI00296434	Isoform 1 of Slit homolog 1 protein precursor	SLIT1	O75093		1	1
IPI00153053	Calreticulin-3 precursor	CALR3	Q96L12		2	1
IPI00013205	Isoform 1 of JmjC domain-containing histone demethylation protein 3B	JMJD2B	O94953		1	
IPI00218715	Isoform 2 of ADAM 33 precursor	ADAM33	Q9BZ11		1	
IPI00030153	Tudor domain-containing protein 6	TDRD6	O60522			1
IPI00010212	U6 snRNA-specific terminal uridylyltransferase 1	TUT1	Q9H6E5			1
IPI00844501	MG44 protein	SUV39H1				1
IPI00000949	Mu-crystallin homolog	CRYM	Q14894		2	3
IPI00456060	Protein	-				1
IPI00000621	HSPC103 (Fragment)	-			1	1
IPI00410666	Isoform 3 of Protein LAP4	SCRIB	Q14160		1	
IPI00016480	Isoform 1 of NACHT, LRR and PYD domains-containing protein 2	NLRP2	Q9NX02			1
IPI00166043	Isoform 2 of Progestin and adipoQ receptor family member 6	PAQR6	Q6TCH4			2
IPI00827815	Light chain Fab (Fragment)	-			1	
IPI00411545	Isoform 3 of Thrombopoietin precursor	THPO	P40225			1
IPI00013651	Isoform 3 of RNA-binding protein 33	RBM33	Q96EV2		1	
IPI00002344	steroid 5 alpha-reductase 3	SRD5A3			1	

Table S1.

IPI	Protein name	Gene symbol	Swiss Prot ID	Number of unique peptides identified<br>Normal	CFS	nPTLS
IPI00444408	CDNA FLJ45600 fis, clone BRTHA3020369	-			1	
IPI00032680	Apolipoprotein-L5	APOL5	Q9BWW9		1	
IPI00168239	Zinc finger protein GLIS1	GLIS1	Q8NBF1			1
IPI00005013	poly(rC) binding protein 4 isoform b	PCBP4			1	
IPI00165990	Isoform 4 of Transcription factor RFX4	RFX4	Q33E94			1
IPI00749489	fibrocystin L	PKHD1L1				1
IPI00294728	DmX-like protein 1	DMXL1	Q9Y485		1	
IPI00018953	Dipeptidyl peptidase 4	DPP4	P27487			2
IPI00004288	Isoform 1 of Sialic acid-binding Ig-like lectin 7 precursor	SIGLEC7	Q9Y286		1	
IPI00855960	Integral membrane protein GPR137C	GPR137C	Q8N3F9		1	
IPI00017603	Coagulation factor VIII precursor	F8	P00451		1	1
IPI00059144	Protein FAM46B	FAM46B	Q96A09			1
IPI00003571	Isoform S-CaBP1 of Calcium-binding protein 1	CABP1	Q9NZU7		1	
IPI00021751	Neurofilament heavy polypeptide	NEFH	P12036		1	
IPI00787697	similar to Oligophrenin 1 isoform 4	FLJ32810				1
IPI00410214	Isoform 1 of 3'(2'),5'-bisphosphate nucleotidase 1	BPNT1	O95861		1	1
IPI00043237	CDNA FLJ32095 fis, clone OCBBF2000998	LOC730193				1
IPI00103604	Voltage-dependent calcium channel gamma-8 subunit	CACNG8	Q8WXS5		1	1
IPI00028641	Isoform 4 of Protein phosphatase 1 regulatory subunit 12B	PPP1R12B	O60237			1
IPI00554711	Junction plakoglobin	JUP	P14923			2
IPI00879159	9 kDa protein	-				1
IPI00174347	Inactive dual specificity phosphatase 27	DUSP27	Q5VZP5		1	
IPI00787774	similar to collagen, type I, alpha 1	LOC730756			1	
IPI00186114	Isoform 2 of ADAMTS-16 precursor	ADAMTS16	Q8TE57		1	1
IPI00300046	Similar to Pregnancy-specific beta 1 glycoprotein	PSG4			1	1
IPI00829750	Uncharacterized protein ENSP00000375006	-			1	1
IPI00007800	Angiopoietin-related protein 2 precursor	ANGPTL2	Q9UKU9			3
IPI00018099	E3 ubiquitin-protein ligase FANCL	FANCL	Q9NW38			1
IPI00472782	PHD finger protein 14 isoform 1	PHF14			1	1
IPI00455076	Double C2-like domain-containing protein alpha	DOC2A	Q14183			1
IPI00304533	Membrane frizzled-related protein	MFRP	Q9BY79		3	1
IPI00419903	Putative L-aspartate dehydrogenase	LOC554235	A6ND91		1	
IPI00165357	Isoform 1 of Metastasis-associated protein MTA3	MTA3	Q9BTC8		1	

Table S1.

Number of unique peptides identified

IPI	Protein name	Gene symbol	Swiss Prot ID	Normal	CFS	nPTLS
IPI00024915	Isoform Mitochondrial of Peroxiredoxin-5, mitochondrial precursor	PRDX5	P30044		1	1
IPI00295940	Protein unc-84 homolog B	UNC84B	Q9UH99			1
IPI00299406	Pituitary adenylate cyclase-activating polypeptide type I receptor precursor	ADCYAP1R1	P41586		1	
IPI00401789	Isoform 2 of Highly divergent homeobox	HDX	Q7Z353		1	
IPI00002970	Beta-sarcoglycan	SGCB	Q16585			1
IPI00303732	Uncharacterized protein C20orf106 precursor	C20orf106	Q5JX71		1	
IPI00827789	HRV Fab N6-VL (Fragment)	-			1	3
IPI00016425	Isoform 2 of Synaptopodin 2-like protein	SYNPO2L	Q9H987		1	
IPI00878142	68 kDa protein	-				1
IPI00554488	cAMP-dependent protein kinase type I-beta regulatory subunit	PRKAR1B	P31321			1
IPI00030279	Isoform 1 of Zinc finger Ran-binding domain-containing protein 3	ZRANB3	Q5FWF4			1
IPI00074656	Isoform 1 of Coiled-coil domain-containing protein C6orf65	C6orf65	Q5SZJ8			1
IPI00017640	Isoform 1 of Slit homolog 3 protein precursor	SLIT3	O75094		1	
IPI00014577	Ras-related protein Rab-18	RAB18	Q9NP72		1	
IPI00033541	Lactosylceramide 4-alpha-galactosyltransferase	A4GALT	Q9NPC4		1	2
IPI00023856	Peregrin	BRPF1	P55201		1	
IPI00020230	Frizzled-9 precursor	FZD9	O00144			2
IPI00294241	Transcription factor IIIA	GTF3A	Q92664		1	
IPI00014903	Isoform 1 of FGFR1 oncogene partner 2	FGFR1OP2	Q9NVK5			1
IPI00025717	Metaxin-2	MTX2	O75431		1	
IPI00011961	Isoform 1 of Sialoadhesin precursor	SIGLEC1	Q9BZZ2			1
IPI00023095	Myeloid leukemia factor 2	MLF2	Q15773		1	
IPI00884399	RNA-directed DNA polymerase (Reverse transcriptase), related domain containing protein	-				1
IPI00382426	Ig lambda chain V-II region TRO	-	P01707		3	2
IPI00009645	Ran-binding protein 17	RANBP17	Q9H2T7		1	
IPI00170770	Isoform 1 of PHD finger protein 3	PHF3	Q92576			1
IPI00000057	Conserved oligomeric Golgi complex component 2	COG2	Q14746			1
IPI00022629	Isoform 1 of Protein regulator of cytokinesis 1	PRC1	O43663		1	
IPI00435928	PP13187	RASGRF1				1
IPI00031082	Putative uncharacterized protein DKFZp761O17121	LDOC1L			1	

Table S1.

IPI	Protein name	Gene symbol	Swiss Prot ID	Number of unique peptides identified: Normal	CFS	nPTLS
IPI00013721	Serine/threonine-protein kinase PRP4 homolog	PRPF4B	Q13523			1
IPI00100867	Transcription initiation factor TFIID 210 kDa subunit	TAF1L	Q8IZX4		1	1
IPI00411979	Isoform 3 of Formin-like protein 3	FMNL3	Q8IVF7		1	1
IPI00788120	similar to CG14182-PA	LOC730094			1	
IPI00748607	Conserved hypothetical protein	-				1
IPI00021266	60S ribosomal protein L23a	RPL23A	P62750			1
IPI00396063	C-myc promoter-binding protein	DENND4A	Q7Z401		1	
IPI00031228	Isoform 2 of Pleckstrin homology domain-containing family B member 2	PLEKHB2	Q96CS7			1
IPI00013296	40S ribosomal protein S18	RPS18	P62269		1	
IPI00004362	MORC family CW-type zinc finger protein 1	MORC1	Q86VD1		1	
IPI00337415	Guanine nucleotide-binding protein G(i), alpha-1 subunit	GNAI1	P63096			1
IPI00787625	similar to 60S ribosomal protein L23a	LOC389101			1	
IPI00106491	mRNA turnover protein 4 homolog	MRTO4	Q9UKD2		1	1
IPI00385020	Putative uncharacterized protein DKFZp686D159 (Fragment)	CAPRIN2			1	
IPI00003377	Isoform 1 of Splicing factor, arginine/serine-rich 7	SFRS7	Q16629			1
IPI00374223	hypothetical protein LOC389119	C3orf54	Q96EL1		1	
IPI00017551	Isoform 1 of Regucalcin	RGN	Q15493			3
IPI00745396	Stromal RNA regulating factor	HNRPLL				1
IPI00039626	Isoform D of UPF0318 protein FAM120A	FAM120A	Q9NZB2		1	
IPI00604752	Isoform 3 of Sterile alpha motif domain-containing protein 13	SAMD13	Q5VXD3			1
IPI00295253	Isoform 1 of Cell surface glycoprotein OX2 receptor precursor	CD200R1	Q8TD46		1	
IPI00299040	Polycystin-2	PKD2	Q13563		1	
IPI00382534	Ig heavy chain V-II region OU	-	P01814			1
IPI00008444	Isoform 1 of Cytidine and dCMP deaminase domain-containing protein 1	CDADC1	Q9BWV3			1
IPI00307165	Tripartite motif-containing protein 47	TRIM47	Q96LD4		1	
IPI00152685	Tetratricopeptide repeat protein 15	TTC15	Q8WVT3		1	
IPI00183526	NCL protein	NCL			2	
IPI00792933	zinc finger protein 462	ZNF462	Q96JM2			1
IPI00013789	SET and MYND domain-containing protein 5	SMYD5	Q6GMV2		1	
IPI00402005	Similar to DNA-binding protein	ZNF528				1
IPI00020036	Neuronal acetylcholine receptor subunit alpha-4 precursor	CHRNA4	P43681			1
IPI00396025	hypothetical protein LOC254122	FLJ30934			1	

Table S1.

Number of unique peptides identified

IPI	Protein name	Gene symbol	Swiss Prot ID	Normal	CFS	nPTLS
IPI00014232	ADP-ribosylation factor-like protein 6-interacting protein 1	ARL6IP1	Q15041		1	
IPI00329054	Osteopetrosis-associated transmembrane protein 1 precursor	OSTM1	Q86WC4			1
IPI00028864	Neurexophilin-3 precursor	NXPH3	O95157			1
IPI00021715	Isoform 1 of Collagen alpha-5(IV) chain precursor	COL4A5	P29400		1	
IPI00023757	Isoform 1 of X-linked retinitis pigmentosa GTPase regulator	RPGR	Q92834			1
IPI00026305	Hook homolog 1	HOOK1	Q9UJC3		1	
IPI00184160	Isoform 2 of RING finger protein 180	RNF180	Q86T96		1	
IPI00000663	Isoform Mitochondrial of Malonyl-CoA decarboxylase, mitochondrial precursor	MLYCD	O95822			1
IPI00216308	Voltage-dependent anion-selective channel protein 1	VDAC1	P21796		1	
IPI00739387	hypothetical protein LOC401478	FLJ45872				1
IPI00065533	Protein FAM20A precursor	FAM20A	Q96MK3		2	2
IPI00298214	Isoform 1 of ATP-binding cassette sub-family G member 2	ABCG2	Q9UNQ0		1	
IPI00164352	zinc finger protein 292	ZNF292	O60281			1
IPI00008913	Alpha-N-acetylgalactosaminide alpha-2,6-sialyltransferase 5	ST6GALNAC5	Q9BVH7			1
IPI00020454	Deoxycytidine kinase	DCK	P27707			1
IPI00514028	21 kDa protein	RNF207				1
IPI00024292	Low-density lipoprotein receptor-related protein 2 precursor	LRP2	P98164			1
IPI00028357	Exportin-4	XPO4	Q9C0E2			1
IPI00438355	Fibrillin-3 precursor	FBN3	Q75N90			1
IPI00009043	Apoptotic-related protein PCAR (Fragment)	-				1
IPI00651633	CASKIN1 protein (Fragment)	CASKIN1			1	
IPI00409659	Ubiquilin-2	UBQLN2	Q9UHD9		2	1
IPI00220113	Isoform 2 of Microtubule-associated protein 4	MAP4	P27816		1	1
IPI00166044	Isoform 1 of Regulatory-associated protein of mTOR	KIAA1303	Q8N122		1	1
IPI00010859	cortistatin preproprotein	CORT	O00230			1
IPI00220412	S100 calcium binding protein A1	S100A1				1
IPI00247063	Neprilysin	MME	P08473		1	
IPI00027442	Alanyl-tRNA synthetase, cytoplasmic	AARS	P49588			1
IPI00784257	Folate receptor beta precursor	FOLR2	P14207		7	9
IPI00014845	Isoform 1 of Ciliary dynein heavy chain 8	DNAH8	Q96JB1			1
IPI00398779	Isoform 4 of Plectin-1	PLEC1	Q15149		1	

Table S1.

Number of unique peptides identified

IPI	Protein name	Gene symbol	Swiss Prot ID	Normal	CFS	nPTLS
IPI00011062	Isoform 1 of Carbamoyl-phosphate synthase [ammonia], mitochondrial precursor	CPS1	P31327		1	6
IPI00168418	Putative uncharacterized protein C11orf64	C11orf64	Q8NCQ3		1	
IPI00167764	Transmembrane protein 102	TMEM102	Q8N9M5		1	
IPI00171716	OTTHUMP00000028696	TTC28	Q96AY4		1	
IPI00394818	Isoform 1 of Ankyrin repeat domain-containing protein 47	KANK3	Q6NY19		1	
IPI00021131	Leucine-rich repeat-containing G-protein coupled receptor 5 precursor	LGR5	O75473		1	1
IPI00883997	Coiled-coil domain-containing protein 19	CCDC19	Q9UL16		1	
IPI00385687	similar to Ubiquinol-cytochrome c reductase complex 14 kDa protein	-				1
IPI00830137	Uncharacterized protein ENSP00000374864	-			1	
IPI00007461	Isoform 1 of Protein dpy-19 homolog 1	DPY19L1	Q2PZI1		1	1
IPI00030874	Uncharacterized gastric protein YA42P (Fragment)	-			1	
IPI00455675	Isoform 2 of Centrosomal protein of 192 kDa	CEP192	Q8TEP8		1	
IPI00789889	Hypothetical protein	-			1	
IPI00829625	Uncharacterized protein FAM91A1	FAM91A1			1	
IPI00377214	Isoform 2 of NLR family member X1	NLRX1	Q86UT6			2
IPI00054521	Frizzled-1 precursor	FZD1	Q9UP38			2
IPI00010676	Isoform 1 of Urokinase plasminogen activator surface receptor precursor	PLAUR	Q03405		1	1
IPI00000873	Valyl-tRNA synthetase	VARS	P26640		1	
IPI00794829	16 kDa protein	ACADS				1
IPI00216423	Isoform 4 of Intersectin-2	ITSN2	Q9NZM3			1
IPI00303870	Seven transmembrane helix receptor	-				1
IPI00386185	FLJ00370 protein (Fragment)	-			1	
IPI00829697	Uncharacterized protein ENSP00000375030	-			1	
IPI00848229	EGF, latrophilin and seven transmembrane domain-containing protein 1 precursor	ELTD1	Q9HBW9			1
IPI00000690	Isoform 1 of Apoptosis-inducing factor 1, mitochondrial precursor	AIFM1	O95831		1	
IPI00016988	WD repeat-containing protein 13	WDR13	Q9H1Z4		1	
IPI00478058	Isoform 2 of NMDA receptor-regulated 1-like protein	NARG1L	Q6N069		1	
IPI00293857	Isoform 1A of Beta-arrestin-1	ARRB1	P49407			1
IPI00293565	fms-related tyrosine kinase 4 isoform 1	FLT4				1

Table S1.

Number of unique peptides identified

IPI	Protein name	Gene symbol	Swiss Prot ID	Normal	CFS	nPTLS
IPI00022471	minor histocompatibility antigen HA-1	HMHA1				1
IPI00848252	hypothetical protein LOC285501	LOC285501				1
IPI00854589	Uncharacterized protein ENSP00000375004	-			3	3
IPI00782960	Isoform 1 of Leucine-rich repeat and IQ domain-containing protein 3	LRRC44	A6PVS8		1	
IPI00288939	Isoform 3 of Coiled-coil domain-containing protein 40	CCDC40	Q4G0X9			1
IPI00442909	CDNA FLJ26301 fis, clone DMC07540	IGHV4-31			3	3
IPI00103373	Cytoglobin	CYGB	Q8WWM9		1	
IPI00646831	34 kDa protein	-			1	
IPI00065057	Putative uncharacterized protein CXorf58	CXorf58	Q96LI9			1
IPI00073769	48 kDa protein	PALM2				1
IPI00065520	Isoform 1 of Protein phosphatase 1M	PPM1M	Q96MI6			1
IPI00436518	Benzodiazepine receptor ligand	-				1
IPI00001580	Isoform 1 of FYVE and coiled-coil domain-containing protein 1	FYCO1	Q9BQS8		1	1
IPI00384164	Full-length cDNA clone CS0DI075YC18 of Placenta of Homo sapiens (Fragment)	PGF			1	
IPI00739611	similar to golgi autoantigen, golgin subfamily a, 8A isoform 3	LOC727909			1	
IPI00012451	Guanine nucleotide-binding protein subunit beta-4	GNB4	Q9HAV0		1	
IPI00394960	Isoform 1 of Probable G-protein coupled receptor 133 precursor	GPR133	Q6QNK2		1	
IPI00555595	Ubiquitin carboxyl-terminal esterase L1 (Ubiquitin thiolesterase) variant (Fragment)	-			1	
IPI00431697	Cancer/testis antigen 45-5	RP13-36C9.6	Q6NSH3		1	
IPI00015345	Cadherin EGF LAG seven-pass G-type receptor 3 precursor	CELSR3	Q9NYQ7			1
IPI00847391	Isoform 1 of Zinc finger protein 276	ZNF276	Q8N554			1
IPI00001654	Isoform 3 of Pericentriolar material 1 protein	PCM1	Q15154			1
IPI00179452	Isoform 1 of Protein CBFA2T2	CBFA2T2	O43439		2	
IPI00028066	class IV alcohol dehydrogenase 7 mu or sigma subunit	ADH7	P40394		1	
IPI00152960	RNA polymerase I-specific transcription initiation factor RRN3	RRN3	Q9NYV6		1	
IPI00004970	Small subunit processome component 20 homolog	UTP20	O75691		2	1
IPI00792370	6 kDa protein	GNAT1			1	

Table S1.

Number of unique peptides identified

IPI	Protein name	Gene symbol	Swiss Prot ID	Normal	CFS	nPTLS
IPI00167762	Isoform 1 of Uncharacterized protein C19orf47	C19orf47	Q8N9M1		1	
IPI00385321	Isoform 2 of Protein kinase C-binding protein 1	ZMYND8	Q9ULU4		1	
IPI00028493	Isoform 1 of Tuberin	TSC2	P49815			1
IPI00385153	HUMEEP	ATXN10			1	
IPI00332583	chorionic somatomammotropin hormone 1 isoform 2	CSH1				1
IPI00337348	Growth differentiation factor 7	GDF7	Q7Z4P5		1	
IPI00301844	Zinc fingers and homeoboxes protein 3	ZHX3	Q9H4I2			1
IPI00013788	HIV Tat-specific factor 1	HTATSF1	O43719			1
IPI00001151	Isoform 2 of Uncharacterized MFS-type transporter C20orf59	C20orf59	Q9BYT1			1
IPI00013079	EMILIN-1 precursor	EMILIN1	Q9Y6C2		1	3
IPI00060627	Coiled-coil domain-containing protein 124	CCDC124	Q96CT7		1	
IPI00401190	LOC155006 protein	LOC155006			1	
IPI00025039	rRNA 2'-O-methyltransferase fibrillarin	FBL	P22087		1	
IPI00479578	similar to Nonhistone chromosomal protein HMG-14	hCG_1659830			1	
IPI00018245	Isoform 3 of DNA-directed RNA polymerase III subunit RPC5	POLR3E	Q9NVU0		1	
IPI00020565	PRO1598	-			1	
IPI00446711	CDNA FLJ41261 fis, clone BRAMY2034920	-			1	
IPI00787990	similar to ribosomal protein S14	LOC644119			1	
IPI00011951	Isoform 2 of Uncharacterized protein KIAA0427	KIAA0427	O43310		1	1
IPI00218743	Isoform 2 of Bestrophin-1	BEST1	O76090		1	
IPI00847237	Conserved hypothetical protein	-				1
IPI00736826	similar to Temporarily Assigned Gene name family member	LOC402217			1	
IPI00295999	Isoform 2 of Collagen alpha-1(IX) chain precursor	COL9A1	P20849			1
IPI00296196	Dimethylglycine dehydrogenase, mitochondrial precursor	DMGDH	Q9UI17			1
IPI00412024	Zinc finger protein 485	ZNF485	Q8NCK3		1	
IPI00396145	FYVE finger-containing phosphoinositide kinase	PIP5K3	Q9Y2I7			1
IPI00478110	Probable G-protein coupled receptor 135	GPR135	Q8IZ08			1
IPI00297559	Isoform 1 of USH1C-binding protein 1	USHBP1	Q8N6Y0		1	
IPI00168995	Olfactory receptor 4D9	OR4D9	Q8NGE8			1
IPI00396147	Protocadherin-20 precursor	PCDH20	Q8N6Y1		1	

Table S1.

Number of unique peptides identified

IPI	Protein name	Gene symbol	Swiss Prot ID	Normal	CFS	nPTLS
IPI00478572	Collagen alpha-4(IV) chain precursor	COL4A4	P53420		1	
IPI00032785	Isoform II of Amyloid beta A4 precursor protein-binding family B member 3	APBB3	O95704		1	
IPI00031195	Isoform 1 of Vang-like protein 1	VANGL1	Q8TAA9			1
IPI00059190	Isoform 1 of SLAM family member 9 precursor	SLAMF9	Q96A28			1
IPI00043467	Tigger transposable element-derived protein 1	TIGD1	Q96MW7			1
IPI00009982	Isoform 1 of Tudor and KH domain-containing protein	TDRKH	Q9Y2W6		1	
IPI00216164	Peroxisomal bifunctional enzyme	EHHADH	Q08426		1	
IPI00419912	YY2 transcription factor	YY2			1	
IPI00235842	Zinc finger protein 483	ZNF483	Q8TF39			1
IPI00735531	proline-rich synapse-associated protein 2 isoform 4	SHANK3			1	
IPI00005104	Inhibitor of nuclear factor kappa-B kinase subunit alpha	CHUK	O15111		1	
IPI00008837	Tyrosine-protein phosphatase non-receptor type 5	PTPN5	P54829		1	
IPI00017800	ATP-binding cassette sub-family A member 3	ABCA3	Q99758		1	
IPI00220473	Isoform 2 of Calcium-transporting ATPase type 2C member 1	ATP2C1	P98194			1
IPI00382831	Cerebral protein-13	BAP1				1
IPI00737972	Transcription factor 24 (Fragment)	TCF24			1	
IPI00374074	similar to CG40449-PA.3	LOC388795			1	
IPI00027765	retbindin isoform 2	RTBDN			3	1
IPI00477692	Uncharacterized protein ENSP00000330808	LOC131149				1
IPI00220533	Isoform D of Transmembrane protease, serine 3	TMPRSS3	P57727		1	
IPI00301583	Isoform 1 of Zinc finger protein 691	ZNF691	Q5VV52			1
IPI00790447	12 kDa protein	-				1
IPI00006543	Complement factor H-related 5	CFHR5			1	1
IPI00829904	Uncharacterized protein ENSP00000374814	-				1
IPI00302458	Exportin-7	XPO7	Q9UIA9		1	
IPI00646826	Putative uncharacterized protein FLJ32658	FLJ32658			1	
IPI00387161	Ig lambda chain V-I region VOR	-	P01699			2
IPI00016859	Isoform 1 of Transcription factor IIIB 90 kDa subunit	BRF1	Q92994		1	
IPI00020533	Protein BEX1	BEX1	Q9HBH7			1

Table S1.

Number of unique peptides identified

IPI	Protein name	Gene symbol	Swiss Prot ID	Normal	CFS	nPTLS
IPI00003893	Isoform 1 of Protocadherin gamma B3 precursor	PCDHGB3	Q9Y5G1		2	
IPI00376436	Isoform 4 of Vacuolar protein sorting-associated protein 13B	VPS13B	Q7Z7G8			1
IPI00029162	Isoform 2 of Cell division cycle 2-like protein kinase 5	CDC2L5	Q14004			1
IPI00031386	Phosphatidylinositol-4,5-bisphosphate 3-kinase catalytic subunit alpha isoform	PIK3CA	P42336		1	
IPI00472426	Zinc finger protein 780B	ZNF780B	Q9Y6R6		1	1
IPI00065547	OTTHUMP00000031495	RP11-93B14.6			1	
IPI00043370	WD repeat-containing protein 52	WDR52	Q96MT7		1	
IPI00328872	hypothetical protein	LOC339742			1	1
IPI00065500	BRO1 domain-containing protein BROX	C1orf58	Q5VW32			1
IPI00740336	formin 1	FMN1	Q68DA7			1
IPI00017203	Protein RMI1 homolog	RMI1	Q9H9A7			1
IPI00064767	Isoform 1 of Rho GTPase-activating protein 17	ARHGAP17	Q68EM7		1	
IPI00012544	Isoform 2 of Protocadherin gamma A9 precursor	PCDHGA9	Q9Y5G4		2	2
IPI00293431	WUGSC:H_DJ0855D21.2 protein	MGC70863				1
IPI00219563	Isoform A of 1-phosphatidylinositol-4,5-bisphosphate phosphodiesterase beta-1	PLCB1	Q9NQ66			2
IPI00295400	Tryptophanyl-tRNA synthetase, cytoplasmic	WARS	P23381			1
IPI00219689	Isoform Beta of Protein vav-3	VAV3	Q9UKW4		1	
IPI00444330	CDNA FLJ45693 fis, clone FEBRA2012625	-				1
IPI00170509	Isoform 3 of Calcium/calmodulin-dependent protein kinase kinase 2	CAMKK2	Q96RR4			1
IPI00328291	Cytoskeleton-associated protein 2-like	CKAP2L			1	
IPI00382539	Ig heavy chain V-II region WAH	-	P01824		2	2
IPI00017533	Cytochrome c oxidase subunit 3	MT-CO3	P00414			1
IPI00647897	RING finger protein 113B	RNF113B	Q8IZP6		1	
IPI00419922	Isoform 1 of IQ domain-containing protein E	IQCE	Q6IPM2			1
IPI00024285	similar to testicular serine protease 2	LOC138652			1	
IPI00005710	Heparan sulfate glucosamine 3-O-sulfotransferase 2	HS3ST2	Q9Y278			1
IPI00783200	38 kDa protein	RAPGEF3			1	
IPI00744872	Isoform 1 of Uncharacterized protein KIAA0423	KIAA0423	Q9Y4F4			1
IPI00014877	Isoform 2 of Glutaminyl-peptide cyclotransferase-like protein	QPCTL	Q9NXS2		1	

Table S1.

Number of unique peptides identified

IPI	Protein name	Gene symbol	Swiss Prot ID	Normal	CFS	nPTLS
IPI00186554	Uncharacterized protein KIAA1853	KIAA1853	A7MD48		1	
IPI00418336	Isoform 2 of Integrator complex subunit 3	INTS3	Q68E01		1	
IPI00872730	Macrophin 1 isoform	MACF1			1	
IPI00796518	16 kDa protein	NGRN			1	1
IPI00787412	similar to Protein C11orf2	LOC441616				1
IPI00168607	tetratricopeptide repeat domain 21A isoform 1	TTC21A	Q8NDW8			1
IPI00303207	ATP-binding cassette sub-family E member 1	ABCE1	P61221		1	
IPI00419385	olfactory receptor, family 52, subfamily E, member 8	OR52E8	Q6IFG1			1
IPI00301609	Serine/threonine-protein kinase Nek9	NEK9	Q8TD19		1	
IPI00744527	Conserved hypothetical protein	-			1	
IPI00006236	Platelet-derived growth factor receptor-like protein precursor	PDGFRL	Q15198			1
IPI00291796	BTB (POZ) domain containing 12	BTBD12				1
IPI00400795	54 kDa protein	-				1
IPI00302351	Isoform 1 of Serine/threonine-protein kinase 33	STK33	Q9BYT3		1	
IPI00303622	Olfactory receptor OR9-23	OR1N2	Q8NGR9			2
IPI00878483	30 kDa protein	-			1	
IPI00031554	ATP-dependent RNA helicase DDX50	DDX50	Q9BQ39			1
IPI00023860	Nucleosome assembly protein 1-like 1	NAP1L1	P55209			1
IPI00376210	Probable G-protein coupled receptor 142	GPR142	Q7Z601		1	1
IPI00744179	Uncharacterized protein ENSP00000370856 (Fragment)	-			1	
IPI00396279	Isoform 1 of CLIP-associating protein 1	CLASP1	Q7Z460		1	
IPI00375225	Olfactory receptor 2AE1	OR2AE1	Q8NHA4			1
IPI00235622	Isoform 3 of CUB domain-containing protein 1 precursor	CDCP1	Q9H5V8			1
IPI00742031	similar to golgi autoantigen, golgin subfamily a, 8G	LOC645137				1
IPI00013378	Isoform Long of DNA topoisomerase 3-alpha	TOP3A	Q13472		1	
IPI00221372	Isoform 2 of 1-acyl-sn-glycerol-3-phosphate acyltransferase beta	AGPAT2	O15120			1
IPI00429190	Ras-related protein Rab-11A	RAB11A	P62491		1	
IPI00644752	hypothetical protein isoform 1	LOC643669			1	2
IPI00464994	Isoform 1 of tRNA modification GTPase GTPBP3, mitochondrial precursor	GTPBP3	Q969Y2		1	
IPI00008449	Isoform 3 of Pre-mRNA 3'-end-processing factor FIP1	FIP1L1	Q6UN15		1	
IPI00414402	Olfactory receptor 2T27	OR2T27	Q8NH04		1	

Table S1.

Number of unique peptides identified

IPI	Protein name	Gene symbol	Swiss Prot ID	Normal	CFS	nPTLS
IPI00796972	62 kDa protein	ETV5				1
IPI00010807	Frizzled-8 precursor	FZD8	Q9H461			1
IPI00103146	PDZ and LIM domain 5 isoform e	PDLIM5			1	1
IPI00175193	Chromosome-associated kinesin KIF4B	KIF4B	Q2VIQ3		1	1
IPI00031410	FKBP12-rapamycin complex-associated protein	FRAP1	P42345			1
IPI00019848	Isoform 1 of Host cell factor	HCFC1	P51610			1
IPI00168913	Isoform 2 of Limbin	EVC2	Q86UK5		1	
IPI00306611	TRAF-interacting protein	TRAIP	Q9BWF2			1
IPI00009315	Golgi resident protein GCP60	ACBD3	Q9H3P7		1	
IPI00410127	Transforming, acidic coiled-coil containing protein 2	TACC2			2	
IPI00871625	cDNA FLJ77072, highly similar to Homo sapiens prostaglandin E receptor 3 (subtype EP3) (PTGER3), transcript variant 9, mRNA	PTGER3			1	
IPI00153031	Isoform 1 of Uncharacterized protein C17orf80	C17orf80	Q9BSJ5			1
IPI00479296	Isoform 1 of ATP-binding cassette sub-family A member 8	ABCA8	O94911		1	
IPI00397622	Isoform 1 of Transmembrane and immunoglobulin domain-containing protein 1 precursor	TMIGD1	Q6UXZ0		1	
IPI00289097	Isoform 1 of Ras-GEF domain-containing family member 1B	RASGEF1B	Q0VAM2			1
IPI00300221	Putative uncharacterized protein DKFZp434I1916	ELL			1	
IPI00026036	Isoform 5 of Dipeptidyl peptidase 8	DPP8	Q6V1X1		1	
IPI00425404	Isoform 1 of Kinesin-like protein KIF21A	KIF21A	Q7Z4S6			1
IPI00783656	39S ribosomal protein L38, mitochondrial precursor	MRPL38	Q96DV4		1	1
IPI00477262	Uncharacterized protein TRIM41 (Fragment)	TRIM41				1
IPI00647205	Uncharacterized protein C6orf174 precursor	C6orf174	Q5TF21		1	
IPI00030703	Motor neuron and pancreas homeobox protein 1	MNX1	P50219			1
IPI00025974	Charged multivesicular body protein 4b	CHMP4B	Q9H444			1
IPI00030945	Urocortin-2 precursor	UCN2	Q96RP3		1	
IPI00413655	ankyrin repeat domain 55 isoform 1	ANKRD55	Q3KP44			1
IPI00006932	Isoform 1 of Putative RNA-binding protein Luc7-like 2	LUC7L2	Q9Y383		1	
IPI00029631	Enhancer of rudimentary homolog	ERH	P84090		1	1

Table S1.

Number of unique peptides identified

IPI	Protein name	Gene symbol	Swiss Prot ID	Normal	CFS	nPTLS
IPI00026497	Isoform 1 of Poly [ADP-ribose] polymerase 2	PARP2	Q9UGN5			1
IPI00387099	Ig kappa chain V-I region Rei	-	P01607		2	1
IPI00025155	Follistatin-related protein 3 precursor	FSTL3	O95633		2	1
IPI00017819	Bile acid-CoA:amino acid N-acyltransferase	BAAT	Q14032		1	
IPI00145121	GPI mannosyltransferase 3	PIGB	Q92521		1	
IPI00061206	Polyadenylate-binding protein 5	PABPC5	Q96DU9			1
IPI00289758	Calpain-2 catalytic subunit precursor	CAPN2	P17655			1
IPI00744357	Conserved hypothetical protein	-			1	
IPI00410320	Sodium-dependent glucose transporter	SLC5A11			1	
IPI00065085	CDNA FLJ25371 fis, clone TST01885	FLJ25371			1	
IPI00295601	Cell cycle exit and neuronal differentiation protein 1	CEND1	Q8N111		3	2
IPI00465177	Transforming growth factor-alpha variant I	TGFA				1
IPI00742682	nuclear pore complex-associated protein TPR	TPR	P12270		1	1
IPI00289914	MutS protein homolog 4	MSH4	O15457		1	
IPI00743360	Calcium-binding and coiled-coil domain-containing protein 2	CALCOCO2	Q13137		1	1
IPI00180305	Isoform 5 of E3 ubiquitin-protein ligase UBR4	UBR4	Q5T4S7			1
IPI00012438	Isoform Long of 52 kDa repressor of the inhibitor of the protein kinase	PRKRIR	O43422		1	
IPI00217895	rai-like protein	SHC4			1	
IPI00294618	Proline-rich protein PRCC	PRCC	Q92733		1	
IPI00333503	Uncharacterized protein ENSP00000315571 (Fragment)	-			1	
IPI00019244	Isoform 1 of RNA-binding protein Nova-1	NOVA1	P51513			1
IPI00376197	Complement C1q-like protein 2 precursor	C1QL2	Q7Z5L3		2	1
IPI00009101	Isoform 2 of Helicase SRCAP	SRCAP	Q6ZRS2			1
IPI00000784	Isoform MEF2DAB of Myocyte-specific enhancer factor 2D	MEF2D	Q14814		1	
IPI00782992	Isoform 1 of Serine/arginine repetitive matrix protein 2	SRRM2	Q9UQ35			1
IPI00848180	Conserved hypothetical protein	-			1	
IPI00006663	Aldehyde dehydrogenase, mitochondrial precursor	ALDH2	P05091			3
IPI00015135	Exostosin-like 3	EXTL3	O43909		2	1
IPI00045208	Zinc finger protein 300-B	ZNF300	Q96RE9		1	
IPI00807675	ZNF440 protein	ZNF440				1
IPI00216236	Troponin I, fast skeletal muscle	TNNI2	P48788		1	1

Table S1.

Number of unique peptides identified

IPI	Protein name	Gene symbol	Swiss Prot ID	Normal	CFS	nPTLS
IPI00871452	Isoform 1 of PWWP domain-containing protein 2A	PWWP2A	Q96N64		1	
IPI00102820	Isoform 3 of Mitochondrial tumor suppressor 1	MTUS1	Q9ULD2		1	
IPI00104050	Thyroid hormone receptor-associated protein 3	THRAP3	Q9Y2W1			1
IPI00007836	Short transient receptor potential channel 5	TRPC5	Q9UL62		1	1
IPI00215786	3',5'-cyclic nucleotide phosphodiesterase 10A2	PDE10A	Q9Y233		1	
IPI00411480	hypothetical protein	LOC729461				1
IPI00845401	Isoform 6 of Neuron navigator 1	NAV1	Q8NEY1		1	
IPI00217979	Myeloid/lymphoid or mixed-lineage leukemia 5	MLL5			1	
IPI00646423	Isoform 2 of Transcription initiation factor TFIID subunit 4B	TAF4B	Q92750			1
IPI00446312	CDNA FLJ42271 fis, clone TKIDN2015788	-				1
IPI00440764	Isoform 1 of Gamma-glutamyltransferase 6 precursor	GGT6	Q6P531		1	
IPI00179298	482 kDa protein	HUWE1				1
IPI00016006	Isoform 1 of Gephyrin	GPHN	Q9NQX3			1
IPI00395630	Putative uncharacterized protein pp6170	SCRIB				1
IPI00737363	similar to CG3173-PA isoform 7	INTS1			1	1
IPI00100067	Isoform 1 of Tumor necrosis factor receptor superfamily member 19 precursor	TNFRSF19	Q9NS68			1
IPI00220308	Isoform 2 of Merlin	NF2	P35240			2
IPI00789476	7 kDa protein	-				1
IPI00374068	Isoform 1 of ADAMTS-like protein 4 precursor	ADAMTSL4	Q6UY14		1	
IPI00306749	Kanadaptin	SLC4A1AP	Q9BWU0			1
IPI00001922	Suppressor of tumorigenicity protein 14	ST14	Q9Y5Y6			1
IPI00464980	Isoform 1 of Paired amphipathic helix protein Sin3b	SIN3B	O75182			1
IPI00186439	Zinc finger protein 643	ZNF643	Q9UJL9		1	
IPI00062467	Isoform 1 of Zinc finger protein 274	ZNF274	Q96GC6		1	
IPI00000890	Isoform 1 of Growth hormone variant precursor	GH2	P01242		1	
IPI00065351	Zinc finger protein 354B	ZNF354B	Q96LW1		1	
IPI00744932	Conserved hypothetical protein	ZDHHC1				1
IPI00552455	12 kDa protein	INPP5A			1	
IPI00395473	Zinc finger protein 135	ZNF135	P52742		1	

Table S1.

Number of unique peptides identified

IPI	Protein name	Gene symbol	Swiss Prot ID	Normal	CFS	nPTLS
IPI00003842	Isoform 1 of Microtubule-associated protein 2	MAP2	P11137		1	
IPI00655760	Envelope polyprotein	-			1	
IPI00022799	Isoform 2 of Aquaporin-4	AQP4	P55087			2
IPI00445750	CDNA FLJ43142 fis, clone CTONG3007870	-				1
IPI00384767	FLJ00332 protein (Fragment)	TBC1D10C			1	
IPI00006077	Protein Jade-3	PHF16	Q92613			1
IPI00061148	Sodium bicarbonate cotransporter NBC4d	SLC4A5				1
IPI00028050	Selenocysteine-specific elongation factor	EEFSEC	P57772		1	
IPI00873139	Isoform 2 of Beta-1,4-galactosyltransferase 3	B4GALT3	O60512		1	1
IPI00004944	Isoform 1 of Sodium-driven chloride bicarbonate exchanger	SLC4A10	Q6U841			1
IPI00304587	Protein dispatched homolog 2	DISP2	A7MBM2			1
IPI00023283	Isoform 2 of Titin	TTN	Q8WZ42		2	3
IPI00001328	Protein	-			4	3
IPI00219703	Parvalbumin alpha	PVALB	P20472			1
IPI00025461	Metallothionein-1E	MT1E	P04732		7	6
IPI00001813	Isoform 1 of RING and PHD-finger domain-containing protein KIAA1542	KIAA1542	Q9P1Y6			2
IPI00025295	Ectonucleoside triphosphate diphosphohydrolase 6	ENTPD6	O75354		1	4
IPI00059366	H2A histone family, member Y isoform 2	H2AFY			3	5
IPI00643747	Isoform 1 of Uncharacterized protein C22orf30	C22orf30	Q5THK1		2	1
IPI00024975	Kinesin-like protein KIF15	KIF15			2	1
IPI00216256	Isoform 2 of WD repeat-containing protein 1	WDR1	O75083		1	2
IPI00064606	Isoform 1 of Multiple epidermal growth factor-like domains 11 precursor	MEGF11	A6BM72		2	2
IPI00002689	Isoform Alpha of Short transient receptor potential channel 4	TRPC4	Q9UBN4		1	1
IPI00288940	Isoform 1 of Obscurin	OBSCN	Q5VST9		1	1
IPI00152033	Isoform 1 of Transient receptor potential cation channel subfamily M member 8	TRPM8	Q7Z2W7		1	
IPI00016604	Isoform PDE4B1 of cAMP-specific 3',5'-cyclic phosphodiesterase 4B	PDE4B	Q07343		2	1
IPI00005711	Histone deacetylase 6	HDAC6	Q9UBN7		2	
IPI00656052	SPANX-C	SPANXB2				1
IPI00658109	Creatine kinase, ubiquitous mitochondrial precursor	CKMT1A	P12532			1

Table S1.

Number of unique peptides identified

IPI	Protein name	Gene symbol	Swiss Prot ID	Normal	CFS	nPTLS
IPI00789564	Isoform 3 of Metallophosphoesterase 1	MPPE1	Q53F39		1	
IPI00413266	Isoform 1 of IQ and AAA domain-containing protein	IQCA	Q86XH1			1
IPI00011253	40S ribosomal protein S3	RPS3	P23396		1	
IPI00013260	Lymphocyte-specific protein 1	LSP1	P33241		1	1
IPI00454620	HERV-K_11q22.1 provirus ancestral Pol protein	-	P63136		1	
IPI00472374	zinc finger protein 98	ZNF98				1
IPI00009286	Isoform 1 of Zinc finger protein HRX	MLL	Q03164		1	2
IPI00337335	Isoform 1 of Myosin-14	MYH14	Q7Z406		1	
IPI00217442	ANKHD1-EIF4EBP3 protein	ANKHD1-EIF4EBP3			1	1
IPI00470596	Isoform 1 of Zinc finger protein 638	ZNF638	Q14966		1	1
IPI00103426	Isoform 1 of Solute carrier family 13 member 3	SLC13A3	Q8WWT9		1	
IPI00291839	Isoform 2 of Rap guanine nucleotide exchange factor 6	RAPGEF6	Q8TEU7		1	
IPI00829803	13 kDa protein	-			1	1
IPI00027258	Isoform 2 of Synaptopodin	SYNPO	Q8N3V7		1	1
