# Supplementary material for: Distinct Cerebrospinal Fluid Proteomes Differentiate Post-Treatment Lyme Disease from Chronic Fatigue Syndrome
Source: PLoS One. 2011 Feb 23;6(2):e17287. doi: 10.1371/journal.pone.0017287 (PMC3044169; doi:10.1371/journal.pone.0017287)
Supplement: Table S2 — Proteins (n = 474) identified in the analysis of non-fractionated and immunodepleted individual patient samples. (PDF) [file pone.0017287.s004.pdf]

Table S2. Proteins (n=474) identified in the analysis of non fractionated and immunodepleted individual patient samples.

IPI	protein name	Gene symbol	Swiss Prot ID
IPI00000044	Platelet-derived growth factor B chain precursor	PDGFB	P01127
IPI00000130	Somatostatin precursor	SST	P61278
IPI00000513	E-cadherin	CDH1	
IPI00000779	Isoform 1 of ADAM 22 precursor	ADAM22	Q9P0K1-1
IPI00000828	Proenkephalin A precursor	PENK	P01210
IPI00000874	Peroxiredoxin-1	PRDX1	Q06830
IPI00000877	Hypoxia up-regulated protein 1 precursor	HYOU1	Q9Y4L1
IPI00000878	Tyrosine-protein kinase Tec	TEC	P42680
IPI00000977	Mitogen-activated protein kinase kinase kinase 11	MAP3K11	Q16584
IPI00001477	Isoform 1 of Epithelial discoidin domain-containing receptor 1 precursor	DDR1	Q08345-1
IPI00001506	Neuropeptide Y precursor	NPY	P01303
IPI00001593	Lysosomal Pro-X carboxypeptidase precursor	PRCP	P42785
IPI00001611	Isoform 1 of Insulin-like growth factor II precursor	IGF2	P01344-1
IPI00001633	Leucine-rich repeat transmembrane protein FLRT2 precursor	FLRT2	O43155
IPI00001662	Opioid-binding protein/cell adhesion molecule precursor	OPCML	Q14982
IPI00001753	Myosin-4	MYH4	Q9Y623
IPI00001872	Isoform 1 of Protocadherin gamma C3 precursor	PCDHGC3	Q9UN70-1
IPI00001893	Isoform A of Protocadherin-7 precursor	PCDH7	O60245-1
IPI00001895	Isoform 1 of Protocadherin-8 precursor	PCDH8	O95206-1
IPI00001952	Endonuclease domain-containing 1 protein precursor	ENDOD1	O94919
IPI00002147	Chitinase-3-like protein 1 precursor	CHI3L1	P36222
IPI00002280	ProSAAS precursor	PCSK1N	Q9UHG2
IPI00002714	Dickkopf-related protein 3 precursor	DKK3	Q9UBP4
IPI00002745	Cathepsin Z precursor	CTSZ	Q9UBR2
IPI00002925	Cocaine- and amphetamine-regulated transcript protein precursor	CARTPT	Q16568
IPI00003176	Serine protease HTRA1 precursor	HTRA1	Q92743
IPI00003351	Extracellular matrix protein 1 precursor	ECM1	Q16610
IPI00003590	Isoform 1 of Sulfhydryl oxidase 1 precursor	QSOX1	O00391-1
IPI00003802	Alpha-mannosidase 2	MAN2A1	Q16706
IPI00003813	Isoform 1 of Cell adhesion molecule 1 precursor	CADM1	Q9BY67-1
IPI00003865	Isoform 1 of Heat shock cognate 71 kDa protein	HSPA8	P11142-1
IPI00003928	Dual specificity protein phosphatase 1	DUSP1	P28562
IPI00004346	C-C chemokine receptor type 10	CCR10	P46092

Table S2. Proteins (n=474) identified in the analysis of non fractionated and immunodepleted individual patient samples.

IPI	protein name	Gene symbol	Swiss Prot ID
IPI00004440	Receptor-type tyrosine-protein phosphatase-like N precursor	PTPRN	Q16849
IPI00004671	Golgin subfamily B member 1	GOLGB1	Q14789
IPI00004798	cDNA FLJ75207	CRISP3	P54108
IPI00005102	Spermine synthase	SMS	P52788
IPI00005222	Ephrin type-B receptor 6 precursor	EPHB6	O15197
IPI00005517	Ephrin-A5 precursor	EFNA5	P52803
IPI00005607	Isoform 1 of Deleted in bladder cancer protein 1 precursor	DBC1	O60477-1
IPI00005708	Heparan sulfate glucosamine 3-O-sulfotransferase 3B1	HS3ST3B1	Q9Y662
IPI00005774	Isoform 1 of Low-density lipoprotein receptor-related protein 8 precursor	LRP8	Q14114-1
IPI00006114	Pigment epithelium-derived factor precursor	SERPINF1	P36955
IPI00006166	Probable G-protein coupled receptor 37 precursor	GPR37	O15354
IPI00006482	Isoform Long of Sodium/potassium-transporting ATPase subunit alpha-1 precursor	ATP1A1	P05023-1
IPI00006601	Secretogranin-1 precursor	CHGB	P05060
IPI00006608	Isoform APP770 of Amyloid beta A4 protein precursor (Fragment)	APP	P05067-1
IPI00006662	Apolipoprotein D precursor	APOD	P05090
IPI00007082	Interleukin-5 precursor	IL5	P05113
IPI00007257	calsyntenin 1 isoform 2	CLSTN1	
IPI00007334	Isoform 1 of Apoptotic chromatin condensation inducer in the nucleus	ACIN1	Q9UKV3-1
IPI00007425	desmocollin 1 isoform Dsc1b preproprotein	DSC1	
IPI00007914	TTLL5 protein	TTLL5	
IPI00007921	Isoform 1 of Neurexin-2-alpha precursor	NRXN2	Q9P2S2-1
IPI00008085	Zinc transporter ZIP10 precursor	SLC39A10	Q9ULF5
IPI00008087	Follistatin-related protein 5 precursor	FSTL5	Q8N475
IPI00008107	Leucine-rich repeat and fibronectin type-III domain-containing protein 2 precursor	LRFN2	Q9ULH4
IPI00008318	Ephrin type-A receptor 4 precursor	EPHA4	P54764
IPI00008586	Isoform 1 of Chondroitin sulfate proteoglycan 5 precursor	CSPG5	O95196-1
IPI00008860	Isoform 1 of Complement C1q tumor necrosis factor-related protein 3 precursor	C1QTNF3	Q9BXJ4-1
IPI00008944	Isoform 1 of Neuroendocrine protein 7B2 precursor	SCG5	P05408-1
IPI00008994	Isoform 1 of Protein NDRG2	NDRG2	Q9UN36-1
IPI00009028	Tetranectin precursor	CLEC3B	P05452

Table S2. Proteins (n=474) identified in the analysis of non fractionated and immunodepleted individual patient samples.

IPI	protein name	Gene symbol	Swiss Prot ID
IPI00009030	Isoform LAMP-2A of Lysosome-associated membrane glycoprotein 2 precursor	LAMP2	P13473-1
IPI00009066	similar to protein tyrosine phosphatase, receptor type, Q isoform 1 precursor	PTPRQ	
IPI00009362	Secretogranin-2 precursor	SCG2	P13521
IPI00009477	Intercellular adhesion molecule 2 precursor	ICAM2	P13598
IPI00009619	Isoform 2 of Cell adhesion molecule 3 precursor	CADM3	Q8N126-2
IPI00009865	Keratin, type I cytoskeletal 10	KRT10	P13645
IPI00009950	Vesicular integral-membrane protein VIP36 precursor	LMAN2	Q12907
IPI00009960	Isoform 1 of Mitochondrial inner membrane protein	IMMT	Q16891-1
IPI00009997	N-acetyllactosaminide beta-1,3-N-acetylglucosaminyltransferase	B3GNT1	O43505
IPI00010136	Isoform 2 of C-terminal-binding protein 2	CTBP2	P56545-2
IPI00010317	Isoform 1 of Ras-related GTP-binding protein B	RRAGB	Q5VZM2-1
IPI00010402	Putative uncharacterized protein	SH3BGRL3	
IPI00010586	TATA element modulatory factor	TMF1	P82094
IPI00011140	Protein NOV homolog precursor	NOV	P48745
IPI00011218	Macrophage colony-stimulating factor 1 receptor precursor	CSF1R	P07333
IPI00011229	Cathepsin D precursor	CTSD	P07339
IPI00011261	Complement component C8 gamma chain precursor	C8G	P07360
IPI00011302	CD59 glycoprotein precursor	CD59	P13987
IPI00011454	Isoform 2 of Neutral alpha-glucosidase AB precursor	GANAB	Q14697-2
IPI00011694	Trypsin-1 precursor	PRSS1	P07477
IPI00012119	Isoform A of Decorin precursor	DCN	P07585-1
IPI00012386	Cochlin precursor	COCH	O43405
IPI00012503	Isoform Sap-mu-0 of Proactivator polypeptide precursor	PSAP	P07602-1
IPI00012545	Isoform TGN51 of Trans-Golgi network integral membrane protein 2 precursor	TGOLN2	O43493-1
IPI00012837	Kinesin heavy chain	KIF5B	P33176
IPI00012887	Cathepsin L1 precursor	CTSL1	P07711
IPI00013179	Prostaglandin-H2 D-isomerase precursor	PTGDS	P41222
IPI00013205	Isoform 1 of JmjC domain-containing histone demethylation protein 3B	JMJD2B	O94953-1
IPI00013299	Neuroblastoma, suppression of tumorigenicity 1	NBL1	

Table S2. Proteins (n=474) identified in the analysis of non fractionated and immunodepleted individual patient samples.

IPI	protein name	Gene symbol	Swiss Prot ID
IPI00013303	Limbic system-associated membrane protein precursor	LSAMP	Q13449
IPI00014048	Ribonuclease pancreatic precursor	RNASE1	P07998
IPI00014439	Dihydropteridine reductase	QDPR	P09417
IPI00014572	SPARC precursor	SPARC	P09486
IPI00014826	Isoform 2 of Zinc finger protein basonuclin-2	BNC2	Q6ZN30-2
IPI00014964	Lymphocyte antigen 6H precursor	LY6H	O94772
IPI00015102	Isoform 1 of CD166 antigen precursor	ALCAM	Q13740-1
IPI00015260	Protein kinase C-binding protein NELL2 precursor	NELL2	Q99435
IPI00015881	Isoform 1 of Macrophage colony-stimulating factor 1 precursor	CSF1	P09603-1
IPI00016150	Neuroserpin precursor	SERPINI1	Q99574
IPI00016334	Isoform 1 of Cell surface glycoprotein MUC18 precursor	MCAM	P43121-1
IPI00016645	Isoform 1 of Ephrin type-A receptor 7 precursor	EPHA7	Q15375-1
IPI00016679	SLIT and NTRK-like protein 5 precursor	SLITRK5	O94991
IPI00016915	Insulin-like growth factor-binding protein 7 precursor	IGFBP7	Q16270
IPI00017569	Fas apoptotic inhibitory molecule 2	FAIM2	Q9BWQ8
IPI00017601	Ceruloplasmin precursor	CP	P00450
IPI00017696	Complement C1s subcomponent precursor	C1S	P09871
IPI00018136	Isoform 1 of Vascular cell adhesion protein 1 precursor	VCAM1	P19320-1
IPI00018236	Ganglioside GM2 activator precursor	GM2A	P17900
IPI00019158	ADAM metallopeptidase domain 8 precursor	ADAM8	P78325
IPI00019176	Retinoic acid receptor responder protein 2 precursor	RARRES2	Q99969
IPI00019372	Serglycin precursor	SRGN	P10124
IPI00019568	Prothrombin precursor (Fragment)	F2	P00734
IPI00019580	Plasminogen precursor	PLG	P00747
IPI00019581	Coagulation factor XII precursor	F12	P00748
IPI00019591	Isoform 1 of Complement factor B precursor (Fragment)	CFB	P00751-1
IPI00019771	Fractalkine precursor	CX3CL1	P78423
IPI00019943	Afamin precursor	AFM	P43652
IPI00020091	Alpha-1-acid glycoprotein 2 precursor	ORM2	P19652
IPI00020557	Prolow-density lipoprotein receptor-related protein 1 precursor	LRP1	Q07954
IPI00020879	Chloride channel protein ClC-Ka	CLCNKA	P51800
IPI00020986	Lumican precursor	LUM	P51884

Table S2. Proteins (n=474) identified in the analysis of non fractionated and immunodepleted individual patient samples.

IPI	protein name	Gene symbol	Swiss Prot ID
IPI00020990	Osteomodulin precursor	OMD	Q99983
IPI00021304	Keratin, type II cytoskeletal 2 epidermal	KRT2	P35908
IPI00021485	Leucine-rich repeat neuronal protein 1 precursor	LRRN1	Q6UXK5
IPI00021733	Bifunctional heparan sulfate N-deacetylase/N-sulfotransferase 4	NDST4	Q9H3R1
IPI00021841	Apolipoprotein A-I precursor	APOA1	P02647
IPI00021842	Apolipoprotein E precursor	APOE	P02649
IPI00021854	Apolipoprotein A-II precursor	APOA2	P02652
IPI00021855	Apolipoprotein C-I precursor	APOC1	P02654
IPI00021857	Apolipoprotein C-III precursor	APOC3	P02656
IPI00021885	Isoform 1 of Fibrinogen alpha chain precursor	FGA	P02671-1
IPI00021891	Isoform Gamma-B of Fibrinogen gamma chain precursor	FGG	P02679-1
IPI00021903	Isoform Alpha of ADAM 23 precursor	ADAM23	O75077-1
IPI00021997	Protein CREG1 precursor	CREG1	O75629
IPI00022200	alpha 3 type VI collagen isoform 1 precursor	COL6A3	P12111-1
IPI00022284	Major prion protein precursor	PRNP	P04156
IPI00022331	Phosphatidylcholine-sterol acyltransferase precursor	LCAT	P04180
IPI00022371	Histidine-rich glycoprotein precursor	HRG	P04196
IPI00022392	Complement C1q subcomponent subunit A precursor	C1QA	P02745
IPI00022394	Complement C1q subcomponent subunit C precursor	C1QC	P02747
IPI00022395	Complement component C9 precursor	C9	P02748
IPI00022417	Leucine-rich alpha-2-glycoprotein precursor	LRG1	P02750
IPI00022418	Isoform 1 of Fibronectin precursor	FN1	P02751-1
IPI00022420	Plasma retinol-binding protein precursor	RBP4	P02753
IPI00022426	AMBP protein precursor	AMBP	P02760
IPI00022429	Alpha-1-acid glycoprotein 1 precursor	ORM1	P02763
IPI00022431	Alpha-2-HS-glycoprotein precursor	AHSG	P02765
IPI00022432	Transthyretin precursor	TTR	P02766
IPI00022463	Serotransferrin precursor	TF	P02787
IPI00022488	Hemopexin precursor	HPX	P02790
IPI00022542	Rho-associated protein kinase 1	ROCK1	Q13464
IPI00022792	Microfibril-associated glycoprotein 4 precursor	MFAP4	P55083
IPI00022892	Thy-1 membrane glycoprotein precursor	THY1	P04216
IPI00022895	Alpha-1B-glycoprotein precursor	A1BG	P04217
IPI00022937	Coagulation factor V	F5	

Table S2. Proteins (n=474) identified in the analysis of non fractionated and immunodepleted individual patient samples.

IPI	protein name	Gene symbol	Swiss Prot ID
IPI00023555	Isoform 1a of Oxysterol-binding protein-related protein 3	OSBPL3	Q9H4L5-1
IPI00023608	Fanconi anemia group C protein	FANCC	Q00597
IPI00023648	Immunoglobulin superfamily containing leucine-rich repeat protein precursor	ISLR	O14498
IPI00023673	Galectin-3-binding protein precursor	LGALS3BP	Q08380
IPI00023814	Isoform 1 of Neogenin precursor	NEO1	Q92859-1
IPI00023845	Kallikrein-6 precursor	KLK6	Q92876
IPI00024046	Cadherin-13 precursor	CDH13	P55290
IPI00024284	Basement membrane-specific heparan sulfate proteoglycan core protein precursor	HSPG2	P98160
IPI00024966	Contactin-2 precursor	CNTN2	Q02246
IPI00025257	Semaphorin-7A precursor	SEMA7A	O75326
IPI00025465	Mimecan precursor	OGN	P20774
IPI00025732	Latent transforming growth factor-beta binding protein 4	LTBP4	
IPI00026174	Cholecystokinins precursor	CCK	P06307
IPI00026197	Similar to Ig kappa chain V-IV region precursor	IGKV4-1	P06312
IPI00026199	Glutathione peroxidase 3 precursor	GPX3	P22352
IPI00026314	Isoform 1 of Gelsolin precursor	GSN	P06396-1
IPI00026800	Scrapie-responsive protein 1 precursor	SCRG1	O75711
IPI00027038	Isoform 1 of V-set and immunoglobulin domain-containing protein 4 precursor	VSIG4	Q9Y279-1
IPI00027139	Inositol polyphosphate 1-phosphatase	INPP1	P49441
IPI00027166	Metalloproteinase inhibitor 2 precursor	TIMP2	P16035
IPI00027228	Probable glutamyl-tRNA(Gln) amidotransferase subunit B, mitochondrial precursor	PET112L	O75879
IPI00027235	Isoform 1 of Attractin precursor	ATRN	O75882-1
IPI00027377	aggrecan isoform 2 precursor	ACAN	P16112-1
IPI00027482	Corticosteroid-binding globulin precursor	SERPINA6	P08185
IPI00027827	Extracellular superoxide dismutase [Cu-Zn] precursor	SOD3	P08294
IPI00027848	Macrophage mannose receptor 1 precursor	MRC1	P22897
IPI00027851	Beta-hexosaminidase alpha chain precursor	HEXA	P06865
IPI00028318	Isoform 1 of Phosphatase and actin regulator 1	PHACTR1	Q9C0D0-1
IPI00028344	Isoform 3 of BR serine/threonine-protein kinase 2	BRSK2	Q8IWQ3-3
IPI00028908	Nidogen-2 precursor	NID2	Q14112
IPI00028911	Dystroglycan precursor	DAG1	Q14118

Table S2. Proteins (n=474) identified in the analysis of non fractionated and immunodepleted individual patient samples.

IPI	protein name	Gene symbol	Swiss Prot ID
IPI00029061	Selenoprotein P precursor	SEPP1	P49908
IPI00029235	Insulin-like growth factor-binding protein 6 precursor	IGFBP6	P24592
IPI00029260	Monocyte differentiation antigen CD14 precursor	CD14	P08571
IPI00029658	Isoform 1 of EGF-containing fibulin-like extracellular matrix protein 1 precursor	EFEMP1	Q12805-1
IPI00029700	Isoform Long of Down syndrome cell adhesion molecule precursor	DSCAM	O60469-1
IPI00029739	Isoform 1 of Complement factor H precursor	CFH	P08603-1
IPI00029751	Isoform 1 of Contactin-1 precursor	CNTN1	Q12860-1
IPI00029863	SERPINF2 protein	SERPINF2	
IPI00029997	6-phosphogluconolactonase	PGLS	O95336
IPI00030255	Procollagen-lysine,2-oxoglutarate 5-dioxygenase 3 precursor	PLOD3	O60568
IPI00030648	Isoform 1 of Zinc finger FYVE domain-containing protein 9	ZFYVE9	O95405-1
IPI00030783	Signal transducer and activator of transcription 5A	STAT5A	P42229
IPI00031030	Isoform 1 of Amyloid-like protein 2 precursor	APLP2	Q06481-1
IPI00031121	Carboxypeptidase E precursor	CPE	P16870
IPI00031461	Rab GDP dissociation inhibitor beta	GDI2	P50395
IPI00031545	Isoform Long of Inositol 1,4,5-trisphosphate receptor type 2	ITPR2	Q14571-1
IPI00031769	Esophageal cancer-related gene 4 protein precursor	C2orf40	Q9H1Z8
IPI00031821	Integral membrane protein 2B	ITM2B	Q9Y287
IPI00032158	Isoform 2 of NMDA receptor-regulated protein 1	NARG1	Q9BXJ9-4
IPI00032179	Antithrombin III variant	SERPINC1	P01008
IPI00032220	Angiotensinogen precursor	AGT	P01019
IPI00032291	Complement C5 precursor	C5	P01031
IPI00032292	Metalloproteinase inhibitor 1 precursor	TIMP1	P01033
IPI00032293	Cystatin-C precursor	CST3	P01034
IPI00033466	C-type lectin domain family 11 member A precursor	CLEC11A	Q9Y240
IPI00034319	Isoform A of Protein CutA precursor	CUTA	O60888-2
IPI00034558	Neurexin-3-beta precursor	NRXN3	Q9HDB5
IPI00042444	Zinc finger, C2H2-type domain containing protein	ZNF799	
IPI00044369	Isoform 1 of Plexin domain-containing protein 2 precursor	PLXDC2	Q6UX71-1

Table S2. Proteins (n=474) identified in the analysis of non fractionated and immunodepleted individual patient samples.

IPI	protein name	Gene symbol	Swiss Prot ID
IPI00056478	Isoform 1 of Immunoglobulin superfamily member 8 precursor	IGSF8	Q969P0-1
IPI00062511	Isoform 1 of Tubulin polyglutamylase complex subunit 1	C19orf20	Q6ZTW0-1
IPI00063048	Isoform 2 of Beta-galactoside alpha-2,6-sialyltransferase 2	ST6GAL2	Q96JF0-2
IPI00064158	Isoform 1 of Tau-tubulin kinase 1	TTBK1	Q5TCY1-1
IPI00064655	Cytochrome c oxidase subunit 4 isoform 2, mitochondrial precursor	COX4I2	Q96KJ9
IPI00064667	Beta-Ala-His dipeptidase precursor	CNDP1	Q96KN2
IPI00065428	Coiled-coil domain-containing protein 11	CCDC11	Q96M91
IPI00069058	VGF nerve growth factor inducible precursor	VGF	
IPI00099834	Ubinuclein	UBN1	Q9NPG3
IPI00100160	Isoform 1 of Cullin-associated NEDD8-dissociated protein 1	CAND1	Q86VP6-1
IPI00102543	SLIT and NTRK-like protein 1 precursor	SLITRK1	Q96PX8
IPI00104074	Isoform 1 of Scavenger receptor cysteine-rich type 1 protein M130 precursor	CD163	Q86VB7-1
IPI00107819	PTPRD protein	PTPRD	
IPI00152418	Decay-accelerating factor splicing variant 4	CD55	
IPI00152491	CD99 antigen-like 2 isoform E3'-E4'-E3-E4	CD99L2	
IPI00153049	Isoform 2 of Matrix-remodeling-associated protein 8 precursor	MXRA8	Q9BRK3-2
IPI00154734	seizure related 6 homolog isoform 1	SEZ6	
IPI00156171	Isoform 1 of Ectonucleotide pyrophosphatase/phosphodiesterase family member 2 precursor	ENPP2	Q13822-1
IPI00157417	Isoform 4 of Seizure 6-like protein precursor	SEZ6L	Q9BYH1-1
IPI00159927	Neurocan core protein precursor	NCAN	O14594
IPI00162549	SP110 nuclear body protein isoform c	SP110	Q9HB58-1
IPI00163207	Isoform 1 of N-acetylmuramoyl-L-alanine amidase precursor	PGLYRP2	Q96PD5-1
IPI00163563	PEBP family protein precursor	PEBP4	Q96S96
IPI00165009	Isoform 3 of MBT domain-containing protein 1	MBTD1	Q05BQ5-3
IPI00165459	similar to SET domain containing 1A	SETD1B	
IPI00165972	Complement factor D preproprotein	CFD	P00746
IPI00166729	alpha-2-glycoprotein 1, zinc	AZGP1	P25311
IPI00166766	hypothetical protein LOC146556 isoform 2	MGC45438	
IPI00167639	Vacuolar proton pump subunit d 2	ATP6V0D2	Q8N8Y2
IPI00168920	collagen, type XXIV, alpha 1	COL24A1	
IPI00170791	Isoform 2 of InaD-like protein	INADL	Q8NI35-2

Table S2. Proteins (n=474) identified in the analysis of non fractionated and immunodepleted individual patient samples.

IPI	protein name	Gene symbol	Swiss Prot ID
IPI00171473	Spondin-1 precursor	SPON1	Q9HCB6
IPI00176221	Neuronal growth regulator 1 precursor	NEGR1	Q7Z3B1
IPI00176427	Cell adhesion molecule 4 precursor	CADM4	Q8NFZ8
IPI00177543	peptidylglycine alpha-amidating monooxygenase isoform a, preproprotein	PAM	P19021-1
IPI00178854	Isoform 1 of Contactin-4 precursor	CNTN4	Q8IWV2-1
IPI00179415	Isoform 1 of Serine/threonine-protein phosphatase 2B catalytic subunit alpha isoform	PPP3CA	Q08209-1
IPI00183445	Isoform 1 of Latrophilin-1 precursor	LPHN1	O94910-1
IPI00185361	ATP-dependent RNA helicase DDX55	DDX55	Q8NHQ9
IPI00215894	Isoform LMW of Kininogen-1 precursor	KNG1	P01042-2
IPI00216171	Gamma-enolase	ENO2	P09104
IPI00216250	Cell recognition protein CASPR4	CNTNAP4	Q9C0A0
IPI00216691	Profilin-1	PFN1	P07737
IPI00216704	Isoform 2 of Spectrin beta chain, erythrocyte	SPTB	P11277-2
IPI00216990	hypothetical protein LOC158358	KIAA2026	
IPI00217269	Guanine nucleotide-binding protein G(t) subunit alpha-2	GNAT2	P19087
IPI00217519	Ras-related protein Ral-A precursor	RALA	P11233
IPI00217778	Isoform 2 of Phospholipid transfer protein precursor	PLTP	P55058-2
IPI00217787	Isoform 1 of Uncharacterized protein C12orf53 precursor	C12orf53	Q8IYJ0-1
IPI00217966	Isoform 1 of L-lactate dehydrogenase A chain	LDHA	P00338-1
IPI00218075	Protein FAM9B	FAM9B	Q8IZU0
IPI00218192	Isoform 2 of Inter-alpha-trypsin inhibitor heavy chain H4 precursor	ITIH4	Q14624-2
IPI00218413	biotinidase precursor	BTD	P43251
IPI00218732	Serum paraoxonase/arylesterase 1	PON1	P27169
IPI00218733	Superoxide dismutase	SOD1	P00441
IPI00218823	Isoform 1 of WW domain-binding protein 7	MLL4	Q9UMN6-1
IPI00219018	Glyceraldehyde-3-phosphate dehydrogenase	GAPDH	P04406
IPI00219217	L-lactate dehydrogenase B chain	LDHB	P07195
IPI00219219	Galectin-1	LGALS1	P09382
IPI00219446	Phosphatidylethanolamine-binding protein 1	PEBP1	P30086
IPI00219798	Isoform 1 of Roundabout homolog 1 precursor	ROBO1	Q9Y6N7-1
IPI00219910	22 kDa protein	-	
IPI00220117	Uncharacterized protein CD99	CD99	P14209-2
IPI00220327	Keratin, type II cytoskeletal 1	KRT1	P04264

Table S2. Proteins (n=474) identified in the analysis of non fractionated and immunodepleted individual patient samples.

IPI	protein name	Gene symbol	Swiss Prot ID
IPI00220334	Isoform 3 of Seizure 6-like protein precursor	SEZ6L	Q9BYH1-4
IPI00220562	Neuronal pentraxin-1 precursor	NPTX1	Q15818
IPI00220644	Isoform M1 of Pyruvate kinase isozymes M1/M2	PKM2	P14618-2
IPI00221088	40S ribosomal protein S9	RPS9	P46781
IPI00221224	Aminopeptidase N	ANPEP	P15144
IPI00235647	similar to fibrillarin	LOC345630	
IPI00241562	reelin isoform a	RELN	P78509-2
IPI00242956	IgGFc-binding protein precursor	FCGBP	Q9Y6R7
IPI00243221	nardilysin (N-arginine dibasic convertase) isoform a	NRD1	O43847-2
IPI00247243	31 kDa protein	-	
IPI00289058	Ly-6/neurotoxin-like protein 1 precursor	LYNX1	Q9BZG9
IPI00289275	Cartilage intermediate layer protein 1 precursor	CILP	O75339
IPI00290085	Cadherin-2 precursor	CDH2	P19022
IPI00290315	Chromogranin-A precursor	CHGA	P10645
IPI00291136	Collagen alpha-1(VI) chain precursor	COL6A1	P12109
IPI00291262	Clusterin precursor	CLU	P10909
IPI00291866	Plasma protease C1 inhibitor precursor	SERPING1	P05155
IPI00292071	Secretogranin-3 precursor	SCG3	Q8WXD2
IPI00292181	Mitogen-activated protein kinase kinase kinase 12	MAP3K12	Q12852
IPI00292300	contactin associated protein-like 5	CNTNAP5	
IPI00292530	Inter-alpha-trypsin inhibitor heavy chain H1 precursor	ITIH1	P19827
IPI00292567	Protein O-linked mannose beta1,2-N-acetylglucosaminyltransferase	POMGNT1	
IPI00292946	Thyroxine-binding globulin precursor	SERPINA7	P05543
IPI00292950	Serpin peptidase inhibitor, clade D (Heparin cofactor), member 1	SERPIND1	
IPI00293057	Isoform 2 of Carboxypeptidase B2 precursor	CPB2	Q96IY4-2
IPI00293533	Nuclear pore glycoprotein p62	NUP62	P37198
IPI00293539	Isoform 2 of Cadherin-11 precursor	CDH11	P55287-2
IPI00293665	Keratin, type II cytoskeletal 6B	KRT6B	P04259
IPI00293836	Isoform 3 of Cell adhesion molecule 2 precursor	CADM2	Q8N3J6-3
IPI00294004	Vitamin K-dependent protein S precursor	PROS1	P07225
IPI00295414	Collagen alpha-1(XV) chain precursor	COL15A1	P39059
IPI00295542	Nucleobindin-1 precursor	NUCB1	Q02818
IPI00295741	Cathepsin B precursor	CTSB	P07858
IPI00295832	Oligodendrocyte-myelin glycoprotein precursor	OMG	P23515

Table S2. Proteins (n=474) identified in the analysis of non fractionated and immunodepleted individual patient samples.

IPI	protein name	Gene symbol	Swiss Prot ID
IPI00296141	Dipeptidyl-peptidase 2 precursor	DPP7	Q9UHL4
IPI00296165	Complement C1r subcomponent precursor	C1R	P00736
IPI00296534	Isoform D of Fibulin-1 precursor	FBLN1	P23142-1
IPI00296537	Isoform C of Fibulin-1 precursor	FBLN1	P23142-4
IPI00296608	Complement component C7 precursor	C7	P10643
IPI00296777	SPARC-like protein 1 precursor	SPARCL1	Q14515
IPI00297124	Isoform 1 of Interleukin-6 receptor subunit beta precursor	IL6ST	P40189-1
IPI00297160	Isoform 12 of CD44 antigen precursor	CD44	P16070-12
IPI00297188	Brain-specific angiogenesis inhibitor 2 precursor	BAI2	O60241
IPI00297224	Sushi domain-containing protein 5	SUSD5	O60279
IPI00297252	Isoform 1 of Extracellular sulfatase Sulf-2 precursor	SULF2	Q8IWU5-1
IPI00297284	Insulin-like growth factor-binding protein 2 precursor	IGFBP2	P18065
IPI00297646	Collagen alpha-1(I) chain precursor	COL1A1	P02452
IPI00298237	Isoform 1 of Tripeptidyl-peptidase 1 precursor	TPP1	O14773-1
IPI00298281	Laminin subunit gamma-1 precursor	LAMC1	P11047
IPI00298388	Isoform 1 of Phosphoinositide-3-kinase-interacting protein 1 precursor	PIK3IP1	Q96FE7-1
IPI00298497	Fibrinogen beta chain precursor	FGB	P02675
IPI00298547	Protein DJ-1	PARK7	Q99497
IPI00298793	Beta-mannosidase precursor	MANBA	O00462
IPI00298828	Beta-2-glycoprotein 1 precursor	APOH	P02749
IPI00298971	Vitronectin precursor	VTN	P04004
IPI00299059	Isoform 2 of Neural cell adhesion molecule L1-like protein precursor	CHL1	O00533-2
IPI00299699	Neural proliferation differentiation and control protein 1 precursor	NPDC1	Q9NQX5
IPI00299738	Procollagen C-endopeptidase enhancer 1 precursor	PCOLCE	Q15113
IPI00301143	Isoform 1 of Peptidase inhibitor 16 precursor	PI16	Q6UXB8-1
IPI00301494	Carbohydrate sulfotransferase 4	CHST4	Q8NCG5
IPI00301579	Epididymal secretory protein E1 precursor	NPC2	P61916
IPI00301743	Putative uncharacterized protein DKFZp434P055	FLJ39660	
IPI00301865	Isoform 1 of Transmembrane protein 132A precursor	TMEM132A	Q24JP5-1
IPI00302592	filamin A, alpha isoform 1	FLNA	
IPI00304273	Apolipoprotein A-IV precursor	APOA4	P06727
IPI00304379	Ubiquitin carboxyl-terminal hydrolase 1	USP1	O94782
IPI00304962	Collagen alpha-2(I) chain precursor	COL1A2	P08123

Table S2. Proteins (n=474) identified in the analysis of non fractionated and immunodepleted individual patient samples.

IPI	protein name	Gene symbol	Swiss Prot ID
IPI00305380	Insulin-like growth factor-binding protein 4 precursor	IGFBP4	P22692
IPI00305461	Inter-alpha-trypsin inhibitor heavy chain H2 precursor	ITIH2	P19823
IPI00306339	secreted phosphoprotein 1 isoform b	SPP1	
IPI00307591	Zinc finger protein 609	ZNF609	O15014
IPI00328243	Phospholipase D3	PLD3	Q8IV08
IPI00328609	Kallistatin precursor	SERPINA4	P29622
IPI00329688	Protein YIPF3	YIPF3	Q9GZM5
IPI00332887	signal-regulatory protein alpha precursor	SIRPA	P78324-3
IPI00333140	Delta and Notch-like epidermal growth factor-related receptor precursor	DNER	Q8NFT8
IPI00333776	Isoform 1 of Neuronal cell adhesion molecule precursor	NRCAM	Q92823-1
IPI00334238	neuronal pentraxin receptor	NPTXR	O95502
IPI00334282	Protein FAM3C precursor	FAM3C	Q92520
IPI00334666	Isoform 1 of Receptor-type tyrosine-protein phosphatase N2 precursor	PTPRN2	Q92932-1
IPI00335343	similar to Dynein heavy chain at 36C CG5526-PA	DNAH14	
IPI00337307	Isoform 1 of HpaII tiny fragments locus 9c protein (Fragment)	HTF9C	Q8IZ69-1
IPI00374065	similar to melanoma inhibitory activity 3 isoform 1	MIA3	
IPI00374563	Agrin precursor	AGRN	O00468
IPI00374914	hypothetical protein	LOC401115	
IPI00376427	Neural cell adhesion molecule 2 precursor	NCAM2	O15394
IPI00376436	Isoform 4 of Vacuolar protein sorting-associated protein 13B	VPS13B	Q7Z7G8-4
IPI00382471	Ig heavy chain V-I region WOL	-	P01760
IPI00382748	Isoform 3 of Putative hydroxypyruvate isomerase	HYI	Q5T013-3
IPI00384697	Isoform 2 of Serum albumin precursor	ALB	P02768-2
IPI00386879	CDNA FLJ14473 fis, clone MAMMA1001080, highly similar to Homo sapiens SNC73 protein (SNC73) mRNA	IGHV3OR16-13	
IPI00386946	CDNA FLJ11786 fis, clone HEMBA1006036	CCDC90A	
IPI00387113	Ig kappa chain V-III region B6	-	P01619
IPI00395667	Interferon-related IFRD2 (PC4-B) protein	NAT6	Q12894
IPI00396423	Alcadein beta	CLSTN3	
IPI00396439	Uncharacterized protein C6orf168	C6orf168	Q5TGI0
IPI00398007	ubiquitin specific protease 40	USP40	Q9NVE5-1
IPI00399180	Serine/threonine-protein kinase SBK1	SBK1	Q52WX2
IPI00401283	Multiple epidermal growth factor-like domains 9 precursor	MEGF9	Q9H1U4

Table S2. Proteins (n=474) identified in the analysis of non fractionated and immunodepleted individual patient samples.

IPI	protein name	Gene symbol	Swiss Prot_ID
IPI00401753	AT rich interactive domain 1A	ARID1A	
IPI00401776	mucin 6, gastric	MUC6	Q6W4X9
IPI00402004	56 kDa protein	-	
IPI00402293	Arylsulfatase G precursor	ARSG	Q96EG1
IPI00410600	Isoform 3 of Voltage-dependent calcium channel subunit alpha-2/delta-2 precursor	CACNA2D2	Q9NY47-3
IPI00410714	Hemoglobin subunit alpha	HBA2	P69905
IPI00411298	Connector enhancer of kinase suppressor of ras 3	CNKSR3	Q6P9H4
IPI00412107	FLJ43980 protein	FLJ43980	
IPI00412264	Pleiotrophin precursor	PTN	P21246
IPI00413272	Isoform 3 of Mediator of RNA polymerase II transcription subunit 23	MED23	Q9ULK4-3
IPI00414249	Isoform 1 of Neurexin-3-alpha precursor	NRXN3	Q9Y4C0-1
IPI00414896	Isoform 1 of Ribonuclease T2 precursor	RNASET2	O00584-1
IPI00418163	C4B1	C4B	
IPI00418262	Fructose-bisphosphate aldolase C	ALDOC	P09972
IPI00418531	Isoform 1 of Gliomedin	GLDN	Q6ZMI3-1
IPI00418931	CDNA FLJ45139 fis, clone BRAWH3039623	FLJ45139	
IPI00419442	IGLV6-57 protein	IGLV6-57	
IPI00419595	Isoform 1 of Podocalyxin-like protein 2 precursor	PODXL2	Q9NZ53-1
IPI00426051	Putative uncharacterized protein DKFZp686C15213	-	
IPI00427330	Shwachman-Bodian-Diamond syndrome protein	SBDS	Q9Y3A5
IPI00430842	IGHA1 protein	IGHV3OR16-13	
IPI00435020	Neural cell adhesion molecule 1, 140 kDa isoform precursor	NCAM1	P13591
IPI00442297	Isoform 2 of Neurotrimin precursor	HNT	Q9P121-2
IPI00442299	Isoform 1 of Neurexin-1-alpha precursor	NRXN1	Q9ULB1-1
IPI00451624	Isoform 1 of Cartilage acidic protein 1 precursor	CRTAC1	Q9NQ79-1
IPI00456623	Isoform 1 of Brevican core protein precursor	BCAN	Q96GW7-1
IPI00456629	Zinc finger protein 534 (Fragment)	ZNF534	
IPI00465028	Isoform 1 of Triosephosphate isomerase	TPI1	P60174-1
IPI00465184	Guanine deaminase	GDA	Q9Y2T3
IPI00465439	Fructose-bisphosphate aldolase A	ALDOA	P04075
IPI00470535	Dihydropyridine receptor alpha 2 subunit	CACNA2D1	
IPI00470625	Neuritin precursor	NRN1	Q9NPD7
IPI00470716	Isoform 2 of Neuroendocrine protein 7B2 precursor	SCG5	P05408-2

Table S2. Proteins (n=474) identified in the analysis of non fractionated and immunodepleted individual patient samples.

IPI	protein name	Gene symbol	Swiss Prot ID
IPI00477992	complement component 1, q subcomponent, B chain precursor	C1QB	P02746
IPI00478003	Alpha-2-macroglobulin precursor	A2M	P01023
IPI00514676	myelin oligodendrocyte glycoprotein isoform beta2 precursor	MOG	
IPI00550162	IGLV3-25 protein	IGLV3-25	
IPI00550991	Alpha-1-antichymotrypsin precursor	SERPINA3	P01011-2
IPI00552771	V2-11 protein	IGLV3-16	
IPI00552905	Isoform 1 of Proline-rich transmembrane protein 3 precursor	PRRT3	Q5FWE3-1
IPI00553177	Isoform 1 of Alpha-1-antitrypsin precursor	SERPINA1	P01009-1
IPI00555812	Vitamin D-binding protein precursor	GC	P02774
IPI00607580	multiple EGF-like-domains 8	MEGF8	Q7Z7M0-2
IPI00607600	amyloid precursor-like protein 1 isoform 1 precursor	APLP1	
IPI00640703	Isoform 1 of Exportin-5	XPO5	Q9HAV4-1
IPI00641737	Haptoglobin precursor	HP	P00738
IPI00642632	C7 protein	-	
IPI00643920	Transketolase	TKT	P29401
IPI00646328	FERM and PDZ domain-containing protein 1	FRMPD1	Q5SYB0
IPI00654755	Hemoglobin subunit beta	HBB	P68871
IPI00654875	Complement C4-B precursor	C4B	P0C0L5
IPI00654888	Plasma kallikrein precursor	KLKB1	P03952
IPI00656087	Isoform 1 of Tyrosine-protein phosphatase non-receptor type substrate 1 precursor	SIRPA	P78324-1
IPI00657936	collagen, type XXVIII precursor	COL28A1	
IPI00735451	Uncharacterized protein ENSP00000375035	-	
IPI00741266	similar to MEGF11 protein	LOC645302	
IPI00742696	vitamin D-binding protein precursor	GC	
IPI00744070	RING finger protein 43 precursor	RNF43	Q68DV7
IPI00745833	similar to Doublecortin domain-containing protein 2	LOC728597	
IPI00746623	Hyaluronan-binding protein 2 precursor	HABP2	Q14520
IPI00748265	Rheumatoid factor RF-ET13	-	
IPI00748312	protein tyrosine phosphatase, receptor-type, zeta1 precursor	PTPRZ1	P23471-1
IPI00783399	Isoform 1 of Transmembrane protein 132D precursor	TMEM132D	Q14C87-1
IPI00783987	Complement C3 precursor (Fragment)	C3	P01024
IPI00784119	Vacuolar ATP synthase subunit S1 precursor	ATP6AP1	Q15904
IPI00784430	Similar to Ig kappa chain V-III region VG precursor	IGKV3D-11	P04433

Table S2. Proteins (n=474) identified in the analysis of non fractionated and immunodepleted individual patient samples.

IPI	protein name	Gene symbol	Swiss Prot ID
IPI00784519	Putative uncharacterized protein	-	
IPI00784589	Putative uncharacterized protein DKFZp781M0386	-	
IPI00784828	Putative uncharacterized protein DKFZp686C11235	-	
IPI00786880	Myosin-XVB	-	Q96JP2
IPI00786926	Myosin-reactive immunoglobulin heavy chain variable region (Fragment)	IGHV1-69	
IPI00787641	similar to zinc finger protein 586	LOC729943	
IPI00787853	Inositol monophosphatase 3	IMPAD1	Q9NX62
IPI00789234	Immunoglobulin V-set domain containing protein	VSTM2A	Q8TAG5
IPI00790257	5 kDa protein	C3orf65	
IPI00792115	Putative uncharacterized protein DKFZp686H17246	CLEC3B	
IPI00794070	CFI protein	CFI	
IPI00794237	Protein	CALR	
IPI00796379	B2M protein	B2M	
IPI00798235	Isoform 32 of Voltage-dependent L-type calcium channel subunit alpha-1C	CACNA1C	Q13936-32
IPI00798430	Transferrin variant (Fragment)	TF	
IPI00829740	V2-6 protein	-	
IPI00829841	13 kDa protein	-	
IPI00844156	SERPINC1 protein	SERPINC1	
IPI00845354	IGKC protein	IGKC	
IPI00852898	Isoform 4 of Uncharacterized protein C14orf138	C14orf138	Q9H867-4
IPI00853369	Plexin-B2 precursor	PLXNB2	O15031
IPI00854644	Uncharacterized protein ENSP00000374805	-	
IPI00855918	mucin 5, subtype B, tracheobronchial	MUC5B	Q9HC84
IPI00873544	Uncharacterized protein ENSP00000380627 (Fragment)	-	
IPI00877800	32 kDa protein	-	
IPI00879442	29 kDa protein	KLHL22	
IPI00884080	Similar to Immunglobulin heavy chain variable region	-	
