# Supplementary material for: Distinct Cerebrospinal Fluid Proteomes Differentiate Post-Treatment Lyme Disease from Chronic Fatigue Syndrome
Source: PLoS One. 2011 Feb 23;6(2):e17287. doi: 10.1371/journal.pone.0017287 (PMC3044169; doi:10.1371/journal.pone.0017287)
Supplement: Table S3 — Proteins (n = 59) that were quantified and determined to be significantly different in abundance by ANOVA (p ≤ 0.01) when comparing CFS from nPTLS subject samples and allow for separation of these two syndromes when performing unsupervised hierarchical cluster analysis. (PDF) [file pone.0017287.s005.pdf]

Table S3. Proteins (n=59) that were quantified and determined to be significantly different in abundance by ANOVA (p ≤ 0.01) when comparing *CFS* from *nPTLS* subject samples and allow for separation of these two syndromes when performing unsupervised hierarchical cluster analysis.

IPI	Protein name	Gene symbol	Swiss Prot ID	p value	q value
IPI00243221	nardilysin (N-arginine dibasic convertase) isoform a	NRD1	O43847	4.3E-05	7.6E-03
IPI00011454	Isoform 2 of Neutral alpha-glucosidase AB precursor	GANAB	Q14697	1.6E-04	8.5E-03
IPI00107819	PTPRD protein	PTPRD		1.6E-04	8.5E-03
IPI00298547	Protein DJ-1	PARK7	Q99497	2.0E-04	8.5E-03
IPI00027139	Inositol polyphosphate 1-phosphatase	INPP1	P49441	2.8E-04	8.5E-03
IPI00044369	Isoform 1 of Plexin domain-containing protein 2 precursor	PLXDC2	Q6UX71	3.3E-04	8.5E-03
IPI00465184	Guanine deaminase	GDA	Q9Y2T3	4.8E-04	8.5E-03
IPI00005222	Ephrin type-B receptor 6 precursor	EPHB6	O15197	4.9E-04	8.5E-03
IPI00030255	Procollagen-lysine,2-oxoglutarate 5-dioxygenase 3 precursor	PLOD3	O60568	4.9E-04	8.5E-03
IPI00395667	Interferon-related IFRD2 (PC4-B) protein	NAT6;IFRD2	Q12894	5.1E-04	8.5E-03
IPI00219910	22 kDa protein	-		5.7E-04	8.5E-03
IPI00552905	Isoform 1 of Proline-rich transmembrane protein 3 precursor	PRRT3	Q5FWE3	6.1E-04	8.5E-03
IPI00550991	Alpha-1-antichymotrypsin precursor	SERPINA3	P01011	6.6E-04	8.5E-03
IPI00412264	Pleiotrophin precursor	PTN	P21246	7.1E-04	8.5E-03
IPI00304379	Ubiquitin carboxyl-terminal hydrolase 1	USP1	O94782	7.3E-04	8.5E-03
IPI00418931	CDNA FLJ45139 fis, clone BRAWH3039623	FLJ45139		7.7E-04	8.5E-03
IPI00217778	Isoform 2 of Phospholipid transfer protein precursor	PLTP	P55058	8.7E-04	9.0E-03
IPI00299738	Procollagen C-endopeptidase enhancer 1 precursor	PCOLCE	Q15113	9.5E-04	9.3E-03
IPI00292950	Serpin peptidase inhibitor, clade D (Heparin cofactor), member 1	SERPIND1		1.2E-03	1.1E-02
IPI00298281	Laminin subunit gamma-1 precursor	LAMC1	P11047	1.3E-03	1.1E-02
IPI00218732	Serum paraoxonase/arylesterase 1	PON1	P27169	1.4E-03	1.1E-02
IPI00386946	CDNA FLJ11786 fis, clone HEMBA1006036	CCDC90A		1.4E-03	1.1E-02

Table S3. Proteins (n=59) that were quantified and determined to be significantly different in abundance by ANOVA (p ≤ 0.01) when comparing *CFS* from *nPTLS* subject samples and allow for separation of these two syndromes when performing unsupervised hierarchical cluster analysis.

IPI	Protein name	Gene symbol	Swiss Prot ID	p value	q value
IPI00029235	Insulin-like growth factor-binding protein 6 precursor	IGFBP6	P24592	1.5E-03	1.1E-02
IPI00026314	Isoform 1 of Gelsolin precursor	GSN	P06396	1.6E-03	1.1E-02
IPI00301865	Isoform 1 of Transmembrane protein 132A precursor	TMEM132A	Q24JP5	1.6E-03	1.1E-02
IPI00018136	Isoform 1 of Vascular cell adhesion protein 1 precursor	VCAM1	P19320	1.7E-03	1.1E-02
IPI00009477	Intercellular adhesion molecule 2 precursor	ICAM2	P13598	1.8E-03	1.1E-02
IPI00787853	Inositol monophosphatase 3	IMPAD1	Q9NX62	1.8E-03	1.1E-02
IPI00413272	Isoform 3 of Mediator of RNA polymerase II transcription subunit 23	MED23	Q9ULK4	1.9E-03	1.1E-02
IPI00219219	Galectin-1	LGALS1	P09382	1.9E-03	1.1E-02
IPI00410600	Isoform 3 of Voltage-dependent calcium channel subunit alpha-2/delta-2 precursor	CACNA2D2	Q9NY47	2.1E-03	1.2E-02
IPI00216691	Profilin-1	PFN1	P07737	2.2E-03	1.2E-02
IPI00216250	Cell recognition protein CASPR4	CNTNAP4	Q9C0A0	2.5E-03	1.3E-02
IPI00401776	mucin 6, gastric	MUC6	Q6W4X9	2.6E-03	1.3E-02
IPI00032292	Metalloproteinase inhibitor 1 precursor	TIMP1	P01033	2.6E-03	1.3E-02
IPI00418163	C4B1	C4B		2.7E-03	1.3E-02
IPI00654875	Complement C4-B precursor	C4B	P0C0L5	2.7E-03	1.3E-02
IPI00029260	Monocyte differentiation antigen CD14 precursor	CD14	P08571	2.9E-03	1.4E-02
IPI00022394	Complement C1q subcomponent subunit C precursor	C1QC	P02747	3.2E-03	1.4E-02
IPI00029061	Selenoprotein P precursor	SEPP1	P49908	3.3E-03	1.4E-02
IPI00335343	similar to Dynein heavy chain at 36C CG5526-PA	DNAH14		3.3E-03	1.4E-02
IPI00746623	Hyaluronan-binding protein 2 precursor	HABP2	Q14520	4.0E-03	1.7E-02
IPI00162549	SP110 nuclear body protein isoform c	SP110	Q9HB58	4.1E-03	1.7E-02
IPI00001895	Isoform 1 of Protocadherin-8 precursor	PCDH8	O95206	5.4E-03	2.2E-02
IPI00217519	Ras-related protein Ral-A precursor	RALA	P11233	5.7E-03	2.3E-02

Table S3. Proteins (n=59) that were quantified and determined to be significantly different in abundance by ANOVA (p ≤ 0.01) when comparing *CFS* from *nPTLS* subject samples and allow for separation of these two syndromes when performing unsupervised hierarchical cluster analysis.

IPI	Protein name	Gene symbol	Swiss Prot ID	p value	q value
IPI00301494	Carbohydrate sulfotransferase 4	CHST4	Q8NCG5	6.4E-03	2.3E-02
IPI00297284	Insulin-like growth factor-binding protein 2 precursor	IGFBP2	P18065	6.5E-03	2.3E-02
IPI00657936	collagen, type XXVIII precursor	COL28A1		6.5E-03	2.3E-02
IPI00000977	Mitogen-activated protein kinase kinase kinase 11	MAP3K11	Q16584	6.5E-03	2.3E-02
IPI00293539	Isoform 2 of Cadherin-11 precursor	CDH11	P55287	8.3E-03	2.9E-02
IPI00784519	Putative uncharacterized protein	-		8.6E-03	3.0E-02
IPI00003865	Isoform 1 of Heat shock cognate 71 kDa protein	HSPA8	P11142	8.9E-03	3.0E-02
IPI00477992	complement component 1, q subcomponent, B chain precursor	C1QB	P02746	9.4E-03	3.0E-02
IPI00011261	Complement component C8 gamma chain precursor	C8G	P07360	9.5E-03	3.0E-02
IPI00550162	IGLV3-25 protein	IGLV3-25		9.5E-03	3.0E-02
IPI00017696	Complement C1s subcomponent precursor	C1S	P09871	9.5E-03	3.0E-02
IPI00552771	V2-11 protein	IGLV3-16		9.5E-03	3.0E-02
IPI00218413	biotinidase precursor	BTD	P43251	9.8E-03	3.0E-02
IPI00376436	Isoform 4 of Vacuolar protein sorting-associated protein 13B	VPS13B	Q7Z7G8	1.0E-02	3.0E-02
