# Supplementary material for: Distinct Cerebrospinal Fluid Proteomes Differentiate Post-Treatment Lyme Disease from Chronic Fatigue Syndrome
Source: PLoS One. 2011 Feb 23;6(2):e17287. doi: 10.1371/journal.pone.0017287 (PMC3044169; doi:10.1371/journal.pone.0017287)
Supplement: Table S4 — Pathway enrichment determination using Ingenuity pathways analysis tools for proteins present in nPTLS and CFS proteomes. Analysis of proteins detected in the highly fractionated, immunodepleted, pooled CSF samples led to the identification of pathways that are significantly enriched (p ≤ 0.05) by the proteins from the CSF proteomes. (PDF) [file pone.0017287.s006.pdf]

Table S4. Pathway enrichment determination using Ingenuity pathway analysis tools for proteins present in *nPTLS* and *CFS* proteomes. Analysis of proteins detected in the highly fractionated immunodepleted pooled CSF samples led to the identification of pathways that are significantly enriched (p ≤ 0.05) by the proteins from the CSF proteomes.

**nPTLS**	-log (p value)	coverage(%)
Complement System	19.2	77.8
Coagulation System	12.6	64.9
N-Glycan Degradation	8.13	56.2
Lipid Antigen Presentation by CD1	2	31.8
Acute Phase Response Signaling	14.7	34.8
Parkinson's Signaling	2.45	41.2
O-Glycan Biosynthesis	2.8	23.4
Hepatic Fibrosis / Hepatic Stellate Cell Activation	7.83	30.6
Semaphorin Signaling in Neurons	1.88	25
Notch Signaling	1.7	23.3
Axonal Guidance Signaling	16.3	26.6
Neuroprotective Role of THOP1 in Alzheimer's Disease	4.12	27.8
Glycosphingolipid Biosynthesis - Globoseries	2.6	21.7
IGF-1 Signaling	2.91	23
Keratan Sulfate Biosynthesis	2.21	25.5
Ephrin Receptor Signaling	2.7	18.4
Virus Entry via Endocytic Pathways	1.9	19.8
N-Glycan Biosynthesis	2.47	16.1
VDR/RXR Activation	2.08	22.5
14-3-3-mediated Signaling	1.62	19.3
Glycosaminoglycan Degradation	1.35	13.9
Clathrin-mediated Endocytosis Signaling	1.39	16.8
Glycolysis/Gluconeogenesis	2.53	16.2
LXR/RXR Activation	1.54	17.4
Glutathione Metabolism	1.44	14.3
Human Embryonic Stem Cell Pluripotency	2	17.6
Role of Macrophages, Fibroblasts and Endothelial Cells in Rheumatoid Arthritis	1.94	16.4
Glycosphingolipid Biosynthesis - Globoseries	2.6	21.7
Lysine Degradation	3.15	15
Pentose Phosphate Pathway	1.7	11.2
Cysteine Metabolism	2.21	15.6
Phenylalanine, Tyrosine and Tryptophan Biosynthesis	2.28	10.4
Pyruvate Metabolism	1.59	11.4
Methane Metabolism	1.93	9.09
Nitrogen Metabolism	2.18	9.77
Phenylalanine Metabolism	1.87	9.17

Table S4. Pathway enrichment determination using Ingenuity pathway analysis tools for proteins present in nPTLS and CF proteomes. Analysis of proteins detected in the highly fractionated immunodepleted pooled CSF samples led to the identification of pathways that are significantly enriched ($p \leq 0.05$) by the proteins from the CSF proteomes.

CFS	-log (p value)	coverage(%)
Complement System	21.00	72.2
Coagulation System	14.30	59.5
N-Glycan Degradation	7.98	46.9
Lipid Antigen Presentation by CD1	3.06	31.8
Acute Phase Response Signaling	18.60	30.9
Parkinson's Signaling	1.95	29.4
O-Glycan Biosynthesis	5.24	25.5
Hepatic Fibrosis / Hepatic Stellate Cell Activation	6.16	21.6
Semaphorin Signaling in Neurons	2.45	21.2
Notch Signaling	2.44	20.9
Axonal Guidance Signaling	16.60	20.6
Neuroprotective Role of THOP1 in Alzheimer's Disease	3.46	20.4
Glycosphingolipid Biosynthesis - Globoseries	3.35	19.6
Agrin Interactions at Neuromuscular Junction	2.39	18.8
Myc Mediated Apoptosis Signaling	1.91	17.5
IGF-1 Signaling	2.79	17
Keratan Sulfate Biosynthesis	1.49	16.4
Ephrin Receptor Signaling	4.42	15.8
Virus Entry via Endocytic Pathways	2.28	15.6
Atherosclerosis Signaling	2.44	15.2
N-Glycan Biosynthesis	3.85	15.1
VDR/RXR Activation	1.56	15
14-3-3-mediated Signaling	1.65	14
Glycosaminoglycan Degradation	2.59	13.9
Clathrin-mediated Endocytosis Signaling	2.25	13.8
Glycolysis/Gluconeogenesis	3.31	13.4
LXR/RXR Activation	1.54	12.8
Glutathione Metabolism	2.12	12.2
Human Embryonic Stem Cell Pluripotency	1.72	12.2
Role of Macrophages, Fibroblasts and Endothelial Cells in Rheumatoid Arthritis	1.49	10.9
Glycosphingolipid Biosynthesis - Ganglioseries	1.67	10.8
Lysine Degradation	2.86	10.7
Pentose Phosphate Pathway	2.44	10.1
Cysteine Metabolism	1.49	10

Phenylalanine, Tyrosine and Tryptophan Biosynthesis	2.56	8.96
Pyruvate Metabolism	1.79	8.72
Galactose Metabolism	1.40	7.83
Methane Metabolism	2.07	7.58
Nitrogen Metabolism	1.75	6.77
Phenylalanine Metabolism	1.54	6.42
