# Supplementary material for: Distinct Cerebrospinal Fluid Proteomes Differentiate Post-Treatment Lyme Disease from Chronic Fatigue Syndrome
Source: PLoS One. 2011 Feb 23;6(2):e17287. doi: 10.1371/journal.pone.0017287 (PMC3044169; doi:10.1371/journal.pone.0017287)
Supplement: Table S5 — Pathways significantly enriched by proteins identified only in the pooled sample proteome for nPTLS patients. (PDF) [file pone.0017287.s007.pdf]

Table S5. Pathway analysis for proteins identified only in *nPTLS* proteome.

**Ingenuity Canonical Pathways**	**-log(p-value)**
Glycine, Serine and Threonine Metabolism	3.46
Glioblastoma Multiforme Signaling	3.38
AMPK Signaling	3.25
Melatonin Signaling	2.99
Molecular Mechanisms of Cancer	2.60
p70S6K Signaling	2.47
Factors Promoting Cardiogenesis in Vertebrates	1.97
Ovarian Cancer Signaling	1.89
Bile Acid Biosynthesis	1.89
Regulation of eIF4 and p70S6K Signaling	1.87
Propanoate Metabolism	1.86
Cardiac Hypertrophy Signaling	1.80
Neuregulin Signaling	1.76
Chemokine Signaling	1.71
Inositol Phosphate Metabolism	1.69
Î²-alanine Metabolism	1.68
mTOR Signaling	1.66
Thrombin Signaling	1.58
PPARÎ±/RXRÎ± Activation	1.57
Aldosterone Signaling in Epithelial Cells	1.53
GNRH Signaling	1.52
Aminoacyl-tRNA Biosynthesis	1.51
Butanoate Metabolism	1.42
Insulin Receptor Signaling	1.38
Cellular Effects of Sildenafil (Viagra)	1.37
CCR3 Signaling in Eosinophils	1.36
Role of NFAT in Cardiac Hypertrophy	1.35
Synthesis and Degradation of Ketone Bodies	1.32
