# Supplementary material for: Distinct Cerebrospinal Fluid Proteomes Differentiate Post-Treatment Lyme Disease from Chronic Fatigue Syndrome
Source: PLoS One. 2011 Feb 23;6(2):e17287. doi: 10.1371/journal.pone.0017287 (PMC3044169; doi:10.1371/journal.pone.0017287)
Supplement: Table S6 — Pathways significantly enriched by proteins identified only in the pooled sample proteome for CFS patients. (PDF) [file pone.0017287.s008.pdf]

Table S6. Pathways significantly enriched by proteins identified only in the pooled sample proteome for *CFS* patients.

Ingenuity Canonical Pathways	-log(p-value)
CDK5 Signaling	4.06
Cardiac beta-adrenergic Signaling	3.40
Dopamine Receptor Signaling	3.30
Protein Kinase A Signaling	2.58
Relaxin Signaling	2.21
Cellular Effects of Sildenafil (Viagra)	2.07
Clathrin-mediated Endocytosis Signaling	2.00
cAMP-mediated Signaling	2.00
Breast Cancer Regulation by Stathmin1	1.88
Sphingosine-1-phosphate Signaling	1.84
Hepatic Fibrosis / Hepatic Stellate Cell Activation	1.70
Î±-Adrenergic Signaling	1.68
Valine, Leucine and Isoleucine Degradation	1.67
Production of Nitric Oxide and Reactive Oxygen Species in Macrophages	1.66
IL-1 Signaling	1.61
Leptin Signaling in Obesity	1.56
G-Protein Coupled Receptor Signaling	1.56
Role of NFAT in Cardiac Hypertrophy	1.56
GÎ±12/13 Signaling	1.52
Actin Cytoskeleton Signaling	1.47
IL-8 Signaling	1.43
CREB Signaling in Neurons	1.43
Synaptic Long Term Potentiation	1.36
CXCR4 Signaling	1.31
